# Multitarget-Directed Gallium(III) Tris(acyl-pyrazolonate) Complexes Induce Ferroptosis in Cancer Cells via Dysregulation of Cell Redox Homeostasis and Inhibition of the Mevalonate Pathway

**DOI:** 10.1021/acs.jmedchem.2c01374

**Published:** 2023-02-21

**Authors:** Daphne Romani, Fabio Marchetti, Corrado Di Nicola, Massimiliano Cuccioloni, Chunmei Gong, Anna Maria Eleuteri, Agustín Galindo, Farzaneh Fadaei-Tirani, Massimo Nabissi, Riccardo Pettinari

**Affiliations:** ^†^School of Science and Technology, ^‡^School of Biosciences and Biotechnology, ^§^School of Pharmacy, University of Camerino, Via Madonna delle Carceri (ChIP), 62032 Camerino MC, Italy; ∥ Departamento de Química Inorgánica, Facultad de Química, Universidad de Sevilla, Aptdo 1203, 41071 Sevilla, Spain; ⊥ Institut of Chemical Sciences and Engineering, Swiss Federal Institute of Technology Lausanne (EPFL), Lausanne CH-1015, Switzerland

## Abstract

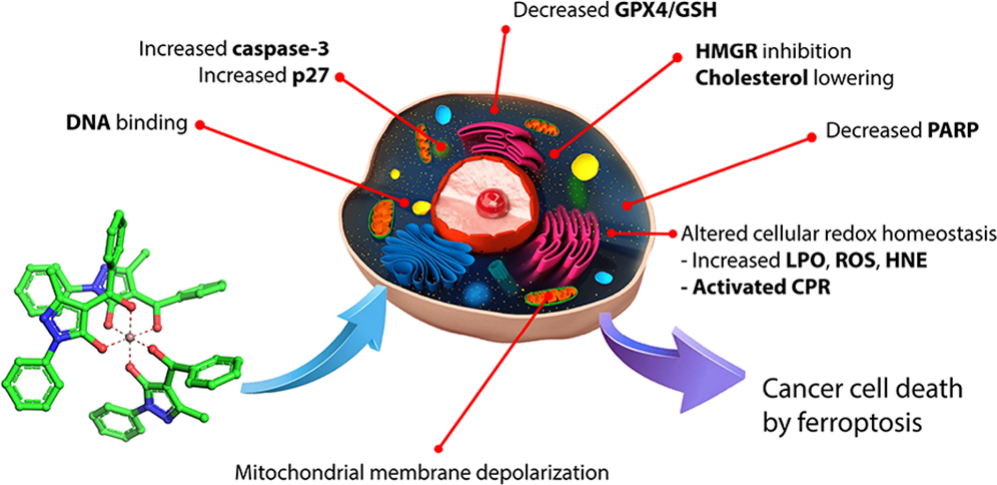

A series of Ga(Q*_n_*)_3_ coordination compounds have been synthesized, where HQ*_n_* is 1-phenyl-3-methyl-4-RC(=O)-pyrazolo-5-one. The complexes have been characterized through analytical data, NMR and IR spectroscopy, ESI mass spectrometry, elemental analysis, X-ray crystallography, and density functional theory (DFT) studies. Cytotoxic activity against a panel of human cancer cell lines was determined by the 3-(4,5-dimethylthiazol-2-yl)-2,5-diphenyltetrazolium bromide (MTT) assay, with interesting results in terms of both cell line selectivity and toxicity values compared with cisplatin. The mechanism of action was explored by spectrophotometric, fluorometric, chromatographic, immunometric, and cytofluorimetric assays, SPR biosensor binding studies, and cell-based experiments. Cell treatment with gallium(III) complexes promoted several cell death triggering signals (accumulation of p27, PCNA, PARP fragments, activation of the caspase cascade, and inhibition of the mevalonate pathway) and induced changes in cell redox homeostasis (decreased levels of GSH/GPX4 and NADP(H), increased reactive oxygen species (ROS) and 4-hydroxynonenal (HNE), mitochondrial damage, and increased activity of CPR and CcO), identifying ferroptosis as the mechanism responsible for cancer cell death.

## Introduction

In the last few years, organometallic compounds have attracted increasing interest as anticancer drugs.^1^ In fact, compared with organic molecules, they synergistically combine the properties of both the metal ion and the organic ligand, enabling the design/synthesis of promising multifunctional therapeutic agents.^2^ In this context, metallocenes, half-sandwich complexes, carbonyl complexes, and carbene derivatives of transition metals are some of the most thoroughly characterized organometallic species that have been exploited for therapeutic purposes.^3^ In particular, the quest for coordination compounds with anticancer therapeutic properties and the ability to overcome the limits associated with long-established metal-based drugs (i.e., cisplatin) stimulated the development of novel cytotoxic complexes of Ag, Au, Co, Cu, Ru, Rh, Sn, Ti, and Ga.^4^ Among these metals, Ga(III) complexes have shown promising results as antineoplastic, anti-inflammatory, antibacterial, and antihypercalcemic agents;^5^ most notably, gallium maltolate^6^ and KP46^7^ are currently in clinical trials for the treatment of hepatocellular carcinoma and renal cancer, respectively. Although the mechanism behind the biological effects of gallium(III)-based compounds remains elusive, the high similarity with iron(III) in terms of the electric charge, ion diameter, coordination number, and electronic configuration is critical.^8^ In fact, Ga^3+^ is able to replace Fe^3+^ in different metalloenzyme and proteins that exploit the redox chemistry of iron, but since Ga(III) cannot be reduced under physiological conditions, it may block those biochemical pathways.^9^ To further enhance the pharmacological and biological properties of the metal, several gallium-based complexes were synthesized in the last few years.^10^ Given the long-term experience of our research group on the study of pyrazolone-based ligands as anticancer and antibacterial agents,^11,12^ we investigated the effect of the coordination of these molecules with Ga(III). In fact, the pyrazolone scaffold is a pharmacophore of many chemotherapeutics,^13^ this core molecule being associated with a broad spectrum of biological activities, such as antibacterial, analgesic, antioxidant, and (mainly) antitumoral. Its general mechanisms of action involve several biological pathways and trigger different apoptotic events, like the stimulation of ROS, the activation of the caspase-3 cascade, and the decrease of the mitochondrial membrane potential.^14^ Here, we report on the syntheses, structural characterization, and evaluation of anticancer activities of five novel complexes obtained by the reaction between gallium(III) nitrate and different 4-acyl-3-methyl-1-phenyl-5-pyrazolone derivatives. Specifically, we tested the cytotoxicity of these complexes against breast adenocarcinoma (MCF-7), colorectal carcinoma (HCT-116, Caco-2 along with its cisplatin-resistant counterpart, namely Caco-2CR), and hepatocarcinoma (HepG2), and we measured their selectivity compared with noncancerous cells, with a specific emphasis toward the exploration of the molecular mechanisms of action behind the observed effects.

## Results and Discussion

**Figure 1:**
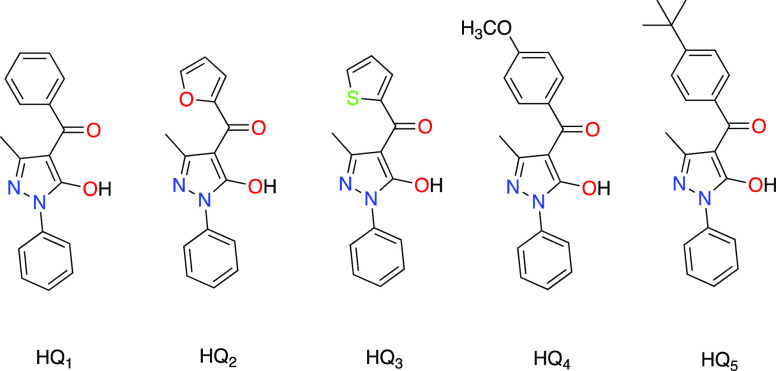
Proligands HQ*_n_* employed in this work.

Steric and electronic effects of the ligands can influence the chemical–physical characteristics of the respective gallium complexes and, in turn, modify their biological activity. Based on these considerations, acylpyrazolones with a phenyl (HQ_1_), a furan (HQ_2_), and a thiophene (HQ_3_) in the acyl fragment were chosen. Furthermore, two substituents were introduced on the phenyl ring, both with an electronic release such as methoxide (HQ_4_) and tert-butyl (HQ_5_), but with different steric hindrance and lipophilicity. The proligands HQ_1_-HQ_5_ (Figure 1) (HQ_1_ = (5-hydroxy-3-methyl-1-phenyl-1H-pyrazol-4-yl)(phenyl)methanone; HQ_2_ = furan-2-yl(5-hydroxy-3-methyl-1-phenyl-1H-pyrazol-4-yl)methanone; HQ_3_ = (5-hydroxy-3-methyl-1-phenyl-1H-pyrazol-4-yl)(thiophen-2-yl)methanone; HQ_4_ = (5-hydroxy-3-methyl-1-phenyl-1H-pyrazol-4-yl)(4-methoxyphenyl)methanone; and HQ_5_ = (4-(tert-butyl)phenyl)(5-hydroxy-3-methyl-1-phenyl-1H-pyrazol-4-yl)methanone) were prepared as described previously.^11^

**Scheme 1:**
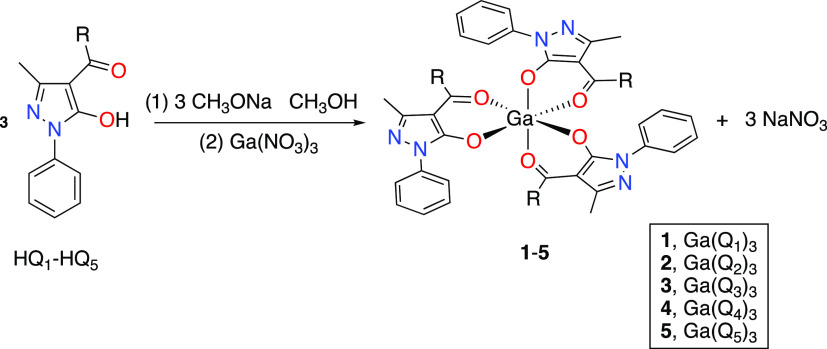
Synthesis of Complexes **1**–**5**

The novel gallium(III) complexes **1**–**5** were prepared in a high yield by reacting Ga(NO_3_)_3_ and the appropriate proligand and deprotonating with sodium methoxide in methanol at room temperature (Scheme 1).

**Figure 2:**
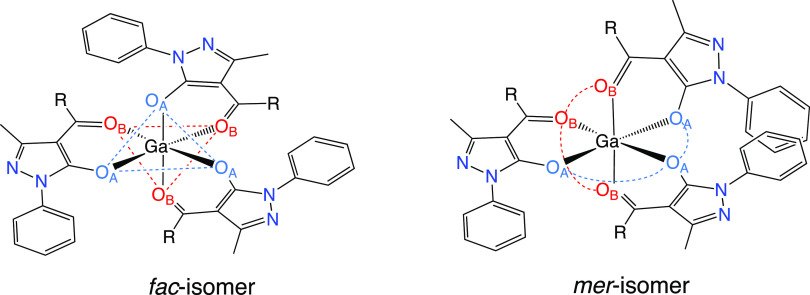
Facial (*fac-*) and meridional (*mer-*) isomers for complexes **1**–**5**.

The **1**–**5** derivatives are pale pink-yellow solids with sharp melting points, soluble in acetone, acetonitrile, chlorinated solvents, DMSO, and DMF, and slightly soluble in alcohols. In the IR spectra of all complexes **1–5**, both the disappearance of the broad band of the (O–H···O) system, present in the range of 2300–3200 cm^–1^ in the neutral HQ*_n_* proligands, and the shift to lower frequencies of the ν(C=O) vibration are in accordance with coordination of the ligands to the Ga^3+^ ion in the O,O′-bidentate chelating mode. It is interesting to note that the far-IR spectra of complexes **1**–**5** show absorption in the range of 666–621 cm^–1^, which may be assigned to ν(Ga–O) stretches.^15^ For complexes **1**–**5**, two geometric isomers could be obtained, the facial (*fac-*) with C3v symmetry and the meridional (*mer-*) with C1 symmetry. Each isomer leads to further two Δ and Λ optical isomers (Figure 2).

In the ^1^H NMR spectra of complexes **1**–**5** in CDCl_3_, four sets of signals were observed; three of these signals are due to the *mer-* configuration, where all ligands are spatially different and chemically nonequivalent, while the more symmetrical *fac-* isomer generates a single signal because all ligands are equivalent. Theoretical calculations confirm that the energy difference between the *fac* and *mer* isomers is less than 1 kcal/mol. This value fits well with the isolation of both isomers and the existence of both in the NMR spectra.

### X-ray Crystallography and Density Functional Theory Calculations

**Figure 3:**
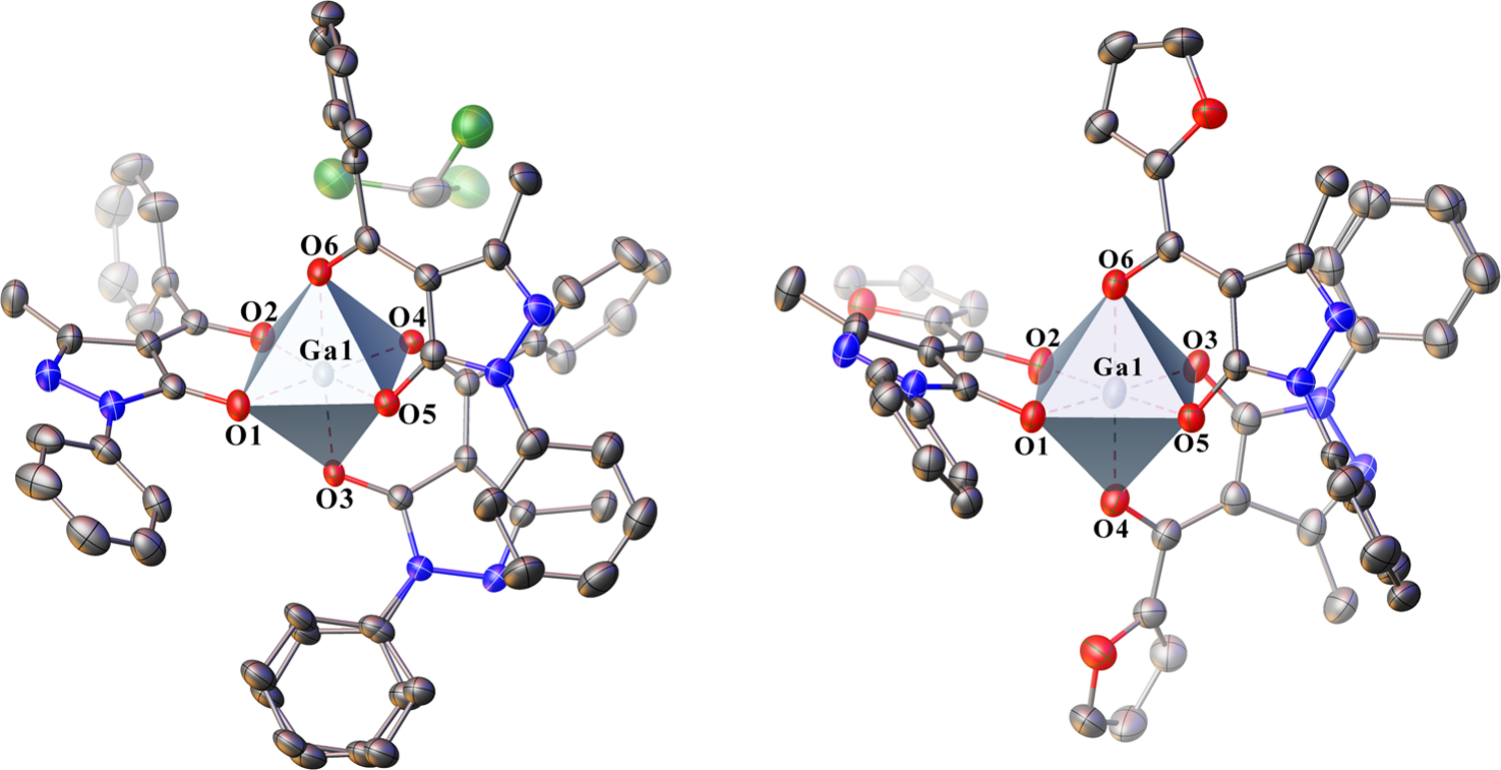
Structures of complexes **1** (left, CCDC number 2190069) and **2** (right, CCDC number 2190070). Displacement ellipsoids are drawn at the 50% probability level. Hydrogen atoms are omitted for clarity.

The X-ray crystal structures of complexes **1** and **2** exhibit a slightly distorted octahedral coordination geometry (Figure 3), both containing Δ and Λ isomers (Tables S10, S11, S23 and S24). The structure of **1** owes its cohesion to a large number of nonclassical hydrogen bonds and Cl···π and van der Waals interactions, similarly in the structure of **2** showing nonclassical hydrogen bonds and van der Waals and also π–π interactions. To check the influence of the different R substituents on the *fac*-/*mer*- isomer energies in complexes **1**–**5**, density functional theory calculations were performed at the B3LYP/6-31G* level of theory to obtain the optimized structures of the *fac*-/*mer*- isomers and their corresponding energies (see Tables S1 and S2 and Figure S32). The calculated energy differences between the *fac*- and *mer*- isomers [Ga(Q*_n_*)_3_] are very small (|Δ*G*| less than 1 kcal mol^–1^) and the different R groups of the acylpyrazolonate Q*_n_*^–^ ligands do not have any influence on these energy differences. Therefore, the two isomers can exist in solution, in agreement with the observed NMR data.^16^ However, the observed experimental *mer-*/*fac-* ratio is not 1:1 but approximately 3:1, despite the similar energy found for both isomers. For this reason, we studied the last steps of the formation of the Ga(Q_1_)_3_ complex, hypothesizing a stepwise substitution of NO_3_^–^ by Q_1_^–^ on the gallium coordination sphere from the starting Ga(NO_3_)_3_ toward the hypothetical Ga(NO_3_)(Q_1_)_2_ intermediate. Three stereoisomers, **I**, **II**, and **III**, are possible for such a pseudo-octahedral Ga(NO_3_)(Q_1_)_2_ intermediate (Figure S35), with again very small (|Δ*G*| less than 1.5 kcal mol^–1^) energy differences between them. In the last step to complex **1**, with substitution of NO_3_^–^ by the third Q_1_^–^, of the three stereoisomers **I**, **II**, and **III**, only **II** would afford the *fac*- isomer (Figure S36). This substitution occurs through the Ga(κ^1^-NO_3_)(Q_1_)_3_^–^ intermediate, in which NO_3_^–^ leaves a vacant coordination position that is occupied by the Q_1_^–^ ligand (Figure S37). This proposal would afford the 3:1 ratio experimentally observed in complexes **1**–**5**, which is the typical *mer*-/*fac*- ratio found in related tris chelate metal complexes.^17^

#### Stability Studies

**Figure 4:**
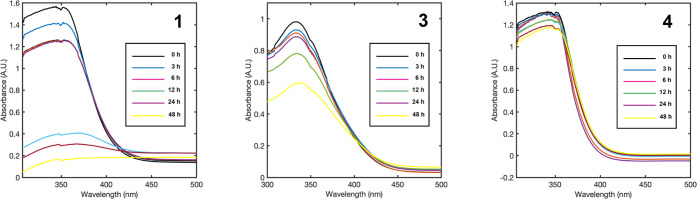
UV–vis spectra of complexes **1**, **3**, and **4** in a 1% DMSO–PBS solution at room temperature.

The stability of Ga(III) complexes was examined both via NMR and UV/vis spectroscopy. A series of ^1^H-NMR spectra were recorded in the DMSO-*d*_6_ solution over a period of 48 h, based on the incubation time of the cells together with the treatment in the MTT assay. Briefly, no variation of the δ values of the characteristic peaks is noticeable, meaning that all of the complexes are overall stable in DMSO (Figure S31). This was further confirmed by the UV/vis spectrum, which remained unchanged for a period of 48 h. Subsequently, the stability of the complexes was evaluated in the phosphate buffer solution (PBS, pH = 7.4) to simulate physiological conditions. Compounds **1**, **3**, and **4** were initially solubilized in DMSO at room temperature (0.85, 0.9, and 1 mg/mL, respectively) and then diluted to 1% DMSO with PBS. The absorbance spectra were collected after 0, 3, 6, 12, 24, and 48 h. As shown in Figure 4, complex **4** was found to be the most stable under physiological-like conditions, with a negligible decrease in the trend over 48 h. Complexes **3** and **1** show similar behavior, with a more evident decrease in absorption over time, meaning that the ionic species in the PBS solution can affect the coordination and structural arrangement of the compounds.

#### Cell Internalization

**Figure 5:**
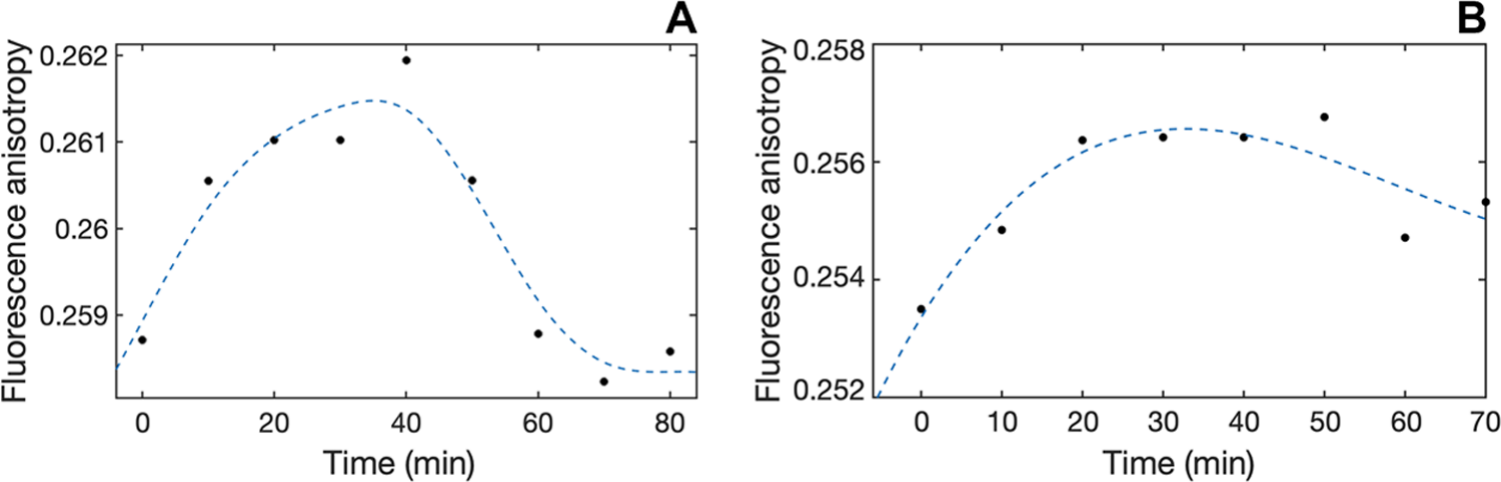
Comparative changes in emission anisotropy with time observed upon the cell membrane passage of **4** (A) and **2** (B).

The ability of complexes **1**–**5** to pass across cell membranes was evaluated using a trimethylammonium diphenylhexatriene (TMA–DPH) fluorescent probe, as previously reported. Qualitatively, with the only exception of **3**, which showed peculiar kinetics, all complexes generally showed a comparable three-stage drug internalization trend in Caco-2 cells, consisting of an initial increase in emission anisotropy (membrane entry), followed by a variably long steady phase (permanence in membranes), and the restoring of the initial conditions (membrane exit) but with major quantitative differences in the rate of individual stages (Figures 5 and S46–S50 and Table S29). The degree of lipophilicity differently affected the passage across membranes: specifically, **1** and **5** (the complexes associated with the highest lipophilicity, Table S30) showed high values of *k*_in_ (faster entry rate in the membrane) but low values of *k*_out_ (slower release rate from the membrane); conversely, **2** and **4** showed an opposite behavior, with low values of *k*_in_ and higher values of *k*_out_. Interestingly, the contribution of the *k*_out_ (hence, a moderate lipophilicity value) was critical to establish the membrane permeability efficacy, with **2** and **4** (in particular) being fully internalized faster than **1** and **5** (Table S29).

#### Effect on Cancer Cell Viability

The cytotoxic activity of gallium complexes **1**–**5** was evaluated against a panel of human cell lines, namely, epithelial breast adenocarcinoma (MCF-7) and their noncancerous counterpart (MCF-10A), hepatocarcinoma (HepG2) and colorectal carcinoma (Caco-2) along with their cisplatin-resistant and noncancerous counterparts (Caco-2CR and CRL-1831, respectively), and HCT-116. The observed cytotoxic effect was time-dependent, peaking at 48 h (Table 1) and remaining unchanged thereafter, and cell line-dependent, with HepG2 and HCT-116 cells being more resistant to the treatments (only complex **1** affected HepG2 viability to a significant extent). In general, complexes **1**, **4**, and **5**, containing a phenyl ring or a para-substituted phenyl ring in the acyl fragment, were the most effective compounds. Focusing on breast cancer cells, complexes **1**, **4**, and **5** showed a promising selectivity for MCF-7 cells, with a 3- to 10-fold lower cytotoxic effect exerted toward the noncancerous MCF-10A counterpart and with potency and a general behavior similar to cisplatin. Likewise, and even more interestingly, all complexes showed both higher cytotoxicity against Caco-2 cells and better selectivity over CLR-1831 noncancerous cells compared with cisplatin. In addition, complexes **1**, **2**, and (mostly) **4** largely retained their cytotoxicity also upon induction of resistance to cisplatin in Caco-2, implying the activation of cell death by a mechanism different from that of cisplatin. For these reasons, Caco-2 cells were used hereafter to dissect the molecular basis of the gallium complexes’ cytotoxicity.

**Table 1:** Cytotoxicity (IC_50_, μM) of Complexes **1**–**5** upon 48 h Treatment of Caco-2 (Colorectal Carcinoma), Caco-2CR (Colorectal Carcinoma, Cisplatin-Resistant), CRL-1831 (Epithelial Normal Colon), MCF-7 (Breast Adenocarcinoma), MCF-10A (Epithelial Normal Breast), HepG2 (Hepatocarcinoma), and HCT-116 (Colorectal Carcinoma) Cell Lines

complex	Caco-2	Caco-2CR	CRL-1831	MCF-7	MCF-10A	HepG2	HCT-116
**1**	8.5 ± 5.8	10.6 ± 4.2	10.2 ± 2.6	2.3 ± 1.0	21.2 ± 9.0	8.5 ± 0.2	>50
**2**	9.9 ± 0.2	35.9 ± 3.6	17.8 ± 6.4	6.6 ± 3.5	15.9 ± 6.0	>50	>50
**3**	17.6 ± 8.9	20.9 ± 3.4	18.7 ± 3.7	7.6 ± 2.6	7.1 ± 4.8	>50	48.0 ± 35.7
**4**	11.0 ± 2.7	12.8 ± 2.1	23.2 ± 6.7	3.5 ± 1.5	21.4 ± 9.0	>50	>50
**5**	8.7 ± 0.4	>50	24.2 ± 5.4	4.1 ± 2.8	21.4 ± 8.0	32.0 ± 1.9	>50
Cisplatin	23.1 ± 5.6	>50	7.7 ± 3.0	4.2 ± 2.3	11.5 ± 4.0	1.4 ± 0.7	15.0 ± 3.0

#### Effect on Cell Redox Homeostasis

Some metal complexes have been demonstrated to exert their anticancer effects by altering cellular redox homeostasis.^18^ Based on this evidence, a number of key redox biomarkers were analyzed to establish the mechanism of cell death induced by complex **4**, which was selected as a representative of the presented Ga complexes because of its good cytotoxicity and selectivity profile against the panel of human cancer cell lines here considered, and also given its higher stability in saline buffers during the 48 h of treatment.

**Figure 6:**
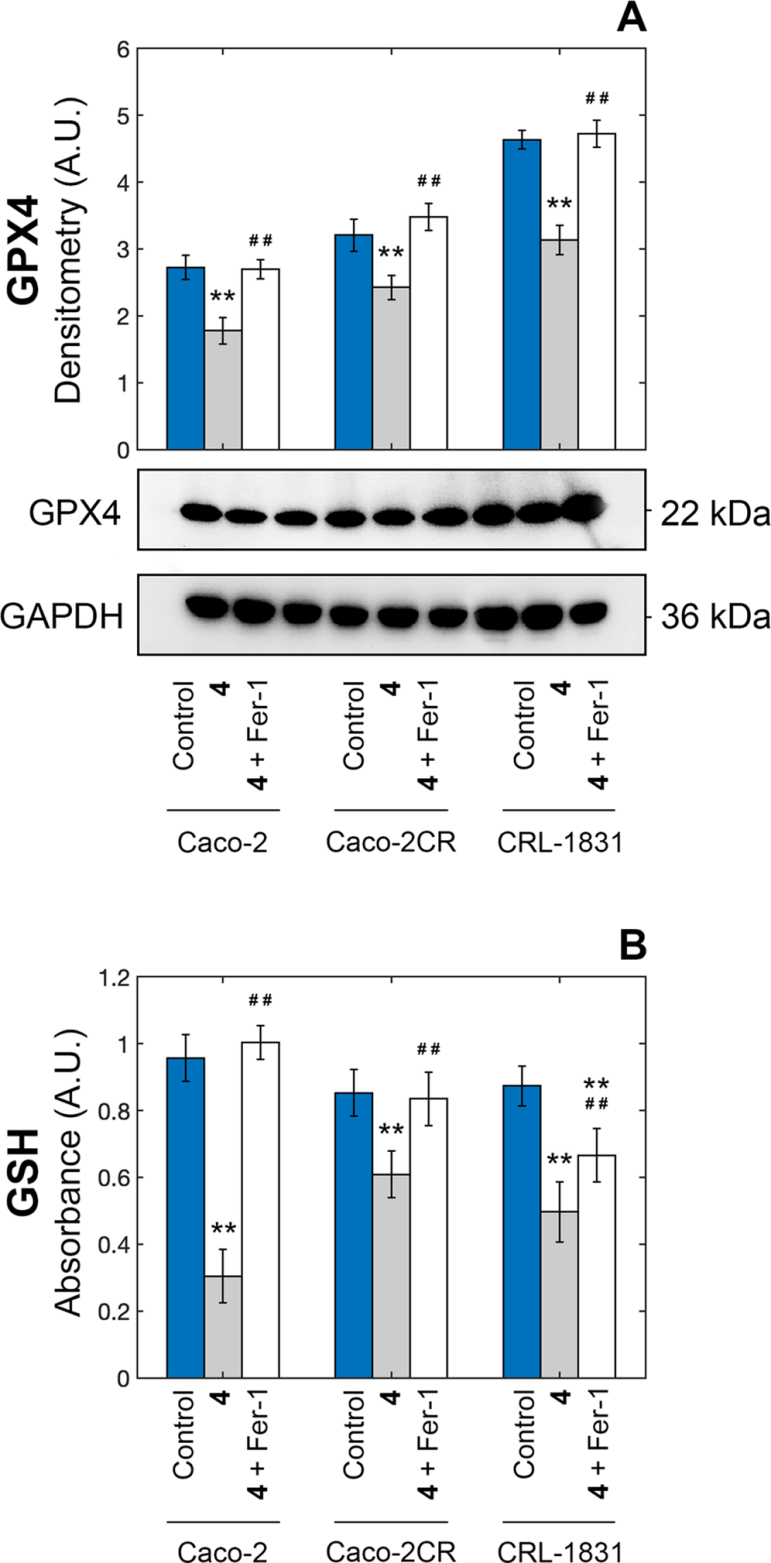
Autoradiographs and densitometric analyses of GPX4 levels (A) in Caco-2 cells and changes in intracellular GSH (B) after treatment with 10 μM of complex **4** for 48 h in the presence and in the absence of 60 nM of ferrostatin-1(*p < 0.05 and **p < 0.01 compared with the control; ^#^*p* < 0.05 and ^##^*p* < 0.01 compared with complex **4**).

First, we focused on the glutathione/glutathione peroxidase 4 (GSH/GPX4) axis, which is pivotal in blocking lipid peroxidation-mediated ferroptosis,^19^ as it can prevent the formation/accumulation of ROS species, and its inhibition/downregulation is a key hallmark for the activation of this form of cell death.^20^ The levels of both GPX4 (Figure 6A) and GSH (Figure 6B) were significantly reduced in Caco-2 cells upon treatment with complex **4**, and also (although to a lower but still significant extent in the case of GSH) in noncancerous and cisplatin-resistant counterparts.

**Figure 7:**
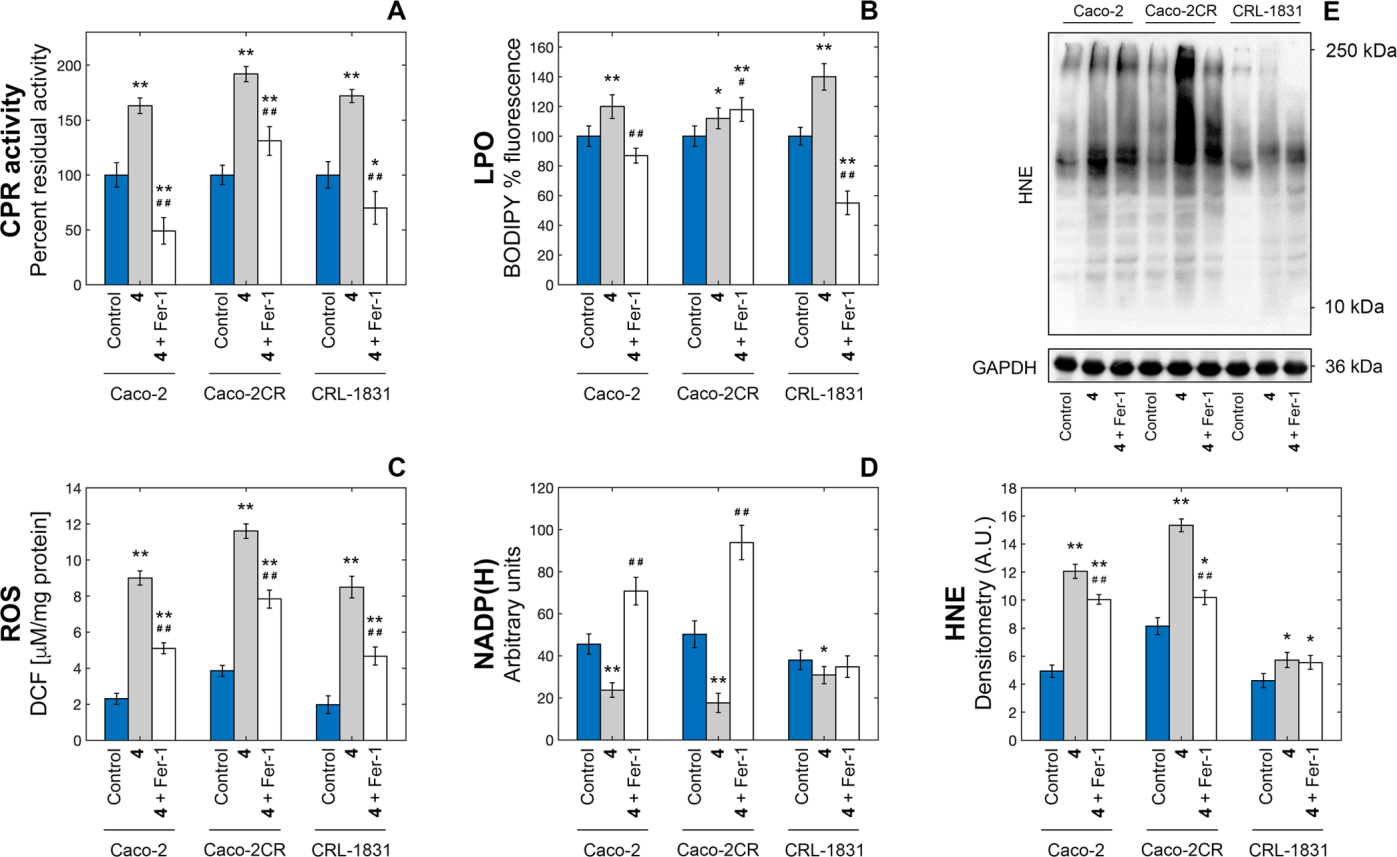
Evaluation of the changes in the expression/activity of selected cell redox biomarkers in Caco-2 cells after treatment with 10 μM of complex **4** for 48 h in the presence and in the absence of 60 nM of ferrostatin-1. Increase in cytochrome p450 reductase activity (A); quantification of intracellular LPO levels based on BODIPY fluorescence (B); quantification of intracellular ROS levels based on the measurement of DCF fluorescence intensity (C); decrease in total NADP(H) levels upon treatment (D); and autoradiographs and densitometric analyses of HNE levels (E) (*p < 0.05 and **p < 0.01 compared with the control; ^#^*p* < 0.05 and ^##^*p* < 0.01 compared with complex **4**).

To check the downstream effects of GPX4 downregulation (and to assess the activation of ferroptosis), the induction of intracellular LPO and ROS on cancer cells was monitored using the BODIPY and DCFH-DA assays, respectively. Specifically, in line with the evidence on the GSH/GPX4 system, we observed a significant increase in LPO and (most evidently) in ROS levels after treatment (Figure 7B,C). In turn, the accumulation of peroxide species caused both a significant increase in 4-hydroxynonenal (HNE) content (Figure 7E), a toxic degradation product of LPO, which may further increase ROS signaling and trigger the mitochondrial caspase signaling pathway, and a mitochondrial depolarization evident at 48 h post-treatment, representative of a strong activity against mitochondrial homeostasis (Figure S58). The loss of NADPH is another consequence of ROS generation during ferroptosis, and because of that, NADPH levels are considered a biomarker of ferroptosis sensitivity/induction in several cancer cell lines.^21^ Consistently, we observed a significant decrease in total NADP(H) content upon 48 h treatment with complex **4** with respect to basal levels (Figure 7D). To further confirm the activation of ferroptosis, Caco-2 cells were cotreated with 60 nM of ferrostatin-1, an established inhibitor of ferroptosis.^22^ Interestingly, upon ferroptosis inhibition, both cell viability (Figure S56) and the levels of the biomarkers not strictly specific to ferroptosis (i.e., total ROS and HNE) were not completely restored, suggesting the induction of other parallel mechanisms of cell death.

#### Effect on NADPH-Cytochrome P450 Reductase Activity

The NADPH-cytochrome P450 reductase (CPR) plays a key role in the regulation of cytochromes P450 (CYP450) activity, catalyzing the electron transfer from NADPH to CYP450 via its FMN and FAD cofactors.^23^ This interplay between CPR and CYP450 is among the major regulators of cellular redox homeostasis.^24^ Upon 48 h treatment of Caco-2 cells with complex **4** in the presence of a specific cytochrome c oxidase (CcO) inhibitor, we observed a significant increase in cellular CPR activity/levels with respect to the control (Figure 7A). This evidence was consistent with the recently established role of CPR as a pro-ferroptotic enzyme that is able to induce lipid peroxidation by accelerating the cycling between Fe(II) and Fe(III) in CYP450.^24^ Additionally, CcO levels were increased (to a higher extent) by the treatment, as confirmed by the results of the same assay performed in the absence of KCN (Figure S54).

#### Other Cell Death Biomarkers

**Figure 8:**
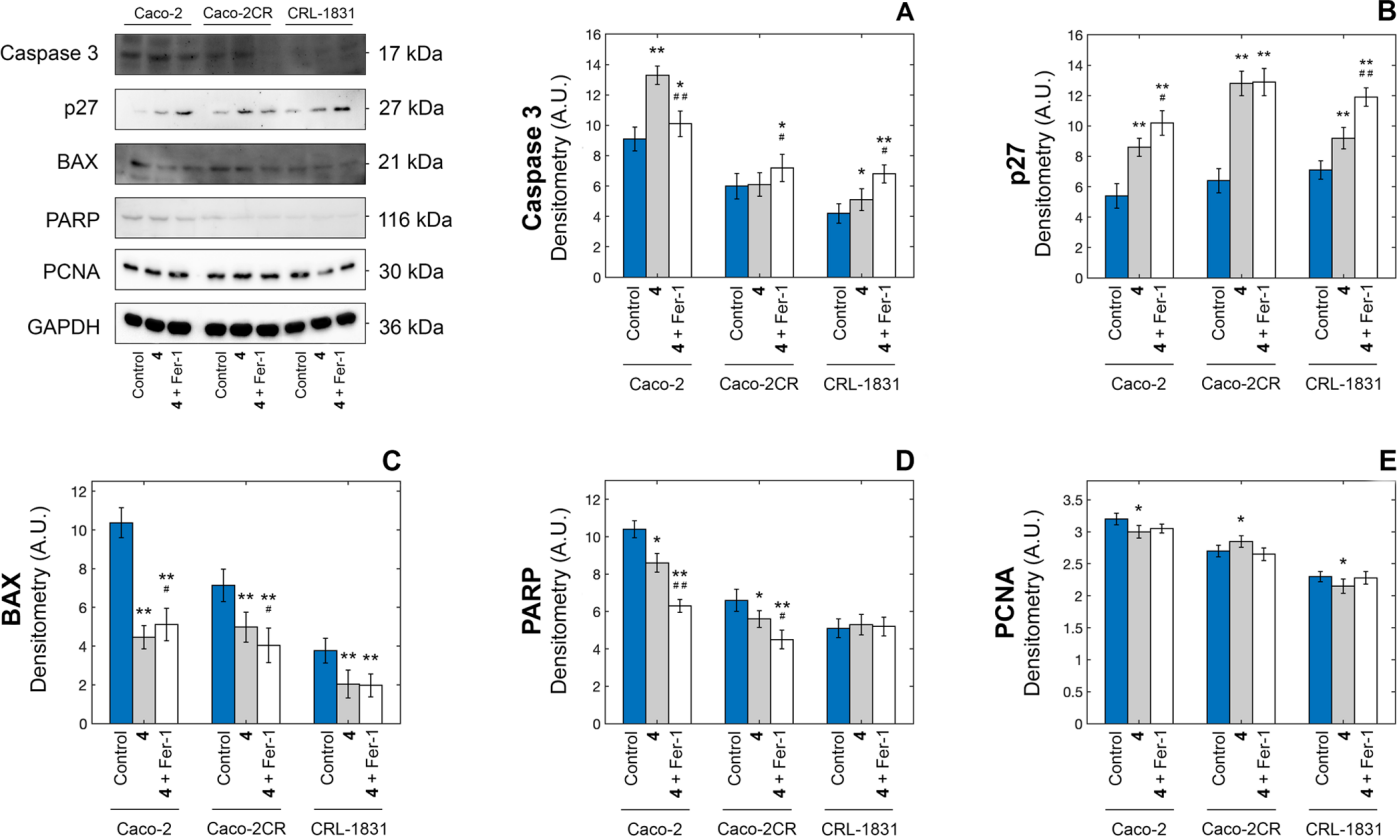
Evaluation of the changes in the expression/activity of selected cell death biomarkers in Caco-2 cells after treatment with 10 μM of complex **4** for 48 h. Autoradiographs and densitometric analysis of caspase-3 (A), p27 (B), BAX (C), full-length 116 kDa PARP (D), and PCNA (E) (*p < 0.05 and **p < 0.01 compared with the control; ^#^*p* < 0.05 and ^##^*p* < 0.01 compared with complex **4**).

Other biomarkers were measured upon treatment with complex **4**. Caspase-3 is a cysteine protease that plays a crucial role in programmed cell death, directly mediating several apoptotic pathways.^25^ Among these, caspase-3 is proven to primarily induce PARP (poly(ADP-ribose) polymerase) cleavage,^26^ directly controlling the DNA repair processes and therefore triggering numerous apoptotic cascade events.^27^ Additionally, high levels of ROS^28^ and oxidative damages associated with dysfunctional mitochondria are among the triggering causes of caspase-3 activation.^29^ The effect on caspase-3 was investigated upon treatment of Caco-2 cells with 10 μM of complex **4** for 48 h, with an observed significant upregulation of both caspase-3 levels (Figure 8A) and activity (Figure S57).

The activation of the caspase-3 cascade was additionally confirmed by the quantification of full-length PARP levels after 48 h treatment with complex **4**. As shown in Figure 8, the treatment significantly lowered active PARP levels (an approx. 40% decrease was observed) compared to the control and DMSO. To further explore the cell death mechanism, we evaluated the levels of p27,^30^ BAX,^31,32^ and PCNA.^33^ As shown in Figure 8, an (proteasome-independent, Figure S55) increase in the level of p27^Kip1^ and a parallel downregulation of BAX were observed after 48 h treatment with compound **4**. In particular, the decrease in active PARP and BAX expression was in line with the activation of ferroptosis and the consequent decrease in apoptosis, further demonstrating the crosstalk between the two cell death pathways.^34,35^ Conversely, PCNA was not significantly affected. Generally, ferrostatin-1 cotreatment did not restore the basal levels of these biomarkers, ruling out any protective effect on parallel cell death mechanisms triggered by complex **4**.

#### Inhibition of HMGR and the Cholesterol-Lowering Effect

**Figure 9:**
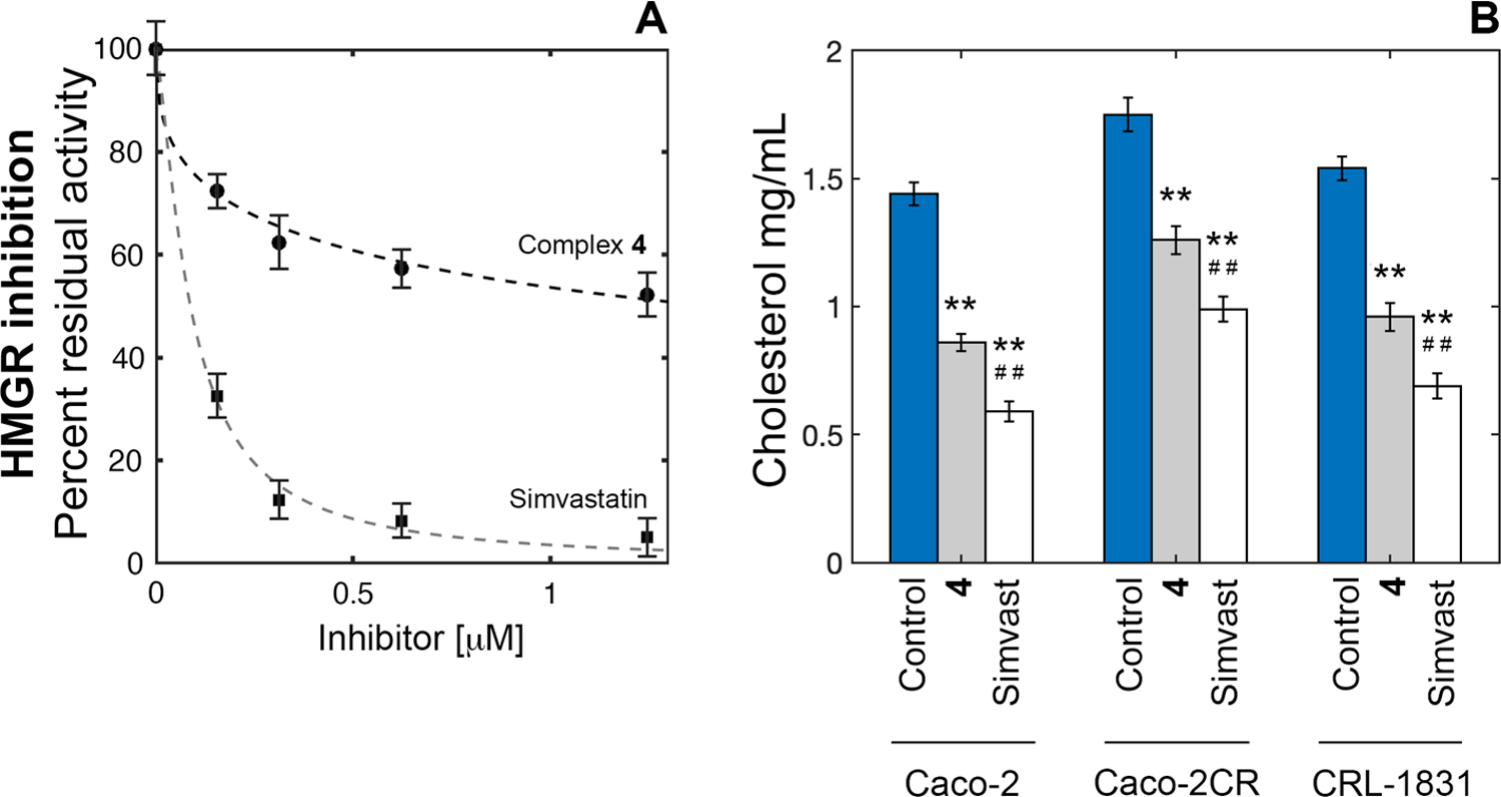
Comparative percent residual activity plot of microsomal HMGR in the presence of increasing levels of complex **4** (●) and Simvastatin (■) (A). Effects of 10 μM of complex **4** and Simvastatin on cellular cholesterol levels (B). Cytoplasmic cholesterol levels were measured in Caco-2, Caco-2CR, and CRL-1831 cells using the AmplexRed Cholesterol Assay kit upon 4 h treatment (*p < 0.05 and **p < 0.01 compared with the control; ^#^*p* < 0.05 and ^##^*p* < 0.01 compared with complex **4**).

The 3-hydroxy-3-methylglutaryl coenzyme A reductase (HMGR) is the rate-limiting enzyme of the mevalonate pathway,^36^ and owing to its involvement in cholesterol and isoprenoid synthesis, it also exerts a regulatory role in cell growth and proliferation, including malignant cells that eagerly demand the end products of this pathway.^37^ Consequently, statins have been promisingly employed in anticancer therapy.^38^ In addition, the disruption of the mevalonate pathway by statins was also recently demonstrated to induce cell death by ferroptosis.^39^ Although according to a different molecular mechanism (Simvastatin competes with HMG-CoA for the binding to HMGR),^40^ complex **4** targeted human HMGR at the NADPH binding site with moderate affinity (Figures S51 and S52) and inhibited the reductase (Figure 9) less potently than Simvastatin, with a *K*_*i*_ = 10 μM (the *K*_*i*_ value was derived from IC_50_).^41^ In line with these results, the cytoplasmic cholesterol concentrations in Caco-2, Caco-2CR, and CRL-1831 cell lines were significantly decreased to a comparable extent upon individual treatment with 10 μM of each molecule for 4 h. Nevertheless, equimolar treatment with statin was always more effective than **4** in reducing cholesterol cytoplasmic levels.

#### Binding to DNA

DNA is among the primary targets for many metal-based drugs.^42^ The interaction between **1**–**5** and DNA was screened according to a biosensor-based approach. All complexes were capable of binding to DNA, generally with monoexponential kinetics upon titration of anchored dsDNA with the Ga complexes in the range of 1–6 μM (Figure S45). This behavior was consistent with the presence of a single binding site on DNA with high affinity (the biexponential model was nonsignificant, as assessed by a standard F-test procedure). All DNA-ligand complexes were characterized by comparable values of equilibrium dissociation constants in the low-to-sub micromolar range (Table 2), the nature of the ligand exerting a negligible effect on DNA binding. Likewise, no major differences in kinetic parameters were observed as well. Additionally, the binding interface on DNA was mapped by exploiting the competition of **1**–**5** with DAPI (4′,6-diamidino-2-phenylindole dihydrochloride) and methyl green for DNA minor and major grooves, respectively. Consistently with the monophasic nature of the interaction, all compounds preferentially bound DNA at the major groove, as proved by the concentration-dependent decrease in absorbance intensity of the DNA–Methyl Green complex. Conversely, only a minor decrease in fluorescence of the DNA–DAPI complex (at the highest concentrations tested) was occasionally observed (Figures S43 and S44).

**Table 2:** Kinetic and Equilibrium Parameters of DNA–Ga Complexes

complex	*k*_ass_ (M^–1^ s^–1^)	*k*_diss_ (s^–1^)	*K*_D_ (μM)
**1**	29800 ± 10500	0.022 ± 0.015	0.76 ± 0.54
**2**	22400 ± 1150	0.081 ± 0.053	3.63 ± 1.88
**3**	34700 ± 3600	0.077 ± 0.039	2.24 ± 1.36
**4**	9600 ± 3500	0.032 ± 0.011	3.34 ± 1.67
**5**	27400 ± 5900	0.095 ± 0.006	3.27 ± 0.78

#### Binding to Serum Albumin

**Figure 10:**
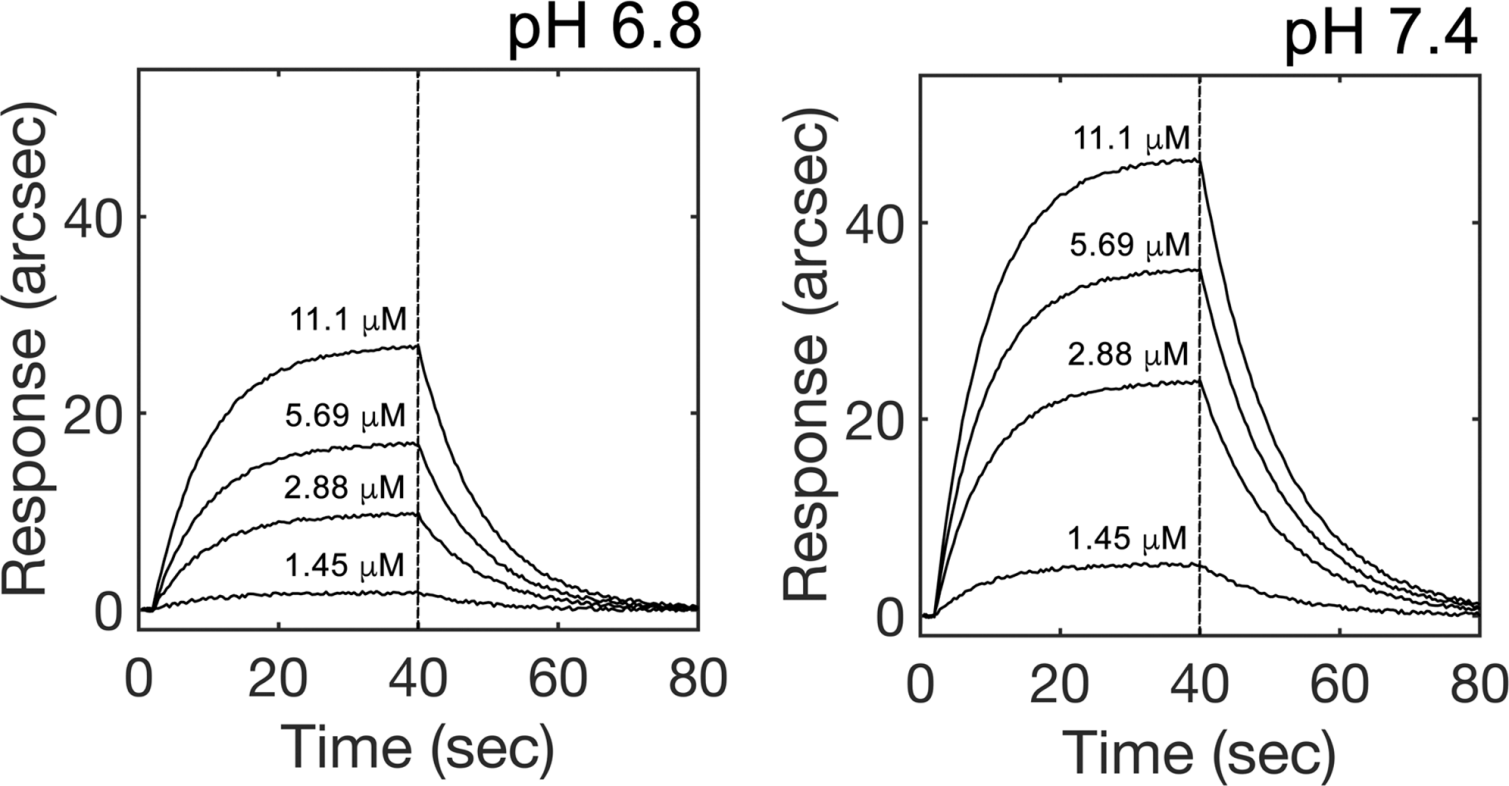
Representative sensor traces showing the changes in association kinetics of **4** to surface-blocked BSA at pH 6.8 (upper panel) and pH 7.4 (lower panel).

Serum albumins are among the most abundant proteins in the circulatory system, where they serve as carriers for both endogenous and exogenous molecules, among these drugs and other small ligands.^43^ Here, we evaluated and characterized the binding ability of **1**–**5** toward bovine serum albumin by combining different approaches. All complexes bound BSA and quenched its intrinsic fluorescence in a concentration-dependent manner (Figure S41) to the extent that was likely a function of the depth of insertion into the cleft formed between domains I and III (the most probable binding site on BSA, as calculated by molecular modeling) (Figure S40) and of the consequent proximity to Trp-213. Next, the kinetic and equilibrium parameters of these complexes were calculated using a biosensor-based binding assay (Figure 10). We reported the interaction to be reversible (*K*_D_ in the micromolar range) and pH-dependent, with the affinity for the molecules of interest decreasing upon BSA conformational transition due to H^+^ binding (Table S28).^44^ Such a modulation by H^+^ ions mostly affected the kinetic stability of the BSA–ligand complex (approx. 4-fold increase in *k*_diss_ and consequently in *K*_D_) and was consistent with the favorable transport of the candidate drug in the blood (pH = 7.35–7.4, high-affinity state of HSA) and its release at the tumor site (pH 6.8–7.0, low-affinity state of HSA).

## Conclusions

In this work, we reported the synthesis and the structural/functional characterization of a group of novel gallium anticancer coordination complexes. All of these compounds favorably interacted with proteins involved in drug transport and passed across cell membranes, with internalization rates in line with their degree of lipophilicity. Regarding their biological activity, we generally observed cell type-dependent cytotoxicity, which was positively influenced by the presence of a phenyl ring in the acyl fragment. These complexes displayed a fair selectivity *in vitro* both for epithelial breast cancer cells and Caco-2 colon carcinoma cells with respect to their nontumoral counterparts. Moreover, focusing on Caco-2 cells, gallium complexes not only showed significantly higher potency than cisplatin but preserved their cytotoxic potential also upon induction of resistance to cisplatin. Interestingly, the presence of the gallium(III) center was critical in establishing a different mechanism of cell death with respect to parent pyrazolonate ligands. In fact, irrespective of interacting with typical cellular targets (DNA) and stimulating several apoptotic signals such as p27 accumulation, PARP fragmentation, and activation of the caspase cascade, the treatment also caused a decrease in the level of pro-apoptotic BAX and had negligible effects on the cell cycle, consistently with a decrease in apoptosis. On the other hand, the treatment induced a statin-like inhibition of the mevalonate pathway and (most importantly) affected cell redox homeostasis, with decreased levels of GHS/GPX4 and NADP(H), increased LPO, ROS, and HNE, increased activity of CPR and CcO, and mitochondrial damage. All of these events highlighted a major role of ferroptosis in cell toxicity induced by the Ga complexes, as unequivocally confirmed by the protective effect of ferrostatin-1 on Caco-2 cell viability and on the activity/expression of ferroptosis-associated biomarkers. The gallium complexes successfully escaped cisplatin resistance by acting on different and multiple cellular targets, supporting their use also as second-line drugs in cancer treatment. Additionally, our data demonstrated the ability of normal cells to better recover from transient inhibition of the HMGR pathway and altered redox homeostasis, consequently evading cell death. In conclusion, our results emphasized the role of organometallic complexes containing nonradioactive gallium as anticancer drug candidates exploiting an atypical mechanism of action.

## Experimental Section

### Materials and Methods

All reagents and solvents were purchased from Aldrich and used as received without further purification. All reactions for the syntheses of proligands and the corresponding gallium complexes were carried out in air. The samples for microanalyses were dried in vacuo to constant weight (35 °C, ca. 0.1 Torr). Elemental analyses (C, H, N) were performed in-house with a Fisons Instruments 1108 CHNS-O elemental analyzer. IR spectra were recorded on a PerkinElmer Frontier FT-IR instrument. ^1^H and ^13^C NMR spectra were recorded on a 500 Bruker Ascend (500 MHz for ^1^H, 125 MHz for ^13^C) instrument operating at room temperature relative to TMS. Positive-ion electrospray mass spectra were obtained on a Series 1100 MSI detector HP spectrometer, using acetonitrile as a solvent for all complexes **1**–**5**. Solutions (3 mg/mL) for electrospray ionization mass spectrometry (ESI-MS) were prepared using reagent-grade methanol. Masses and intensities were compared to those calculated using the IsoPro Isotopic Abundance Simulator, version 2.1.28. Melting points were recorded on an STMP3 Stuart scientific instrument and a capillary apparatus. Samples for microanalysis were dried in vacuo to constant weight (20 °C, ca. 0.1 Torr) and analyzed on a Fisons Instruments 1108 CHNS-O elemental analyzer. The purity of all complexes was assessed by HPLC, generally obtaining ε 95% purity (Supporting Information). All cell lines (MCF-7, MCF-10A, Caco-2, CRL-1831, HCT-116, and HepG2) were obtained from ATCC (Rockville, MD). All media and reagents for cell cultures were purchased from EuroClone S.p.A. (Milan, Italy). Anti-PCNA Antibody PC10 was obtained from Santa Cruz Biotech (Heidelberg, Germany), Anti-Poly(ADP)-Ribose Polymerase and Anti-Caspase-3 antibodies were obtained from Calbiochem (Darmstadt, Germany), and p27/Hip1 antibody was acquired from Cell Signaling Technology (Danvers, Massachusetts). Binding experiments were performed on a resonant mirror optical biosensor (IAsys plus - Affinity Sensors Ltd.) equipped with dual-well carboxylate cuvettes (NeoSensors, Ltd).

### X-ray Crystallography

Bragg intensities of **1** and **2** were collected at 140 K using Cu Kα radiation. Suitable crystals of **1** and **2** were selected and mounted on an XtaLAB Synergy R, a DW system, and a HyPix-Arc 150 diffractometer. The datasets were reduced and corrected for absorption with the help of a set of faces enclosing the crystals as snugly as possible with the latest available version of *CrysAlis*^*Pro*^.^37^

The solution and refinement of the structures were performed by the latest available version of *ShelXT*(38) and *ShelXL*(39) using *Olex*2^40^ as the graphical interface. All non-hydrogen atoms were refined anisotropically using full-matrix least-squares based on |*F*|^2^. The hydrogen atoms were placed at calculated positions employing the “riding” model, where each H-atom was assigned a fixed isotropic displacement parameter with a value equal to 1.2 *U*eq of its parent C-atom.

Crystallographic and refinement data for **1** and **2** are summarized in Tables S4 and S16, respectively. The CCDC numbers 2190069 and 2190070 contain the crystallographic data for compounds **1** and **2**. These data can be obtained free of charge via www.ccdc.cam.ac.uk/data_request/cif.

#### Cell Viability Assay

Cells were grown in a 5% CO_2_ atmosphere at 37 °C in dedicated media in 100 mm tissue culture dishes. Specifically, Caco-2, Caco-CR (cisplatin resistance in Caco-2 cells was induced by chronic exposure to 15 μM cisplatin over a 4-week period), MCF-7, and HepG2 cells were cultured in MEM supplemented with 10% FBS, 1% sodium pyruvate, antibiotic, and antimycotic. HCT-116 cells were cultured in RPMI supplemented with 10% FBS; MCF-10A cells were cultured in DMEM/F12 Ham’s mixture supplemented with 5% equine serum, 20 ng/mL EGF, 10 μg/mL insulin, 0.5 mg/mL hydrocortisone, antibiotics, and antimycotics. CRL-1831 cells were cultured in DMEM/F12 supplemented with 10 mM HEPES, 20% FBS, 5 μg/mL insulin, 100 ng/mL hydrocortisol, and 1% penicillin/streptomycin. The effect of gallium complexes on cell viability was determined using the 3-(4,5-dimethylthiazol-2-yl)-2,5-diphenyltetrazolium bromide (MTT) assay.^45^ Stock solutions of **1**–**5** were prepared in DMSO and diluted in the proper media to give a final compound concentration range of 0–50 μM (the in-assay concentration of DMSO was always <0.5%). Cisplatin was used as a positive control. Cells were exposed to these treatments for 24 and 48 h (longer-term treatment did not significantly increase the cytotoxicity of the complexes). Next, MTT was added to the culture medium at a final concentration of 0.5 mg/mL and incubated for 4 h at 37 °C. The medium was removed and the purple formazan crystals (exclusively produced by the mitochondrial dehydrogenase activity of viable cells) were dissolved in 100 μL of DMSO. The optical density of the resulting solutions, which increases with the number of living cells, was measured at 550 nm after 10 min on a microplate reader. The percentage of viable cells was calculated with reference to DMSO-treated cells. At least five independent cultures were used for each time point.

#### Computational Details

The electronic structure and geometries of compounds **1**–**5** were investigated using density functional theory at the B3LYP level^46^ with the 6-31G* basis set. Additionally, the HQ*_n_* precursor ligands and the Q*_n_*^–^ ligands were optimized at the B3LYP/6-311G* level of theory. Frequency calculations were carried out at the same level of theory to identify all of the stationary points as transition states (one imaginary frequency) or as minima (zero imaginary frequencies) and to provide the thermal correction to free energies at 298.15 K and 1 atm. Molecular geometries were optimized without symmetry restrictions. DFT calculations were performed using the Gaussian 09 suite of programs.^47^ The coordinates of optimized compounds are reported in Table S3.

#### Fluorescence Anisotropy Measurements

The kinetics of transport across cell membranes were explored by monitoring the change in membrane fluidity of Caco-2 cells upon internalization of complexes **1**–**5**.^48^ Anisotropy measurements were carried out using a membrane-anchoring TMA–DPH fluorescent probe (λ_exc_ = 340 nm; λ_em_ = 460 nm) at 37 °C on an RF-5301PC Shimadzu spectrofluorometer. In detail, 1.5 × 10^5^/mL Caco-2 cells were preincubated with 1 μM TMA–DPH and individually added to 10 μM **1**–**5** and kept at 37 °C. Fluorescence anisotropy (r) was calculated at 10 min intervals for 200 min using the following model.
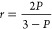
Fluorescence polarization (P) was derived using the equation
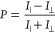
with *I*_|_ and *I*_⊥_ being the fluorescence intensities parallel and perpendicular to the excitation beam, respectively. The kinetic rate constants characterizing the main steps of the permeation event (namely, *k*_in_ and *k*_out_) were derived according to a general monoexponential model.
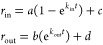
where *r*_in_ and *r*_out_ are the fluorescence anisotropy intervals corresponding to drug entry and exit phases from the membrane, respectively.

### Binding to DNA

#### Biosensor Binding Assay

The DNA binding ability of complexes **1**–**5** was tested according to a biosensor-based assay.^49^ The carboxylate surface of the biosensor cuvette was rinsed and equilibrated with PBS buffer (10 mM Na_2_HPO_4_, 2.7 mM KCl, 138 mM NaCl, pH = 7.4) and activated with an equimolar mixture of EDC and NHS.^50^ Streptavidin was dissolved in 10 mM CH_3_COONa buffer (pH 5) and then anchored to the carboxylic surface. Unoccupied carboxylic sites on the sensor surface were blocked by the injection of 1 M ethanolamine, pH 8.5. Finally, 5′-biotinylated dsDNA (3′-CCACCCACTACCCTGGTTGGATGCTAATGT-5′) was coupled to the streptavidin-coated surface. Complexes **1**–**5** were independently added to the DNA-coated surface at different concentrations in the range of 1–10 μM, each time following binding kinetics up to equilibrium. Dissociation steps were performed by a single 1 min wash with fresh PBS buffer, whereas free DNA surface regeneration was achieved by serial PBS washes, each time assessing the recovery of the free DNA baseline prior to any further addition. The biosensor chamber was thermostatted at 37 °C throughout. Raw data were globally fitted to both mono- and biexponential models and the validity of each model to fit time courses was assessed by a standard F-test procedure.

#### DAPI Displacement Assay

4′,6-Diamidino-2-phenylindole (DAPI) is a DNA minor groove-targeting molecule whose fluorescence intensity is enhanced upon binding to DNA.^51^ To evaluate the ability of **1–5** to bind to the DNA minor groove, the DAPI displacement assay was performed by monitoring the changes in the emission spectra (400–650 nm) of solutions containing different concentrations of the compounds of interest in the range of 0**–**200 μM, DNA (20 μM), and DAPI (15 μM) in phosphate buffer (10 mM, pH 7.4) at room temperature after excitation at 338 nm on a Gemini XPS microplate reader.

#### Methyl Green Displacement Assay

Methyl Green is a major groove-targeting molecule whose evanescent native UV–vis absorbance is retained in complexes with DNA.^52^ The competition of **1–5** with Methyl Green was explored to establish the ability of the Ga complexes to bind to the DNA major groove. Methyl Green (500 μM) was dissolved in 50 mM Tris-HCl buffer, pH 7.5, containing 7.5 mM MgSO_4_ and incubated with DNA (200 μM) for 24 h at 37 °C. Next, **1–5** at 0–200 μM were added to the preformed DNA–Methyl Green complex and the changes in absorbance at 630 nm were monitored with a Bio-Tek Visible plate reader.

#### Cytochrome *C* Reductase Assay

The residual activity of the NADPH-cytochrome p450 reductase in cell lysates was evaluated using horse heart cytochrome *c* as an electron acceptor substrate. Reaction mixtures consisted of 25 mM Tris-HCl, pH 8, 10 μg of protein lysate, and saturating concentrations of cytochrome *c* (38 μM). To rule out possible interference in the assay due to cytochrome c oxidase, all tests were performed in the presence or in the absence of 10 mM KCN. The kinetics of cytochrome *c* reduction were monitored at 550 nm for 5 min. The reaction was initiated by the addition of 150 μM NADPH in a final volume of 1 mL. The slope of cytochrome c reduction curves (the initial velocity, *v*_0_) was a measure of functional NADPH-cytochrome p450 reductase levels. Kinetic assays were performed at 25 °C on a Cary 100 spectrophotometric device.

### Binding to Serum Albumin

#### Fluorescence Quenching

Bovine serum albumin (BSA) was used in our experiments owing to the high structural homology with human serum albumin.^53^ Quenching of BSA tryptophan fluorescence was used to study the interaction between serum albumin and **1**–**5**. Fluorescence spectra of 10 μM BSA were recorded in the presence and in the absence of **1–5** from 300 to 450 nm upon tryptophan excitation at 295 nm. Fluorometric titrations were performed by individual additions of Ga complexes in the range of 1–10 μM. All titrations were performed at 37 °C.

#### Biosensor Binding Assay

The binding kinetics of **1**–**5** to BSA were explored on an IAsys plus biosensor. The BSA sensing surface was prepared via EDC/NHS chemistry^50^ and then the complexes of interest were independently added at different concentrations in the range of 0–12 μM at 37 °C and replicated at different pH values (6.8 and 7.4). Raw data were globally fitted to both mono- and biexponential models and the validity of each model to fit time courses was assessed by a standard F-test procedure.

#### Proteasome Activities on Cell Lysates

The proteasome is a validated anticancer drug target and some proteasome inhibitors have been approved by the FDA for the treatment of different tumors.^54^ The effect of **1**–**5** on 20S and 26S proteasomes peptidase activities in cell lysates was evaluated with fluorogenic peptides, as previously described.^55^ Briefly, Suc-Leu-Leu-Val-Tyr-AMC was used for chymotrypsin-like activity (ChT-L), Z-Leu-Ser-Thr-Arg-AMC for trypsin-like activity (T-L), and Z-Leu-Leu-Glu-AMC for peptidylglutamyl peptide hydrolase activity (PGPH). For the analysis of 20S activity, the reaction mixtures consisted of 1 μg of total proteins from each treatment (control, DMSO, and **4**), the individual substrate, and 50 mm Tris-HCl, pH 8.0. Incubation was carried out at 37 °C and after 60 min, the fluorescence of the hydrolyzed AMC was recorded (AMC: λ_exc_ = 365 nm, λ_em_ = 449 nm) on a SpectraMax Gemini XPS microplate reader.

Analogously, 26S proteasome ChT-L activity was tested using 1 μg of total proteins from each treatment, Suc-Leu-Leu-Val-Tyr-AMC as a substrate, and a 50 mm Tris-HCl (pH 8.0) buffer containing 10 mm MgCl_2_, 1 mm DTT, and 2 mm ATP. Incubation was carried out at 37 °C for 60 min and then the fluorescence of hydrolyzed AMC was measured (AMC: λ_exc_ = 365 nm, λ_em_ = 449 nm) on a SpectraMax Gemini XPS microplate reader.

#### Western Blotting Analyses

Western blotting assays were performed to quantitate the levels of cellular markers of oxidative damage-triggered apoptosis, namely, poly(ADP-ribose) polymerase (PARP), p27, proliferating cell nuclear antigen (PCNA), caspase-3, 4-hydroxynonenal (4-HNE), and glutathione peroxidase 4 (GPX4), upon treatment of Caco-2 cells with 10 μM of **4**. Cell lysates (15 μg of proteins) were electrophoresed on 12% SDS-PAGE (10% for PARP) and electroblotted onto PVDF membranes Millipore (Milan, Italy). After incubation with primary antibodies, the immunoblot detections were carried out with the Enhanced ChemiLuminescence Western Blotting analysis system (Amersham-Pharmacia-Biotech). Each gel was loaded with molecular weight markers including proteins in the range of 6.5–205 kDa. Glyceraldehyde-3-phosphate dehydrogenase (GAPDH) was used as a control for equal protein loading. ImageJ software^56^ was used to quantitate the western blot results.

#### Caspase Activity Assay

The caspase-3 activity assay was performed by incubating cell lysates (5 μg of total proteins) using the Ac-Asp-Glu-Val-Asp-AMC substrate (Sigma-Aldrich S.r.L., Milan, Italy) in 50 mM Tris-HCl, 50 mM NaCl, 5 mM CaCl_2_, 1 mM EDTA, 0.1% CHAPS, and 5 mM β-mercaptoethanol, pH 7.5. Incubation was carried out at 37 °C for 60 min, and the hydrolysis product was detected (AMC: λ_exc_ = 365 nm, λ_em_ = 449 nm) on a SpectraMax Gemini XPS microplate reader.

### Binding to the 3-Hydroxy-3-methylglutaryl-coenzyme A Reductase

#### Biosensor Binding Assay

The binding of complex **4** to the 3-hydroxy-3-methylglutaryl-coenzyme A reductase (HMGR) was evaluated according to a standard biosensor assay. Briefly, upon activation of carboxylate groups with an equimolar solution of EDC and NHS, HMGR was covalently anchored to the surface. The preservation of the native-like conformation/functionality upon immobilization was confirmed by checking the ability of HMGR to recognize its physiological substrates, namely, HMG-CoA and NADPH. Next, surface-blocked HMGR was tested for binding to **4** at different concentrations in the range of 1–50 nM. Finally, the binding for the Ga complex on HMGR was mapped by competitive binding with HMG-CoA and NADPH. The biosensor chamber was thermostatted at 37 °C throughout. Raw data were analyzed with mono- and biexponential models, the validity of each model to fit time courses being assessed by the F-test procedure.

#### Inhibition of HMGR Activity

The HMGR inhibiting ability of **4** was explored according to a previously described chromatographic protocol.^57^ Briefly, the microsomal reductase purified from the human liver (10 μg of proteins) was preincubated for 20 min with increasing levels of the complex in the range of 0–1 μM. The reaction was started by the addition of 1.55 μM HMG-CoA and 2.68 μM NADPH and additionally stored for 60 min at 37 °C. The resulting mixture was separated with a Phenomenex Luna C18 reverse phase (RP)-HPLC column at 26 ± 0.1 °C, following both the decrease in HMG-CoA/NADPH consumption and mevalonate/NADP^+^ production rates. Residual activities were calculated from raw data using a standard model for reversible competitive inhibition.^41^

#### Effect on Cytoplasmic Cholesterol Levels

Changes in cytoplasmic levels of cholesterol in Caco-2 cells were determined to assess the effective cholesterol-lowering capacity of **4** compared to an established HMGR inhibitor (Simvastatin). After 4 h exposure to 10 μM of both compounds at 37 °C, cells were trypsinized, washed with PBS, and pelleted by centrifugation at 8000*g* for 5 min. Cytoplasmic cholesterol levels were quantitated using the AmplexRed Cholesterol Assay kit.^48^ Briefly, the pellets were resuspended in the reaction buffer and lysed with a 29G syringe. The working solution, containing the Amplex Red reagent (300 mM), horseradish peroxidase (2 U/mL), cholesterol oxidase (2 U/mL), and cholesterol esterase (0.2 U/mL) in 1× reaction buffer was freshly prepared before each experiment. The cholesterol reference standard (5.17 mM) was used to generate the calibration curves. Working solution (50 μL), 40 μL of 1× reaction buffer, and 40 μL of cell lysates were placed in a 96-well plate and incubated at 37 °C for 30 min. After 4 h, fluorescence measurements were recorded (λ_exc_ = 540 nm, λ_em_ = 590 nm) using a SpectraMax Gemini XPS microplate reader (Molecular Device, Milan – Italy).

#### Mitochondrial Transmembrane Potential

The mitochondrial transmembrane potential (ΔΨm) was evaluated by 5,5′,6,6′-tetrachloro-1,1′,3,3′-tetraehylbenzimidazolylcarbocyanineiodide (JC-1) staining. Caco-2 cell lines (4 × 10^4^/well) were seeded into 6-well plates and treated with 10 μM complex **4** or the vehicle for 48 h and then incubated with 10 μg/ml of JC-1. Carbonyl cyanide chlorophenylhydrazone protonophore (CCCP, 50 μM, Sigma-Aldrich), a mitochondrial uncoupler that collapses ΔΨm, was used as a positive control. Samples were analyzed using the FACScan cytofluorimeter with CellQuest software.

#### ROS/RNS Determination

Cellular levels of reactive oxygen species (ROS) and reactive nitrogen species (RNS) were measured using the DCF ROS/RNS Assay Kit (Abcam) according to the manufacturer’s guidelines. Briefly, upon 48 h treatment with complex **4**, cells were lysed in PBS and promptly tested. The dichlorodihydrofluorescin DiOxyQ (DCFH-DiOxyQ) fluorescent probe was primed with a quench removal reagent and stabilized in the highly reactive DCFH form. The probe was then added to the cell homogenate in a 96-well plate for 45 min. The fluorescence of the probe was measured using a SpectraMax Gemini XPS microplate reader (Molecular Device – Milan, Italy) at an excitation wavelength of 480 nm and an emission of 530 nm.

#### Lipid Peroxides Analysis by Flow Cytometry

Lipid peroxides were quantitated using the C11-BODIPY (581/591) sensor in compliance with the manufacturer’s instructions. Briefly, colon cells were treated with compound **4** in the presence and in the absence of ferrostatin for 48 h. After incubation, cells were washed with PBS, trypsinized, and pelleted by centrifugation. The pellet was stained with C11-BODIPY 581/591 (2 μM) for 30 min at 37 °C. The oxidation of the polyunsaturated butadienyl portion of the dye resulted in a fluorescence emission peak shift from 590 to 510 nm, detected using a BD Accuri C6 Plus flow cytometer (BD Biosciences, San Jose, CA). Fluorescence intensity was quantitated using BD Accuri C6 Plus software.

#### Determination of Total GSH

GSH levels were determined according to a standard colorimetric assay.^58^ Briefly, cells treated with complex **4** were lysed in 150 μL of lysis buffer containing 5% sulfosalicylic acid. Lysates were centrifuged and 50 μL of the supernatant was mixed with 150 μL of potassium phosphate buffer, pH 7.0, containing 5 mM EDTA, 1.5 mg/mL DTNB (5,5′-dithiobis-(2-nitrobenzoic acid)), and (6 U/mL glutathione reductase). NADPH (50 μL, 0.16 mg/mL) dissolved in potassium phosphate buffer was added to this mixture, and the absorbance was recorded at 412 nm using a SpectraMax ABS Plus UV–vis microplate reader (Molecular Devices – Milan, Italy).

#### Quantification of NADP(H)

Caco-22 cells were treated for 48 h with complex **4** 10 μM and then lysed by performing two freeze/thaw cycles in dry ice. The resulting samples were centrifuged at 13,000*g* for 5, and the supernatants were filtered on a 10 kDa spin column to remove enzymes. NADP and NADPH levels were determined using a colorimetric NADP/NADPH ratio assay kit (Abcam) according to the manufacturer’s guidelines. Absorbances at 450 nm were recorded on a Bio-Tek Visible plate reader.

#### Statistical Analysis

Results of experiments presented in this study are expressed as mean values with their standard deviations obtained from at least three independent experiments. Statistical analysis was performed with one-way ANOVA, followed by the Bonferroni test using MATLAB R2021b. *p*-values < 0.05 (*) and < 0.01 (**) were considered significant.

### Syntheses of the Proligands

#### HQ_1_

It was prepared by the general procedure previously reported. It is soluble in alcohols, DMSO, acetone, acetonitrile, and chlorinated solvents. Anal. Calcd for C_17_H_14_N_2_O_2_: C, 73.22; H, 5.27; N, 9.98. Found: C, 73.16; H, 5.16; N, 10.12. Mp 94–96 °C. IR (cm^–1^): 3057w, 2700br (ν_O–H_), 1599s, 1570s, 1560s, 1554s, 1536sh (ν_C=O_, ν_C=C_) 602m, 533m, 507m, 412w, 400w, 392w, 361w, 328w, 296w, 281w. ^1^H NMR (C*D*Cl_3_, 500 MHz): δ, 2.12 (3H, s, C*H*_3_), 7.33, 7.5, 7.9 (10H, m, C_6_H_5_), 10.5 (1H, s br, OH). ^13^C{^1^H} NMR (C*D*Cl_3_): δ, 15.8 (CH_3_), 120.8, 126.7, 128.3, 129.1, 131.9, 137.3, 137.6 (s, C_arom_ of C_6_H_5_), 103.6 (s, C3), 148.0 (s, C4), 162.5 (s, C5), 192.0 (s, CO).

#### HQ_2_

It was prepared by the general procedure previously reported. It is soluble in alcohols, DMSO, acetone, acetonitrile, and chlorinated solvents. Anal. Calcd for C_15_H_12_N_2_O_3_: C, 67.16; H, 4.51; N, 10.44. Found: C, 67.19; H, 4.57; N, 10.40. Mp 103–105 °C. IR (cm^–1^): 2700br (ν_O–H_), 1625m, 1585s, 1531sm (ν_C=O_, ν_C=C_), 690s, 604s, 585s, 509s, 391m, 355s, 282s. ^1^H NMR (CDCl_3_, 500 MHz): δ, 2.65 (3H, s, CH_3_), 6.68 (1H, t, C_4_H_3_O), 7.31 (1H, t, C_4_H_3_O), 7.45 (3H, m, C_6_H_5_), 7.69 (1H, d, C_4_H_3_O), 7.92 (2H, d, C_6_H_5_).

#### HQ_3_

It was prepared by the general procedure previously reported. It is soluble in alcohols, DMSO, acetone, acetonitrile, and chlorinated solvents. Anal. Calcd for C_15_H_12_N_2_O_2_S: C, 63.38; H, 4.23; N, 9.86; S, 11.27. Found: C, 63.89; H, 4.35; N, 9.76; S, 11.01. Mp 152–153 °C. IR (cm^–1^): 3200–2400 (ν_O–H_)1634s, 1596s, 1558s (ν_C=O_, ν_C=C_), 429s, 383s, 369s, 315s, 295s, 277s. ^1^H NMR (CDCl3, 500 MHz): δ, 2.48 (3H, s, CH_3_), 7.17 (1H, t, C_4_H_3_S), 7.29 (1H, t, C_4_H_3_S), 7.47 (2H, t, Ph), 7.72 (1H, t, Ph), 7.74 (1H, d, C_4_H_3_S), 7.84 (2H, d, Ph), 12.1 (1H, s br, OH).

#### HQ_4_

It was prepared by the general procedure previously reported. It is soluble in alcohols, DMSO, acetone, acetonitrile, and chlorinated solvents. Anal. Calcd for C_18_H_16_N_2_O_3_: C, 70.12; H, 5.23; N, 9.09. Found: C, 70.01; H, 5.24; N, 9.12. Mp 163–165 °C. IR (cm^–1^): 2600 sbr (ν_O–H_), 1704s, 1594s, 1497s (ν_C=O_, ν_C=C_), 1351s, 1168s, 1021s, 594s, 500s. ^1^H NMR (CDCl3, 500 MHz): δ, 2.22 (1H, s, CH_3_), 3.92 (1H, s, OMe), 7.03 (2H, d, C_6_H_4_-*p*-OMe), 7.32 (1H, t, Ph), 7.49 (2H, t, Ph), 7.68 (2H, d, C_6_H_4_-*p*-OMe), 7.92 (2H, d, Ph).

#### HQ_5_

It was prepared by the general procedure previously reported. It is soluble in alcohols, DMSO, acetone, acetonitrile, and chlorinated solvents. Anal. Calcd for C_21_H_22_N_2_O_2_: C, 75.42; H, 6.63; N, 8.38. Found: Found: C, 75.38; H, 6.71; N, 8.49. Mp 111–112 °C. IR (cm^–1^): 2500br (ν_O···H_), 2867w (ν_C···H_ aliphatic), 1606s, 1539s (ν_C=O_, ν_C=C_), 1461m, 1362m, 948s, 855s, 757s, 689s, 639w, 552w, 279s, 245s. ^1^H NMR (CDCl3, 500 MHz): δ, 1.36 (3H, s, CH_3_), 1.39 (12H, s, CH(CH_3_)_3_), 7.31 (1H, t, Ph), 7.49 (2H, m, Ph), 7.55 (2H, d, C_8_H_13_), 7.63 (2H, d, C_8_H_13_), 7.9 (2H, d, Ph), 11.23 (1H, s br, OH).

### Syntheses of Gallium Complexes

#### Ga(Q_1_)_3_ (**1**)

Proligand HQ_1_ (167 mg, 0.60 mmol) was dissolved in methanol (10 mL) and NaOCH_3_ (32 mg, 0.60 mmol) was added. The mixture was stirred for 1 h under reflux and then Ga(NO_3_)_3_ (51 mg, 0.20 mmol) was added. A pale pink precipitate immediately formed. The mixture was stirred under reflux for 24 h. Then, the suspension was filtered off, and the precipitate was washed with Et_2_O and dried to constant weight under reduced pressure (180 mg, yield 75%) and recrystallized from chloroform/*n*-hexane. It is soluble in acetone, acetonitrile, and chlorinated solvents and sparingly soluble in DMSO, DMF, and alcohols. Mp: 172–174 °C. Anal. Calcd for C_51_H_39_GaN_6_O_6_: C, 67.94; H, 4.36; N, 9.32. Found: C, 67.73; H, 4.27; N, 9.29. HPLC purity: 99.46%. IR (cm^–1^): 3062w (ν_C–Haromatic_), 1601m, 1563vs (ν_C=C_), 1476s (ν_C=N_), 1383s, 1164m, 1058m, 1026w, 956m, 842m, 765m, 690s, 621m (ν_Ga-O_), 508m, 470w, 356m, 254s. ^1^H NMR (C*D*Cl_3_, 298 K): δ, 1.70 (3H, s, CH_3_), 1.80 (3H, s, CH_3_), 1.85 (3H, s, CH_3_), 1.92 (3H, s, CH_3_), 7.36 (32H, m, Ph), 7.90 (8H, m, H7, H7′). ^13^C{^1^H} NMR (C*D*Cl_3_, 298 K): δ, 191.1, 190.8 (q, C10), 165.2, 165.1, 165.0, 164.9 (q, C5), 149.5, 149.4, 149.3 (q, C3), 138.3 (d, C11), 138.2, 138.0 (q, C6), 131.5, 131.3, 131.2 (dd, C14), 128.7 (q, C8, C8′), 128.4 (q, C12, C12′), 128.1 (q, C13, C13′), 125.7 (t, C9), 120.9, 120.8, 120.5 (q, C7, C7′), 105.4 (t, C4), 16.3, 16.2 (q, CH_3_). ESI-MS (+) CH_3_CN (*m*/*z*, relative intensity %): 923 [100] [Ga(L_1_)_3_ + Na^+^], 941[78] [Ga(L_1_)_3_ + K^+^], 901 [23] [Ga(L_1_)_3_ + H^+^].

#### Ga(Q_2_)_3_ (**2**)

Complex **2** (104 mg, yield 60%) has been synthesized similarly to **1** using HQ^2^ (161 mg, 0.60 mmol), NaOCH_3_ (32 mg, 0.60 mmol), and Ga(NO_3_)_3_ (51 mg, 0.20 mmol). It is soluble in acetone, acetonitrile, and chlorinated solvents and sparingly soluble in DMSO, DMF, and alcohols. Mp: 271.4, 272.8 °C. Anal. Calcd for C_45_H_33_GaN_6_O_9_: C, 62.02; H, 3.82; N, 9.64. Found: C, 62.00; H, 3.74; N, 9.53. HPLC purity: 97.35%. IR (cm^–1^): 3174w (ν_C–Haromatic_), 1619w, 1575m (ν_C=C_), 1548m, 1528m, 1472s (ν_C=N_), 1445s, 1375m, 1234w, 1154w, 1014m, 833m, 756s, 627s (ν_Ga-O_), 510m, 386w, 253s. ^1^H NMR (C*D*Cl_3_, 298 K): δ, 2.41 (9H, t, CH_3_), 2.49 (3H, s, CH_3_), 6.58 (4H, d, H13), 7.23 (16H, m, H8, H8′, H9, H12), 7.67 (4H, s, H14), 7.88 (8H, dd, H7, H7′). ^13^C{^1^H} NMR (C*D*Cl_3_, 298 K): δ, 174.5 (s, C10), 166.0 (s, C5), 151.3 (s, C11), 148.9 (s, C3), 146.1 (s, C14), 138.1 (s, C6), 128.7 (s, C8, C8′), 125.6 (s, C9), 121.1, 121.0, 120.5 (q, C7, C7′), 119.6 (t, C12), 112.7, 112.5 (d, C13), 103.4 (t, C4), 17.5, 17.4 (d, CH_3_). ESI-MS (+) CH_3_CN (*m*/*z*, relative intensity %): 909 [100] [Ga(L_1_)_3_ + K^+^], 893 [81] [Ga(L_1_)_3_ + Na^+^], 871 [19] [Ga(L_1_)_3_ + H^+^].

#### Ga(Q_3_)_3_ (**3**)

Complex **3** (104 mg, yield 60%) has been synthesized similarly to **1** using HQ^3^ (183 mg, 0.60 mmol), NaOCH_3_ (32 mg, 0.60 mmol), and Ga(NO_3_)_3_ (51 mg, 0.20 mmol). It is soluble in acetone, acetonitrile, and chlorinated solvents and sparingly soluble in DMSO, DMF, and alcohols. Mp: 175.8, 176.4 °C. Anal. Calcd for C_45_H_33_GaN_6_O_6_S_3_: C, 58.77; H, 3.62; N, 9.14. Found: C, 58.54; H, 3.57; N, 8.99. HPLC purity: 95.21%. IR (cm^–1^): 3084w (ν_C–Haromatic_), 1593m (ν_C=C_), 1562s, 1528m, 1480s (ν_C=N_), 1459s, 1380m, 1340s, 1228w, 1148w, 1057m, 909m, 822s, 723s, 688s, 623s (ν_Ga-O_), 507m, 361w, 246s. ^1^H NMR (C*D*Cl_3_, 298 K): δ, 2.16 (9H, t, CH_3_), 2.28 (3H, s, CH_3_), 7.14 (8H, m, H9 and H13), 7.26 (8H, m, H8 and H8′), 7.59 (8H, m, H12 and H14), 7.91 (8H, m, H7 and H7′). ^13^C{^1^H} NMR (C*D*Cl_3_, 298 K): δ, 181.7 (s, C10), 165.3 (q, C5), 148.4 (t, C3), 140.7 (t, C11), 138.0 (s, C6), 132.7 (s, C14), 132.3 (s, C12), 128.7 (s, C8, C8′), 127.1 (s, C13), 125.8 (s, C9) 121.3, 121.0, 120.5 (t, C7, C7′), 105.4 (t, C4), 16.9, 16.8, 16.7 (t, CH_3_). ESI-MS (+) CH_3_CN (*m*/*z*, relative intensity %): 941 [100] [Ga(L_1_)_3_ + Na^+^], 957 [17] [Ga(L_1_)_3_ + K^+^], 919 [10] [Ga(L_1_)_3_ + H^+^].

#### Ga(Q_4_)_3_ (**4**)

Complex **4** (125 mg, yield 63%) has been synthesized similarly to **1** using HQ^4^ (185 mg, 0.60 mmol), NaOCH_3_ (32 mg, 0.60 mmol), and Ga(NO_3_)_3_ (51 mg, 0.20 mmol). It is soluble in acetone, acetonitrile, chlorinated solvents, DMSO, and DMF and sparingly soluble in alcohols. Mp: 171.3, 174.2 °C. Anal. Calcd for C_54_H_45_GaN_6_O_9_: C, 65.40; H, 4.57; N, 8.47. Found: C, 65.28; H, 4.39; N, 8.46. HPLC purity: 99.58%. IR (cm^–1^): 2970w (ν_C–Haromatic_), 1601m (ν_C=C_), 1558s, 1532m, 1472s (ν_C=N_), 1456s, 1382m, 1306m, 1251s, 1160s, 1057m, 955m, 849m, 785s, 762s, 667s (ν_Ga-O_), 498m, 326w, 249s. ^1^H NMR (C*D*Cl_3_, 298 K): δ, 1.80 (3H, s, CH_3_), 1.94 (6H, d, CH_3_), 2.05 (3H, s, CH_3_), 3.87 (12H, s, OCH_3_), 6.93 (8H, t, H13 and H13′), 7.13 (4H, m, H9), 7.27 (12H, m, H8 and H8′), 7.51 (8H, m, H12 and H12′), 7.90 (8H, m, H7 and H7′). ^13^C{^1^H} NMR (C*D*Cl_3_, 298 K): δ, 189.9, 190.3 (d, C10), 165.2, 165.0 (d, C5), 162.7, 162.5 (d, C14), 149.0 (s, C3), 138.1 (s, C6), 131.2, 130.1 (d, C12, C12′), 130.4 (s, C11), 128.7, 128.6 (s, C8, C8′), 125.6 (s, C9), 120.8, 120.7, 120.5, 120.4 (q, C7, C7′), 113.5, 113.4 (d, C13, C13′), 104.9 (t, C4), 55.4 (s, OCH_3_), 16.5 (s, CH_3_). ESI-MS (+) CH_3_CN (*m*/*z*, relative intensity %): 1013 [100] [Ga(L_1_)_3_ + Na^+^], 1029 [33] [Ga(L_1_)_3_ + K^+^], 991 [23] [Ga(L_1_)_3_ + H^+^].

#### Ga(Q_5_)_3_ (**5**)

Complex **5** (189 mg, yield 88%) has been synthesized similarly to **1** using HQ^5^ (200 mg, 0.60 mmol), NaOCH_3_ (32 mg, 0.60 mmol), and Ga(NO_3_)_3_ (51 mg, 0.20 mmol). It is soluble in acetone, acetonitrile, and chlorinated solvents and sparingly soluble in DMSO, DMF, and alcohols. Mp: 189.3, 190.7 °C. Anal. Calcd for C_63_H_63_GaN_6_O_6_: C, 70.72; H, 5.94; N, 7.85. Found: C, 70.66; H, 5.82; N, 7.73. HPLC purity: 96.01%. IR (cm^–1^): 2970w (ν_C–Halifatic_), 1595m (ν_C=C_), 1575s, 1537m, 1477s (ν_C=N_), 1462s, 1382m, 1268w, 1164m, 1056m, 959m, 850m, 787m, 690s, 629s (ν_Ga-O_), 506m, 326w, 264s. ^1^H NMR (C*D*Cl_3_, 298 K): δ, 1.30 (18H, t, −C(CH_3_)_3_), 1.73 (3H, s, CH_3_), 1.89 (6H, d, CH_3_), 1.98 (3H, s, CH_3_), 7.19–7.50 (28H, m, H13, H13′, H9, H8, H8′, H12, H12′), 7.90 (8H, m, H7 and H7′). ^13^C{^1^H} NMR (C*D*Cl_3_, 298 K): δ, 191.2, 191.2, 190.8 (q, C10), 165.3, 165.2, 165.0, 164.9 (q, C5), 155.2, 155.0, 154.9 (q, C14), 149.4, 149.4, 149.2 (q, C3), 138.1, 138.0 (q, C6), 135.5, 135.2 (q, C11), 128.7, 128.6 (q, C8, C8′), 128.2, 128.1 (d, C12, C12′), 125.6 (q, C9), 125.1, 125.0 (q, C13, C13′), 120.8, 120.5 (d, C7, C7′), 105.3, 105.1, 105.0 (q C4), 35.0 (s, −C(CH_3_)_3_), 31.2 (s, −C(CH_3_)_3_), 16.4 (t, CH_3_). ESI-MS (+) CH_3_CN (*m*/*z*, relative intensity %): 1091 [100] [Ga(L_1_)_3_ + Na^+^], 1071 [30] [Ga(L_1_)_3_ + H^+^].

## Supporting Information Available

Relevant NMR spectra, stability studies, DFT data, crystal and experimental data, molecular docking, binding to BSA, binding to DNA, cell membrane permeability, binding to HMGR, effect on the cell cycle, effect on the cytochrome p450 oxidase, effect on 20S and 26S cellular proteasomes, effect of ferrostatin-1 on complex **4** cytotoxicity, effect of ferrostatin-1 on cellular redox homeostasis, and purity evaluation (PDF)
Crystallographic data 1 (CIF)
Crystallographic data 2 (CIF)
Molecular formula strings (CSV)

## Supplementary Material

jm2c01374_si_001.pdf

jm2c01374_si_002.cif

jm2c01374_si_003.cif

## Notes

The authors will release the atomic coordinates upon article publication.

## Abbreviations Used

BAX: BCL2 associated X protein
CCCP: carbonyl cyanide chlorophenylhydrazone protonophore
CcO: citochrome C oxidase
CHT-L: chymotrypsin-like
CPR: NADPH-cytochrome P450 reductase
CR: cisplatin-resistant
CYP450: cytochromes P450
DAPI: 4′,6-diamidino-2-phenylindole
DCF: 2′,7′-dichlorofluorescein
DCFH-DA: diacetyldichlorofluorescein
DMEM: Dulbecco’s modified Eagle’s medium
DTNB: 5,5′-dithiobis-(2-nitrobenzoic acid)
EDC: N-ethyl-N′-(3-(dimethylamino)propyl)carbodiimide
ESI: electrospray ionization
FBS: fetal bovine serum
FMN: flavin mononucleotide
GPX4: glutathione peroxidase
GSH: glutathione
HMG-CoA: 3-hydroxy-3-methylglutaryl coenzyme A
HMGR: 3-hydroxy-3-methylglutaryl coenzyme A reductase
HNE: 4-hydroxynonenal
JC-1: 5,5′,6,6′-tetrachloro-1,1′,3,3′-tetraehylbenzimidazolylcarbocyanineiodide
LPO: lipid peroxidation
MTT: 3-(4,5-dimethylthiazol-2-yl)-2,5-diphenyltetrazolium bromide
NHS: *N*-hydroxysuccinimide
p27: cyclin-dependent kinase inhibitor
PARP: poly (ADP-ribose) polymerase
PCNA: proliferating cell nuclear antigen
PGPH: peptidylglutamyl peptide hydrolase
RNS: reactive nitrogen species
SPR: surface plasma resonance
TMA–DPH: trimethylammonium diphenylhexatriene
