# Supplementary material for: Multitarget-Directed Gallium(III) Tris(acyl-pyrazolonate) Complexes Induce Ferroptosis in Cancer Cells via Dysregulation of Cell Redox Homeostasis and Inhibition of the Mevalonate Pathway
Source: J Med Chem. 2023 Feb 21;66(5):3212–25. doi: 10.1021/acs.jmedchem.2c01374 (PMC10009752; doi:10.1021/acs.jmedchem.2c01374)
Supplement: Supplementary file 1 — jm2c01374_si_001.pdf [file jm2c01374_si_001.pdf]

Supporting Information

Multitarget-directed gallium(III) tris(acyl-pyrazolonate) complexes induce ferroptosis in cancer cells via dysregulation of cell redox homeostasis and inhibition of the mevalonate pathway

Daphne Romani,[a] Fabio Marchetti,[a] Corrado Di Nicola,[a] Massimiliano Cuccioloni,[b] Chunmei Gong,[b] Anna Maria Eleuteri,[b] Agustín Galindo,[c] Farzaneh Fadaei-Tirani,[d] Massimo Nabissi,[e] Riccardo Pettinari.[e]*

[a]School of Science and Technology, [b] School of Biosciences and Biotechnology,[e] School of Phamacy, University of Camerino, Via Madonna delle Carceri (ChIP), 62032 Camerino MC, Italy.

[c]Departamento de Química Inorgánica, Facultad de Química, Universidad de Sevilla, Aptdo 1203, 41071 Sevilla, Spain.

[d]Institut of Chemical Sciences and Engineering, Swiss Federal Institute of Technology Lausanne (EPFL), Lausanne CH-1015, Switzerland.

Corresponding Author

Riccardo Pettinari – School of Pharmacy, University of Camerino, Via Madonna delle Carceri (ChIP), 62032 Camerino MC, Italy.

Email: riccardo.pettinari@unicam.it

Contents of Supporting Information

Relevant NMR spectra *S6*

Stability Studies *S22*

DFT data *S23*

Crystal Data and Experimental *S54*

Molecular docking. *S82*

Binding to BSA *S82*

Binding to DNA *S83*

Cell membrane permeability *S85*

Binding to HMGR *S87*

Effect on cell cycle *S88*

Effect on cytochrome p450 oxidase *S89*

Effect on 20S and 26S cellular proteasomes *S89*

Effect of ferrostatin-1 on complex 4 cytotoxicity *S90*

Effect of ferrostatin-1 on cellular redox homeostasis *S91*

Purity evaluation *S92*

REFERENCES *S98*

List of Figures

Figure S1. *^1H NMR spectrum in $CDCl_3$ at 298 K of* ***Ga(Q1)$_3$*** S6
Figure S2. *Magnification of ^1H NMR spectrum in CDCl3 at 298 K of* ***Ga(Q1)$_3$*** S7
Figure S3. *Magnification of {^1H, ^1H}-COSY spectrum in $CDCl_3$ at 298 K of* ***Ga(Q1)$_3$*** S7
Figure S4. *^{13}C NMR spectrum in $CDCl_3$ at 298 K of* ***Ga(Q1)$_3$*** S8
Figure S5. *Magnification of ^{13}C NMR spectrum in $CDCl_3$ at 298 K of* ***Ga(Q1)$_3$*** S8
Figure S6. *Magnification of ^{13}C NMR spectrum in $CDCl_3$ at 298 K of* ***Ga(Q1)$_3$*** S9
Figure S7. *Magnification of {^1H, ^{13}C}-HSQC spectrum in $CDCl_3$ at 298 K of* ***Ga(Q1)$_3$*** S9
Figure S8. *{^1H, ^{15}N}-HMBC spectrum in $CDCl_3$ at 298 K of* ***Ga(Q1)$_3$*** S10
Figure S9. *^1H NMR spectrum in $CDCl_3$ at 298 K of* ***Ga(Q2)$_3$***. S10
Figure S10. *{^1H,^1H}-COSY spectrum in $CDCl_3$ at 298 K of* ***Ga(Q2)$_3$*** S11
Figure S11. *^{13}C NMR spectrum in $CDCl_3$ at 298 K of* ***Ga(Q2)$_3$*** S11
Figure S12. *{^1H,^{13}C}-HSQC spectrum in $CDCl_3$ at 298 K of* ***Ga(Q2)$_3$***. S12
Figure S13. *^1H NMR spectrum in $CDCl_3$ at 298 K of* ***Ga(Q3)$_3$*** S12
Figure S14. *{^1H,^1H}-COSY spectrum in $CDCl_3$ at 298 K of* ***Ga(Q3)$_3$*** S13
Figure S15. *^{13}C NMR spectrum in $CDCl_3$ at 298 K of* ***Ga(Q3)$_3$*** S13
Figure S16. *Magnification of ^{13}C NMR spectrum in $CDCl_3$ at 298 K of* ***Ga(Q3)$_3$***. S14
Figure S17. *{^1H,^{13}C}-HSQC spectrum in $CDCl_3$ at 298 K of* ***Ga(Q3)$_3$***. S14
Figure S18. *^1H NMR spectrum in $CDCl_3$ at 298 K of* ***Ga(Q4)$_3$*** S15
Figure S19. *Magnification of ^1H NMR spectrum in $CDCl_3$ at 298 K of* ***Ga(Q4)$_3$***. S15
Figure S20. *{^1H,^1H}-COSY spectrum in $CDCl_3$ at 298 K of* ***Ga(Q4)$_3$*** S16
Figure S21. *^{13}C NMR spectrum in $CDCl_3$ at 298 K of* ***Ga(Q4)$_3$*** S16
Figure S22. *Magnification of ^{13}C NMR spectrum in $CDCl_3$ at 298 K of* ***Ga(Q4)$_3$***. S17
Figure S23. *{1H,13C}-HSQC spectrum in CDCl3 at 298 K of* ***Ga(Q4)3***. S17
Figure S24. *Magnification of {^1H,^{13}C}-HSQC spectrum in $CDCl_3$ at 298 K of* ***Ga(Q4)$_3$***. S18
Figure S25. *^1H NMR spectrum in $CDCl_3$ at 298 K of* ***Ga(Q5)$_3$*** S18
Figure S26. *Magnification of ^1H NMR spectrum in $CDCl_3$ at 298 K of* ***Ga(Q5)$_3$***. S19
Figure S27. *{^1H,^1H}-COSY spectrum in $CDCl_3$ at 298 K of* ***Ga(Q5)$_3$*** S20
Figure S28. *^{13}C NMR spectrum in $CDCl_3$ at 298 K of* ***Ga(Q5)$_3$*** S20
Figure S29. *Magnification of ^{13}C NMR spectrum in $CDCl_3$ at 298 K of* ***Ga(Q5)$_3$***. S21
Figure S30. *{^1H,^{13}C}-HSQC spectrum in $CDCl_3$ at 298 K of* ***Ga(Q5)$_3$***. S21
Figure S31. *NMR spectroscopy stability studies of Ga(III) complexes. All the spectra were recorded in DMSO-d_6 solution over a period of 48 h. Representative of ^1H-NMR spectra for complex* ***4***. S22
Figure S32. *Optimized fac and mer isomers of complexes* ***1-5*** *(H atoms were omitted for clarity).* S25
Figure S33. *MOs of the Q_1^- anion showing the σ in-phase and σ out-of-phase combinations.* S25
Figure S34. *Comparison of the HOMO of fac-**1** with the HOMO and HOMO-1 of the HQ_1 precursor and the Q_1^- ligand, respectively.* S26
Figure S35. *Optimized structures of the isomers* ***I, II*** *and* ***III*** *of $Ga(NO_3)(Q_1)_2$ intermediates (H atoms were omitted for clarity).* S26
Figure S36. *Distribution of fac- and mer- isomers of complex* ***1*** *upon substitution of nitrate from $Ga(NO_3)(Q_1)_2$ intermediates by Q_1^- anion.* S27
Figure S37. *Formation of the fac- and mer- isomers of complex* ***1*** *from isomer* ***II*** *of $Ga(NO_3)(Q_1)_2$ (up) and optimized structures of the two isomers of $Ga(\kappa^1\text{-}NO_3)(Q_1)^{3-}$,* ***IV*** *and* ***V*** *(bottom).* S28
Figure S38. *Image of the crystal of complex* ***1*** *on the diffractometer* S56
Figure S39. *Image of the crystal of complex* ***2*** *on the diffractometer.* S72
Figure S40. *Superimposition of best scoring molecular docking models for complexes* ***1-5*** *binding to HSA (PDB ID: 1AO6).* S82
Figure S41. *Changes in fluorescence emission spectra of BSA (black curves) upon titration with 1-5 in the range 0–10 μM (control, black line; 1 μM, blue line; 2 μM, purple line; 5 μM, red line; 10 μM, green line).* S82
Figure S42. *Comparison of mono-exponential binding kinetics for compounds* ***1-5*** *to surface-blocked DNA 30-mer.* S83
Figure S43. *Changes in fluorescence emission spectra of DAPI-DNA complex upon titration with complexes* ***1-5*** *in the range 1-200 μM.* S83

Figure S44. *Changes in absorbance at 630 nm of Methyl green-DNA complex upon titration with complexes* ***1-5*** *in the range 1-200 µM.* S83
Figure S45. *Comparison of the best scoring complexes formed upon docking* ***2*** *(Panel A),* ***4*** *(Panel B) and* ***5*** *(Panel C) on dsDNA (prepared and energy minimized using Avogadro). Major groove is emphasized as solid cyan surface (left panels).* S84
Figure S46. *Visualization of changes in emission anisotropy with time observed upon Caco-2 cell membrane passage of* ***1*** *(left panel). Kinetic analysis of entry and release stages (right panel).* S85
Figure S47. *Visualization of changes in emission anisotropy with time observed upon Caco-2 cell membrane passage of* ***2*** *(left panel). Kinetic analysis of entry and release stages (right panel).* S85
Figure S48. *Visualization of changes in emission anisotropy with time observed upon Caco-2 cell membrane passage of* ***3****.* S85
Figure S49. *Visualization of changes in emission anisotropy with time observed upon Caco-2 cell membrane passage of* ***4*** *(left panel). Kinetic analysis of entry and release stages (right panel).* S86
Figure S50. *Visualization of changes in emission anisotropy with time observed upon Caco-2 cell membrane passage of* ***5*** *(left panel). Kinetic analysis of entry and release stages (right panel).* S86
Figure S51. *Molecular docking of the crystallographic structure of* ***4*** *within the catalytic region of HMG-CoA reductase (PDB ID: 1HW8). Surface, and cartoon and stick representations are reported in left and right panels, respectively. Residues involved in the formation of the complex are rendered as light violet.* S87
Figure S52. *Competitive binding assay of complex* ***4*** *to HMGR. Comparative binding of* ***4*** *to surface-blocked HMGR in the presence and in the absence of HMG-CoA (left panel) and NADPH (right panel).* S88
Figure S53. *Effect of complex* ***4*** *on cell cycle distribution in Caco-2 cell* S88
Figure S54. *Increase in cytochrome p450 oxidase activity after 48 h treatment of Caco-2, Caco-2CR and CRL-1831 cells with complex* ***4*** *in the presence and in the absence of ferrostatin-1, calculated as percentage decrease in the levels of reduced cytochrome c.* S89
Figure S55. *Effect of complex* ***4*** *on the main proteolytic activities of 20S proteasome after 48 h treatment of Caco-2, Caco-2CR and CRL-1831 cells in the presence and in the absence of Fer-1.* S90
Figure S56. *Cytotoxic effect of different concentrations of complex* ***4*** *on Caco-2 (Left Panel) and on Caco-2CR (Right Panel) cells viability in the absence (l, orange line) and in the presence (n, yellow line) of 60 nM of ferrostatin.* S90
Figure S57. *Changes in caspase 3 activity upon treatment of Caco-2, Caco-2CR and CRL-1831 cells with complex* ***4****, in the presence and in the absence 60 nM of ferrostatin.* S91
Figure S58. *Analysis of ΔΨm changes in Caco-2 cell line treated for 48 h with medium, vehicle, and 10 µM of complex* ***4*** *evaluated by JC-1 staining and biparametric FL1(green)/FL2(red) flow cytometric analysis. Numbers in the bottom right square indicate the percenta* S91
Figure S59. *HPLC chromatographic profiles of complexes* ***1-5****.* S92

List of Tables

Table S1. *Comparison of selected computed and experimental structural parameters of compound* ***1****, fac and* ***2****, mer.* S29
Table S2. *Energies (Hartree) and relative energy differences (kcal/mol) for the isomers of complexes* ***1-5****.* S30
Table S3. *Coordinates of optimized compounds.* S31
Table S4. *Crystal data for complex* ***1*** S54
Table S5. *Structure Quality Indicators for complex* ***1*** S55
Table S6. *Data Plots: Diffraction Data for complex* ***1*** S57
Table S7. *Reflection Statistics for complex* ***1*** S58
Table S8. *Fractional Atomic Coordinates (×10^4) and Equivalent Isotropic Displacement Parameters (Å2×10^3) for complex 1. U_{eq} is defined as 1/3 of the trace of the orthogonalised U_{ij}.* S58
Table S9. *Anisotropic Displacement Parameters (×10^4) for complex* ***1****. The anisotropic displacement factor exponent takes the form: $-2\pi^2[h^2a^{*2} \times U_{11} + ... +2hka^* \times b^* \times U_{12}]$* S60
Table S10. *Bond Lengths (Å) in complex* ***1*** S62
Table S11. *Bond Angles in ° for complex* ***1*** S63
Table S12. *Torsion Angles in ° for complex* ***1*** S65

Table S13. *Hydrogen Fractional Atomic Coordinates (×10^4) and Equivalent Isotropic Displacement Parameters (Å^2×10^3) for complex* ***1****. U_{eq} is defined as 1/3 of the trace of the orthogonalised U_{ij}.* S68
Table S14. *Atomic Occupancies for all atoms that are not fully occupied in complex* ***1****.* S69
Table S15. *Solvent masking (PLATON/SQUEEZE) information for complex* S69
Table S16. *Crystal data for complex* ***2*** S70
Table S17. *Structure Quality Indicators for complex* ***2*** S71
Table S18. *Data plots of diffraction data for complex* ***2*** S72
Table S19. *Refinement and Data plots for complex* ***2*** S73
Table S20. *Reflection statistics for complex* ***2*** S73
Table S21. *Fractional Atomic Coordinates (×10^4) and Equivalent Isotropic Displacement Parameters (Å^2×10^3) for* **complex 2***. U_{eq} is defined as 1/3 of the trace of the orthogonalised U_{ij}.* S73
Table S22. *Anisotropic Displacement Parameters (×10^4) for* **complex 2***. The anisotropic displacement factor exponent takes the form: $-2\pi^2[h^2a^{*2} \times U_{11}+ ... +2hka^* \times b^* \times U_{12}]$* S74
Table S23. *Bond lengths in Å for complex* ***2*** S76
Table S24. *Bond Angles in ˚ for complex* ***2*** S77
Table S25. *Torsion Angles in ° for complex* ***2*** S78
Table S26. *Hydrogen Fractional Atomic Coordinates (×10^4) and Equivalent Isotropic Displacement Parameters (Å^2×10^3) for complex* ***2****. U_{eq} is defined as 1/3 of the trace of the orthogonalised U_{ij}.* S81
Table S27. *Atomic Occupancies for all atoms that are not fully occupied in complex* ***2*** S81
Table S28. *Representative comparison of equilibrium and kinetic parameters for the interaction between complex* ***4*** *and BSA.* S83
Table S29. *Rate constants for membrane entry (k_{in}) and release from membrane (k_{out}) of complex* ***1****,* ***2****,* ***4*** *and* ***5****.* S86
Table S30. *Calculated lipophilicity of complexes* ***1-5*** *expressed as logarithm of octanol/water partition coefficient (Molinspiration Cheminformatics web services, https://www.molinspiration.com, Slovensky Grob, Slovakia).* S87
Table S31. *HPLC analysis of complex* ***1****.* S92
Table S32*. HPLC analysis of complex* ***2****.* S93
Table S33*. HPLC analysis of complex* ***3****.* S94
Table S34. *HPLC analysis of complex* ***4****.* S95
Table S35*. HPLC analysis of complex* ***5****.* S96

Relevant NMR spectra

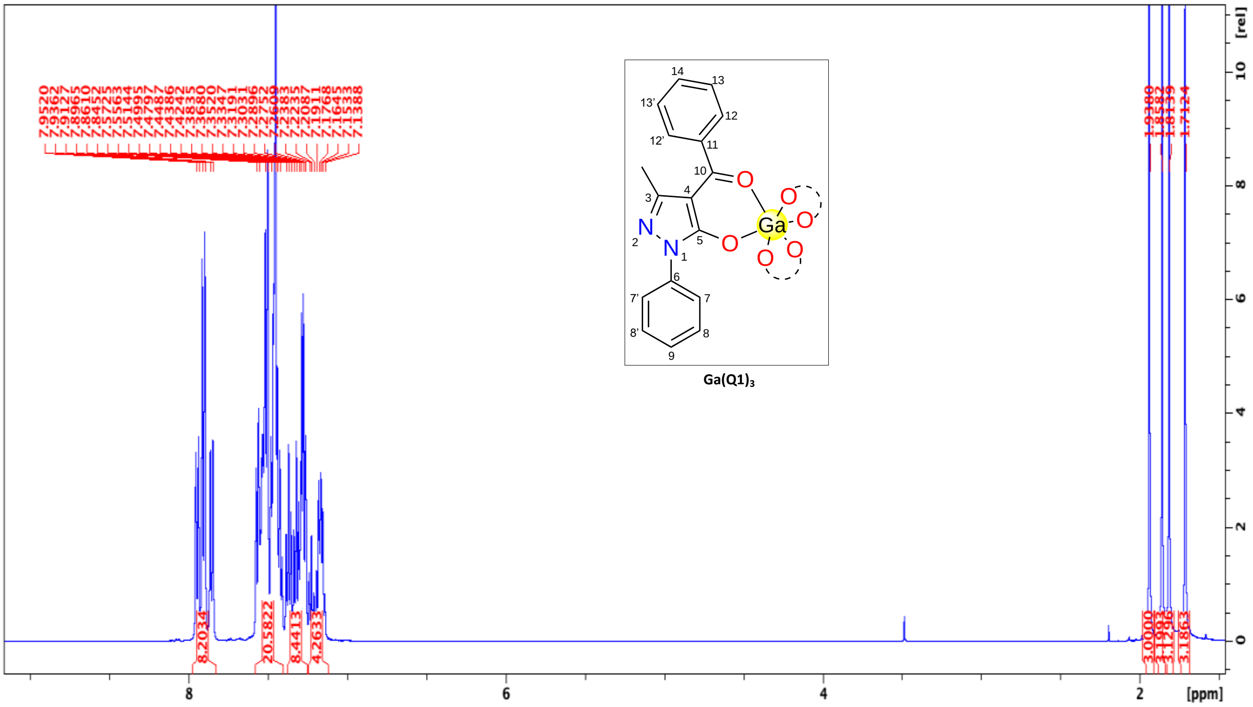

Figure S1. ^{1}H NMR spectrum in $CDCl_3$ at 298 K of **Ga(Q1)$_3$**

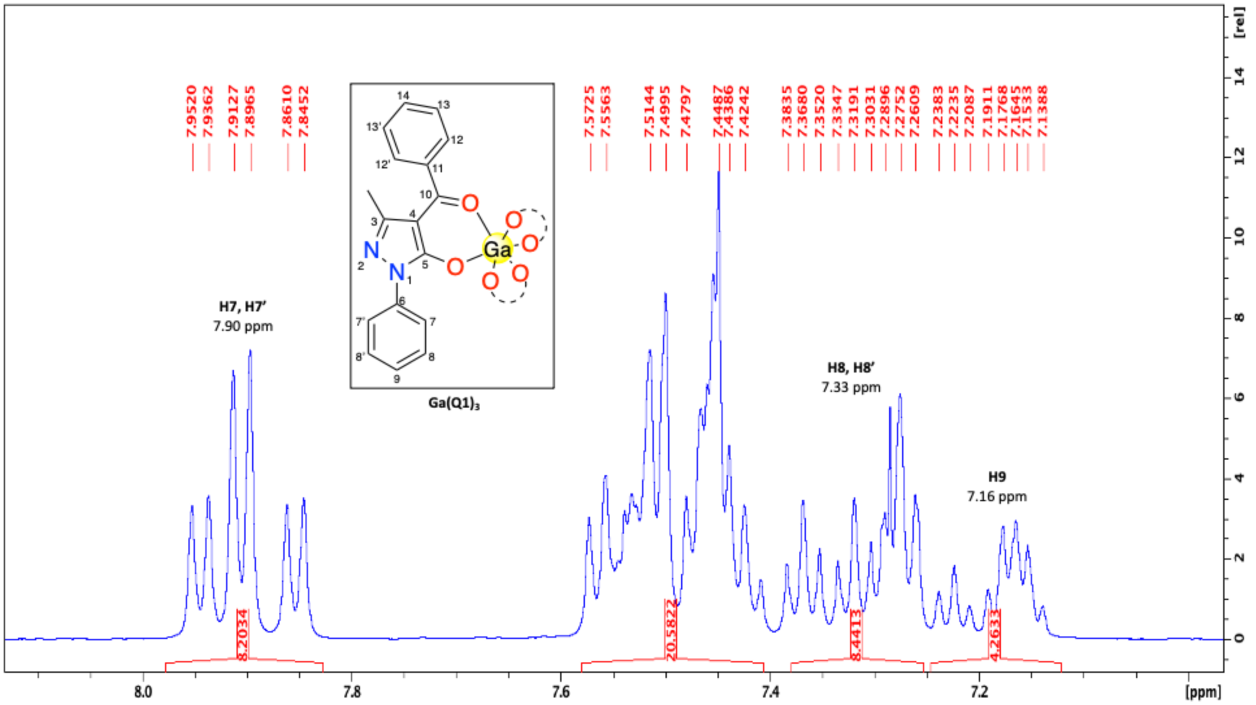

Figure S2. Magnification of ^{1}H NMR spectrum in CDCl3 at 298 K of **Ga(Q1)$_3$**

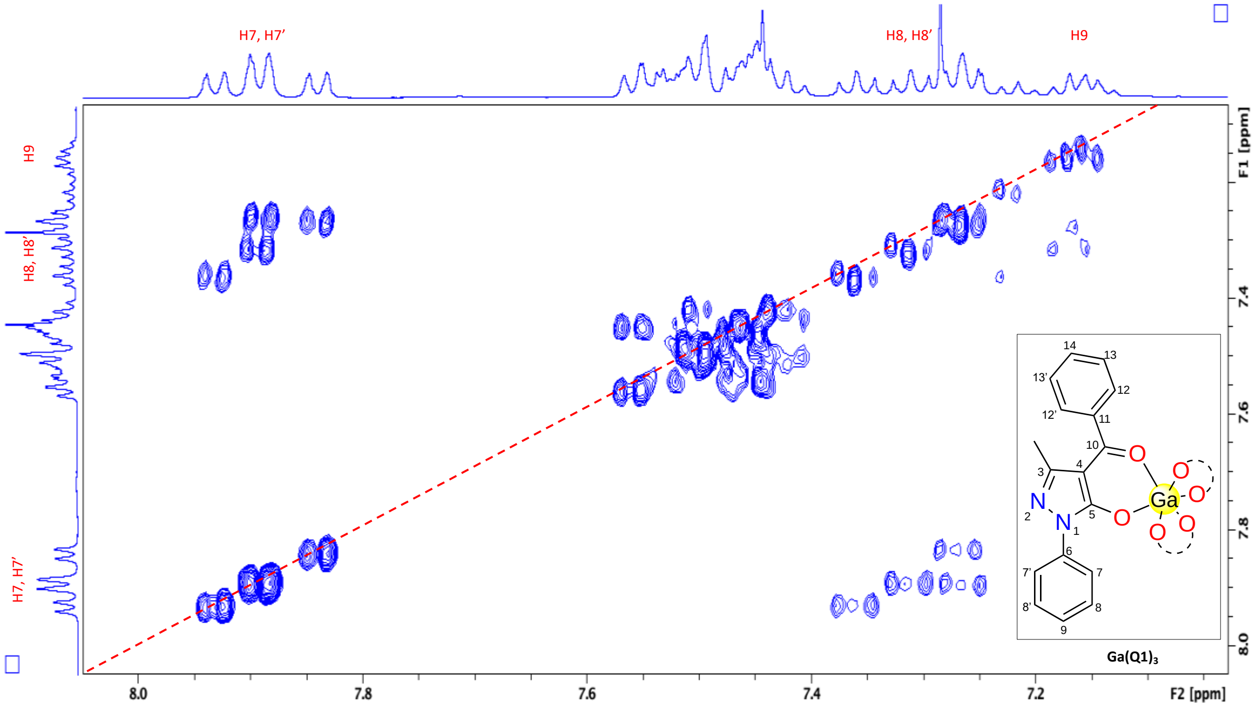

Figure S3. Magnification of {^{1}H, ^{1}H}-COSY spectrum in $CDCl_3$ at 298 K of **Ga(Q1)$_3$**

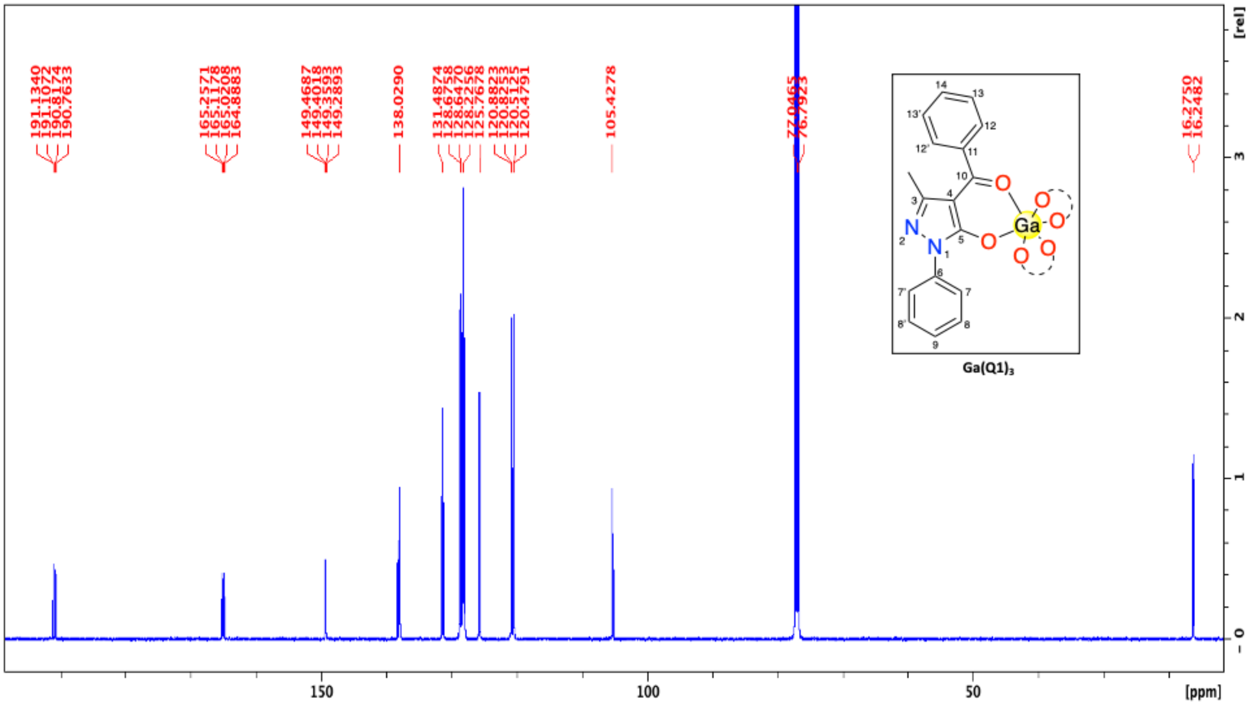

Figure S4. ^{13}C NMR spectrum in $CDCl_3$ at 298 K of **Ga(Q1)$_3$**

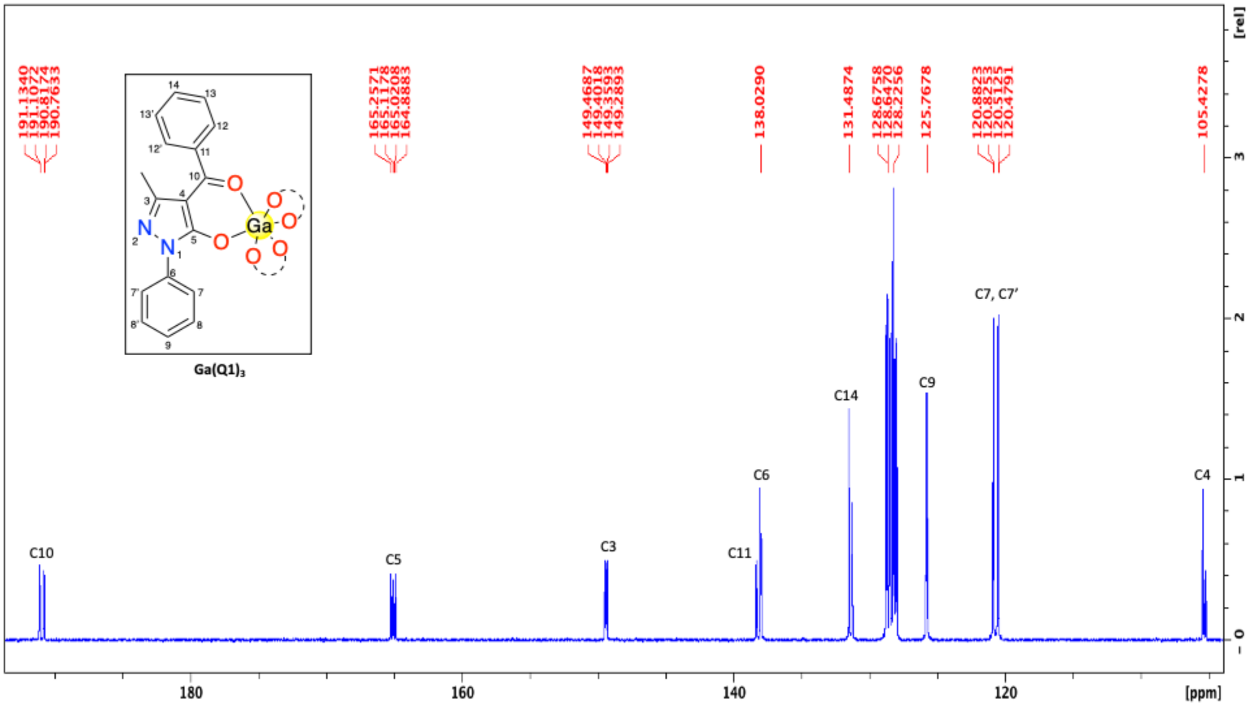

Figure S5. Magnification of ^{13}C NMR spectrum in $CDCl_3$ at 298 K of **Ga(Q1)$_3$**

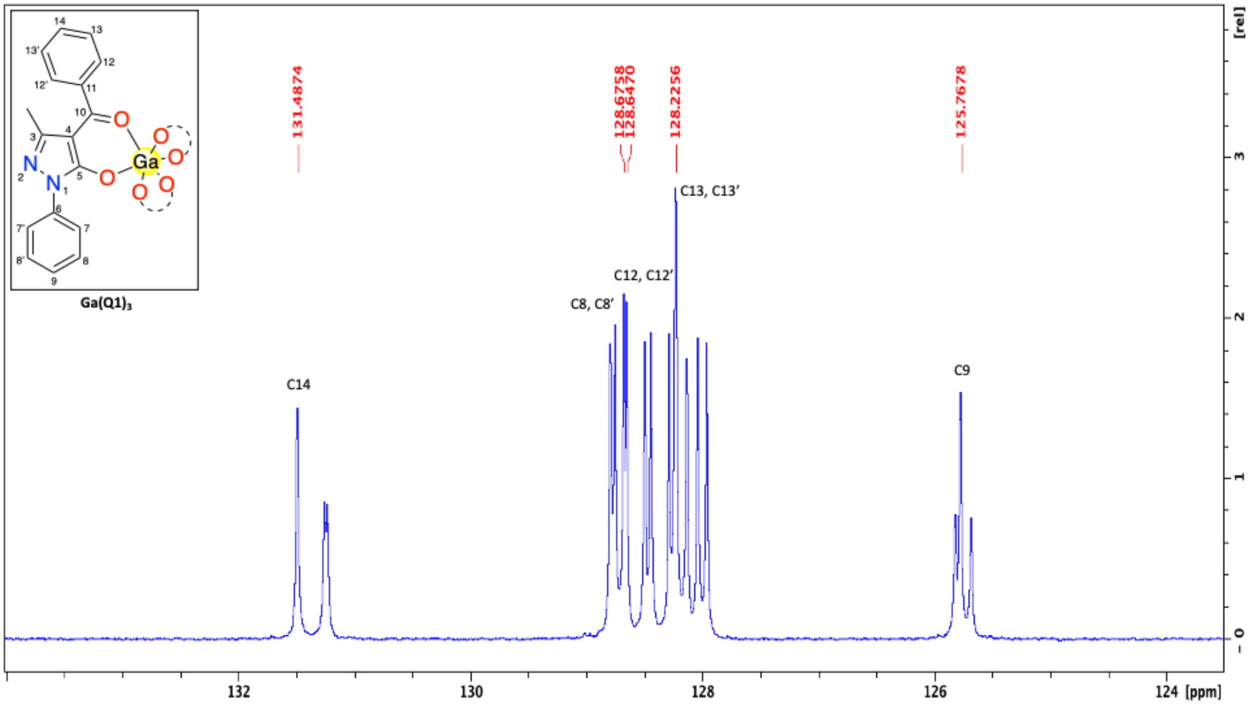

Figure S6. Magnification of ^{13}C NMR spectrum in $CDCl_3$ at 298 K of **Ga(Q1)$_3$**

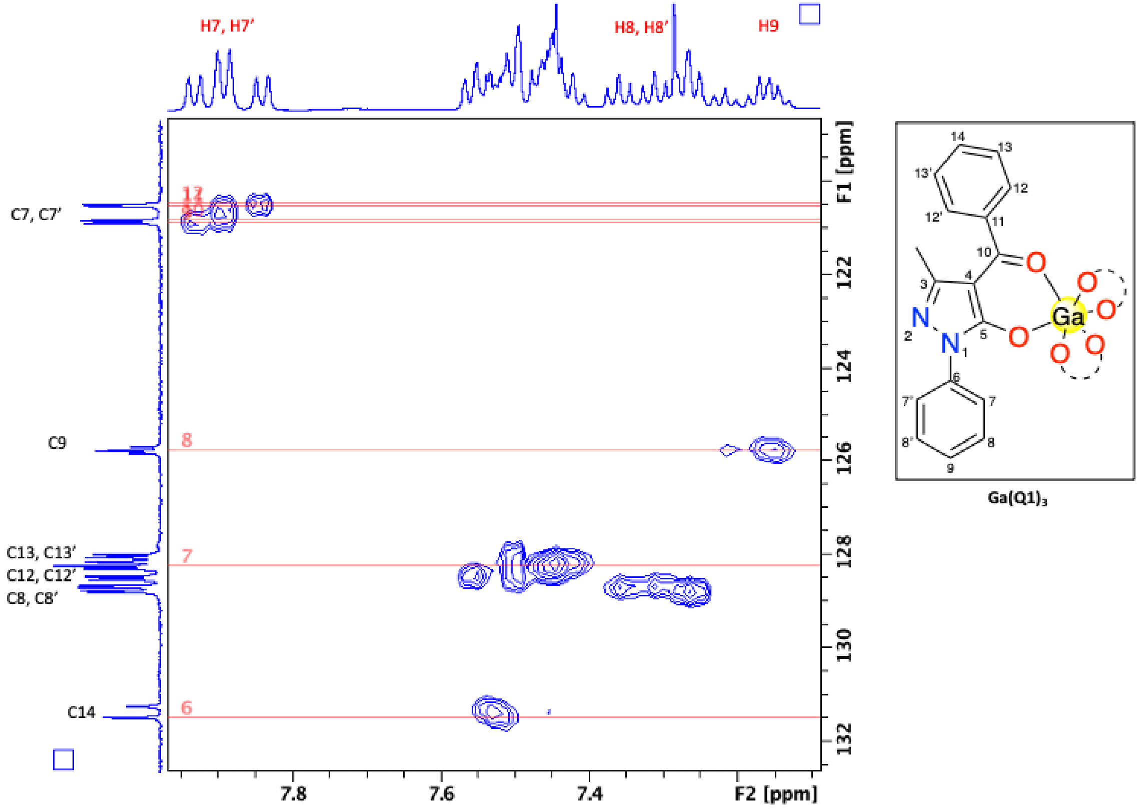

Figure S7. Magnification of {^{1}H, ^{13}C}-HSQC spectrum in $CDCl_3$ at 298 K of **Ga(Q1)$_3$**.

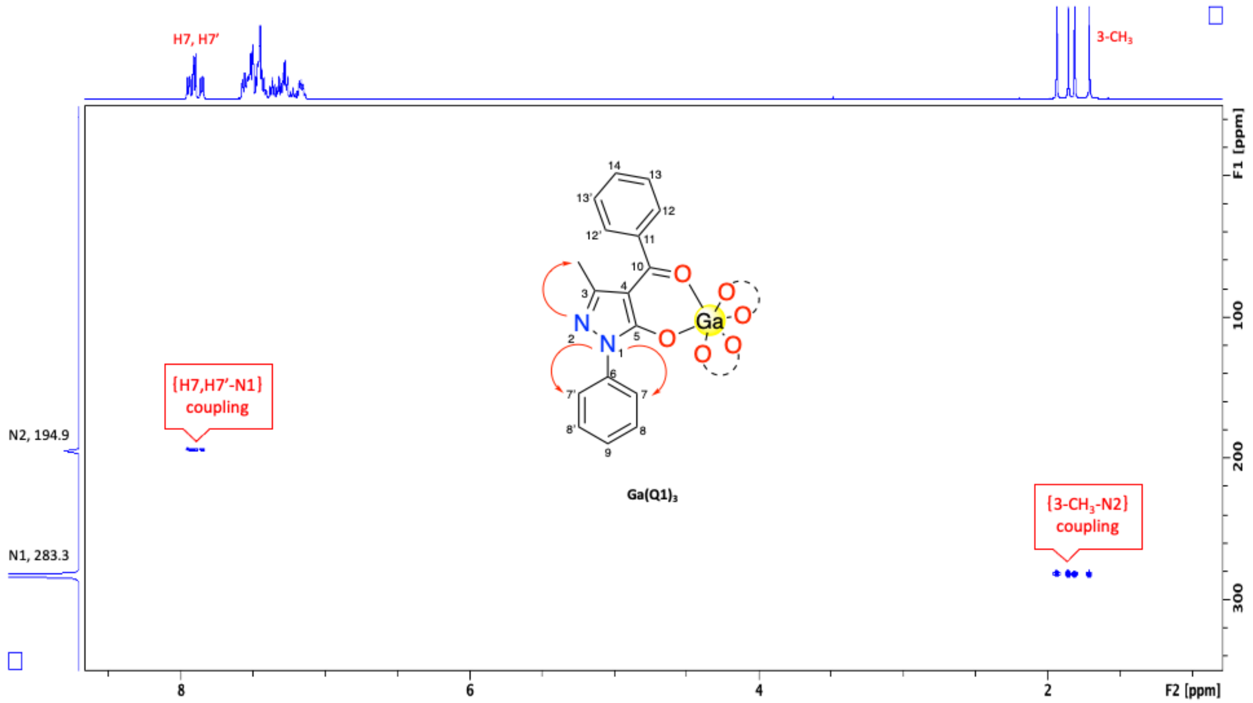

Figure S8. {^{1}H, ^{15}N}-HMBC spectrum in $CDCl_3$ at 298 K of **Ga(Q1)3**

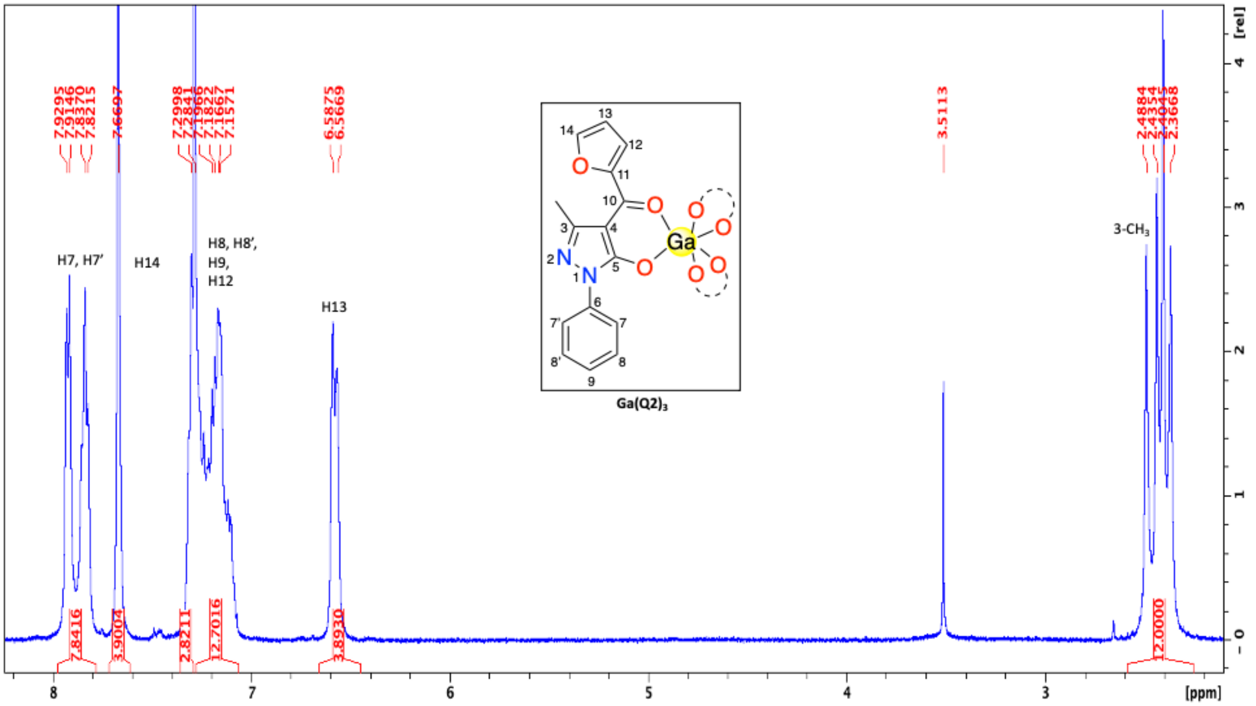

Figure S9. ^{1}H NMR spectrum in $CDCl_3$ at 298 K of **Ga(Q2)3**.

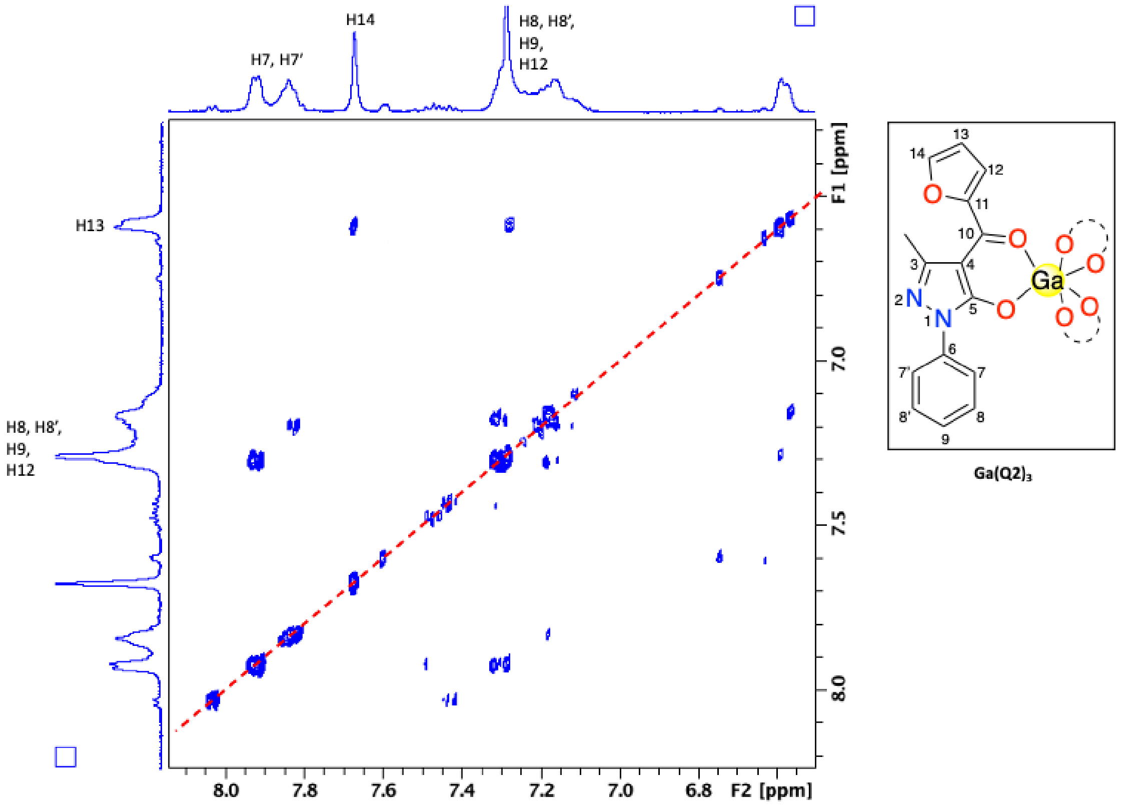

Figure S10. {^{1}H,^{1}H}-COSY spectrum in $CDCl_3$ at 298 K of **Ga(Q2)**$_3$.

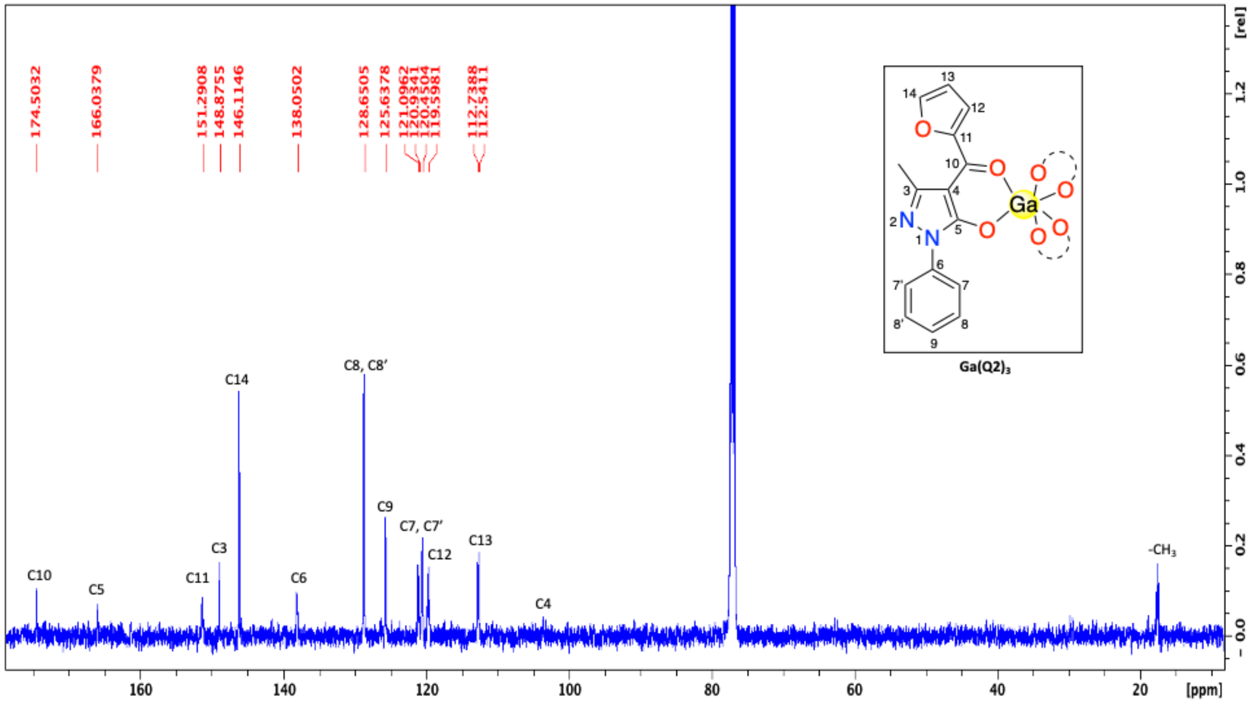

Figure S11. ^{13}C NMR spectrum in $CDCl_3$ at 298 K of **Ga(Q2)**$_3$.

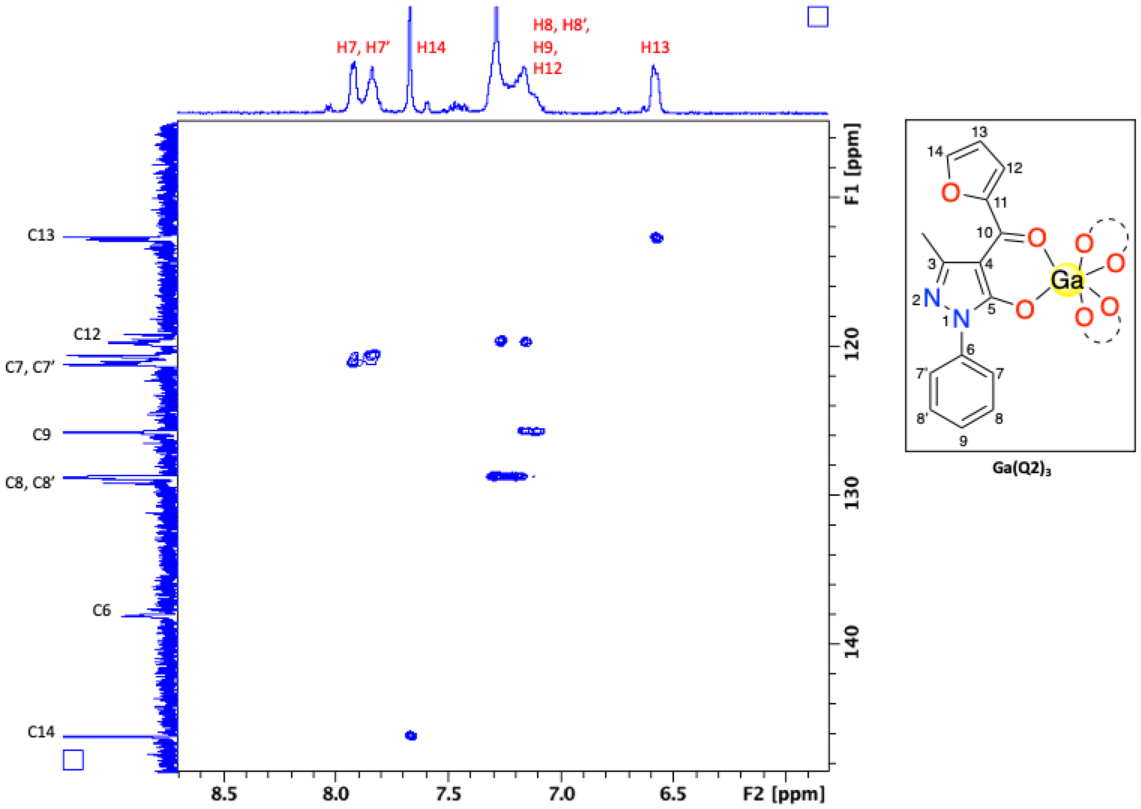

Figure S12. {^{1}H,^{13}C}-HSQC spectrum in $CDCl_3$ at 298 K of **Ga(Q2)$_3$**.

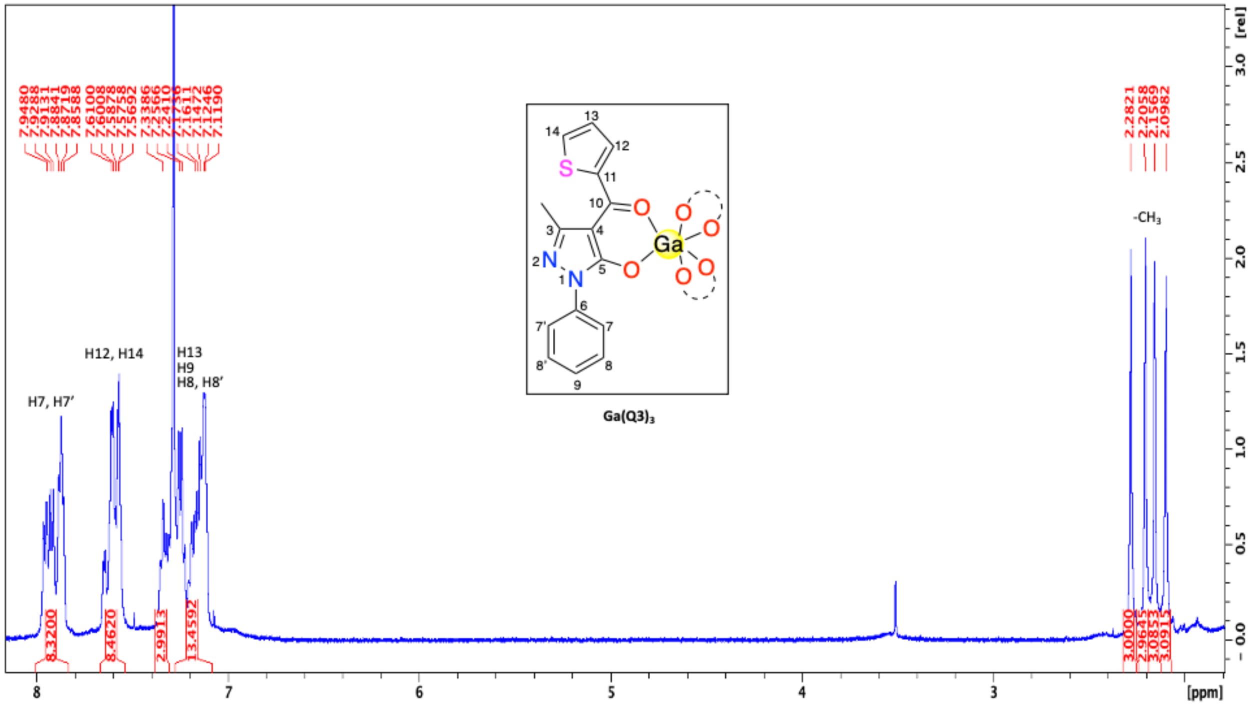

Figure S13. ^{1}H NMR spectrum in $CDCl_3$ at 298 K of **Ga(Q3)$_3$**.

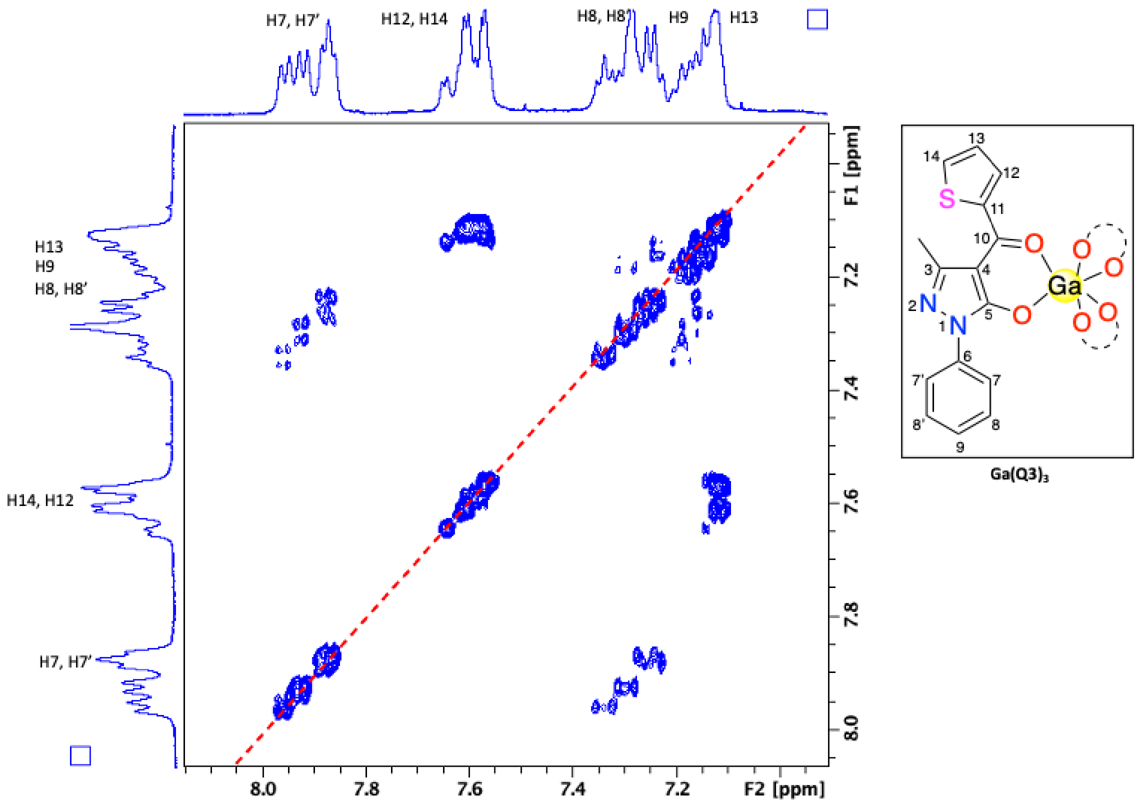

Figure S14. {^{1}H,^{1}H}-COSY spectrum in $CDCl_3$ at 298 K of **Ga(Q3)$_3$**.

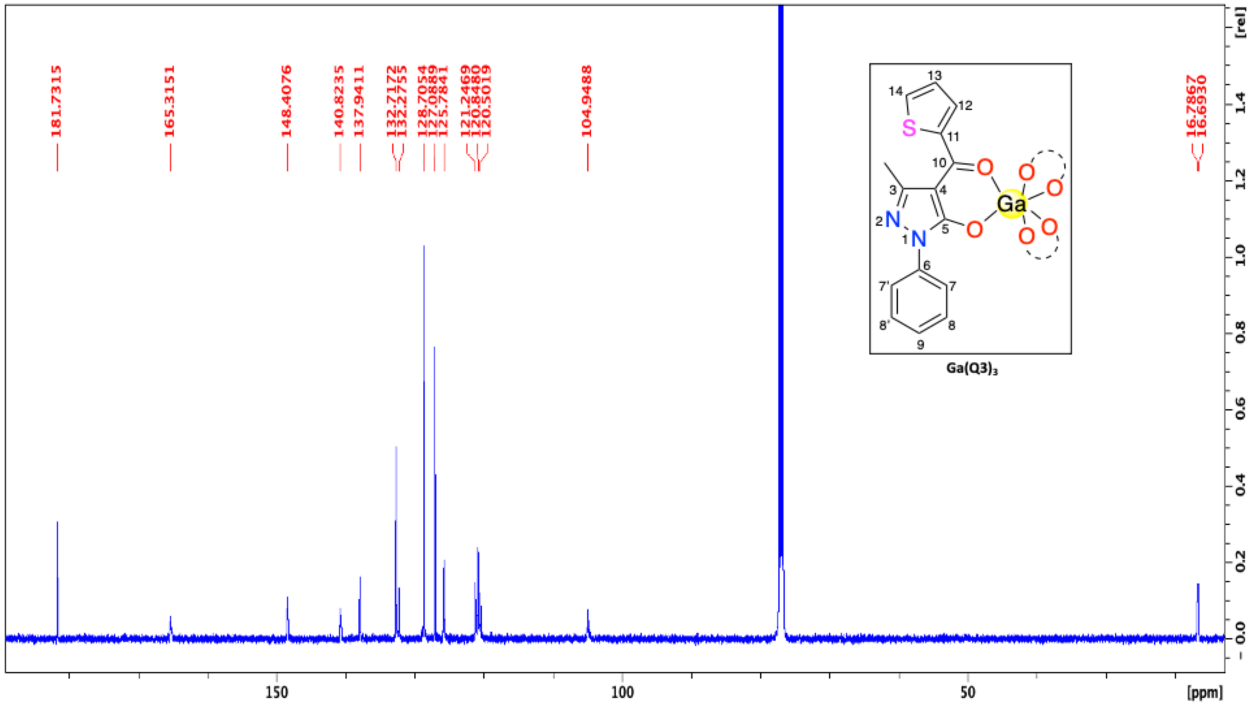

Figure S15. ^{13}C NMR spectrum in $CDCl_3$ at 298 K of **Ga(Q3)$_3$**.

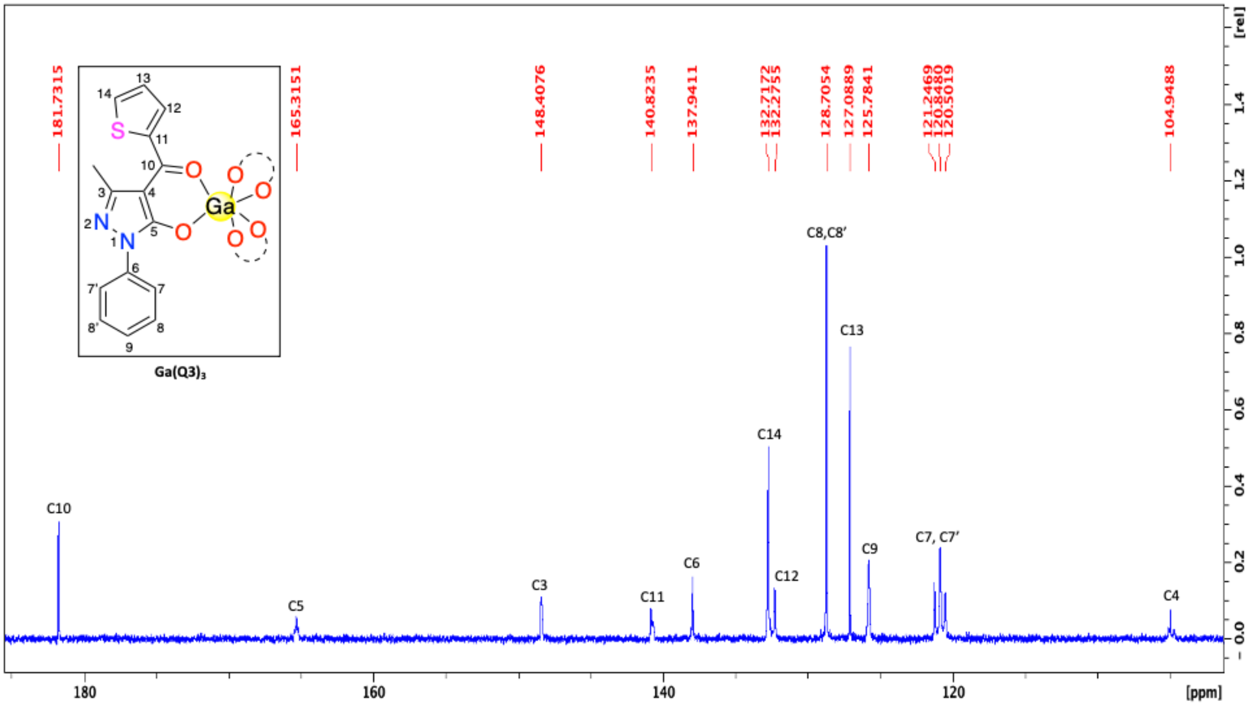

Figure S16. Magnification of ^{13}C NMR spectrum in $CDCl_3$ at 298 K of **Ga(Q3)$_3$**.

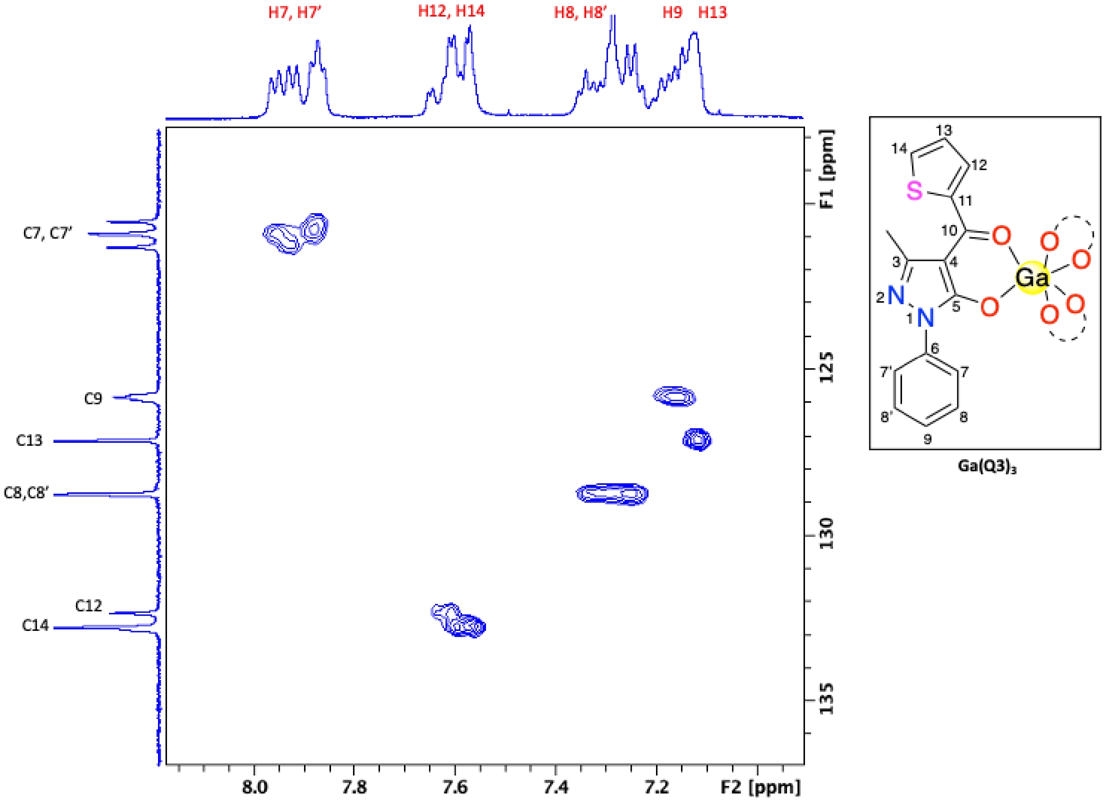

Figure S17. {^1H,^{13}C}-HSQC spectrum in $CDCl_3$ at 298 K of **Ga(Q3)$_3$**.

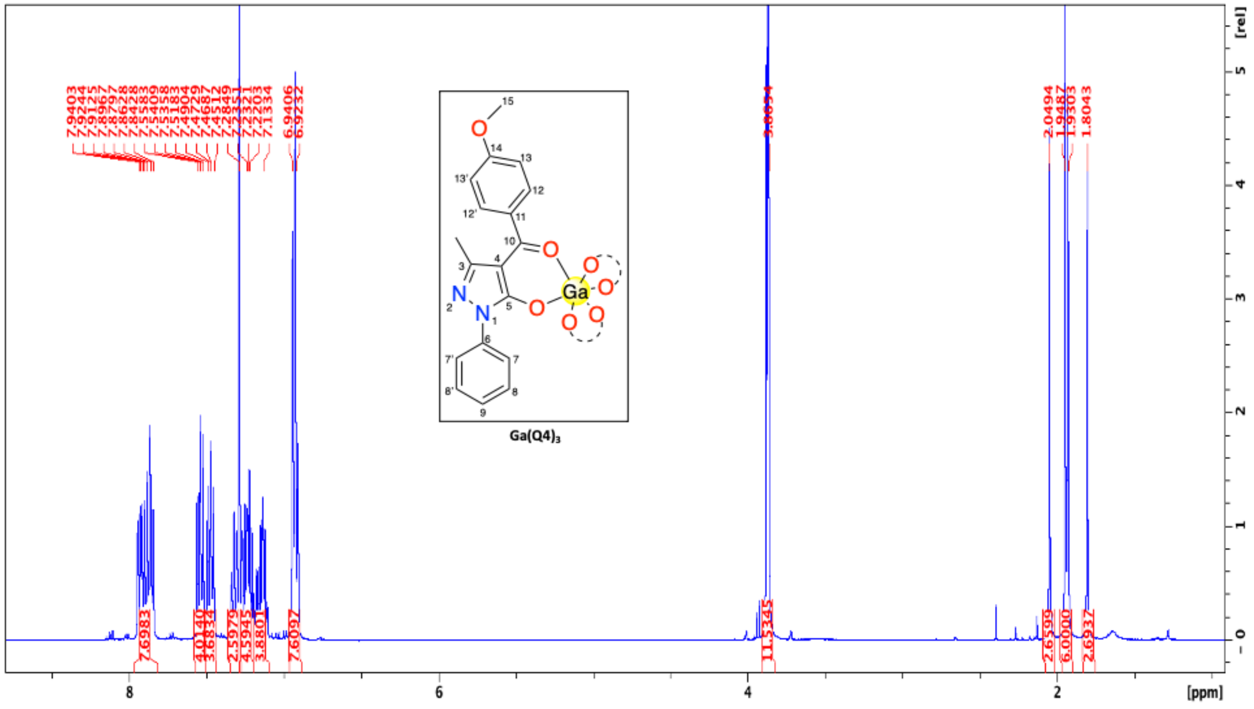

Figure S18. ^{1}H NMR spectrum in $CDCl_3$ at 298 K of **Ga(Q4)$_3$**.

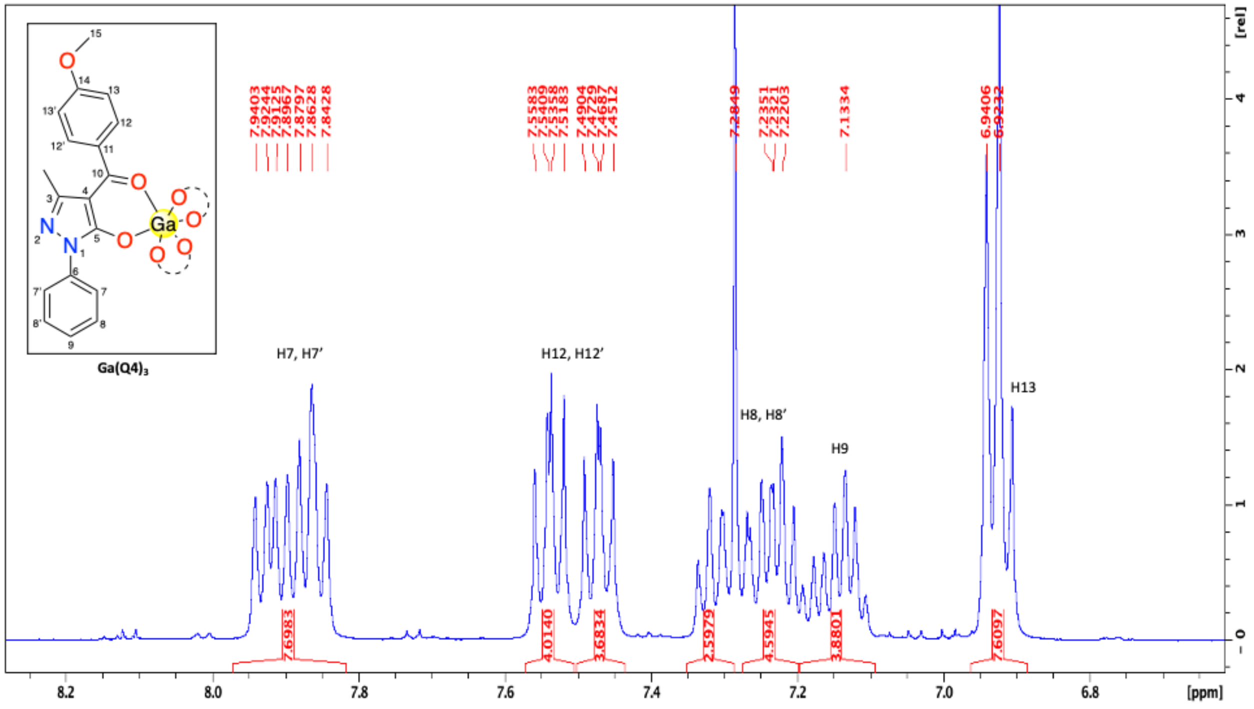

Figure S19. Magnification of ^{1}H NMR spectrum in $CDCl_3$ at 298 K of **Ga(Q4)$_3$**.

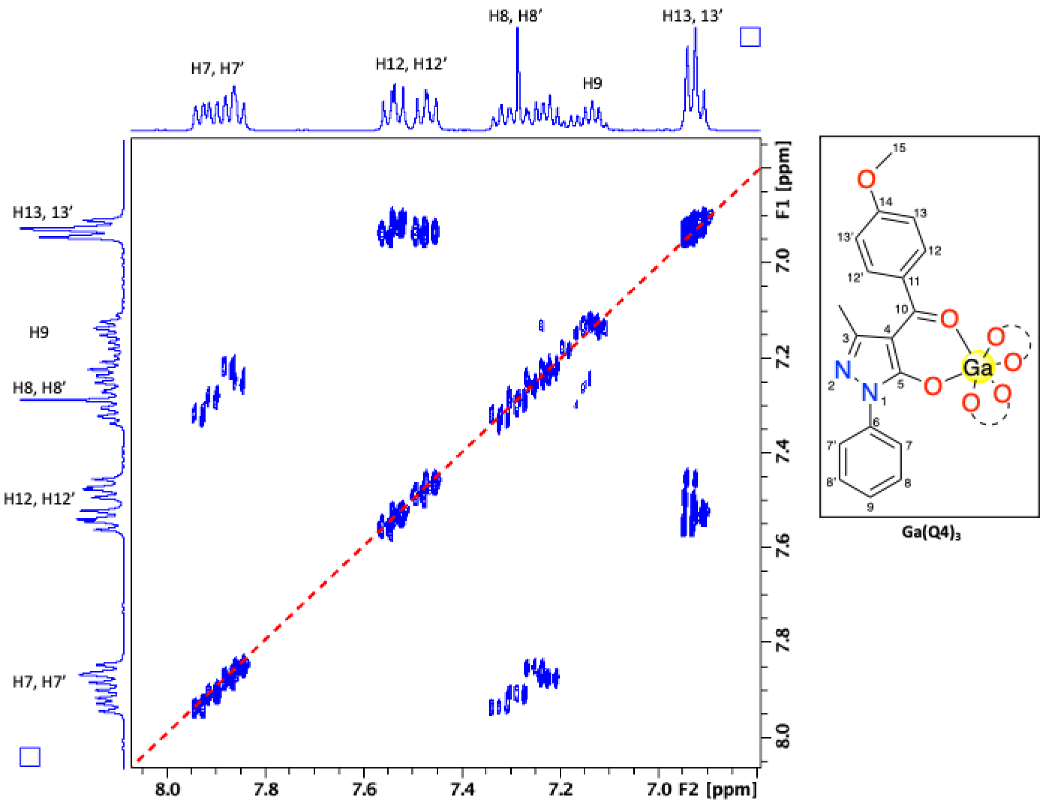

Figure S20. {^{1}H,^{1}H}-COSY spectrum in $CDCl_3$ at 298 K of **Ga(Q4)$_3$**.

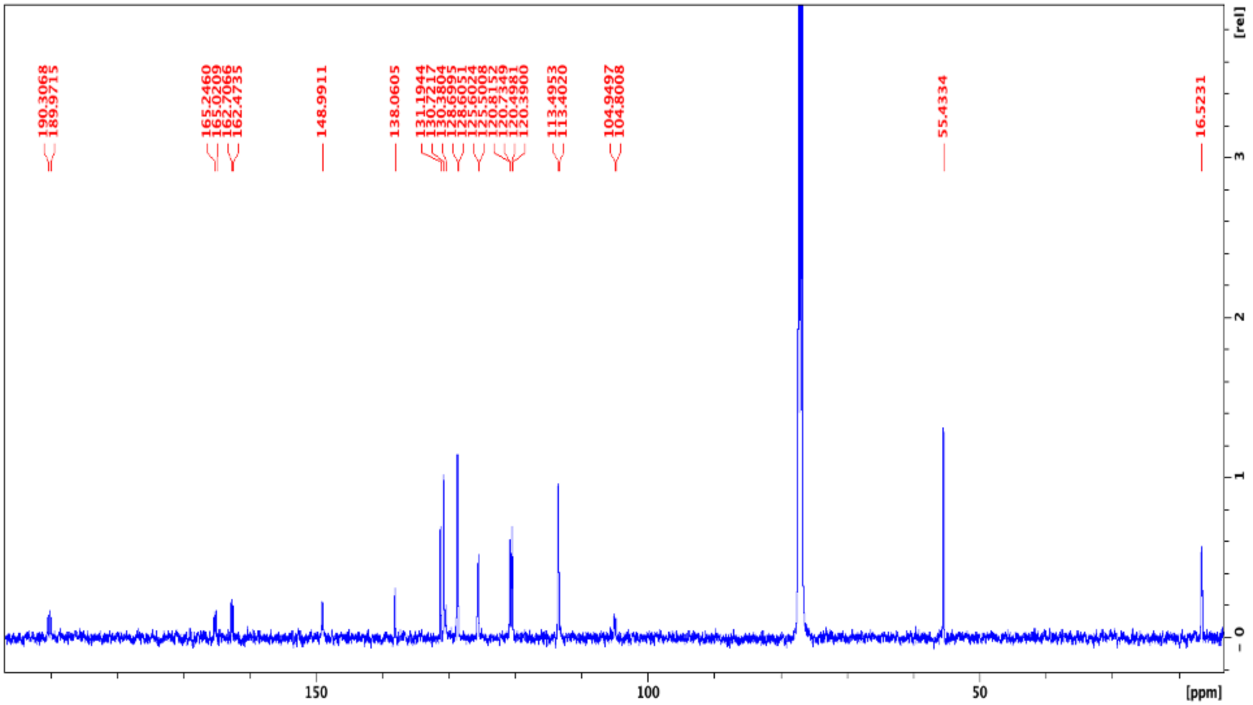

Figure S21. ^{13}C NMR spectrum in $CDCl_3$ at 298 K of **Ga(Q4)$_3$**.

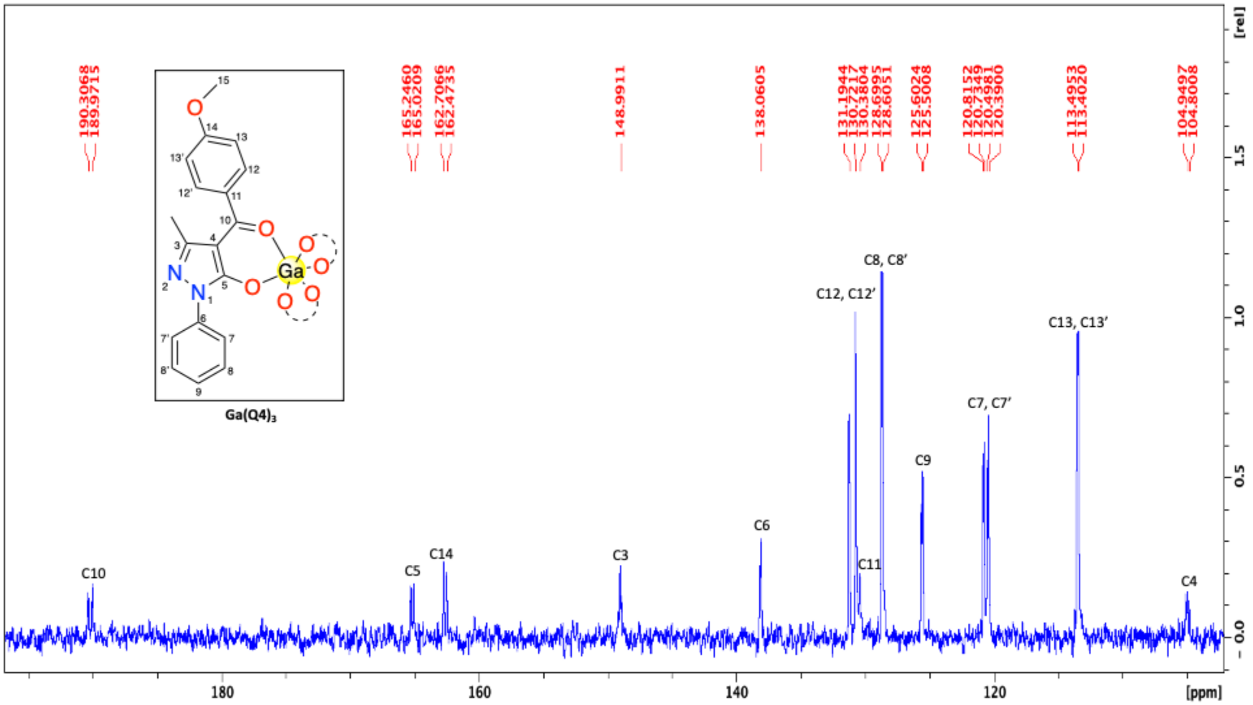

Figure S22. Magnification of ^{13}C NMR spectrum in $CDCl_3$ at 298 K of **Ga(Q4)$_3$**.

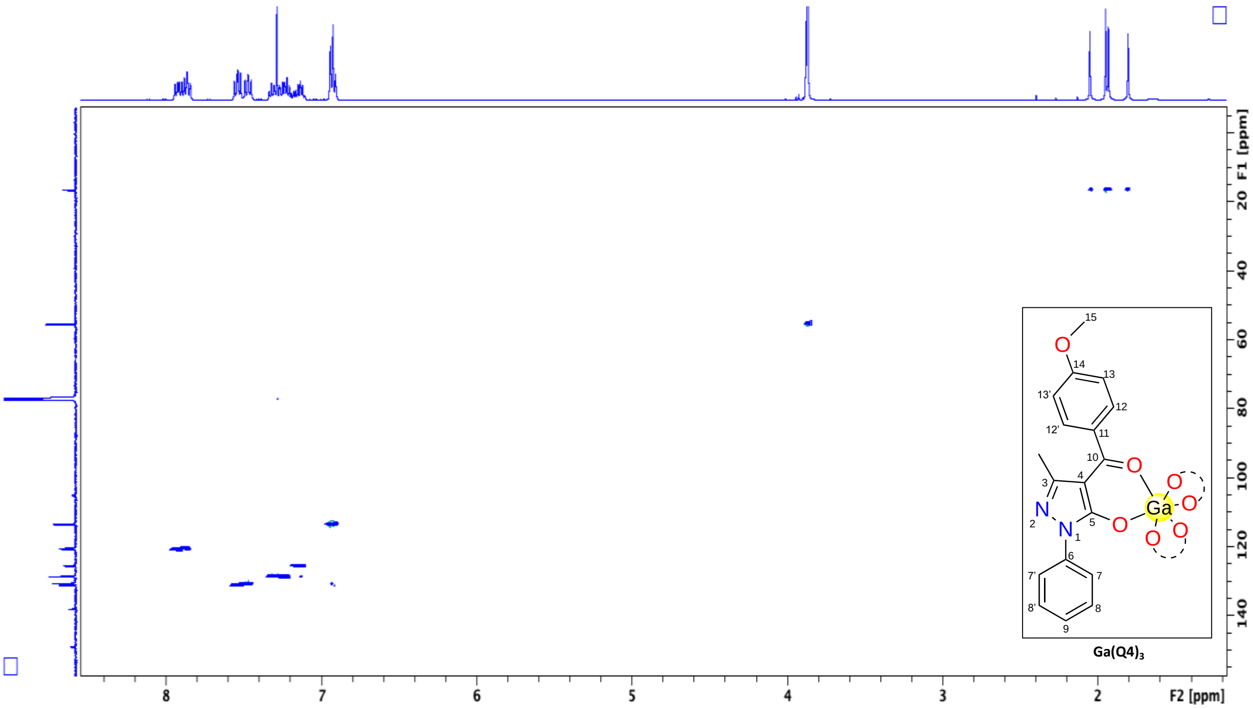

Figure S23. {1H,13C}-HSQC spectrum in CDCl3 at 298 K of **Ga(Q4)3**.

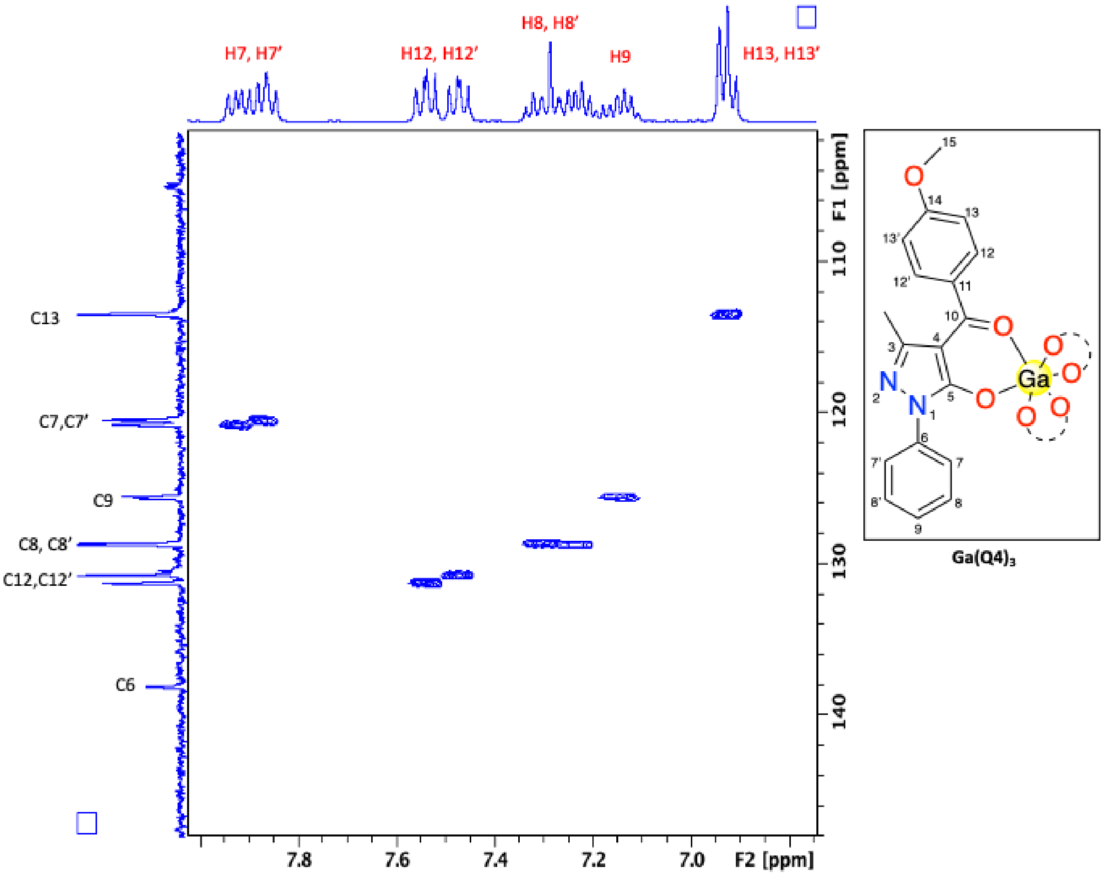

Figure S24. Magnification of {^{1}H,^{13}C}-HSQC spectrum in $CDCl_3$ at 298 K of **Ga(Q4)**$_3$.

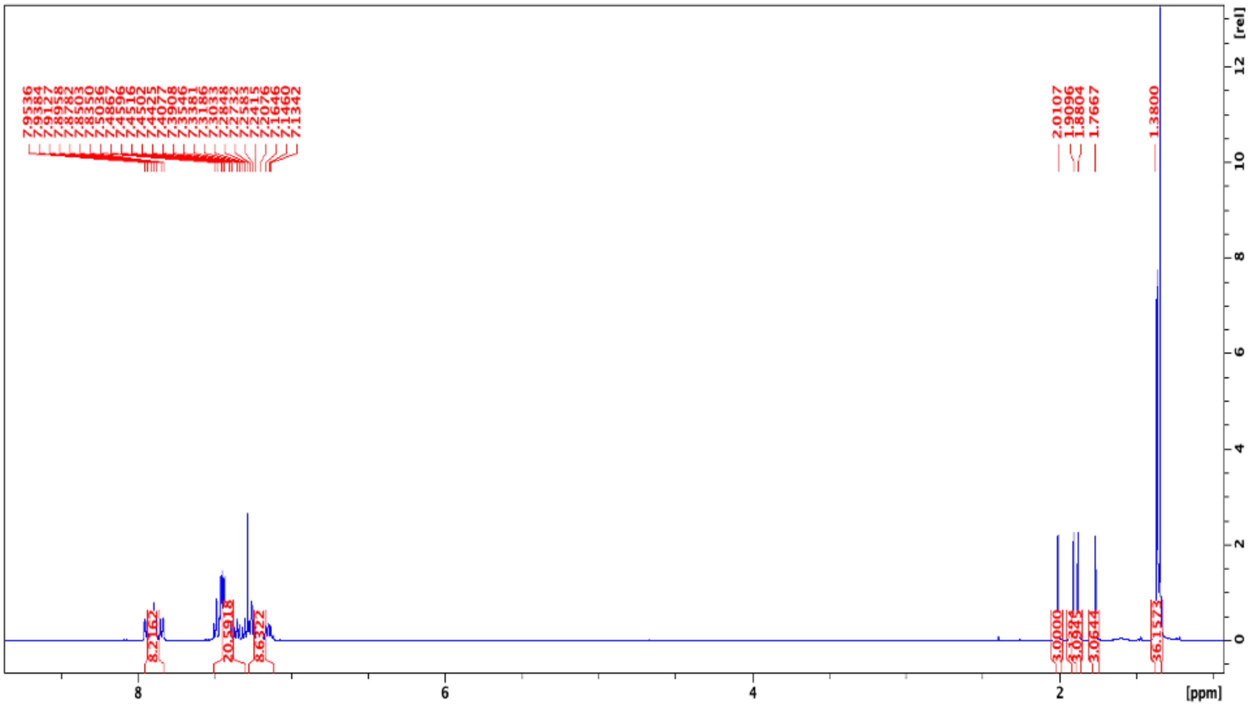

Figure S25. ^{1}H NMR spectrum in $CDCl_3$ at 298 K of **Ga(Q5)**$_3$.

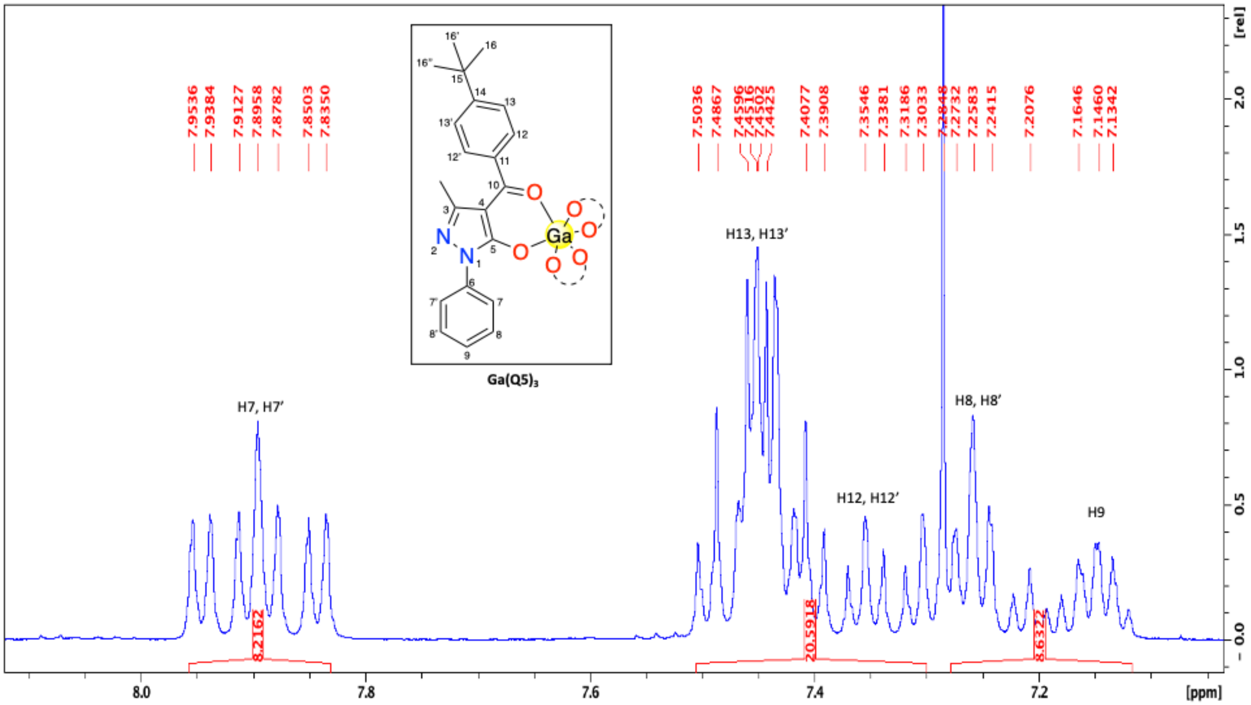

Figure S26. Magnification of ^{1}H NMR spectrum in $CDCl_3$ at 298 K of **Ga(Q5)$_3$**.

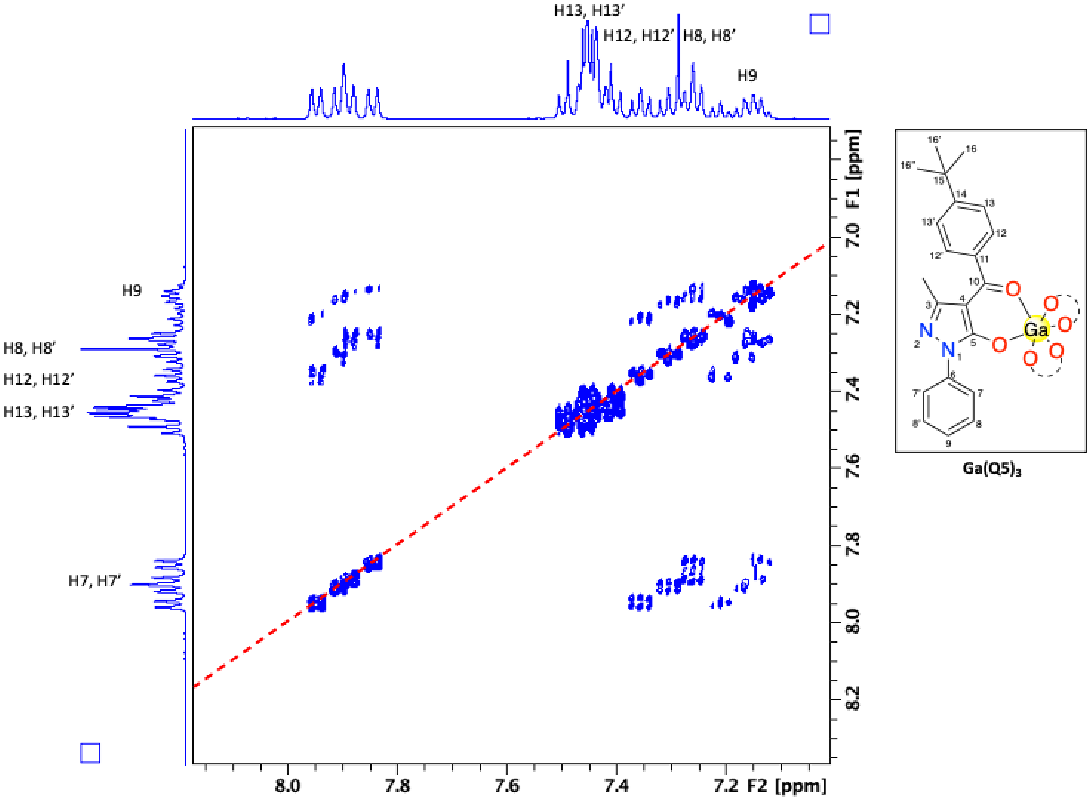

Figure S27. {^{1}H,^{1}H}-COSY spectrum in $CDCl_3$ at 298 K of **Ga(Q5)$_3$**.

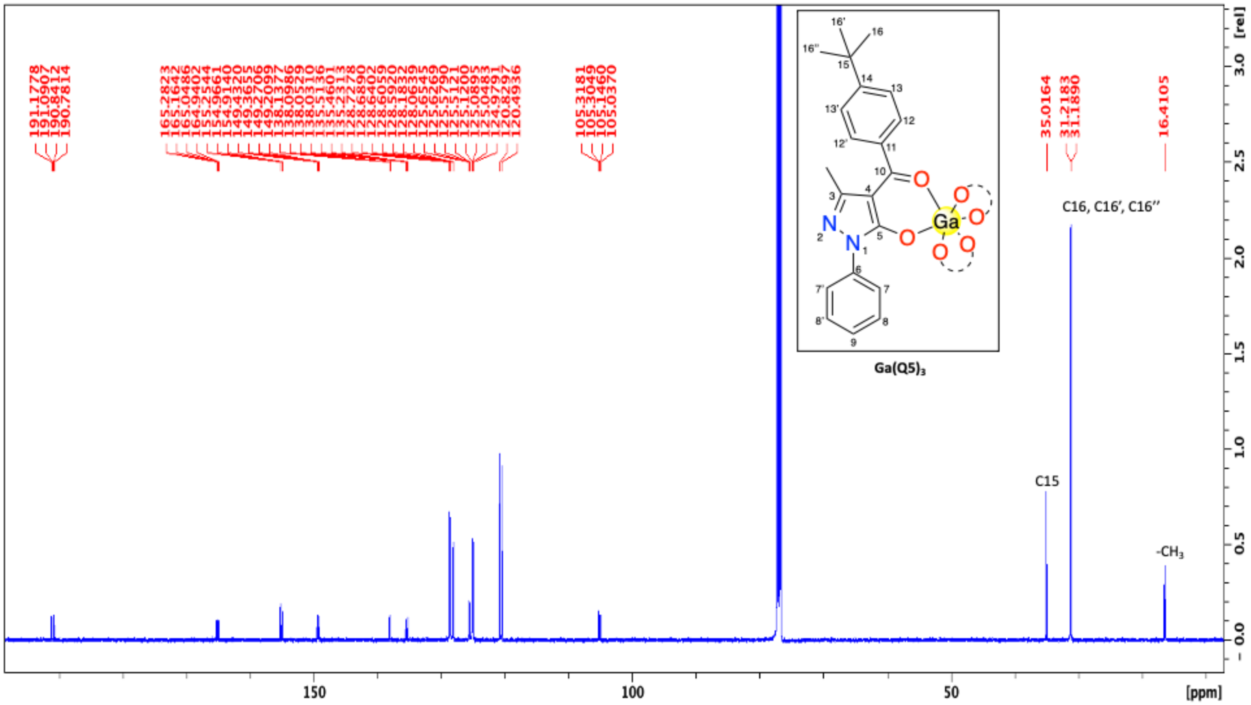

Figure S28. ^{13}C NMR spectrum in $CDCl_3$ at 298 K of **Ga(Q5)$_3$**.

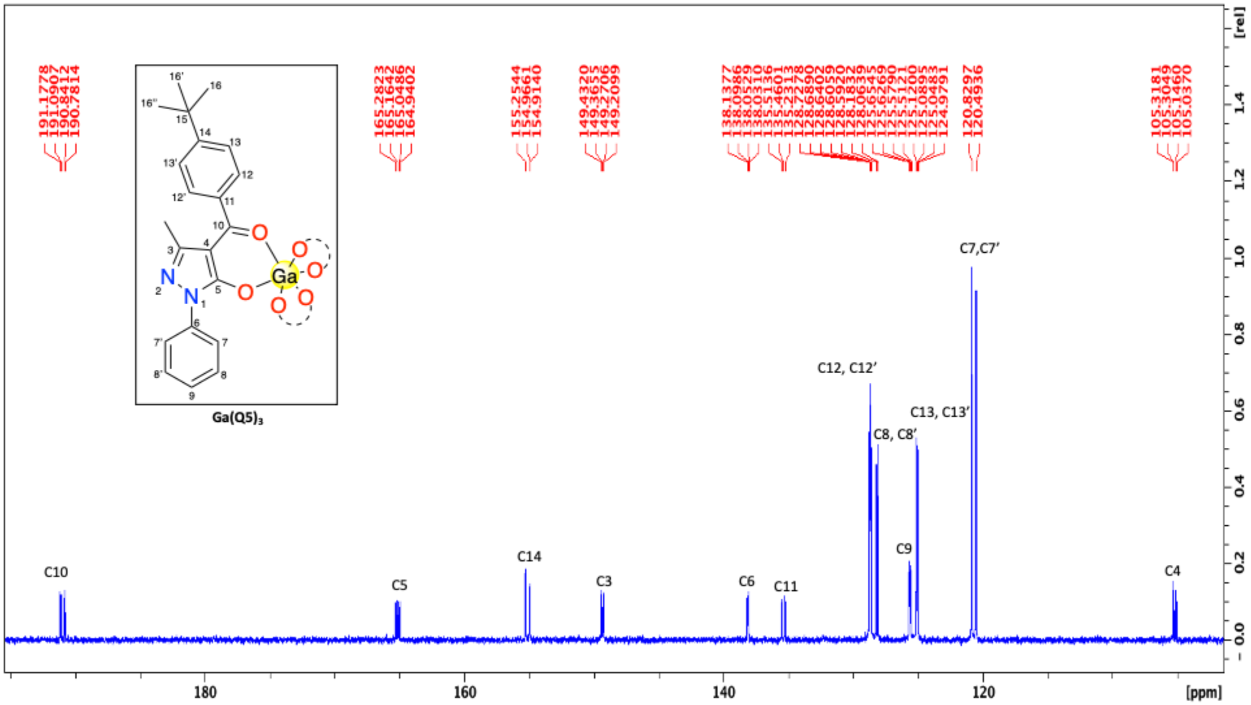

Figure S29. Magnification of ^{13}C NMR spectrum in $CDCl_3$ at 298 K of **Ga(Q5)3**.

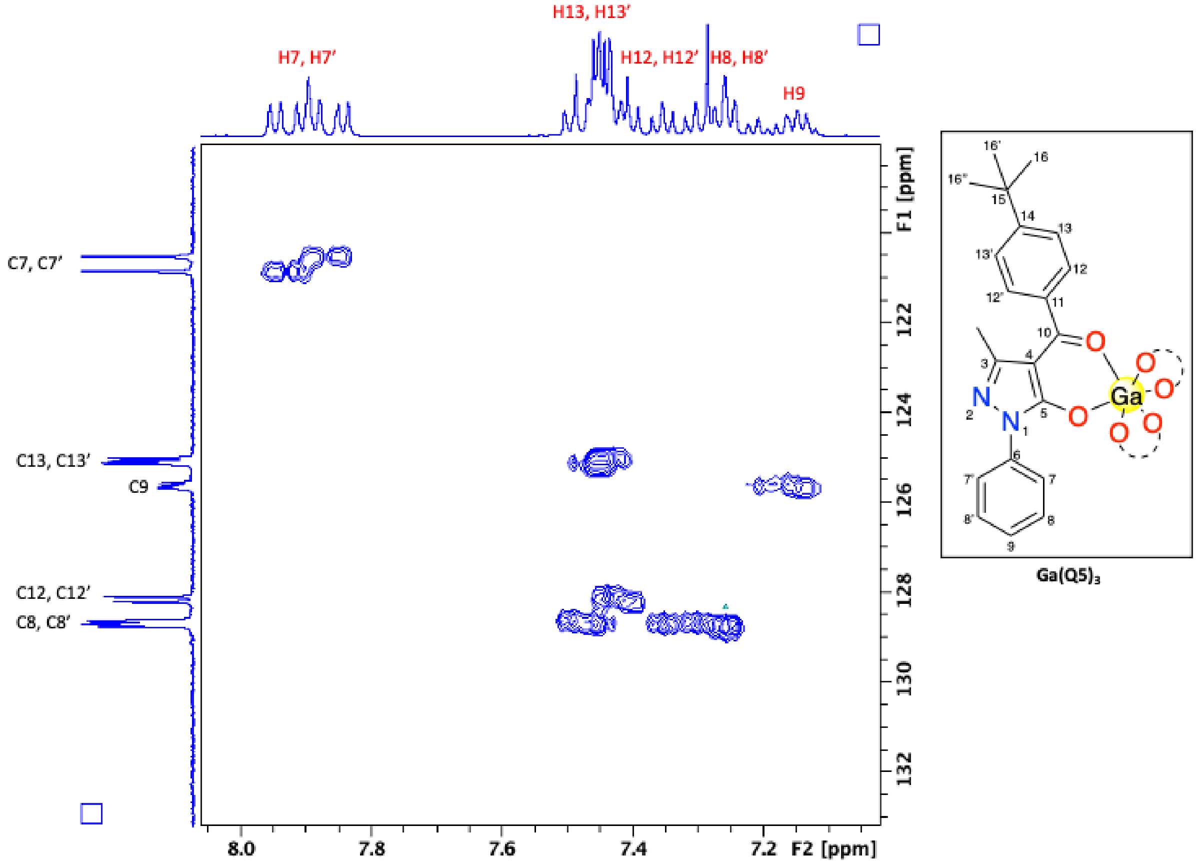

Figure S30. $\{^{1}H,^{13}C\}$-HSQC spectrum in $CDCl_3$ at 298 K of **Ga(Q5)3**.

Stability Studies

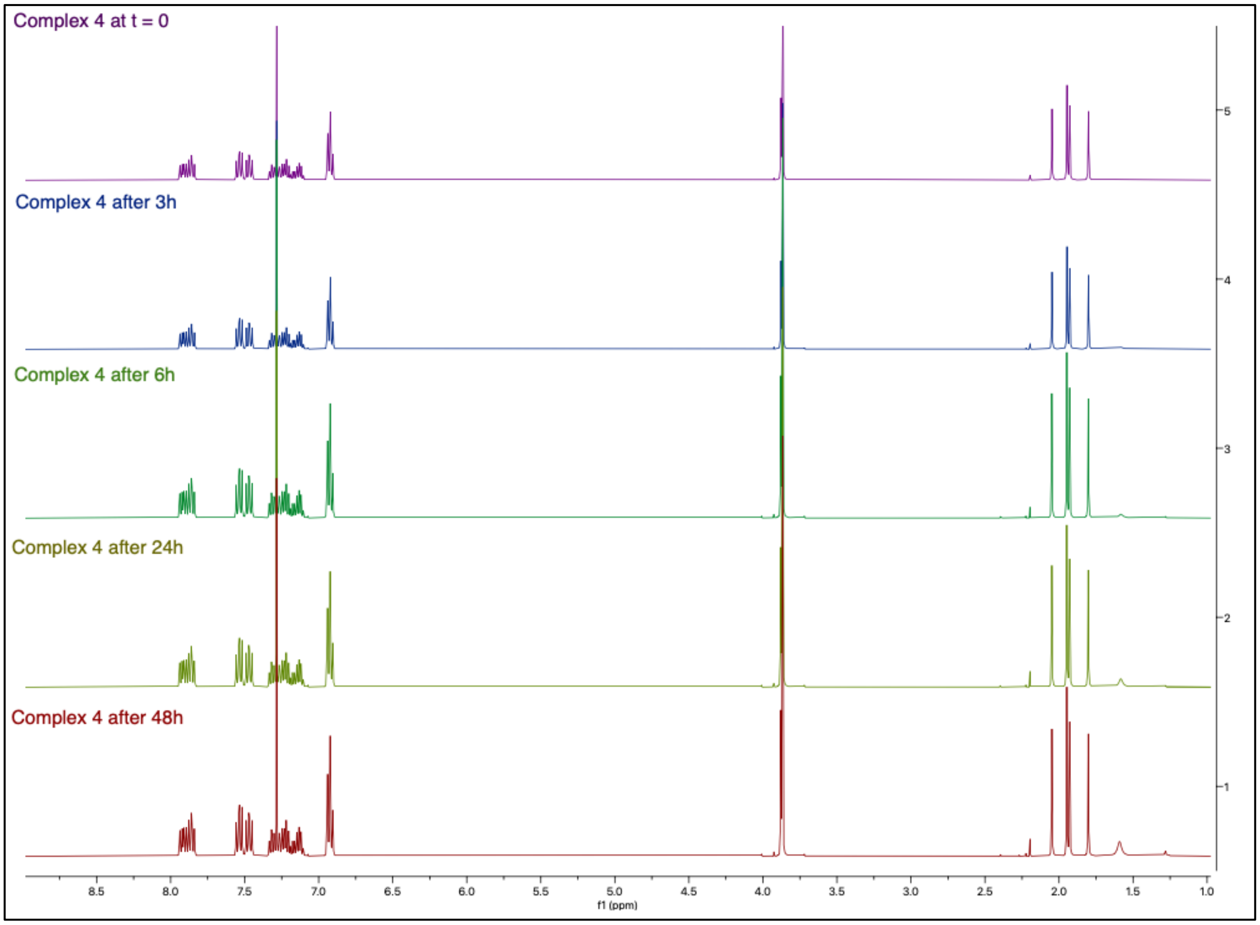

Figure S31. NMR spectroscopy stability studies of Ga(III) complexes. All the spectra were recorded in DMSO-d_6 solution over a period of 48 h. Representative of ^{1}H-NMR spectra for complex **4**.

DFT data

1

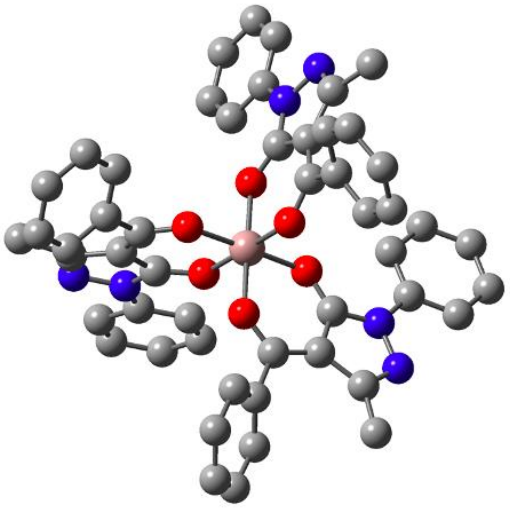

fac isomer

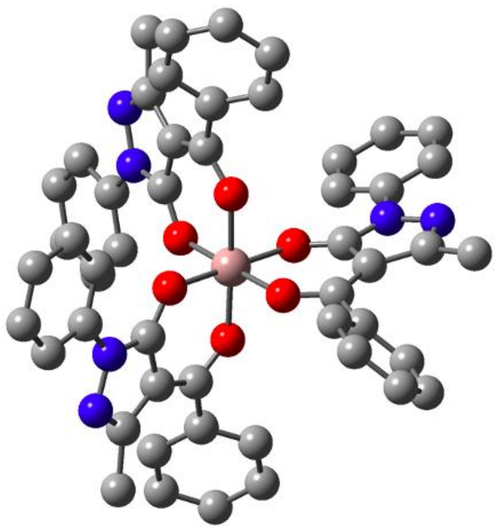

mer isomer

2

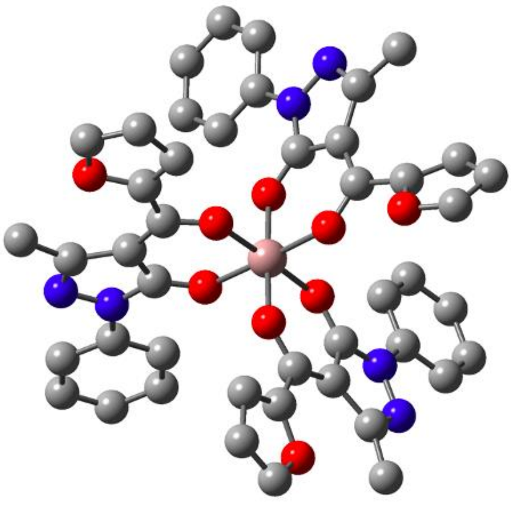

fac isomer

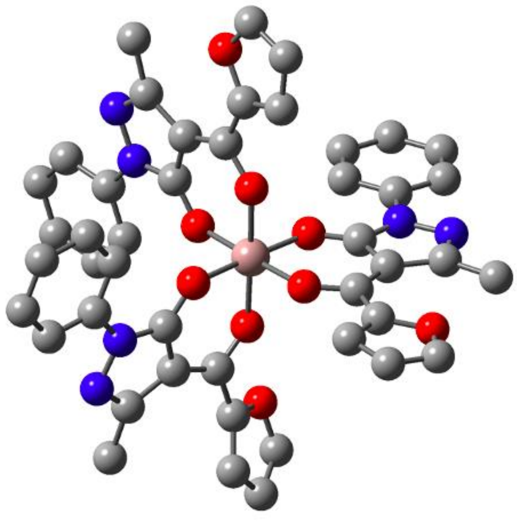

mer isomer

3

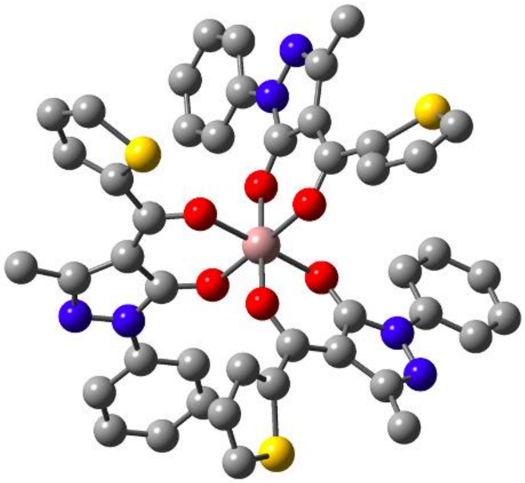

fac isomer

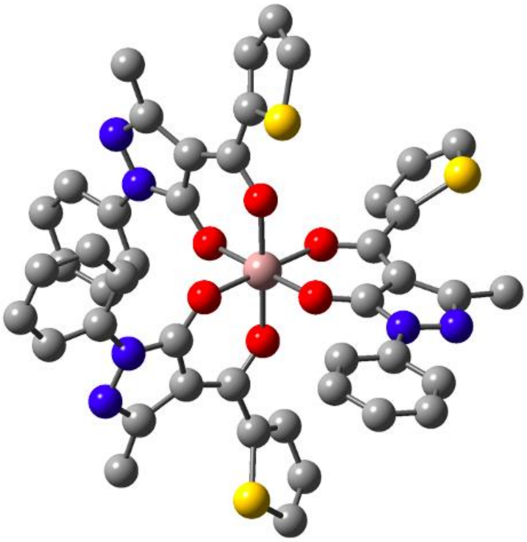

mer isomer

4

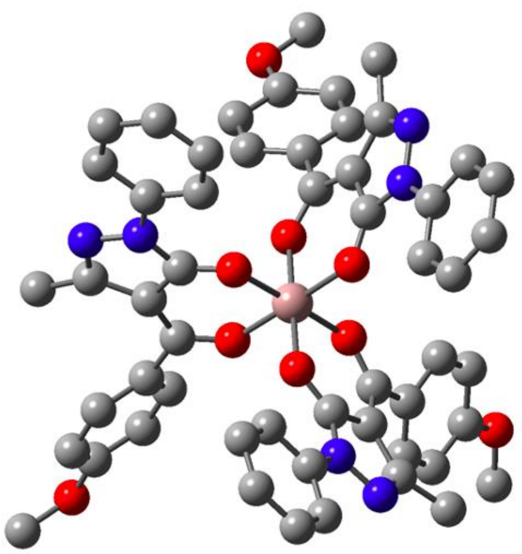

fac isomer

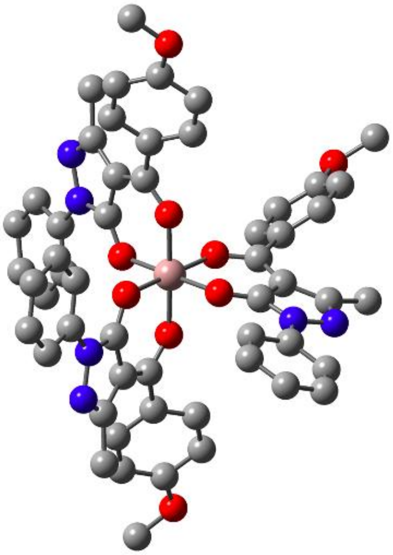

mer isomer

5

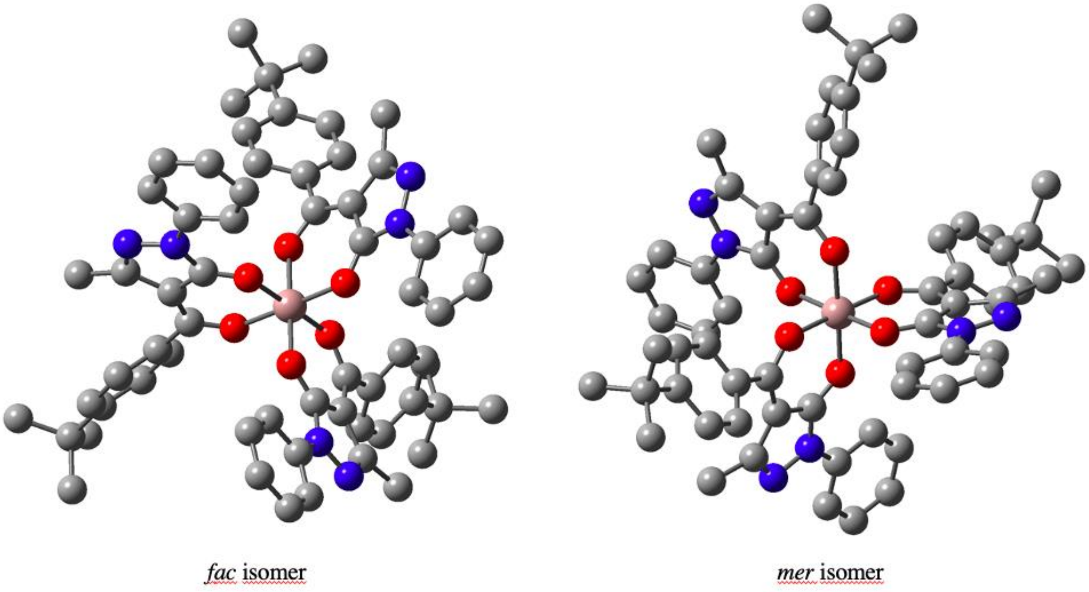

Figure S32. Optimized *fac* and *mer* isomers of complexes **1-5** (H atoms were omitted for clarity).

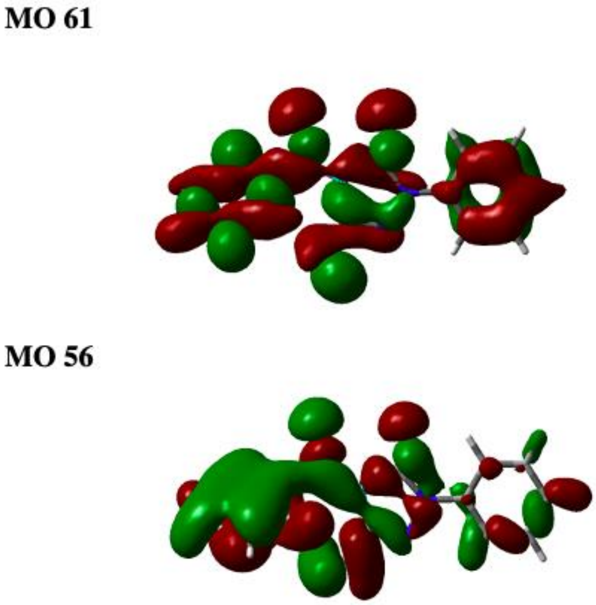

Figure S33. MOs of the Q_1^- anion showing the σ in-phase and σ out-of-phase combinations.

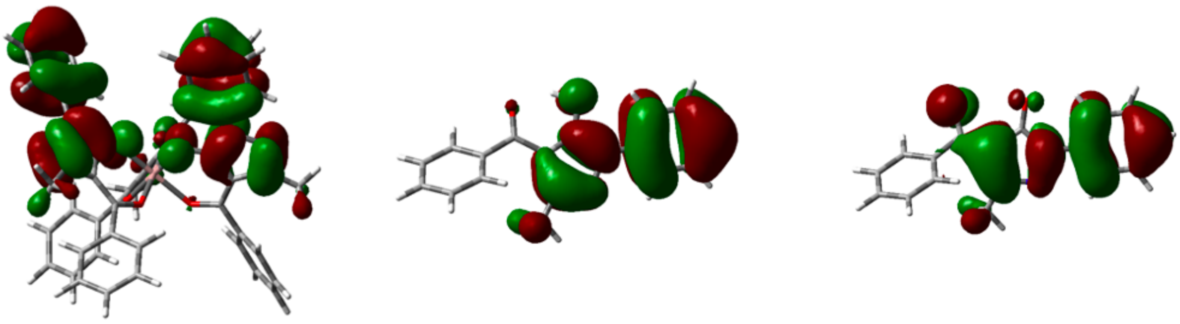

HOMO of *fac*-1 | **HOMO of HQ_1** | **HOMO-1 of Q_1^-**

Figure S34. Comparison of the HOMO of *fac*-**1** with the HOMO and HOMO-1 of the HQ_1 precursor and the Q_1^- ligand, respectively.

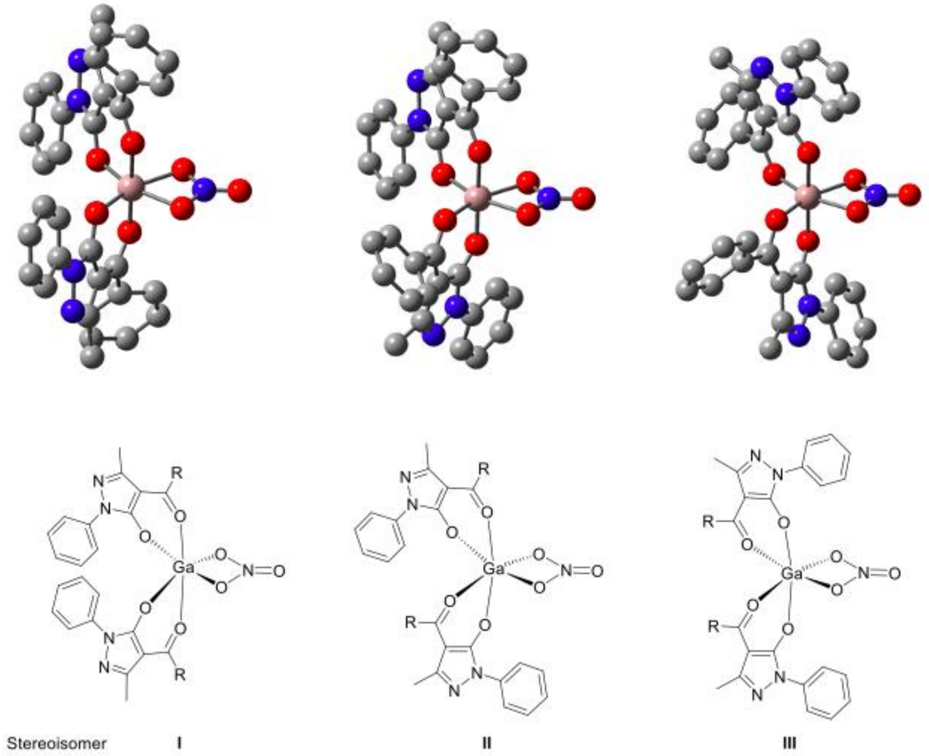

Figure S35. Optimized structures of the isomers **I**, **II** and **III** of $Ga(NO_3)(Q_1)_2$ intermediates (H atoms were omitted for clarity).

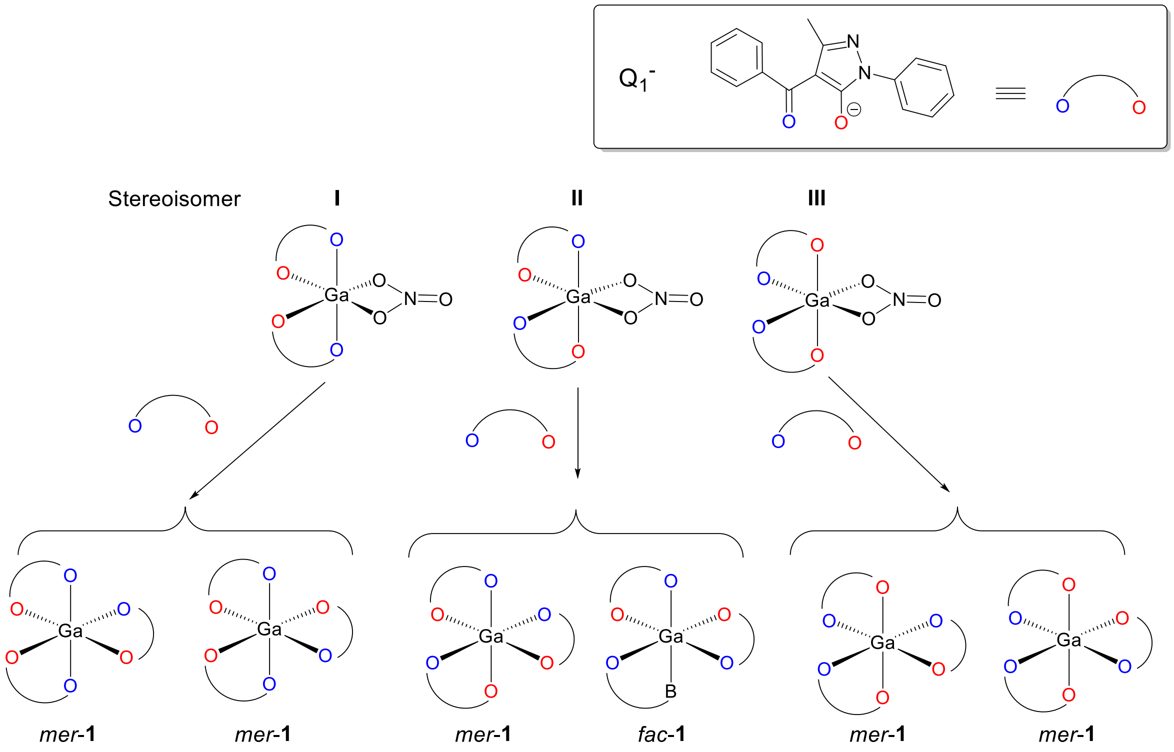

Figure S36. Distribution of *fac*- and mer- isomers of complex **1** upon substitution of nitrate from $Ga(NO_3)(Q_1)_2$ intermediates by Q_1^- anion.

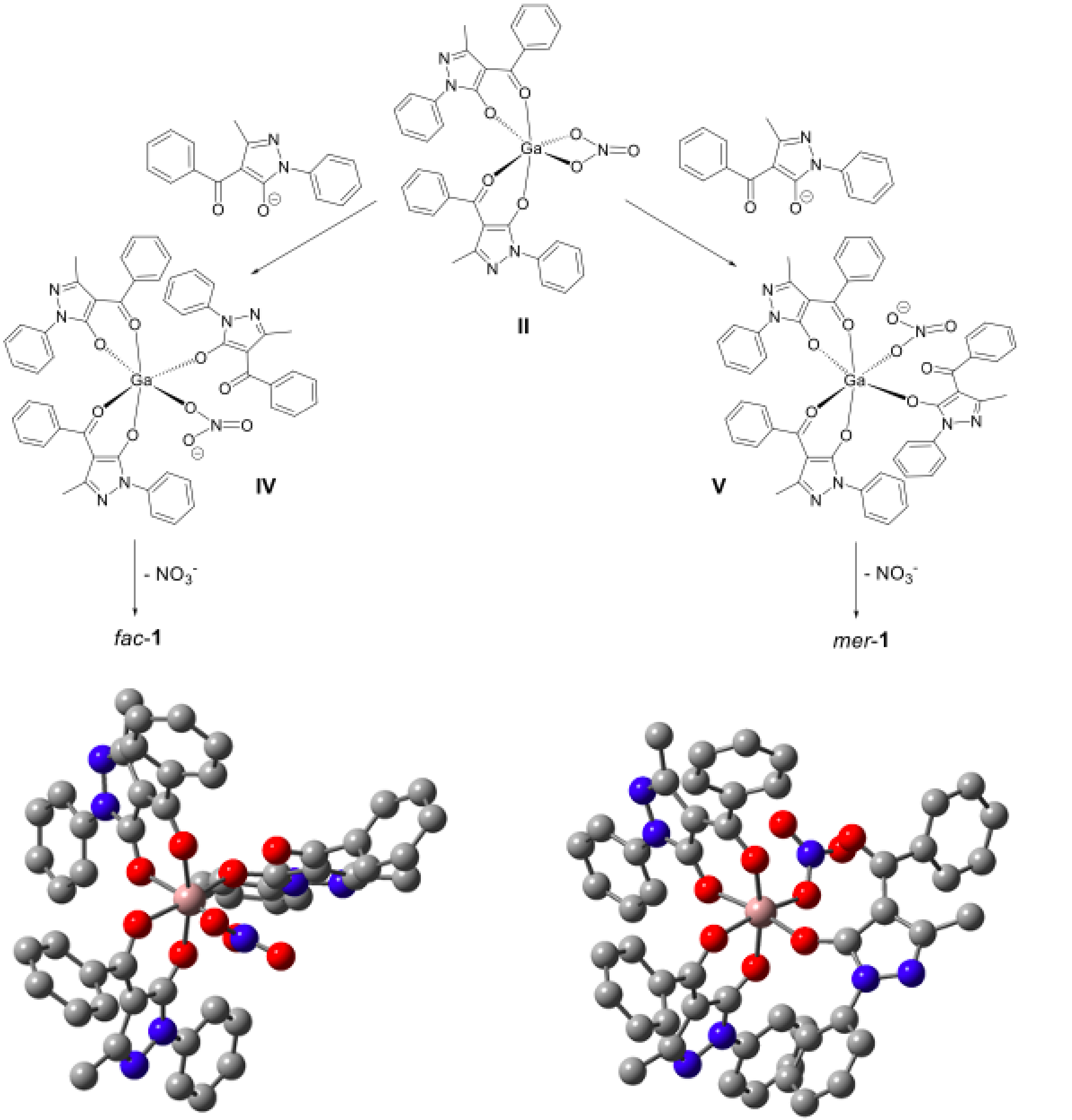

Figure S37. Formation of the *fac*- and *mer*- isomers of complex **1** from isomer **II** of $Ga(NO_3)(Q_1)_2$ (up) and optimized structures of the two isomers of $Ga(\kappa^1\text{-}NO_3)(Q_1)^{3-}$, **IV** and **V** (bottom).

Table S1. Comparison of selected computed and experimental structural parameters of compound **1**, *fac* and **2**, *mer*.

**Bond distances (Å) and angles (º)**	**1**, *fac*		**2**, *mer*	
	**X-ray**	**DFT**	**X-ray**	**DFT**
Ga-O (C=O)	1.969<br>1.993<br>2.006	1.958<br>1.959<br>1.963	1.958<br>1.964<br>1.997	1.942<br>1.944<br>1.951
Ga-O	1.939<br>1.946<br>1.951	1.942<br>1.944<br>1.950	1.920<br>1.947<br>1.949	1.941<br>1.941<br>1.956
C-O (C=O)	1.270<br>1.271<br>1.272	1.276<br>1.277<br>1.277	1.275<br>1.282<br>1.283	1.276<br>1.283<br>1.283
C-O	1.287<br>1.294<br>1.296	1.278<br>1.278<br>1.278	1.287<br>1.295<br>1.297	1.276<br>1.277<br>1.278
O-Ga-O (bite angle)	90.0<br>90.2<br>92.7	91.0<br>91.1<br>91.6	90.4<br>90.7<br>90.8	89.8<br>90.2<br>90.4

Table S2. Energies (Hartree) and relative energy differences (kcal/mol) for the isomers of complexes **1-5**.

	**1**		**2**	
	fac	*mer*	*fac*	*mer*
**E**	-4669.93160806	-4669.93072818	-4663.26313622	-4663.26250408
**E_o**	-4669.13160200	-4669.13089400	-4662.55311700	-4662.55269000
**E_t**	-4669.07751800	-4669.07673800	-4662.50190500	-4662.50142100
**H**	-4669.07657300	-4669.07579300	-4662.50096100	-4662.50047700
**G**	-4669.22860300	-4669.22873600	-4662.64742600	-4662.64743700
**ΔE**	0.0	0.6	0.0	0.4
**ΔG**	0.0	-0.1	0.0	0.0
	3		4	
	fac	*mer*	*fac*	*mer*
**E**	-5632.19190270	-5632.19239488	-5013.504424540	-5013.50376373
**E_o**	-5631.49274800	-5631.49328200	-5012.605923000	-5012.60549800
**E_t**	-5631.43977800	-5631.44026800	-5012.544017000	-5012.54353400
**H**	-5631.43883400	-5631.43932300	-5012.543073000	-5012.54259000
**G**	-5631.58925100	-5631.58988500	-5012.712985000	-5012.71361300
**ΔE**	0.0	-0.3	0.0	0.4
**ΔG**	0.0	-0.4	0.0	-0.4
	5			
	fac	*mer*		
**E**	-5141.69838425	-5141.69776741		
**E_o**	-5140.55929800	-5140.55861100		
**E_t**	-5140.48839500	-5140.48781900		
**H**	-5140.48745100	-5140.48687500		
**G**	-5140.67637000	-5140.67486900		
**ΔE**	0.0	0.4		
**ΔG**	0.0	0.9		

Table S3. Coordinates of optimized compounds.

fac-**1**

Ga	-0.14345100	-0.19733000	0.15098100
O	0.66870900	1.45341200	0.77954300
O	-0.93512000	-1.88344800	-0.45440000
O	-1.35930100	0.84664100	-0.94557000
O	1.05839100	-1.26183200	1.27999400
O	-1.42339300	-0.23476400	1.63249000
O	1.11931800	-0.21819800	-1.33489000
N	2.32810300	2.65830000	1.89608300
N	-3.32708500	2.05380400	-1.28817900
N	2.32244600	-2.59869300	-3.60107400
N	3.44279200	2.39750000	2.69017500
N	-4.47403100	2.39417500	-0.57379600
N	2.34565900	-1.35958800	-2.96457700
C	1.68433600	1.50306700	1.55387500
C	2.85576300	5.02200000	2.06376700
H	3.67399000	4.75624700	2.72020300
C	4.50507400	1.68029700	-3.02673400
H	4.53091200	2.67400900	-2.58769600
C	2.38455300	0.43989700	2.21768200
C	-3.29052600	-0.36654500	5.37875000
H	-2.77771400	-0.22853200	6.32665000
C	1.98731900	-0.90673300	2.08081800
C	-4.16276400	3.40258600	-3.12533500
H	-5.04218900	3.60619300	-2.52851400
C	3.48486500	1.10224000	2.88388900
C	-4.02105200	3.93754200	-4.40321000
H	-4.81073200	4.56866700	-4.80244900
C	-0.61758000	-2.65809500	-1.41763400
C	5.45908400	-0.00725700	-4.45286400
H	6.23584300	-0.34112600	-5.13587100
C	-2.50482600	1.25164800	-0.54927600
C	2.75111000	-1.96981500	4.24045700
H	2.44077400	-1.07893900	4.77743000
C	2.60169000	-2.02653500	2.84857300
C	-4.56789400	-0.93296800	5.34899300
H	-5.04979900	-1.23684900	6.27436200
C	-3.18461300	1.01131400	0.69181200
C	-2.66618000	0.01027600	4.19244900
H	-1.66353200	0.42601400	4.20102600
C	-2.61267600	0.22355000	1.71183700
C	-4.60661900	-0.71874600	2.94076900
H	-5.10109700	-0.88535200	1.98882000
C	1.26319300	-3.23607700	-3.16515400
C	3.44851600	0.82967400	-2.70148900
H	2.67161800	1.15251600	-2.02392300
C	0.53646400	-2.44040700	-2.20095700
C	-1.55692500	-3.79189800	-1.64105300
C	0.98007600	4.33075200	0.68329400
H	0.35579400	3.54988100	0.27468400
C	1.31342300	-1.24051700	-2.07729400
C	-5.49016900	2.05024700	1.58227800
H	-6.09354900	2.88785200	1.22316600
H	-5.08049700	2.30090700	2.56597000
H	-6.14895300	1.18665200	1.71741500
C	-2.96145400	-5.52864600	-0.70083700
H	-3.31948200	-6.08263700	0.16258800
C	-1.88281000	2.85792200	-4.63197800
H	-0.98912800	2.64090200	-5.21104900
C	-2.04396800	-4.49539700	-0.52831200
H	-1.69205600	-4.22420100	0.46202300
C	1.04083400	-4.63943800	-3.64601600
H	0.81450100	-5.31571600	-2.81538600
H	1.94890200	-4.98223500	-4.14859300
H	0.21002400	-4.71111200	-4.35500700
C	-2.04059000	-4.10323600	-2.91893000

H	-1.70765700	-3.52326600	-3.77394500
C	-2.88260900	3.67068800	-5.16572600
H	-2.77656000	4.09092200	-6.16199200
C	4.64981700	0.54740900	3.64840300
H	4.36793300	0.19749800	4.64637400
H	5.39658100	1.33743400	3.76135700
H	5.10399100	-0.30011400	3.12503600
C	3.46546700	-4.29110800	2.86221600
H	3.74387900	-5.19332400	2.32466200
C	-3.15210500	2.58685700	-2.59587900
C	3.25495500	-3.06776100	4.93794300
H	3.35357100	-3.02118300	6.01899300
C	-2.00422100	2.31151000	-3.35430300
H	-1.22292000	1.68583800	-2.94813600
C	3.62461500	-4.22417600	4.24925900
H	4.02629200	-5.07544000	4.79228500
C	2.04415000	4.00615400	1.53796400
C	4.41374100	-0.87290900	-4.14089600
H	4.36378200	-1.86599500	-4.56804100
C	-4.40062700	1.78818300	0.58553700
C	2.60046700	6.35066400	1.73400300
H	3.23623700	7.12932600	2.14727700
C	-3.33164000	-0.13883000	2.96624700
C	-5.21871900	-1.11997400	4.12842800
H	-6.20147600	-1.58246800	4.09905900
C	-2.97882200	-5.12181700	-3.08540000
H	-3.36118800	-5.34631200	-4.07727500
C	5.51407500	1.27312300	-3.89927900
H	6.33204700	1.94440000	-4.14554200
C	3.40431700	-0.45599000	-3.26119300
C	1.54373400	6.68437600	0.88499700
H	1.34887500	7.72257500	0.63094000
C	-3.43042300	-5.84383500	-1.97936200
H	-4.15471000	-6.64322700	-2.11110500
C	0.74169900	5.66809100	0.36640800
H	-0.08399400	5.90924800	-0.29765900
C	2.93807000	-3.20609300	2.16683900
H	2.78507200	-3.25638200	1.09342900

mer-**1**

Ga	-0.11639700	-0.06768800	-0.13556300
O	-1.76351100	0.44942600	0.77188900
O	-1.08307800	-1.61606100	-0.84466000
O	0.88183800	1.45487700	0.54771300
O	-0.57459500	1.01728300	-1.68995500
O	0.30574400	-1.11505400	1.46612300
O	1.51209800	-0.65212900	-1.04305400
N	-3.89787100	1.39561700	0.79732500
N	2.36670400	2.45945200	2.04444200
N	2.71843600	-3.63798100	-2.41112100
N	-4.71758900	2.12856000	-0.05830200
N	3.03588600	2.12856600	3.22123000
N	2.85909100	-2.32983200	-1.95410500
C	-2.68123400	1.14911100	0.22545700
C	-5.74202600	1.32008300	2.37456800
H	-6.32855500	1.88483100	1.66180400
C	5.64906400	0.10737400	-1.53127200
H	5.78979600	1.14997100	-1.25916700
C	-2.68939000	1.82206400	-1.04286500
C	1.03902000	-4.18488200	4.20386000
H	1.19914000	-5.22337400	3.92752300
C	-1.58314900	1.76939000	-1.91626100
C	3.50917900	4.60094400	2.08873600
H	3.97249200	4.29459300	3.01745400
C	-4.01883200	2.38566200	-1.13638200
C	3.77876300	5.84645100	1.52729100

H	4.46622800	6.51745400	2.03565600
C	-0.68849800	-2.61883200	-1.52983500
C	6.54514600	-2.02557200	-2.19491400
H	7.39097400	-2.66085500	-2.44456300
C	1.56259000	1.43600300	1.62861000
C	-1.75880300	3.95459300	-3.16660500
H	-2.09044900	4.43848400	-2.25344400
C	-1.47812800	2.58147100	-3.16009400
C	0.74062900	-3.84988800	5.52770200
H	0.66753900	-4.62840500	6.28230800
C	1.66703600	0.41100200	2.62845100
C	1.11890400	-3.18998900	3.23292400
H	1.32224300	-3.44061500	2.19662300
C	0.97296400	-0.81077300	2.51236000
C	0.62913900	-1.51453500	4.91183400
H	0.43721800	-0.47943600	5.17647700
C	1.44294700	-3.93402100	-2.34760700
C	4.35425800	-0.41057500	-1.56001500
H	3.50771400	0.21415900	-1.31466900
C	0.67617000	-2.82351600	-1.82766000
C	-1.77597200	-3.53192300	-1.97628500
C	-3.64366600	0.24985300	2.96590000
H	-2.62601400	-0.01532800	2.72021400
C	1.65990500	-1.81743900	-1.54520500
C	3.26654600	0.30484600	4.77726600
H	4.13824600	0.90078600	5.05932700
H	3.58715000	-0.72134400	4.57146900
H	2.58718300	0.26579100	5.63457300
C	-3.87820900	-4.63313200	-1.48253200
H	-4.67170000	-4.87028800	-0.77936500
C	2.29893100	5.35548600	-0.30499500
H	1.82315700	5.63889700	-1.24019800
C	-2.81767600	-3.82508800	-1.08163300
H	-2.78055900	-3.41408100	-0.07792300
C	1.02704200	-5.32735500	-2.71726200
H	0.33073500	-5.74491400	-1.98321400
H	1.92010700	-5.95605400	-2.76118400
H	0.53242500	-5.37191800	-3.69288100
C	-1.84030300	-4.01655300	-3.28983400
H	-1.06709000	-3.74351800	-4.00086300
C	3.17760700	6.23394000	0.32843100
H	3.39204700	7.20666700	-0.10543600
C	-4.70658500	3.10290100	-2.26010500
H	-4.37111400	4.13954900	-2.36334400
H	-5.78085500	3.11090900	-2.05887700
H	-4.52670400	2.60781200	-3.21965900
C	-0.80870600	2.72228100	-5.48552700
H	-0.43599200	2.24254600	-6.38627100
C	2.62432400	3.72342300	1.44497000
C	-1.56791400	4.70498000	-4.32685300
H	-1.77373900	5.77178500	-4.31909600
C	2.01448800	4.10347900	0.24007400
H	1.33480100	3.42885200	-0.25921500
C	-1.10567000	4.08813700	-5.49055500
H	-0.96476800	4.67188500	-6.39621600
C	-4.41979500	0.97969100	2.05312400
C	5.26094600	-2.56336900	-2.22791100
H	5.09462700	-3.59762600	-2.49970300
C	2.63245300	0.93245600	3.57161900
C	-6.27889500	0.92967900	3.59871500
H	-7.30459700	1.19974700	3.83598000
C	0.94107100	-1.84348500	3.58596500
C	0.52335800	-2.51605400	5.87714100
H	0.26675000	-2.25444700	6.90001200
C	-2.91605100	-4.80624300	-3.69524500
H	-2.96622000	-5.16515400	-4.71954600
C	6.74966500	-0.68949500	-1.84577100
H	7.75369300	-0.27518800	-1.82020400
C	4.16028600	-1.75561400	-1.90771600
C	-5.51546400	0.20160500	4.51295700
H	-5.93938200	-0.10062400	5.46658400

C	-3.92937500	-5.12552600	-2.78975300
H	-4.76352300	-5.74682100	-3.10455000
C	-4.20134500	-0.13194800	4.18593800
H	-3.59198600	-0.69909000	4.88484200
C	-0.97437300	1.97611400	-4.32189400
H	-0.71750000	0.92199800	-4.29738800

fac-**2**

Ga	-0.03340000	-0.02875000	-0.45905300
O	0.92468200	-1.28877600	0.65929400
O	-3.14544600	-3.99819900	-2.43921700
O	-0.94572600	1.26572400	-1.61218700
O	-1.51802500	-0.08883500	0.78329300
O	4.64867900	-0.70173600	-3.10620000
O	-0.85982900	-1.44280900	-1.51539600
O	-2.32069300	2.41678000	-3.57459300
O	0.74036700	1.42066600	0.58785900
O	1.44310500	-0.03643900	-1.72730900
N	-3.65931600	-0.70590000	1.44908500
N	1.34100400	3.65278600	0.91073400
N	-4.67201300	-1.56273200	1.03655300
N	2.66103900	-2.75357600	1.15279100
N	1.07420900	4.89160400	0.33757000
N	3.87611000	-3.14956900	0.60786900
C	2.05165200	-1.81520100	0.37014300
C	3.48904200	2.34695900	3.66634200
H	3.70493800	1.40172100	4.15712200
C	4.14951200	0.69481100	-4.78454300
H	4.24177800	1.27904100	-5.68919600
C	-2.90051100	-1.76699700	-0.38118300
C	4.12820500	3.51459900	4.08245800
H	4.84539800	3.48846000	4.89813900
C	-1.96790000	-2.07712100	-1.40168300
C	-5.19135300	-3.16676800	-0.65934800
H	-4.81574400	-4.19240900	-0.60700000
H	-5.36624100	-2.93900100	-1.71505800
H	-6.14015000	-3.10854400	-0.12008500
C	2.23303000	-3.34743200	2.37345200
C	5.30978400	-2.74827900	-1.26261100
H	5.10025800	-3.06800800	-2.28765600
H	5.84789100	-3.54542800	-0.74363900
H	5.95319500	-1.86611900	-1.32387300
C	-2.58020900	-0.78000100	0.61544900
C	3.83413400	4.71744500	3.43786100
H	4.32184000	5.63763200	3.74895100
C	3.37516400	-0.24471900	-2.89994500
C	2.66053900	-4.92721100	4.15386600
H	3.30140100	-5.68495700	4.59720200
C	-4.24144500	-2.19304100	-0.02901100
C	-1.80228700	-4.49963400	-4.15997400
H	-1.40382100	-4.99420000	-5.03453900
C	2.57377100	-0.64317400	-1.74593800
C	-4.38224700	1.66258600	4.87333200
H	-4.57738500	2.26712300	5.75475800
C	2.93384200	-1.57520900	-0.74257000
C	-5.37050000	0.82099100	4.35962000
H	-6.34415600	0.76474500	4.83948000
C	-2.10336400	-3.11099600	-2.42378400
C	-2.94816400	-4.82815200	-3.49261100
H	-3.69793700	-5.59248200	-3.63202000
C	-3.87510600	0.10379900	2.59914000
C	0.66101000	-3.58980500	4.19081800
H	-0.27189800	-3.29426400	4.66343600
C	0.64871200	2.65690400	0.28036400
C	-2.87440400	0.94509400	3.10890200
H	-1.90895000	0.99358000	2.62732500

C	0.19144800	4.69614500	-0.61179700
C	2.56554300	2.36501900	2.62108000
H	2.07657400	1.45546800	2.30428800
C	3.04872600	-4.32724400	2.95878200
H	3.97367000	-4.60345500	2.46978600
C	5.10060900	-0.12519100	-4.24674300
H	6.10497100	-0.39264600	-4.53920600
C	-0.99550100	2.54020300	-1.57051700
C	-5.12651800	0.04366600	3.23041900
H	-5.88777000	-0.61195300	2.82839700
C	-1.25075400	-3.38770800	-3.46569500
H	-0.34471600	-2.84365500	-3.68500100
C	-2.83475800	4.22637100	-2.36102700
H	-2.85545900	4.94736700	-1.55883800
C	1.03075900	-2.97578400	2.99406200
H	0.40067300	-2.22125100	2.54705600
C	-3.14046600	1.71576000	4.24087600
H	-2.35823200	2.36334600	4.62803800
C	-0.16017300	3.29814100	-0.71739200
C	-3.70760600	4.17191400	-3.48567600
H	-4.50261900	4.86086300	-3.73351700
C	4.05154400	-2.46760400	-0.49728400
C	-3.34155300	3.05833000	-4.18608200
H	-3.69294200	2.61077200	-5.10379200
C	-1.99620900	3.14286100	-2.45651500
C	-0.22215600	5.88563000	-1.42735500
H	-1.18729500	6.29302400	-1.10525500
H	-0.30639000	5.64059400	-2.48994400
H	0.52658800	6.67134000	-1.29831500
C	2.27840800	3.57892200	1.97898700
C	2.91568200	4.75807800	2.39190200
H	2.68247900	5.68746100	1.88894700
C	1.46608500	-4.56488800	4.77895700
H	1.16774200	-5.03605300	5.71138900
C	3.02973700	0.62043600	-3.91084200
H	2.08106400	1.12880700	-3.99344400

mer-**2**

Ga	0.17240400	-0.27784800	0.10121100
O	1.93345000	0.48615000	-0.18762900
O	-0.35067200	2.55393500	4.68911200
O	0.18652900	-0.90716500	-1.74558800
O	-0.60908400	1.39493400	-0.49722000
O	3.47395100	-4.35019900	1.45781000
O	0.10501700	0.35395100	1.93863300
O	0.80950000	-1.25416700	-4.28923600
O	-1.57508400	-1.10988800	0.38727700
O	0.98375300	-1.92470000	0.73290500
N	-1.71010500	3.41902100	-0.17390800
N	-3.59428900	-1.98488700	-0.39262200
N	-2.07615100	4.24845800	0.87908400
N	4.25650100	0.46730000	-0.13236700
N	-4.13626500	-2.43190200	-1.59293600
N	5.28314700	-0.40334800	0.21063600
C	3.03392900	-0.11183100	0.05599500
C	-4.80985400	-1.47387500	3.09650800
H	-4.44420600	-1.08038200	4.04143300
C	1.83723800	-5.75720900	2.05653200
H	1.32978000	-6.64854500	2.39748900
C	-0.96764700	2.43956200	1.70797000
C	-6.10761600	-1.97579200	3.00335000
H	-6.76094400	-1.97789400	3.87144100
C	-0.32890500	1.43540300	2.47534200
C	-1.94931900	4.41575900	3.26184000
H	-1.03890100	4.75659200	3.76258400
H	-2.49053200	3.78074000	3.96953600

H	-2.56675900	5.28394600	3.01818800
C	4.59260300	1.76731800	-0.60411400
C	5.67006800	-2.61337000	1.03379600
H	5.48436500	-2.92887400	2.06467700
H	6.68949700	-2.22686000	0.95810200
H	5.57859200	-3.50179600	0.40256100
C	-1.05342700	2.30972900	0.27678400
C	-6.55674500	-2.47385300	1.77901200
H	-7.56540000	-2.86799100	1.68641600
C	2.28542700	-3.68627300	1.31840800
C	6.31397300	3.37172100	-1.16371400
H	7.36886200	3.62335100	-1.23686300
C	-1.64481700	3.69238200	1.98449600
C	0.55045100	0.98947800	6.01485800
H	0.93173700	0.49308300	6.89600700
C	2.20387500	-2.30718400	0.84460400
C	-2.77899400	4.63870300	-4.07591200
H	-3.05774600	4.96122400	-5.07529600
C	3.28452100	-1.44454600	0.54373300
C	-3.12742200	5.40522300	-2.96244300
H	-3.68142100	6.33179000	-3.08849500
C	-0.07157200	1.46272500	3.91196500
C	0.03023700	2.25133800	5.95437000
H	-0.12856300	3.02714000	6.68833700
C	-2.05821900	3.80414700	-1.49857700
C	3.99560900	3.95912300	-1.42360500
H	3.22535900	4.67341400	-1.70216200
C	-2.29372000	-1.59222300	-0.55192200
C	-1.70339300	3.02652600	-2.61121300
H	-1.15277400	2.10749100	-2.47620400
C	-3.18590800	-2.36779200	-2.49359600
C	-3.96008700	-1.46356800	1.99042800
H	-2.95657500	-1.07239500	2.07053800
C	5.94965800	2.10858900	-0.70473700
H	6.69817200	1.37991800	-0.42248400
C	3.18530300	-5.59760600	1.90383800
H	4.03460200	-6.24553500	2.06101900
C	-0.66819400	-1.56985600	-2.42305000
C	-2.77277200	4.99786000	-1.67908500
H	-3.03926900	5.58775500	-0.81200000
C	0.48807100	0.47712900	4.68970600
H	0.80747900	-0.48890500	4.32939400
C	-0.39456500	-3.12745800	-4.51317800
H	-1.07998200	-3.93360500	-4.30414900
C	3.60838400	2.69956400	-0.96601100
H	2.56241400	2.44145500	-0.89228200
C	-2.06825600	3.45378100	-3.88793500
H	-1.78733800	2.84498500	-4.74338800
C	-1.95890800	-1.86064700	-1.92255700
C	0.51262500	-3.03818400	-5.60747900
H	0.63491200	-3.74228000	-6.41830000
C	4.73225800	-1.52490900	0.60487200
C	1.20876300	-1.87806000	-5.41890500
H	1.98363300	-1.37803600	-5.98061300
C	-0.18917400	-2.01381800	-3.73509000
C	-3.54281000	-2.74022800	-3.90263200
H	-3.23739400	-3.76337600	-4.15055800
H	-3.07641700	-2.07043900	-4.63045700
H	-4.62905000	-2.68616100	-4.01063800
C	-4.42077100	-1.96719700	0.76472800
C	-5.72475400	-2.47387800	0.66246200
H	-6.06778800	-2.85690700	-0.28971900
C	5.34239000	4.30606200	-1.52657100
H	5.63146700	5.29036900	-1.88443100
C	1.25101800	-4.51867600	1.67509400
H	0.20558100	-4.24981300	1.65884900

fac-**3**

Ga	-0.01402700	-0.02458600	-0.28005300
O	-0.60043400	1.41255800	0.88547900
O	0.54833900	-1.47540900	-1.47367500
O	1.51214000	-0.31185000	0.88969800
O	1.03670800	1.20715700	-1.37745500
O	-1.03105600	-1.27233700	0.80511700
O	-1.53122600	0.28578900	-1.47208300
N	3.80341800	-0.30587700	1.32052100
N	-2.08307200	-3.32319400	1.16230400
N	4.96027300	0.24273400	0.77707600
N	-1.77905300	3.38351000	1.28158200
N	-2.11781700	-4.58652300	0.58165000
N	-2.79430000	4.12363200	0.68557700
C	-1.51887400	2.24468900	0.57461700
C	-3.76005400	-1.60611400	4.01755400
H	-3.72830900	-0.64743200	4.52837700
C	-4.52632300	-0.04896800	-4.32930500
H	-4.55443900	-0.52850700	-5.30142100
C	3.15504800	1.00983200	-0.37773400
C	-4.64100800	-2.59893500	4.44563700
H	-5.30108700	-2.42188300	5.29021900
C	2.24699500	1.61146100	-1.28034500
C	5.67904000	1.66812600	-1.01877000
H	5.80770700	2.72563400	-0.76832200
H	5.47472600	1.60698600	-2.09223800
H	6.61927800	1.15315700	-0.80535600
C	-1.14941500	3.90007100	2.44885200
C	-4.26490500	4.07804100	-1.21233400
H	-4.03767400	3.96069700	-2.27649600
H	-4.30376100	5.14374000	-0.97242900
H	-5.25970000	3.65598400	-1.04010500
C	2.69662200	0.08653400	0.62295000
C	-4.66309200	-3.82333000	3.77542800
H	-5.34242300	-4.60922400	4.09513300
C	-3.54923100	0.86534500	-2.40937000
C	-0.99397300	5.66536400	4.09387600
H	-1.34060200	6.62335500	4.47264800
C	4.59274800	1.01955500	-0.21266600
C	2.29711500	4.16689700	-4.01457300
H	1.92186900	4.49617300	-4.97714800
C	-2.48850400	1.13431500	-1.42175400
C	4.23099900	-2.91134700	4.61855800
H	4.35032200	-3.57818800	5.46797100
C	-2.48020900	2.20182400	-0.49348700
C	5.34689800	-2.33122700	4.01272000
H	6.34464400	-2.54342300	4.38800200
C	2.55891300	2.73268700	-2.18475300
C	3.17551900	4.89723500	-3.25465800
H	3.60503700	5.86607600	-3.47360400
C	3.91985100	-1.19036900	2.42901900
C	0.45704500	3.75202900	4.24556100
H	1.25250200	3.20429900	4.74385800
C	-1.21783000	-2.49897400	0.50040100
C	2.79157600	-1.76735800	3.03129800
H	1.80402000	-1.54988400	2.65161700
C	-1.24910900	-4.58515600	-0.40013600
C	-2.90610500	-1.81871900	2.93531000
H	-2.22861300	-1.04274400	2.61003400
C	-1.58545400	5.13533900	2.95011300
H	-2.38023100	5.66116900	2.43747800
C	-5.64915100	0.35107000	-3.64995600
H	-6.68407600	0.25291200	-3.95059700
C	0.34543700	-2.73874000	-1.41192400
C	5.20081000	-1.47464900	2.92458000
H	6.06144000	-1.02016800	2.45151000
C	1.92828300	2.94645600	-3.39335000
H	1.22083800	2.23427800	-3.80059100
C	1.72251100	-4.78447700	-2.18717400
H	1.55971900	-5.40205500	-1.31382500
C	-0.12165200	3.20246800	3.10144400
H	0.21653200	2.25054600	2.71897500

C	2.96081500	-2.62249400	4.12030300
H	2.08078000	-3.06389000	4.58054200
C	-0.60136100	-3.29753500	-0.52154000
C	2.57607200	-5.14872600	-3.26242200
H	3.10169200	-6.09517600	-3.32324700
C	-3.21669500	3.43962700	-0.34958900
C	2.66985400	-4.16301500	-4.21205900
H	3.23688700	-4.18320400	-5.13372000
C	1.16842800	-3.53068400	-2.33245500
C	-1.15268700	-5.82719300	-1.23708500
H	-0.30198500	-6.45657200	-0.95255900
H	-1.04732500	-5.59187800	-2.29991700
H	-2.06197700	-6.41501800	-1.08799000
C	-2.93769600	-3.05225900	2.26762200
C	-3.81969300	-4.05659000	2.69219100
H	-3.83098200	-5.00384900	2.16905300
C	0.03019200	4.98020000	4.74985500
H	0.48847000	5.39794000	5.64197700
C	-3.33381400	0.22230800	-3.61158300
H	-2.33801100	-0.04338900	-3.94516000
S	3.58090600	4.09245500	-1.78230700
S	1.74902200	-2.76394200	-3.79649600
S	-5.26217400	1.08466100	-2.13631200

mer-**3**

Ga	0.15913600	0.17821600	-0.12659800
O	1.74439800	-0.42944500	0.81872000
O	-0.01095100	1.63846900	1.15295300
O	-0.90404400	-0.94786300	1.05461100
O	0.29751000	-1.28162500	-1.41637700
O	-1.42120800	0.80376400	-1.09215900
O	1.24857300	1.29330300	-1.29709000
N	-2.39546400	-2.70774700	1.39946800
N	-3.44559700	1.96335500	-1.17057500
N	-2.82703900	-3.84798700	0.73058800
N	4.03887100	-0.81245900	0.94328400
N	-4.11455100	2.93366500	-0.43225000
N	5.19387200	-0.42496600	0.27303400
C	2.93484100	-0.20768600	0.41343300
C	-4.20712100	-0.18012100	-4.12676700
H	-3.75541900	-0.99646900	-4.68446200
C	2.61199800	4.16932200	-3.95574100
H	2.15864300	4.62075500	-4.83119000
C	-1.16860200	-2.85795500	-0.47358400
C	-5.44818500	0.32652600	-4.51137400
H	-5.97050700	-0.08810200	-5.36906600
C	-0.20020200	-2.46113100	-1.42608700
C	-2.45192500	-5.06705200	-1.30532900
H	-1.74452300	-5.89928500	-1.23830900
H	-2.46290700	-4.72682000	-2.34543300
H	-3.44392900	-5.44507200	-1.04510000
C	4.14354300	-1.76981100	1.99117300
C	5.91546800	0.96767100	-1.54465000
H	5.57480000	1.00530300	-2.58400700
H	6.77277700	0.29309500	-1.47563000
H	6.25343000	1.97240400	-1.27262600
C	-1.43631000	-2.04931900	0.68422300
C	-6.01125500	1.37254600	-3.77824000
H	-6.97812900	1.77995900	-4.06181800
C	2.88734600	2.59394900	-2.24692700
C	5.54924600	-3.20886000	3.33287000
H	6.53799000	-3.59359400	3.56894900
C	-2.11146900	-3.95055700	-0.36268300
C	1.44193100	-3.81296400	-4.50415500
H	1.87254900	-3.61987500	-5.48037500
C	2.51342100	1.48620300	-1.34936800

C	-4.26315700	-1.76534200	5.06963000
H	-4.74776400	-1.53184000	6.01359000
C	3.41299500	0.69002100	-0.60308000
C	-4.67760400	-2.86406400	4.31478300
H	-5.48991600	-3.49415300	4.66747000
C	0.34392600	-3.31028400	-2.49975100
C	1.42429000	-5.05272400	-3.91636900
H	1.81986500	-5.98261500	-4.30360900
C	-3.01169900	-2.36510900	2.63512300
C	3.17724300	-3.13868400	3.72981000
H	2.29906400	-3.46862800	4.27849100
C	-2.22068200	1.68713300	-0.62983700
C	-2.58650200	-1.25956800	3.38672200
H	-1.77893100	-0.63971000	3.02608000
C	-3.30944400	3.31016400	0.53124100
C	-3.52549800	0.34137100	-3.02724600
H	-2.56584600	-0.05770600	-2.73399500
C	5.41269800	-2.27576000	2.30826400
H	6.27227800	-1.93132800	1.74835200
C	3.71533100	4.68426400	-3.32373100
H	4.27683200	5.57430700	-3.57609900
C	-0.87889000	2.57488700	1.25979000
C	-4.06080000	-3.16901300	3.10424600
H	-4.37656000	-4.02000300	2.51513400
C	0.84822400	-2.81812900	-3.68684900
H	0.78701500	-1.76626600	-3.93843500
C	-0.79830100	4.95830900	2.26291300
H	-1.38008400	5.45552200	1.49835500
C	3.01762300	-2.20331200	2.70737500
H	2.03858000	-1.81630600	2.46581700
C	-3.21850300	-0.97179000	4.59639800
H	-2.88157400	-0.11350600	5.17183800
C	-2.05786700	2.58751200	0.47717600
C	-0.17653100	5.63520300	3.34497700
H	-0.26742400	6.70072900	3.52450800
C	4.84183900	0.45862200	-0.62860800
C	0.56154400	4.78939600	4.13481200
H	1.12301600	5.03798800	5.02613700
C	-0.53554900	3.60463100	2.24490900
C	-3.84230900	4.29916200	1.52702500
H	-3.52169200	5.32438200	1.31004200
H	-3.52151300	4.06083900	2.54488600
H	-4.93416000	4.27961800	1.48194100
C	-4.09930000	1.39314600	-2.29774100
C	-5.34721100	1.90859800	-2.67792700
H	-5.77951200	2.71782800	-2.10407300
C	4.43541700	-3.64736900	4.05102400
H	4.54715100	-4.37525700	4.84986000
C	2.12740000	2.99373700	-3.32778700
H	1.24510100	2.44658500	-3.63731100
S	4.18994600	3.72682100	-1.96813500
S	0.66254000	-5.02422400	-2.36806700
S	0.53968800	3.16478200	3.55620300

fac-**4**

Ga	0.20944200	-0.17912600	-0.05844500
O	1.65929400	-0.14713900	-1.35450700
O	-1.24433700	-0.16356000	1.25350100
O	0.12167400	-2.10957600	-0.24499200
O	0.27408400	1.77367400	0.10560900
O	-1.16061300	0.06487700	-1.43437400
O	1.52411700	-0.42201800	1.36288600
N	3.45208800	0.89012900	-2.43160200
N	-0.59764700	-4.06117400	-1.30231500
N	1.61986800	0.09789100	4.82197800
N	4.01827100	2.15904900	-2.51572700

N	-1.44077800	-4.42416600	-2.34873900
N	2.20428600	-0.14123600	3.58140400
C	2.33017300	0.90027000	-1.65016100
C	5.25254100	0.07647000	-3.84225100
H	5.61554900	1.09403900	-3.90429700
C	5.66237200	-0.53368000	2.24190100
H	6.15281700	-0.76890300	1.30113200
C	2.11719200	2.26701300	-1.26910100
C	-3.74643600	1.60713300	-4.29612000
H	-3.57903200	2.52649400	-4.84815600
C	1.03141800	2.64359100	-0.44493300
C	-0.02719500	-6.40634900	-1.04446300
H	-0.73384200	-6.62773100	-1.83363900
C	3.24404600	2.97208900	-1.83873100
C	0.69670000	-7.42002300	-0.42182200
H	0.54412200	-8.44888600	-0.73748500
C	-1.22675200	-0.21369200	2.52972900
C	5.76937400	0.02044200	4.58127300
H	6.34459400	0.22087400	5.48151600
C	-0.61668000	-2.70931200	-1.09867500
C	0.62041700	5.03703300	-1.13901400
H	0.87968400	4.78286200	-2.16150600
C	0.66999300	4.05148800	-0.14840100
C	-5.03369600	1.04203700	-4.31053700
C	-1.58037900	-2.17617700	-2.01736700
C	-2.72948400	1.00073700	-3.57937800
H	-1.74226600	1.44974300	-3.54398600
C	-1.85452700	-0.79018600	-2.08318200
C	-4.24171000	-0.75062900	-2.89861900
H	-4.44912800	-1.64377100	-2.31858600
C	0.32054800	0.09028800	4.64238700
C	4.26863000	-0.49380600	2.28073100
H	3.68833800	-0.69129300	1.39125700
C	-0.01375200	-0.14612000	3.25623900
C	-2.55731900	-0.35346700	3.16785300
C	3.61788400	-1.50630900	-2.99208800
H	2.73461300	-1.71639500	-2.40723800
C	1.25713200	-0.24214200	2.60011900
C	-2.92127900	-3.43833800	-3.96910700
H	-2.75562800	-4.41270300	-4.43600700
H	-2.71226100	-2.65175300	-4.70090400
H	-3.98156700	-3.36374500	-3.70697300
C	-4.92714300	0.17198200	3.14280400
H	-5.77959700	0.70621000	2.73537300
C	1.78617100	-5.79748600	0.98150600
H	2.49238800	-5.54920200	1.76939000
C	-3.65886000	0.32437200	2.61004200
H	-3.49747900	0.97578100	1.75726000
C	-0.54951600	0.40814900	5.82320800
H	-1.33600900	1.12230200	5.55954100
H	0.07624100	0.83946900	6.60887100
H	-1.04317300	-0.47928500	6.23234000
C	-2.78209400	-1.23049800	4.23407400
H	-1.95693700	-1.81219500	4.63152800
C	1.60748400	-7.12534500	0.59427200
H	2.17023200	-7.91983700	1.07655000
C	3.68782200	4.39879000	-1.69821600
H	3.11461200	5.08251400	-2.33282000
H	4.73817600	4.46417100	-1.99357700
H	3.58399100	4.74919400	-0.66675000
C	-0.15575400	5.67240100	1.45954400
H	-0.46277800	5.94483000	2.46427600
C	0.15891500	-5.07336100	-0.64932700
C	0.19361800	6.33368700	-0.84966400
H	0.14880400	7.06911200	-1.64454200
C	1.07169800	-4.76649900	0.37128300
H	1.21847400	-3.74072100	0.67633800
C	-0.18297000	6.66008600	0.45938900
C	4.10230000	-0.19299400	-3.08594500
C	4.37886100	0.06670000	4.64185500
H	3.86660400	0.29589900	5.56720400

C	-2.02152500	-3.32787700	-2.77309200
C	5.90905900	-0.96275300	-4.49659500
H	6.79951900	-0.74141500	-5.07932500
C	-2.95297600	-0.20821700	-2.89288500
C	-5.28307800	-0.13558300	-3.59454300
H	-6.27464900	-0.57174300	-3.56265000
C	-4.05960500	-1.41348200	4.76381800
H	-4.20292700	-2.11555000	5.57675300
C	6.42159300	-0.27852900	3.38371500
H	7.50672900	-0.31260300	3.34172500
C	3.62298300	-0.18946500	3.48855200
C	5.43506000	-2.27295100	-4.40938700
H	5.95124800	-3.08022500	-4.92157700
C	-5.13813600	-0.69945400	4.22595300
C	4.29075300	-2.53235600	-3.65549100
H	3.90847000	-3.54639100	-3.57450600
C	0.24395000	4.38361700	1.15220400
H	0.24124100	3.61312100	1.91628100
O	-0.59886800	7.89132800	0.86025500
O	-6.42102400	-0.78717100	4.66834600
O	-5.96475800	1.71820000	-5.03583200
C	-7.28618600	1.19979200	-5.09371200
H	-7.74819400	1.16395400	-4.09868700
H	-7.84816400	1.88717200	-5.72810200
H	-7.30507200	0.19650300	-5.53846800
C	-6.70563200	-1.65608600	5.75607700
H	-7.77697500	-1.56109600	5.94031600
H	-6.47055300	-2.69937900	5.50934500
H	-6.15413300	-1.36373900	6.65886600
C	-0.64521100	8.93689400	-0.10029400
H	-1.35142400	8.70911500	-0.90911000
H	-0.98739200	9.82167700	0.43898300
H	0.34608400	9.13269700	-0.52887200

mer-**4**

Ga	-0.09403200	-0.08579800	-0.05484000
O	-0.01864600	0.68556000	-1.84391800
O	0.28959600	1.70065800	0.65159300
O	-0.47050500	-1.87652500	-0.71409000
O	-2.01760600	0.20941200	0.04463300
O	1.83276200	-0.39786600	-0.20719400
O	-0.13439900	-0.80528100	1.76382400
N	-0.92111800	1.98857700	-3.55848300
N	0.14648300	-4.08853600	-1.13839300
N	1.18161300	0.14079100	4.86522800
N	-2.13824300	2.58396700	-3.87794800
N	1.31109500	-4.85067900	-1.17913200
N	0.60626900	-0.76677400	3.98050900
C	-1.00859900	1.27713900	-2.39328700
C	0.05956800	3.14849800	-5.45227400
H	-0.89773300	3.62814500	-5.60906900
C	-0.41095900	-4.35221700	4.05846300
H	-0.95834600	-5.06022200	3.44201200
C	-2.38085600	1.34960000	-1.98105700
C	5.95296900	-0.14876100	0.21641100
H	6.45993200	0.46034600	0.95797700
C	-2.83237200	0.72526500	-0.79546500
C	-1.10911900	-6.12764100	-1.53450800
H	-0.16703100	-6.65932600	-1.56202200
C	-3.00012500	2.21257500	-2.96249200
C	-2.31773300	-6.79557200	-1.71362500
H	-2.30805500	-7.86907600	-1.88371300
C	0.54271800	2.10859900	1.83586100
C	0.83164700	-3.83092900	6.05298400
H	1.26168100	-4.12939100	7.00558600
C	0.42324400	-2.77660600	-0.86842600

C	-5.24134000	0.22960100	-1.35479300
H	-4.96088000	0.05738200	-2.38865100
C	-4.26238900	0.59838500	-0.42620000
C	6.70262800	-0.65780100	-0.85860700
C	1.85284400	-2.67097700	-0.81077000
C	4.59805000	-0.41550200	0.30636300
H	4.01193300	-0.00157400	1.12034400
C	2.49380200	-1.43893400	-0.54557600
C	4.71084500	-1.71844800	-1.72078600
H	4.22188200	-2.29288700	-2.50068200
C	1.23222800	1.29795600	4.24937600
C	-0.26808200	-3.03968400	3.60843400
H	-0.69191400	-2.73342400	2.66294800
C	0.69741500	1.20020100	2.91027100
C	0.65024300	3.57939400	1.97901500
C	1.43483300	1.60750200	-4.17484000
H	1.54789600	0.91190600	-3.35630500
C	0.35002700	-0.18372900	2.76991800
C	3.69090700	-4.62209200	-0.89845900
H	3.59315700	-5.70240300	-0.76386400
H	4.24970800	-4.20595200	-0.05457500
H	4.28572700	-4.44575900	-1.80055500
C	1.29867800	5.70584000	1.00332900
H	1.76163500	6.28902500	0.21374900
C	-3.51444900	-4.72447000	-1.45701800
H	-4.44730700	-4.16767200	-1.42284100
C	1.23053000	4.32551900	0.93410900
H	1.62977100	3.79990600	0.07285700
C	1.89184500	2.44204600	4.96247500
H	2.57084000	2.98709300	4.29923700
H	2.45954100	2.04365000	5.80739200
H	1.17001900	3.16719500	5.35202500
C	0.08962200	4.26769100	3.06025400
H	-0.42264600	3.71406700	3.84021700
C	-3.52855000	-6.10175400	-1.67614600
H	-4.46895800	-6.62785500	-1.81553700
C	-4.38159600	2.79480100	-3.03207500
H	-5.12444600	2.07584700	-3.39229600
H	-4.36526700	3.63979900	-3.72528100
H	-4.71807900	3.14391800	-2.05077800
C	-5.94892900	0.55436000	1.31954400
H	-6.24941600	0.67056000	2.35595000
C	-1.10375700	-4.74226400	-1.31369900
C	-6.56647400	0.02669200	-0.96818400
H	-7.29621600	-0.27761400	-1.70939800
C	-2.31524700	-4.03550900	-1.27623100
H	-2.31439200	-2.96932300	-1.10392400
C	-6.92855500	0.20491400	0.37328100
C	0.20315600	2.24371200	-4.39007700
C	0.98833700	-2.51631200	5.62083500
H	1.52565900	-1.79094400	6.21790500
C	2.31517100	-4.02960400	-0.98909600
C	1.14252400	3.41386500	-6.28650700
H	1.01863100	4.11706500	-7.10602600
C	3.95920000	-1.23304200	-0.64611800
C	6.07197400	-1.43453400	-1.83901700
H	6.62142100	-1.81031400	-2.69434000
C	0.12850000	5.66034600	3.13046800
H	-0.33252800	6.16402400	3.97201800
C	0.13370200	-4.75798900	5.27674200
H	0.01569100	-5.78286900	5.61760200
C	0.43912100	-2.11739500	4.39384800
C	2.37348900	2.78924100	-6.07802400
H	3.21560500	3.00091200	-6.73118400
C	0.74848400	6.38561400	2.10446500
C	2.50729400	1.88943100	-5.02085700
H	3.45801900	1.39338500	-4.84346100
C	-4.63315900	0.72527700	0.92673800
H	-3.86976000	0.96313300	1.66013700
O	0.85821700	7.74018700	2.07311100
O	-8.18827500	0.05203400	0.86102100

O	8.02254600	-0.33102200	-0.86119900
C	8.83751700	-0.80948800	-1.92196200
H	8.50056300	-0.42416600	-2.89289700
H	9.84280500	-0.43872100	-1.71550200
H	8.85363700	-1.90652600	-1.95299000
C	0.31443400	8.49089900	3.14987000
H	0.52122000	9.53681000	2.91752300
H	-0.77013200	8.34510300	3.23398000
H	0.79078300	8.22917400	4.10346000
C	-9.22510900	-0.31243800	-0.03945700
H	-9.03243800	-1.29000400	-0.49972400
H	-10.13437900	-0.36920200	0.56110100
H	-9.35595400	0.44022700	-0.82760600

fac-**5**

Ga	0.74402100	-0.24263000	-0.08111600
O	2.09645100	0.37723000	-1.33414100
O	-0.63144900	-0.82612000	1.18520800
O	1.28468800	-2.07234800	-0.44280100
O	0.16901300	1.59975600	0.26710700
O	-0.63295300	-0.31941000	-1.47027700
O	2.07478800	-0.17442000	1.34281100
N	3.38458300	2.04181100	-2.34366200
N	1.21702600	-4.04958700	-1.68053600
N	2.03163500	-0.07764700	4.84138800
N	3.47356500	3.43194000	-2.33826200
N	0.52440500	-4.56710800	-2.77293500
N	2.64960300	0.04005400	3.59857000
C	2.35622400	1.60916200	-1.55465000
C	5.25676100	2.00102800	-3.88809600
H	5.22379300	3.08263900	-3.90038900
C	6.03476900	0.95161600	2.32765000
H	6.56472400	1.00713300	1.38057000
C	1.70204600	2.78857000	-1.06313900
C	-3.44919200	0.55623700	-4.43169900
H	-3.48498600	1.51250700	-4.94604700
C	0.58516800	2.71209000	-0.20280300
C	2.52557000	-6.09355800	-1.63644300
H	1.93598500	-6.44657300	-2.47240100
C	2.49356900	3.87822500	-1.59125600
C	3.53857100	-6.87679900	-1.08921100
H	3.73497300	-7.85829300	-1.51289400
C	-0.58487600	-1.00241500	2.44858200
C	5.98311200	1.20895400	4.71987100
H	6.47305700	1.46736600	5.65507400
C	0.77360200	-2.79681700	-1.36321800
C	-0.66959500	4.85703300	-0.66955900
H	-0.43493200	4.76237200	-1.72522300
C	-0.20148400	3.89776500	0.23250100
C	-4.54881400	-0.31966400	-4.50615400
C	-0.30914100	-2.51245200	-2.26079800
C	-2.31079700	0.23928300	-3.70214200
H	-1.48620900	0.94224000	-3.63656700
C	-1.00205000	-1.28316800	-2.22291000
C	-3.29739400	-1.87131300	-3.09820100
H	-3.26315500	-2.80700900	-2.54873200
C	0.80329100	-0.48120500	4.62334000
C	4.70308600	0.53681200	2.32867000
H	4.20731000	0.27725300	1.40476700
C	0.54929100	-0.63908900	3.20918100
C	-1.80164800	-1.61894000	3.03971700
C	4.36447900	-0.10363200	-3.06629000
H	3.65672700	-0.64672700	-2.45743900
C	1.77742300	-0.24174900	2.58432900
C	-1.20799600	-3.95977900	-4.33076300
H	-0.76960300	-4.80868300	-4.86163400

H	-1.24304000	-3.09670400	-5.00294600
H	-2.24196500	-4.21286400	-4.07576900
C	-4.22043800	-1.80839200	3.08815900
H	-5.17895900	-1.44794900	2.73229400
C	4.02485100	-5.15043100	0.51470800
H	4.60491800	-4.77266700	1.35250500
C	-3.06399500	-1.20910300	2.59310600
H	-3.13126300	-0.41337100	1.85771000
C	-0.11170600	-0.61438700	5.80463500
H	-1.07728000	-0.13352700	5.61715900
H	0.36291700	-0.13920900	6.66701300
H	-0.31695100	-1.65879300	6.05957000
C	-1.72732300	-2.67928000	3.95337500
H	-0.75730300	-3.05313900	4.26612300
C	4.29531600	-6.41339400	-0.01153800
H	5.08499000	-7.02821000	0.41135100
C	2.41516200	5.35703500	-1.35233000
H	1.59250100	5.82715100	-1.90048000
H	3.35135300	5.81129700	-1.68678800
H	2.26720400	5.58205500	-0.29155800
C	-1.36115200	5.07066600	2.00801600
H	-1.61780800	5.13362800	3.06158900
C	2.26035800	-4.82436600	-1.10144900
C	-1.47349900	5.91181900	-0.23391300
H	-1.82700600	6.62552300	-0.96947300
C	3.01473200	-4.34931700	-0.01783300
H	2.81493700	-3.37082800	0.39390800
C	-1.82713900	6.05373600	1.11457100
C	4.33531200	1.29865500	-3.09755900
C	4.65261500	0.79835600	4.74337700
H	4.10421000	0.73185400	5.67399100
C	-0.37179700	-3.67545500	-3.11853900
C	6.19782700	1.30150500	-4.63925600
H	6.90687900	1.85675800	-5.24781200
C	-2.20845700	-0.99684900	-3.04570400
C	-4.44832800	-1.53266700	-3.81195200
H	-5.27573800	-2.23334900	-3.81351400
C	-2.88662800	-3.28830300	4.42237200
H	-2.79026000	-4.11933300	5.11508700
C	6.68366900	1.28972300	3.51503000
H	7.72139700	1.61122400	3.50253300
C	4.00730300	0.46176300	3.54454100
C	6.23495500	-0.09382300	-4.61543800
H	6.97127500	-0.63480500	-5.20330300
C	-4.16242000	-2.86218200	4.01330300
C	5.31522300	-0.78452800	-3.82668900
H	5.33052100	-1.87065000	-3.79445300
C	-0.58318700	4.00338000	1.57906800
H	-0.25629000	3.24365500	2.28206800
C	-5.41800200	-3.55777300	4.56826900
C	-2.69266100	7.21484800	1.63476900
C	-5.79402400	0.07994100	-5.31671400
C	-6.71972800	-2.95761800	4.00385500
H	-6.82292200	-1.89563800	4.25408900
H	-6.77788300	-3.06068200	2.91437600
H	-7.58165100	-3.48243100	4.43082400
C	-5.44700000	-3.41049600	6.10863400
H	-6.33233300	-3.90940100	6.52103800
H	-4.56449400	-3.85652600	6.57901500
H	-5.48444200	-2.35506300	6.40173000
C	-5.37942300	-5.05961300	4.19734100
H	-5.36350800	-5.19475600	3.11001700
H	-4.49705700	-5.56022700	4.60904500
H	-6.26643800	-5.57041800	4.59113600
C	-3.97966600	6.64826100	2.28057400
H	-4.56875100	6.08226800	1.55008600
H	-3.75656900	5.98081400	3.11938200
H	-4.60417900	7.46539200	2.66155500
C	-1.89494700	8.01169900	2.69490300
H	-0.97719600	8.42833800	2.26452600
H	-2.49887000	8.84327300	3.07767000

H	-1.61022900	7.38679300	3.54766600
C	-3.10573000	8.18751900	0.51407000
H	-2.23623500	8.64451200	0.02814800
H	-3.70837600	7.69237400	-0.25592600
H	-3.71142800	8.99768100	0.93531300
C	-6.89502200	-0.99644600	-5.27028300
H	-7.24819200	-1.17593400	-4.24837100
H	-7.75590900	-0.66767600	-5.86302500
H	-6.55249300	-1.95025200	-5.68732200
C	-5.39304400	0.29761700	-6.79551900
H	-4.63883200	1.08413500	-6.90235700
H	-4.98229300	-0.62072400	-7.23027000
H	-6.26847900	0.59182600	-7.38705100
C	-6.37953400	1.39347100	-4.74534500
H	-6.67725200	1.26701200	-3.69826800
H	-5.65866700	2.21631900	-4.79082900
H	-7.26561800	1.69419800	-5.31742900

mer-**5**

Ga	-0.15218800	-0.33751100	-0.08329300
O	0.10351000	0.17845100	-1.94615600
O	0.38056300	1.47934400	0.41270800
O	-0.67234600	-2.15700200	-0.53188900
O	-2.03910300	0.14769700	-0.15530900
O	1.73986800	-0.84592500	-0.06618500
O	-0.37366300	-0.79922700	1.80377800
N	-0.58756900	1.24521400	-3.90480100
N	-0.24721100	-4.44457000	-0.73051900
N	0.74436500	0.46852700	4.86743200
N	-1.73408900	1.86149200	-4.40104800
N	0.84423200	-5.30719500	-0.65473500
N	0.16193800	-0.50465700	4.05936600
C	-0.79974000	0.74203100	-2.65134100
C	0.55116000	1.89400500	-5.94783600
H	-0.37390000	2.34908900	-6.27667800
C	-1.20347900	-3.94427300	4.51592600
H	-1.72176500	-4.70191500	3.93430200
C	-2.18263600	0.98229700	-2.35068900
C	5.84419600	-1.01614200	0.65094300
H	6.32859000	-0.42198900	1.42036200
C	-2.75086100	0.59832900	-1.11703900
C	-1.66509500	-6.38971200	-1.04469400
H	-0.77337400	-7.00154300	-1.00614600
C	-2.67379000	1.71035600	-3.50017800
C	-2.92228600	-6.96111300	-1.22363900
H	-3.00239000	-8.04016200	-1.32664400
C	0.59894000	2.01906400	1.54943100
C	-0.22195600	-3.19371800	6.58105000
H	0.03225500	-3.36112200	7.62449700
C	0.13860500	-3.14359900	-0.56575200
C	-5.16684700	0.19207900	-1.72600600
H	-4.85356000	-0.21060800	-2.68406500
C	-4.20708000	0.66546400	-0.82637900
C	6.62068400	-1.63061800	-0.34985900
C	1.56892000	-3.16033000	-0.44769000
C	4.46108600	-1.13647600	0.67911000
H	3.88036000	-0.63208900	1.44498100
C	2.30933200	-1.97383700	-0.25886700
C	4.55110700	-2.52073800	-1.28834500
H	4.05158700	-3.07834400	-2.07442200
C	0.97552600	1.51223600	4.10822200
C	-0.86715100	-2.73867600	3.90028400
H	-1.11777300	-2.56262000	2.86431200
C	0.56003500	1.26740500	2.74521300
C	0.88959100	3.47579800	1.50015700
C	1.78397100	0.66561500	-4.25375500

H	1.81986600	0.17925400	-3.29002600
C	0.07947200	-0.08452700	2.76069000
C	3.22474800	-5.26358100	-0.29517200
H	3.02911500	-6.32458100	-0.12012700
H	3.77020200	-4.85495900	0.56137100
H	3.87974000	-5.17593200	-1.16759900
C	1.94854300	5.33898000	0.36138100
H	2.59244700	5.68009700	-0.44107600
C	-3.93927700	-4.78253200	-1.13684800
H	-4.81991600	-4.14634400	-1.16975100
C	1.69373300	3.97353000	0.46694700
H	2.12068000	3.27965800	-0.25029000
C	1.67929200	2.68456300	4.72589400
H	2.49148900	3.04496100	4.08663200
H	2.09335300	2.37249100	5.68815700
H	1.00852000	3.53125700	4.90254400
C	0.30371000	4.38780600	2.38887700
H	-0.37227300	4.02979400	3.15897600
C	-4.06787700	-6.16472600	-1.27102400
H	-5.04636000	-6.61600200	-1.41047400
C	-3.99963200	2.36288600	-3.75978300
H	-4.77863900	1.64024400	-4.02220900
H	-3.88475000	3.05803900	-4.59542400
H	-4.35270300	2.91394200	-2.88228400
C	-5.98444400	1.11899000	0.75766000
H	-6.28065100	1.48454200	1.73670900
C	-1.54464400	-4.99863400	-0.90974200
C	-6.52028800	0.18631600	-1.38511900
H	-7.22925400	-0.20430100	-2.10628100
C	-2.68974800	-4.18935200	-0.95741000
H	-2.60054000	-3.11818200	-0.85229600
C	-6.96447500	0.66084300	-0.14356700
C	0.59399900	1.26352900	-4.69544800
C	0.12473200	-1.98360900	5.98531300
H	0.63785900	-1.21197500	6.54432900
C	1.91165100	-4.56510500	-0.48996700
C	1.69167300	1.92548800	-6.74616700
H	1.64538000	2.41674400	-7.71463500
C	3.79562200	-1.91774100	-0.27865700
C	5.93877000	-2.37304300	-1.32323200
H	6.48470500	-2.84529600	-2.13194200
C	0.54491000	5.75179700	2.26082600
H	0.06257600	6.43081300	2.95819400
C	-0.88635200	-4.18240900	5.85309400
H	-1.15362900	-5.12520400	6.32225700
C	-0.19661000	-1.75308300	4.63979800
C	2.88117700	1.33580700	-6.31469200
H	3.76819500	1.36353900	-6.94157000
C	1.38327700	6.26244000	1.25419000
C	2.91496200	0.70972900	-5.06888700
H	3.83207400	0.24497700	-4.71628100
C	-4.63356400	1.10400800	0.43713900
H	-3.89305700	1.43253400	1.15947100
C	8.14985300	-1.45952900	-0.34765900
C	8.49875400	0.04362100	-0.46550300
H	8.11059400	0.46466900	-1.39987400
H	8.08171100	0.62602500	0.36257900
H	9.58667000	0.18187100	-0.45607500
C	8.82599400	-2.20303700	-1.51508700
H	8.64072900	-3.28249700	-1.47443500
H	8.48480500	-1.83266900	-2.48850300
H	9.91029600	-2.05275100	-1.46787300
C	8.72513700	-2.01643200	0.97677500
H	8.31741800	-1.49693700	1.85018000
H	8.49830200	-3.08307100	1.08558700
H	9.81508900	-1.89676700	0.99678500
C	1.63961700	7.77689800	1.16338600
C	2.30501300	8.25967800	2.47473700
H	2.48862000	9.34015700	2.43289600
H	1.67510000	8.06223300	3.34844800
H	3.26627200	7.75807100	2.63502700

C	2.56583600	8.14647100	-0.01096300
H	3.55121500	7.67587000	0.08103400
H	2.13643200	7.85719300	-0.97679400
H	2.71919300	9.23119300	-0.03029400
C	0.29503800	8.51744900	0.96729800
H	-0.19730400	8.19733000	0.04214000
H	-0.39792300	8.33561300	1.79535200
H	0.46420100	9.59925800	0.90632700
C	-8.44875400	0.68355900	0.26051900
C	-9.36857000	0.14527600	-0.85173200
H	-9.29040100	0.73873900	-1.76977400
H	-9.14509300	-0.89905000	-1.09744000
H	-10.41161800	0.18881600	-0.51910600
C	-8.87178300	2.13913500	0.57310400
H	-9.92523100	2.17069600	0.87634900
H	-8.27836100	2.57157900	1.38530900
H	-8.75195800	2.78076800	-0.30734000
C	-8.65170300	-0.19132900	1.52076600
H	-8.36503900	-1.23115400	1.32721300
H	-8.05721800	0.16657600	2.36758400
H	-9.70554000	-0.17954600	1.82445900

I, $Ga(NO_3)(Q_1)_2$

Ga	-0.00046800	-0.00034600	0.89123000
O	-0.38774900	1.48333300	-0.26256400
O	0.38812800	-1.48537800	-0.26032100
O	-1.85456900	-0.58197800	0.88612200
O	1.85362700	0.58119900	0.88805300
N	-1.87668800	3.14020700	-0.97387700
N	1.87822100	-3.14094900	-0.97211700
N	-3.24977500	3.37591400	-0.96271300
N	3.25151800	-3.37547300	-0.96144900
C	-1.58318100	1.93220000	-0.41982100
C	-1.58625500	5.35304000	-1.93007000
H	-2.65913600	5.48059100	-1.87122800
C	-2.83616200	1.31224500	-0.10477100
C	5.52825100	2.23836400	1.95756400
H	5.69938200	2.77645500	2.88555100
C	-2.90328000	0.02609400	0.47083500
C	1.58971100	-5.35265400	-1.93146500
H	2.66292800	-5.47842000	-1.87498200
C	-3.82183600	2.31076500	-0.45785000
C	0.77405300	-6.35951000	-2.44144200
H	1.22895500	-7.28429100	-2.78609300
C	1.58389100	-1.93317400	-0.41799200
C	-5.08565400	-0.85473100	-0.43121800
H	-4.89042200	-0.34798800	-1.37089300
C	-4.16395400	-0.74635200	0.61956900
C	6.45085500	2.33221300	0.91176600
H	7.34113600	2.94458500	1.02616200
C	2.83645200	-1.31219000	-0.10326400
C	4.37968100	1.46603100	1.80808500
H	3.64440400	1.40760400	2.60399900
C	2.90271400	-0.02612300	0.47261800
C	5.08443600	0.85664000	-0.42913100
H	4.88949200	0.35022100	-1.36904000
C	0.37414000	3.96829400	-1.55384500
H	0.81876300	3.04643200	-1.20856200
C	5.30856000	-2.33664800	-0.25323400
H	5.65542200	-3.36651600	-0.36864200
H	5.57959300	-1.97805600	0.74476600
H	5.84171000	-1.71318100	-0.97756400
C	-1.17088000	-4.99388200	-2.06233300
H	-2.24638200	-4.84522900	-2.10771900
C	-0.60961200	-6.18914700	-2.51079300
H	-1.24145600	-6.97825500	-2.90866800

C	-5.30745100	2.33874400	-0.25383400
H	-5.84138400	1.71582300	-0.97805600
H	-5.65351100	3.36891300	-0.36896300
H	-5.57840900	1.98023200	0.74421600
C	-5.53025400	-2.23734600	1.95482000
H	-5.70169500	-2.77578000	2.88255100
C	1.01628100	-4.15460900	-1.48264400
C	-6.22279200	-1.64882700	-0.28765500
H	-6.92431200	-1.74059500	-1.11207700
C	-0.37236900	-3.97202900	-1.54890100
H	-0.81794700	-3.05154700	-1.20117900
C	-6.45301700	-2.32999700	0.90905400
H	-7.34373000	-2.94178400	1.02321900
C	-1.01403300	4.15324400	-1.48440600
C	3.82285100	-2.30987400	-0.45671900
C	-0.76985200	6.35927800	-2.44007600
H	-1.22381000	7.28543400	-2.78227100
C	4.16290500	0.74707500	0.62167700
C	6.22101800	1.65148100	-0.28526500
H	6.92240200	1.74416500	-1.10970100
C	0.61336500	6.18657500	-2.51253500
H	1.24579500	6.97521800	-2.91040200
C	1.17342700	4.98957500	-2.06720500
H	2.24856600	4.83908000	-2.11506600
C	-4.38114600	-1.46575700	1.80563400
H	-3.64576500	-1.40824200	2.60151800
N	-0.00263400	0.00121400	3.36309700
O	-0.00382700	0.00190600	4.56790300
O	0.29332100	-1.03314500	2.64376100
O	-0.29714900	1.03474100	2.64198100

II, $Ga(NO_3)(Q_1)_2$

Ga	0.21386800	0.27831200	0.87509500
O	0.39277500	-1.24205200	-0.28125400
O	-1.69927100	-0.00246600	0.87238400
O	2.13889300	0.56357600	0.89456000
O	0.07336200	1.80170300	-0.29971500
N	1.62831900	-3.05652800	-1.08421900
N	-3.86540700	0.54347100	0.19826000
N	2.95718800	-3.47341900	-1.11807900
N	-4.49374200	1.49009700	-0.60945500
C	1.51335000	-1.83992600	-0.48539900
C	1.03313000	-5.12370000	-2.20941300
H	2.08787800	-5.36152400	-2.25248400
C	2.84370300	-1.40680800	-0.17830500
C	0.71937300	5.00411300	-2.89930300
H	1.51897900	5.03697700	-3.63396800
C	3.09087300	-0.16689400	0.44827700
C	-6.01340200	-0.55994100	0.44946900
H	-6.42798800	0.16890200	-0.23454000
C	3.67812300	-2.51460200	-0.59059800
C	-6.80970800	-1.55763300	1.00597400
H	-7.86334100	-1.60101100	0.74337900
C	-2.52252100	0.76039400	0.25478200
C	5.37280800	0.44912100	-0.42796400
H	5.10629000	0.02690300	-1.39160300
C	4.44837200	0.41245800	0.62514300
C	0.03938000	6.17446900	-2.55041400
H	0.31081900	7.11913100	-3.01365900
C	-2.26803800	1.94759400	-0.51265500
C	0.38359900	3.79551400	-2.29558000
H	0.92333200	2.88580600	-2.53817100
C	-0.96607700	2.44740900	-0.69583200
C	-1.33477900	4.91903300	-1.00805900
H	-2.10524100	4.88881500	-0.24426400
C	-0.73624600	-3.59794200	-1.54296900

H	-1.05537700	-2.67512700	-1.08120100
C	-3.97847700	3.40207200	-1.98060400
H	-4.98817200	3.18556000	-2.33833000
H	-3.29948200	3.45726000	-2.83728500
H	-3.98711000	4.39018200	-1.51012400
C	-4.91276200	-2.41602300	2.21359700
H	-4.47471800	-3.13393000	2.90157500
C	-6.26701300	-2.49227800	1.88940400
H	-6.89232600	-3.26891100	2.32066200
C	5.15029200	-2.75036100	-0.42873500
H	5.74788200	-2.16952900	-1.13807800
H	5.35186300	-3.81063900	-0.60043200
H	5.48867300	-2.48113400	0.57682400
C	6.01373400	1.62315200	2.02412000
H	6.26153900	2.08031300	2.97784700
C	-4.65258600	-0.49221200	0.77990100
C	6.61001700	1.06835900	-0.25402100
H	7.31496400	1.10680000	-1.07977700
C	-4.09734100	-1.42398200	1.66903000
H	-3.04971100	-1.37570800	1.92812500
C	6.93690800	1.64424600	0.97492900
H	7.90469700	2.11886900	1.11217900
C	0.62620000	-3.92291600	-1.61090700
C	-3.57056900	2.31930400	-1.02704600
C	0.07877000	-5.99161700	-2.73386700
H	0.40528000	-6.92004400	-3.19471000
C	-0.66565500	3.74011700	-1.36472200
C	-0.97764100	6.13244400	-1.59524400
H	-1.48898600	7.04474800	-1.30140500
C	-1.28008300	-5.67866600	-2.67074400
H	-2.02081900	-6.35957200	-3.08046100
C	-1.67553900	-4.48179800	-2.07413000
H	-2.72929800	-4.22366400	-2.01387700
C	4.76791800	1.02781700	1.84595300
H	4.03472500	1.02902400	2.64593500
N	0.22576600	0.32465900	3.34151500
O	0.23702500	0.35058800	4.54546100
O	0.32680800	-0.76110000	2.64313800
O	0.11318700	1.37900000	2.59909100

III, $Ga(NO_3)(Q_1)_2$

Ga	-0.08917100	0.13385900	-0.68700100
O	1.79752400	-0.21582500	-0.95161300
O	-0.14992600	-1.40831800	0.47175000
O	0.26886800	1.61613000	0.49077300
O	-1.98501200	0.45630200	-0.43900600
N	4.04852300	0.15001700	-0.45980500
N	-4.72402500	-1.60988700	0.25753200
N	4.81238100	1.00190200	0.33628600
N	-4.13451800	-0.43344400	-0.20225600
C	2.72325800	0.45097900	-0.37121300
C	6.06477300	-1.12031600	-0.92452100
H	6.57965000	-0.50087900	-0.20174100
C	-5.30737500	2.77443700	-1.69830900
H	-4.90322100	3.74496100	-1.97288300
C	2.62397700	1.60455900	0.47880300
C	1.38209900	2.17900600	0.80480500
C	3.99068900	1.86094100	0.88546200
C	-1.15693700	-2.07085600	0.92060600
C	-7.15581200	1.23963200	-1.54756800
H	-8.20496700	1.00335600	-1.70378500
C	1.96660600	4.60127900	1.16935300
H	2.67787800	4.54092000	0.35192400
C	1.23219800	3.46426100	1.53434300
C	-3.76522500	-2.36487200	0.73314500
C	-4.45373600	1.82525100	-1.13635800

H	-3.40918200	2.05322200	-0.98013500
C	-2.47908900	-1.71432500	0.59755100
C	-0.79664700	-3.20633800	1.80649800
C	4.01949700	-1.68076700	-2.11094400
H	2.97058600	-1.51026700	-2.30612700
C	-2.78040200	-0.47748300	-0.06662900
C	0.70462400	-5.04224400	2.30592600
H	1.55208300	-5.66389500	2.03134800
C	0.31313400	-3.99928900	1.47123200
H	0.85430000	-3.78956600	0.55434100
C	-4.12259200	-3.73734700	1.22091000
H	-3.42866300	-4.49000000	0.83348900
H	-5.13325700	-3.97472800	0.87962900
H	-4.10513200	-3.81160200	2.31271000
C	-1.47243300	-3.44074000	3.01254300
H	-2.29159200	-2.79115200	3.30366300
C	4.55746400	2.85280500	1.85702600
H	4.60129500	3.86477500	1.44257600
H	5.57449900	2.54465000	2.11207600
H	3.95888500	2.90311600	2.77203300
C	0.05649000	4.77394200	3.20031500
H	-0.68799200	4.84121200	3.98857800
C	1.74713700	5.81582100	1.81824000
H	2.30776400	6.69654900	1.51802400
C	0.80230900	5.90067400	2.84233100
H	0.63746300	6.84582700	3.35240200
C	4.70339200	-0.89527500	-1.17179700
C	-6.32028000	0.27629000	-0.98790800
H	-6.69993500	-0.69711300	-0.70602900
C	6.73384900	-2.12841700	-1.61412400
H	7.78933900	-2.29431600	-1.41565600
C	-1.06239600	-4.47119000	3.85747500
H	-1.58084200	-4.63662500	4.79772300
C	-6.65714800	2.49272900	-1.90764800
H	-7.31273900	3.23965000	-2.34640000
C	-4.96474800	0.56833900	-0.78284600
C	6.06249900	-2.91817700	-2.54904600
H	6.58901400	-3.70289700	-3.08498100
C	0.01758400	-5.28043000	3.49972300
H	0.33146600	-6.08813300	4.15534000
C	4.70837900	-2.68606200	-2.78944500
H	4.17217000	-3.28896300	-3.51728800
C	0.25485900	3.56612400	2.53731900
H	-0.33856400	2.69268100	2.78747000
N	-0.40313900	0.21888000	-3.12954800
O	-0.44337000	-0.87585900	-2.43718400
O	-0.56105200	0.26283600	-4.32190200
O	-0.17085200	1.25844100	-2.39385200

IV, Ga(κ^1-NO_3)(Q_1)$_3^-$

Ga	-0.14716600	0.44981000	-0.24135900
O	0.87767400	0.85863100	1.42842200
O	1.64650800	-0.00178600	-0.98797000
O	-1.73505100	1.19057100	0.61793600
O	0.18082900	2.33973600	-0.76066000
N	0.97571900	1.01706700	3.76192500
N	3.43327300	0.37620300	-2.45519600
N	0.08779900	1.13773200	4.82768800
N	3.94145500	1.42904800	-3.21322800
C	0.31149500	1.02009400	2.55512100
C	2.78570300	0.84073300	5.36807200
H	2.04486300	0.89593700	6.15521500
C	-1.06395200	1.26559500	2.86662600
C	0.17834300	6.52060700	-1.37631400
H	0.10377600	7.27426700	-0.59642100
C	-2.01776900	1.42733400	1.82765300

C	5.32240500	-0.95779000	-3.19554200
H	5.66456200	-0.09902400	-3.75826900
C	-1.10957500	1.28425400	4.30715200
C	6.03225900	-2.15587500	-3.20572800
H	6.94702200	-2.22315200	-3.79060600
C	2.27574700	0.73881700	-1.80399500
C	-3.66842100	3.01978800	2.89610200
H	-2.85014200	3.50256100	3.42105800
C	-3.40292700	1.93545100	2.04942900
C	-0.08103500	6.86076300	-2.70663800
H	-0.35713500	7.88032900	-2.96443400
C	2.01991800	2.09746500	-2.19662900
C	0.51601300	5.21057500	-1.04291700
H	0.68732500	4.92840300	-0.00853500
C	0.94084600	2.82424500	-1.65113500
C	0.36004500	4.57778200	-3.37222000
H	0.39730900	3.81061300	-4.13958300
C	3.30509000	0.81742300	2.99545200
H	2.97539800	0.84399100	1.96643900
C	3.46420400	3.72919900	-3.74657400
H	4.47008500	3.64096800	-4.16650900
H	3.44150100	4.57094200	-3.04609500
H	2.76966200	3.97049400	-4.55803600
C	4.40425000	-3.14850300	-1.73686000
H	4.03506200	-3.99098500	-1.15919600
C	5.58123400	-3.25870100	-2.47770700
H	6.13978200	-4.19138000	-2.48805300
C	-2.27681500	1.34180500	5.24957800
H	-2.68603300	2.35166400	5.35586300
H	-1.94471200	1.00266300	6.23473400
H	-3.09402300	0.69919900	4.90658300
C	-5.74596000	1.84666700	1.45136900
H	-6.55302100	1.39460400	0.88068700
C	4.13908500	-0.85421300	-2.44735100
C	-4.96771700	3.51058800	3.02465500
H	-5.16274400	4.36260100	3.67138800
C	3.67807100	-1.95789100	-1.71536300
H	2.76923900	-1.88086600	-1.13762200
C	-6.01076400	2.91446700	2.31335400
H	-7.02525200	3.29248900	2.41758800
C	2.35855400	0.88487400	4.03143100
C	3.11731200	2.43813000	-3.06704500
C	4.14254300	0.72879700	5.66014600
H	4.45804600	0.69409700	6.70038900
C	0.63390400	4.23568900	-2.04279700
C	-0.00175500	5.88477300	-3.70105400
H	-0.23004000	6.13705000	-4.73342500
C	5.09065200	0.65942500	4.63730500
H	6.14854900	0.57068700	4.87044500
C	4.65964200	0.70454800	3.31084600
H	5.38122400	0.65080800	2.49962800
C	-4.44646000	1.36667100	1.29997600
H	-4.21762800	0.56416900	0.60332500
N	-1.77675700	0.51822300	-2.66703000
O	-2.23248100	-0.09028200	-3.63807200
O	-0.77410700	-0.07028900	-2.02861400
O	-2.16218900	1.62284500	-2.29156300
O	-0.24128200	-1.35181800	0.46914900
O	-2.98953300	-0.90310000	-0.53490100
N	0.12853200	-3.64328700	0.25117800
N	-0.47978200	-4.76504600	-0.29127000
C	-6.06948800	-2.65013800	-2.92133900
H	-6.46076300	-2.28014100	-3.86622400
C	1.96955800	-5.09226200	0.87274800
H	1.46474900	-5.88771700	0.34061300
C	3.17753300	-5.31620000	1.52876500
H	3.61966600	-6.30981100	1.49642200
C	-0.64302300	-2.50558800	0.07222000
C	-6.81915200	-3.55052000	-2.15898600
H	-7.79404300	-3.88311200	-2.50856900
C	-1.84495600	-2.95714400	-0.54474700

C	-4.82455400	-2.21110600	-2.47097100
H	-4.24454700	-1.49074600	-3.04202700
C	-2.99737800	-2.11866900	-0.76079500
C	-5.05872100	-3.59562300	-0.50105800
H	-4.66692200	-3.95467100	0.44757900
C	-2.48954900	-5.37273900	-1.47510100
H	-1.89603600	-6.27967200	-1.62366900
H	-2.82056100	-4.99934300	-2.45000300
H	-3.39149400	-5.63597600	-0.91304600
C	3.22455800	-3.02233700	2.24621400
H	3.70463100	-2.20311700	2.77447700
C	3.81592200	-4.28512900	2.22138300
H	4.75956100	-4.46281100	2.73111400
C	1.37451100	-3.81793200	0.90278500
C	2.01327600	-2.77766600	1.59769100
H	1.57260600	-1.79202100	1.61762300
C	-1.63995100	-4.36073400	-0.76241200
C	-4.30229900	-2.69820600	-1.26390100
C	-6.31570900	-4.01496100	-0.94262500
H	-6.90045200	-4.70353700	-0.33659800

V, Ga(κ^1-NO_3)$(Q_1)_3^-$

Ga	0.07694200	0.20385400	-0.10584200
O	-1.25603100	1.49223900	-0.82624400
O	-1.02615800	-1.17171200	-0.99300800
O	0.87704200	1.62271500	0.96125800
O	-1.14509800	0.05011300	1.48619100
N	-2.04994600	3.65705700	-1.19648900
N	-2.90341300	-2.53692000	-1.25115600
N	-1.75717200	4.95437400	-0.78308800
N	-4.04263300	-2.87875600	-0.52560300
C	-1.22468200	2.74106100	-0.58873700
C	-3.86429700	4.55830300	-2.52967400
H	-3.66433300	5.52320900	-2.08181800
C	-0.40588700	3.48816200	0.31968600
C	-3.46883100	0.90648300	4.90321400
H	-3.67554900	1.90166700	5.28850800
C	0.60050700	2.85381800	1.08692200
C	-3.74078900	-3.88080500	-3.08974900
H	-4.61660000	-4.09053500	-2.48925800
C	-0.79085000	4.86130500	0.10046100
C	-3.59966700	-4.41173900	-4.36938700
H	-4.38723100	-5.04707400	-4.76815800
C	-2.10219800	-1.67056100	-0.54396300
C	0.83317100	4.39003100	3.08630000
H	-0.23925400	4.55825100	3.07295900
C	1.41430100	3.55643700	2.12261900
C	-3.67068500	-0.21468600	5.71270300
H	-4.03547400	-0.09364200	6.72976100
C	-2.74031100	-1.49334700	0.72636000
C	-2.98655400	0.75317400	3.60509300
H	-2.79727400	1.61867600	2.97745000
C	-2.17014900	-0.65480800	1.71217800
C	-2.92723100	-1.64676200	3.91013000
H	-2.67288700	-2.63345000	3.53499900
C	-3.31028300	2.20332400	-2.73396800
H	-2.69808100	1.35841900	-2.45379400
C	-5.09105200	-2.41759300	1.59787000
H	-5.91294700	-2.92925100	1.08956400
H	-5.44224900	-1.44170200	1.95049800
H	-4.81618800	-2.99696000	2.48528800
C	-1.46920300	-3.31814300	-4.59970700
H	-0.57850800	-3.09050100	-5.17945100
C	-2.46530800	-4.13529100	-5.13488300
H	-2.35962100	-4.55079700	-6.13378800
C	-0.22057700	6.14139200	0.63757500

H	-0.51426800	6.33284300	1.67519700
H	-0.58612000	6.96718500	0.02096300
H	0.87346600	6.13080100	0.60933000
C	3.57211100	3.87076100	3.17079400
H	4.63846500	3.66248800	3.20725300
C	-2.73442500	-3.05899200	-2.55884800
C	1.61939500	4.96556200	4.08499800
H	1.15832400	5.59963400	4.83842000
C	-1.58795400	-2.77665400	-3.31979100
H	-0.80938900	-2.14369000	-2.91698400
C	2.99266100	4.71769500	4.12001200
H	3.60746700	5.17120700	4.89411800
C	-3.07268400	3.46064600	-2.15591300
C	-3.95081100	-2.27065900	0.63458500
C	-4.88031900	4.39494700	-3.46777800
H	-5.48429300	5.25501100	-3.74817200
C	-2.73700200	-0.52708100	3.09113800
C	-3.38661200	-1.48866900	5.21846400
H	-3.51365600	-2.36148200	5.85378300
C	-5.12590100	3.14727900	-4.04437100
H	-5.92045500	3.02433800	-4.77598500
C	-4.33416900	2.06121100	-3.67009000
H	-4.50543700	1.08245600	-4.11092700
C	2.78813300	3.27287200	2.18612700
H	3.21160000	2.58064800	1.46418000
N	1.59984200	1.16026400	-2.38981600
O	2.26741500	0.92296300	-3.39069000
O	1.12335500	0.10723800	-1.73918500
O	1.34350700	2.29118700	-1.97000100
O	1.12723400	-1.15865100	0.75179000
O	3.55531600	0.98123500	-0.05309600
N	2.45051100	-2.96765200	1.32208700
N	3.76107800	-3.40428100	1.27339200
C	6.80101400	0.90198700	-2.71654500
H	7.49882300	1.72994100	-2.81902300
C	1.89583300	-4.66459800	2.96374000
H	2.93793900	-4.66578500	3.26027200
C	0.97086700	-5.51661600	3.56274400
H	1.29975900	-6.18655200	4.35413800
C	2.28382200	-1.71911300	0.73568600
C	6.81759200	-0.14561400	-3.64195000
H	7.52848300	-0.13429900	-4.46516800
C	3.56361800	-1.37830000	0.22844400
C	5.88020400	0.89327100	-1.67157500
H	5.82872400	1.71369100	-0.96234300
C	3.97457700	-0.12426200	-0.40126200
C	5.00230900	-1.21653900	-2.45617700
H	4.28072600	-2.02222200	-2.36130400
C	5.89039600	-2.64662600	0.40868600
H	6.22449400	-3.50018800	1.00605700
H	6.44461200	-1.75542700	0.72597600
H	6.15526700	-2.82798300	-0.63832600
C	-0.76486300	-4.65259600	2.13331500
H	-1.79479900	-4.65558100	1.78409000
C	-0.36587000	-5.51487200	3.15691300
H	-1.08398300	-6.18421400	3.62441300
C	1.48834300	-3.79441100	1.94135700
C	0.15083500	-3.79591600	1.52263400
H	-0.15164500	-3.14265800	0.71710900
C	4.41092100	-2.47411800	0.60076200
C	4.98696900	-0.17553000	-1.51936900
C	5.90919700	-1.19841900	-3.51626700
H	5.90070800	-2.00159700	-4.24917000

Crystal Data and Experimental

Table S4. Crystal data for complex **1**

**Compound**	**Complex 1**
Formula	$C_{52}H_{40}Cl_3GaN_6O_6$
$D_{calc.}$/ g cm^{-3}	1.432
μ/mm^{-1}	2.827
Formula Weight	1020.97
Colour	clear intense brown
Shape	prism-shaped
Size/mm^3	0.16×0.04×0.03
T/K	140.00(10)
Crystal System	monoclinic
Space Group	$P2_1/n$
a/Å	13.68381(17)
b/Å	24.2144(3)
c/Å	14.38731(19)
α/°	90
β/°	96.4951(12)
γ/°	90
V/Å^3	4736.57(11)
Z	4
Z'	1
Wavelength/Å	1.54184
Radiation type	Cu$K\alpha$
Θ_{min}/°	3.591
Θ_{max}/°	76.040
Measured Refl's.	98328
Indep't Refl's	9745
Refl's I≥2σ(I)	8360
R_{int}	0.0710
Parameters	659
Restraints	181
Largest Peak/e Å^{-3}	1.088
Deepest Hole/e Å^{-3}	-0.863
GooF	1.037
wR_2 (all data)	0.1455
wR_2	0.1414
R_1 (all data)	0.0615
R_1	0.0540
CCDC number	2190069

Experimental. Single clear intense brown prism-shaped crystals of complex **1** were used as supplied. A suitable crystal with dimensions 0.16 × 0.04 × 0.03 mm3 was selected and mounted on an XtaLAB Synergy R, DW system, HyPix-Arc 150 diffractometer. During data collection, the crystal was kept at a steady T = 140.00(10) Ktion. The structure was solved

with the **ShelXT** 2018/2[1] solution program using a dual method using **Olex2** 1.5[2] as the graphical interface. The model was refined with **ShelXL** 2018/3[3] using full-matrix least-squares minimisation on F^2.

Crystal Data. $C_{52}H_{40}Cl_3GaN_6O_6$, M_r = 1020.97, monoclinic, P2$_1$/n (No. 14), a = 13.68381(17) Å, b = 24.2144(3) Å, c = 14.38731(19) Å, β = 96.4951(12)°, α = γ = 90°, V = 4736.57(11) Å^3, T = 140.00(10) K, Z = 4, Z' = 1, μ (Cu K_α) = 2.827, 98328 reflections measured, 9745 unique (R_{int} = 0.0710) which were used in all calculations. The final wR_2 was 0.1455 (all data) and R_1 was 0.0540 (I≥2 σ(I)).

Table S5. Structure Quality Indicators for complex **1**

**Reflections:**	d min (Cu\a) 2Θ=152.1° 0.79	I/σ(I) CIF 36.4	Rint CIF 7.10%	Full 135.4° 99% to 152.1° 99.9
**Refinement:**	Shift CIF 0.001	Max Peak CIF 1.1	Min Peak CIF -0.9	GooF CIF 1.037

A clear, intense brown prism-shaped crystal with dimensions 0.16 × 0.04 × 0.03 mm^3 was mounted. Data were collected using an XtaLAB Synergy R, DW system, HyPix-Arc 150 diffractometer operating at T = 140.00(10) K.

Data were measured using ω scans with Cu K_α radiation. The diffraction pattern was indexed, and the total number of runs and images was based on the strategy calculation from the program CrysAlisPro 1.171.41.110a.[4] The maximum resolution achieved was Θ = 76.040° (0.79 Å).

The unit cell was refined using CrysAlisPro 1.171.41.110a[4] on 47546 reflections, 48% of the observed reflections.

Data reduction, scaling, and absorption corrections were performed using CrysAlisPro 1.171.41.110a.[4] The final completeness is 99.90 % out to 76.040° in Θ. A Gaussian absorption correction was performed using CrysAlisPro 1.171.41.110a[4] Numerical absorption correction was based on Gaussian integration over a multifaceted crystal model. Empirical absorption correction using spherical harmonics as implemented in SCALE3 ABSPACK

scaling algorithm. The absorption coefficient μ of this material is 2.827 mm^{-1} at this wavelength (λ = 1.54184Å), and the minimum and maximum transmissions are 0.708 and 1.000.

The structure was solved in the space group $P2_1/n$ (# 14) by the ShelXT 2018/2[1] structure solution program using dual methods and refined by full-matrix least-squares minimisation on $\boldsymbol{F^2}$ using version 2018/3 of **ShelXL** 2018/3.[3] All non-hydrogen atoms were refined anisotropically. Hydrogen atom positions were calculated geometrically and refined using the riding model.

There is a single molecule in the asymmetric unit, represented by the reported sum formula. In other words: Z is 4 and Z' is 1.

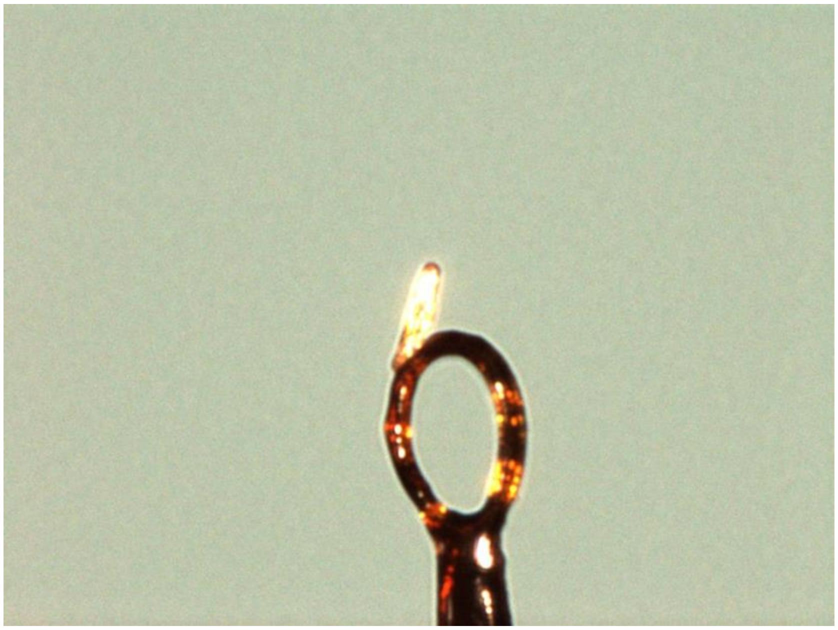

Figure S38. Image of the crystal of complex **1** on the diffractometer

Table S6. Data Plots: Diffraction Data for complex **1**

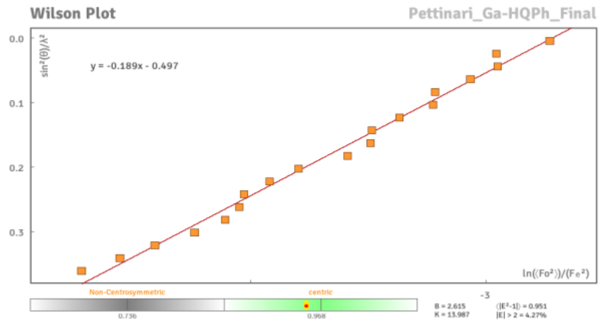

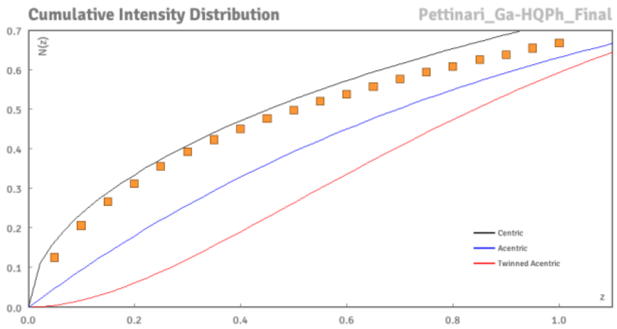

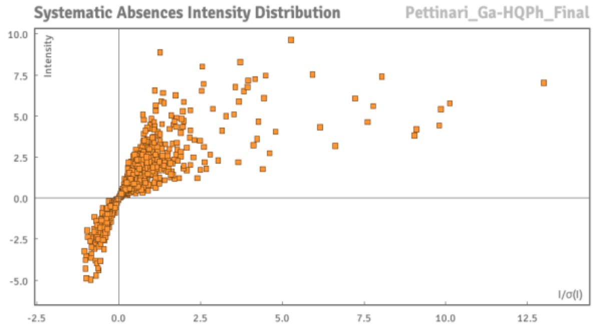

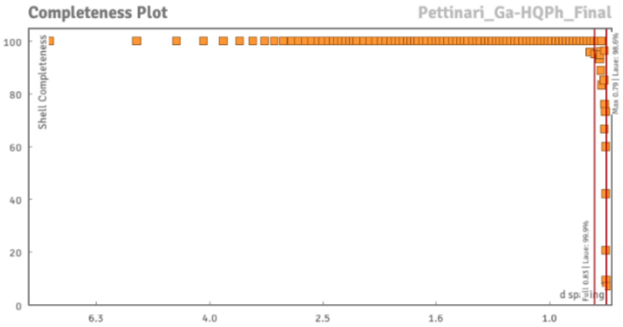

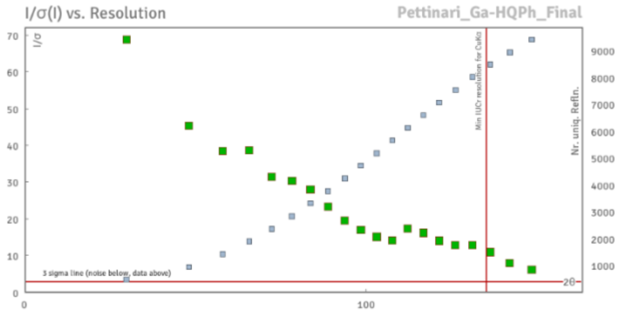

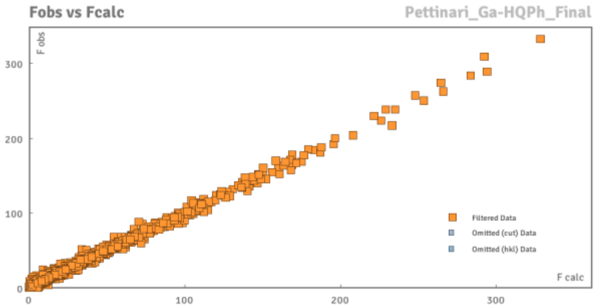

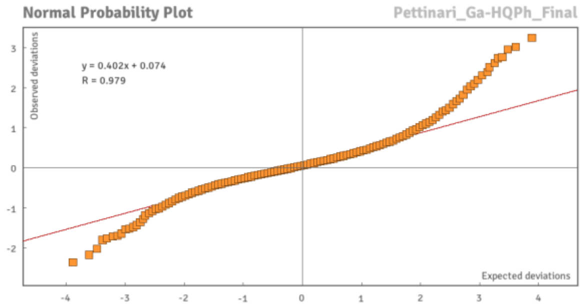

Table S7. Reflection Statistics for complex **1**

Total reflections (after filtering)	100169	Unique reflections	9745
Completeness	0.986	Mean I/σ	23.67
hkl_{max} collected	(15, 30, 17)	hkl_{min} collected	(-17, -30, -17)
hkl_{max} used	(17, 30, 17)	hkl_{min} used	(-17, 0, 0)
Lim d_{max} collected	100.0	Lim d_{min} collected	0.77
d_{max} used	14.29	d_{min} used	0.79
Friedel pairs	9758	Friedel pairs merged	1
Inconsistent equivalents	233	R_{int}	0.071
R_{sigma}	0.0275	Intensity transformed	0
Omitted reflections	0	Omitted by user (OMIT hkl)	0
Multiplicity	(7712, 7433, 5019, 3229, 1544, 869, 552, 414, 301, 279, 259, 216, 190, 148, 128, 85, 73, 57, 59, 63, 56, 61, 59, 51, 24, 10, 1, 4)	Maximum multiplicity	53
Removed systematic absences	1841	Filtered off (Shel/OMIT)	0

Table S8. Fractional Atomic Coordinates (×10^4) and Equivalent Isotropic Displacement Parameters (Å2×10^3) for complex 1. U_{eq} is defined as 1/3 of the trace of the orthogonalised U_{ij}.

Atom	x	y	z	U_{eq}
Ga1	2671.7(3)	2588.2(2)	6298.1(2)	25.22(10)
O1	3121.8(15)	2331.4(9)	7550.3(13)	30.7(4)
O2	2120.0(15)	3272.4(8)	6763.8(13)	31.9(4)
O3	1479.6(14)	2151.7(8)	6313.6(12)	27.0(4)
O4	2201.7(15)	2852.1(8)	5002.6(13)	27.9(4)
O5	3276.7(15)	1945.9(8)	5777.8(13)	28.3(4)
O6	3888.4(15)	3025.5(9)	6194.9(13)	30.6(4)
N1	3455(2)	2495.7(10)	9146.0(17)	32.2(5)
N2	3406(2)	2931.1(11)	9782.7(17)	37.4(6)
N3	703.7(18)	1410.9(10)	5496.1(16)	28.3(5)
N4	448.1(19)	1245.9(10)	4561.1(17)	32.9(5)
N5	3975(2)	1676.4(10)	4451.7(18)	33.7(5)
N6	4641(2)	1877.5(11)	3861.8(19)	38.3(6)
C1	3125(2)	2654.4(12)	8267.3(19)	28.5(6)
C2	3030(3)	3352.5(13)	9296(2)	35.8(7)
C3	2810(2)	3208.6(12)	8316.3(19)	30.6(6)
C4	3823(2)	1974.2(13)	9482(2)	34.2(6)
C5	3534(3)	1487.9(14)	9016(2)	39.2(7)
C6	3888(3)	985.8(16)	9377(3)	47.8(8)
C7	4538(3)	970.8(17)	10189(3)	53.0(10)
C8	4829(3)	1452.2(19)	10634(3)	57.1(11)
C9	4463(3)	1960.9(16)	10300(2)	44.8(8)
C10	2904(3)	3888.1(14)	9771(2)	46.9(8)
C11	2311(2)	3490.4(12)	7566.7(19)	29.1(6)
C12	1932(2)	4065.2(13)	7675(2)	34.6(6)
C13	974(3)	4150.9(14)	7876(3)	45.0(8)
C14	628(3)	4685.1(16)	7962(3)	52.9(9)
C15	1239(3)	5127.8(15)	7838(3)	55.6(10)

Atom	x	y	z	U_{eq}
C16	2191(4)	5041.7(15)	7676(3)	57.8(10)
C17	2544(3)	4505.9(14)	7592(3)	48.7(8)
C18	1167(2)	1904.5(11)	5545.3(19)	25.2(5)
C19	740(2)	1641.3(12)	4042.3(19)	28.4(6)
C20	1210(2)	2078.1(11)	4608.8(18)	25.5(5)
C21A	510(8)	1027(4)	6189(7)	32.8(17)
C21B	562(8)	1048(4)	6253(7)	34(2)
C26B	887(10)	1183(3)	7175(8)	40.3(19)
C25B	764(11)	811(4)	7889(6)	49(2)
C24B	316(9)	304(4)	7679(7)	46(2)
C23B	-9(8)	169(4)	6757(9)	46(2)
C22B	113(9)	541(5)	6044(7)	40(2)
C22A	305(9)	479(5)	5917(7)	36.9(18)
C23A	87(9)	105(5)	6580(8)	44.7(18)
C24A	92(10)	248(4)	7475(9)	51(2)
C25A	364(11)	784(4)	7775(7)	50.4(19)
C26A	547(11)	1181(4)	7106(8)	44.2(18)
C27	588(3)	1575.9(13)	3001(2)	37.3(7)
C28	1691(2)	2563.5(11)	4388.8(19)	26.6(6)
C29	1633(2)	2774.1(12)	3405.2(19)	30.1(6)
C30	2480(3)	2817.5(16)	2963(2)	44.2(8)
C31	2434(3)	3025.4(19)	2064(3)	55.8(10)
C32	1543(3)	3208.8(17)	1619(2)	55.0(10)
C33	693(3)	3174.9(15)	2065(2)	46.8(8)
C34	738(3)	2943.6(14)	2944(2)	38.9(7)
C35	3866(2)	2032.6(12)	5148.6(19)	29.3(6)
C36	4952(2)	2348.8(13)	4214(2)	33.7(6)
C37	4488(2)	2487.1(12)	5040(2)	29.2(6)
C38	3466(2)	1168.3(13)	4225(2)	37.4(7)
C39	3175(4)	841.5(15)	4918(3)	55.3(10)
C40	2701(4)	344.6(17)	4689(3)	69.7(13)
C41	2515(3)	185.1(16)	3781(3)	58.1(10)
C42	2784(3)	520.1(16)	3087(3)	54.4(10)
C43	3256(3)	1018.5(15)	3303(3)	46.5(8)
C44	5656(3)	2678.6(15)	3713(3)	49.5(9)
C45	4520(2)	2950.4(12)	5623.8(18)	27.2(6)
C46	5301(2)	3377.0(12)	5619.8(19)	28.2(6)
C47	5035(2)	3932.2(12)	5590(2)	33.6(6)
C48	5775(3)	4331.5(13)	5597(2)	41.7(8)
C49	6746(3)	4173.0(14)	5667(2)	43.1(8)
C50	7009(2)	3624.7(15)	5746(2)	41.0(7)
C51	6288(2)	3223.9(13)	5705(2)	34.8(6)
Cl1	2252.4(9)	4461.4(5)	5290.2(7)	62.7(3)
Cl2	3163.3(9)	4243.5(5)	3605.0(8)	65.1(3)
Cl3	1107.4(9)	4532.5(6)	3489.6(9)	74.8(3)
C52	2078(3)	4185.0(16)	4151(3)	55.1(10)

Table S9. Anisotropic Displacement Parameters (×10[4]) for complex **1**. The anisotropic displacement factor exponent takes the form: $-2\pi^2[h^2a^{*2} \times U_{11} + ... + 2hka^* \times b^* \times U_{12}]$

Atom	U_{11}	U_{22}	U_{33}	U_{23}	U_{13}	U_{12}
Ga1	27.68(18)	31.61(19)	16.58(16)	-2.84(12)	3.47(12)	-6.39(14)
O1	33.4(11)	38.8(11)	19.3(9)	-1.7(8)	0.3(8)	-1.5(9)
O2	36.9(11)	35.0(11)	23.6(10)	-5.3(8)	2.8(8)	-4.2(9)
O3	32.1(10)	32.7(10)	16.7(8)	-2.0(7)	4.6(7)	-5.9(8)
O4	32.6(10)	29.2(10)	22.0(9)	-0.5(7)	3.0(8)	-9.1(8)
O5	31.0(10)	30.9(10)	23.4(9)	-0.8(7)	5.2(8)	-3.0(8)
O6	30.6(10)	38.3(11)	23.2(9)	-5.3(8)	3.9(8)	-8.7(8)
N1	38.4(14)	34.9(13)	23.5(12)	-0.1(9)	4.3(10)	-2.6(10)
N2	51.2(16)	37.9(14)	23.4(12)	-2.8(10)	5.7(11)	-5.3(12)
N3	33.6(13)	27.1(12)	24.8(11)	0.2(9)	5.3(10)	-5.6(10)
N4	38.7(14)	30.7(12)	28.6(12)	-3.8(10)	0.9(10)	-7.5(10)
N5	41.8(14)	28.0(12)	32.8(13)	-4.4(10)	11.2(11)	-1.8(11)
N6	45.0(15)	35.7(14)	36.6(14)	-2.5(11)	15.1(12)	0.7(12)
C1	25.6(13)	36.0(15)	24.2(13)	3.1(11)	3.6(11)	-5.2(11)
C2	46.0(18)	37.2(16)	23.6(14)	-1.2(12)	0.7(12)	-8.4(14)
C3	35.4(15)	33.1(14)	24.0(13)	0.8(11)	6.5(11)	-4.0(12)
C4	34.3(15)	38.9(16)	30.6(15)	10.2(12)	8.2(12)	0.2(13)
C5	43.5(18)	48.4(18)	26.5(15)	4.7(13)	8.0(13)	7.4(15)
C6	58(2)	42.8(19)	45.9(19)	2.5(15)	17.5(17)	9.9(16)
C7	46(2)	55(2)	60(2)	20.4(19)	13.8(18)	16.5(17)
C8	37.3(19)	70(3)	61(2)	30(2)	-8.5(17)	-0.9(18)
C9	35.5(17)	54(2)	43.4(18)	15.0(16)	-2.8(14)	-11.5(15)
C10	72(3)	39.4(18)	29.4(16)	-5.0(13)	4.7(16)	-0.2(17)
C11	29.1(14)	33.1(14)	25.5(13)	0.9(11)	4.3(11)	-6.6(11)
C12	45.3(18)	32.3(15)	25.4(14)	0.9(11)	0.7(12)	-2.5(13)
C13	48(2)	36.5(17)	51(2)	0.5(15)	9.2(16)	-1.3(15)
C14	53(2)	46(2)	59(2)	-1.3(17)	3.9(18)	10.4(17)
C15	75(3)	35.5(18)	53(2)	-0.4(16)	-7(2)	14.1(18)
C16	74(3)	30.8(17)	67(3)	1.9(17)	4(2)	-10.4(18)
C17	52(2)	34.8(17)	59(2)	2.9(15)	6.3(17)	-6.5(15)
C18	24.6(13)	27.5(13)	23.9(13)	-0.2(10)	4.4(10)	-2.1(10)
C19	30.6(14)	28.1(14)	25.9(14)	-3.3(11)	0.0(11)	-3.5(11)
C20	29.4(14)	28.0(13)	18.9(12)	-1.4(10)	1.4(10)	-2.5(11)
C21A	37(3)	32(3)	31(3)	3(3)	10(3)	-5(3)
C21B	37(4)	28(3)	37(4)	5(3)	12(3)	-5(3)
C26B	53(4)	36(3)	36(3)	6(3)	23(4)	-4(3)
C25B	63(5)	44(3)	44(4)	7(3)	25(4)	-6(4)
C24B	56(4)	43(4)	42(4)	12(3)	23(3)	-6(3)
C23B	56(4)	35(4)	49(4)	10(4)	18(4)	-10(3)
C22B	43(4)	34(4)	44(4)	4(3)	10(3)	-5(3)
C22A	43(4)	32(3)	37(3)	1(3)	9(3)	-5(3)
C23A	53(3)	37(3)	47(4)	2(3)	19(3)	-6(3)
C24A	62(4)	42(3)	50(4)	7(3)	21(3)	-17(3)
C25A	68(5)	50(3)	39(3)	5(3)	26(3)	-18(4)
C26A	59(4)	40(3)	39(3)	-3(2)	25(3)	-16(3)
C27	47.0(18)	36.4(16)	26.5(15)	-4.0(12)	-4.0(13)	-5.1(14)
C28	27.0(14)	30.1(14)	22.7(13)	-0.2(10)	2.7(11)	-0.2(11)
C29	37.2(15)	29.7(14)	23.0(13)	0.3(11)	1.6(11)	-7.1(12)
C30	40.0(18)	61(2)	31.2(16)	8.3(15)	2.9(14)	-8.4(16)
C31	57(2)	75(3)	37.7(19)	13.4(18)	12.9(17)	-13(2)
C32	80(3)	56(2)	27.8(16)	12.2(15)	1.5(17)	-16(2)
C33	58(2)	44.0(19)	35.2(17)	4.8(14)	-8.9(16)	1.2(16)
C34	42.3(18)	44.3(18)	29.7(15)	2.1(13)	1.8(13)	1.2(14)
C35	33.8(15)	28.2(13)	25.6(13)	-0.7(11)	2.3(11)	3.2(11)
C36	38.4(16)	35.0(15)	29.3(15)	-2.0(12)	10.7(12)	2.2(13)

Atom	U_{11}	U_{22}	U_{33}	U_{23}	U_{13}	U_{12}
C37	31.3(15)	31.2(14)	25.7(14)	1.8(11)	5.3(11)	-2.5(11)
C38	40.3(17)	29.8(15)	42.1(17)	-7.0(13)	5.5(14)	0.9(13)
C39	88(3)	33.7(18)	44(2)	-4.4(15)	6.3(19)	-14.0(18)
C40	105(4)	40(2)	65(3)	-3.9(18)	10(3)	-26(2)
C41	68(3)	37.6(19)	67(3)	-11.8(18)	0(2)	-10.0(18)
C42	62(2)	50(2)	49(2)	-17.0(17)	-6.2(18)	1.5(18)
C43	57(2)	41.2(18)	40.0(18)	-6.2(14)	1.7(16)	-1.5(16)
C44	65(2)	44.1(19)	44.8(19)	-7.6(15)	31.3(18)	-7.5(17)
C45	27.8(14)	32.0(14)	21.5(12)	2.1(10)	2.0(10)	-0.3(11)
C46	29.8(14)	29.8(14)	25.3(13)	-1.7(10)	4.2(11)	-3.3(11)
C47	38.2(16)	32.5(15)	30.9(15)	-1.4(12)	7.4(12)	1.7(12)
C48	58(2)	30.4(15)	38.2(17)	-4.3(13)	13.4(15)	-5.7(14)
C49	47.7(19)	41.0(18)	44.0(18)	-8.5(14)	19.7(15)	-17.3(15)
C50	32.7(16)	51.2(19)	40.5(17)	-5.8(14)	10.5(13)	-6.4(14)
C51	34.0(16)	35.9(16)	35.1(16)	-2.0(12)	7.0(13)	-1.1(13)
Cl1	70.2(7)	64.5(6)	53.6(6)	7.3(4)	8.0(5)	-2.8(5)
Cl2	64.4(6)	67.4(6)	64.2(6)	-5.3(5)	9.9(5)	-0.4(5)
Cl3	57.9(6)	94.9(9)	69.8(7)	22.0(6)	-0.7(5)	-8.3(6)
C52	64(3)	36.3(18)	64(2)	6.0(17)	5(2)	-12.1(17)

Table S10. Bond Lengths (Å) in complex **1**

Atom	Atom	Length/Å
Ga1	O1	1.9393(19)
Ga1	O2	1.969(2)
Ga1	O3	1.9460(19)
Ga1	O4	2.0062(19)
Ga1	O5	1.951(2)
Ga1	O6	1.993(2)
O1	C1	1.294(4)
O2	C11	1.270(3)
O3	C18	1.287(3)
O4	C28	1.272(3)
O5	C35	1.296(3)
O6	C45	1.271(3)
N1	N2	1.403(3)
N1	C1	1.349(4)
N1	C4	1.424(4)
N2	C2	1.310(4)
N3	N4	1.409(3)
N3	C18	1.351(4)
N3	C21A	1.410(6)
N3	C21B	1.430(6)
N4	C19	1.304(4)
N5	N6	1.401(4)
N5	C35	1.343(4)
N5	C38	1.433(4)
N6	C36	1.301(4)
C1	C3	1.413(4)
C2	C3	1.450(4)
C2	C10	1.485(4)
C3	C11	1.389(4)
C4	C5	1.390(5)
C4	C9	1.386(5)
C5	C6	1.388(5)
C6	C7	1.386(6)
C7	C8	1.367(6)
C8	C9	1.395(5)
C11	C12	1.499(4)
C12	C13	1.390(5)
C12	C17	1.369(5)
C13	C14	1.388(5)
C14	C15	1.383(6)
C15	C16	1.365(6)
C16	C17	1.395(5)
C18	C20	1.419(4)
C19	C20	1.442(4)
C19	C27	1.497(4)
C20	C28	1.401(4)
C21A	C22A	1.402(10)
C21A	C26A	1.366(10)
C21B	C26B	1.3900
C21B	C22B	1.3900
C26B	C25B	1.3900
C25B	C24B	1.3900
C24B	C23B	1.3900
C23B	C22B	1.3900
C22A	C23A	1.373(10)
C23A	C24A	1.333(13)
C24A	C25A	1.406(12)
C25A	C26A	1.400(10)
C28	C29	1.498(4)
C29	C30	1.388(5)
C29	C34	1.387(5)
C30	C31	1.383(5)
C31	C32	1.385(6)
C32	C33	1.393(6)
C33	C34	1.377(5)
C35	C37	1.410(4)
C36	C37	1.448(4)
C36	C44	1.497(5)
C37	C45	1.399(4)
C38	C39	1.367(5)
C38	C43	1.373(5)
C39	C40	1.389(5)
C40	C41	1.359(6)
C41	C42	1.369(6)
C42	C43	1.387(5)
C45	C46	1.487(4)
C46	C47	1.392(4)
C46	C51	1.392(4)
C47	C48	1.398(5)
C48	C49	1.376(5)
C49	C50	1.377(5)
C50	C51	1.381(4)
Cl1	C52	1.761(4)
Cl2	C52	1.762(5)
Cl3	C52	1.759(4)

Table S11. Bond Angles in ° for complex **1**

Atom	Atom	Atom	Angle/°
O1	Ga1	O2	92.72(9)
O1	Ga1	O3	89.64(8)
O1	Ga1	O4	179.79(9)
O1	Ga1	O5	89.82(8)
O1	Ga1	O6	93.41(8)
O2	Ga1	O4	87.31(8)
O2	Ga1	O6	86.22(9)
O3	Ga1	O2	95.68(8)
O3	Ga1	O4	90.15(8)
O3	Ga1	O5	87.94(8)
O3	Ga1	O6	176.34(8)
O5	Ga1	O2	175.59(8)
O5	Ga1	O4	90.16(8)
O5	Ga1	O6	90.04(8)
O6	Ga1	O4	86.81(8)
C1	O1	Ga1	121.24(19)
C11	O2	Ga1	127.7(2)
C18	O3	Ga1	116.14(17)
C28	O4	Ga1	124.28(17)
C35	O5	Ga1	117.62(18)
C45	O6	Ga1	127.37(18)
N2	N1	C4	119.1(2)
C1	N1	N2	111.2(2)
C1	N1	C4	129.7(3)
C2	N2	N1	106.3(2)
N4	N3	C21A	116.2(6)
N4	N3	C21B	120.8(6)
C18	N3	N4	111.4(2)
C18	N3	C21A	132.1(6)
C18	N3	C21B	127.4(6)
C19	N4	N3	106.2(2)
N6	N5	C38	119.5(2)
C35	N5	N6	111.3(2)
C35	N5	C38	129.0(3)
C36	N6	N5	106.0(2)
O1	C1	N1	123.0(3)
O1	C1	C3	129.8(3)
N1	C1	C3	107.2(3)
N2	C2	C3	111.0(3)
N2	C2	C10	119.5(3)
C3	C2	C10	129.5(3)
C1	C3	C2	104.2(3)
C11	C3	C1	123.6(3)
C11	C3	C2	131.8(3)
C5	C4	N1	121.2(3)
C9	C4	N1	118.3(3)
C9	C4	C5	120.5(3)
C6	C5	C4	119.6(3)
C7	C6	C5	120.1(4)
C8	C7	C6	119.8(3)
C7	C8	C9	121.2(4)
C4	C9	C8	118.7(4)
O2	C11	C3	122.3(3)
O2	C11	C12	116.3(3)
C3	C11	C12	121.4(3)
C13	C12	C11	120.4(3)
C17	C12	C11	119.5(3)
C17	C12	C13	120.1(3)
C14	C13	C12	119.8(3)
C15	C14	C13	119.6(4)
C16	C15	C14	120.4(4)
C15	C16	C17	120.2(4)
C12	C17	C16	119.8(4)
O3	C18	N3	124.4(2)
O3	C18	C20	129.2(2)
N3	C18	C20	106.4(2)
N4	C19	C20	111.2(2)
N4	C19	C27	118.6(3)
C20	C19	C27	130.2(3)
C18	C20	C19	104.8(2)
C28	C20	C18	122.3(2)
C28	C20	C19	132.8(2)
C22A	C21A	N3	118.2(9)
C26A	C21A	N3	121.0(9)
C26A	C21A	C22A	120.8(6)
C26B	C21B	N3	121.8(7)
C26B	C21B	C22B	120.0
C22B	C21B	N3	118.2(7)
C21B	C26B	C25B	120.0
C24B	C25B	C26B	120.0
C23B	C24B	C25B	120.0
C24B	C23B	C22B	120.0
C23B	C22B	C21B	120.0
C23A	C22A	C21A	118.8(7)
C24A	C23A	C22A	121.5(8)
C23A	C24A	C25A	120.5(7)
C26A	C25A	C24A	119.0(7)
C21A	C26A	C25A	119.1(7)
O4	C28	C20	122.7(2)
O4	C28	C29	115.8(2)
C20	C28	C29	121.5(2)
C30	C29	C28	120.0(3)
C34	C29	C28	120.2(3)
C34	C29	C30	119.7(3)
C31	C30	C29	120.3(3)
C30	C31	C32	119.6(4)
C31	C32	C33	120.3(3)
C34	C33	C32	119.6(3)
C33	C34	C29	120.3(3)
O5	C35	N5	123.2(3)
O5	C35	C37	129.4(3)
N5	C35	C37	107.4(3)
N6	C36	C37	111.6(3)
N6	C36	C44	118.7(3)
C37	C36	C44	129.6(3)
C35	C37	C36	103.7(2)
C45	C37	C35	122.5(3)
C45	C37	C36	133.7(3)
C39	C38	N5	120.3(3)
C39	C38	C43	120.3(3)
C43	C38	N5	119.4(3)
C38	C39	C40	119.7(4)
C41	C40	C39	120.4(4)
C40	C41	C42	119.8(4)

Atom	Atom	Atom	Angle/°
C41	C42	C43	120.5(4)
C38	C43	C42	119.2(4)
O6	C45	C37	121.7(3)
O6	C45	C46	116.4(2)
C37	C45	C46	121.9(3)
C47	C46	C45	119.1(3)
C47	C46	C51	120.5(3)
C51	C46	C45	120.3(3)
C46	C47	C48	118.8(3)
C49	C48	C47	120.0(3)
C48	C49	C50	121.1(3)
C49	C50	C51	119.7(3)
C50	C51	C46	119.9(3)
Cl1	C52	Cl2	110.6(2)
Cl3	C52	Cl1	109.6(2)
Cl3	C52	Cl2	110.1(2)

Table S12. Torsion Angles in ° for complex **1**

Atom	Atom	Atom	Atom	Angle/°
Ga1	O1	C1	N1	-178.3(2)
Ga1	O1	C1	C3	1.8(4)
Ga1	O2	C11	C3	-13.5(4)
Ga1	O2	C11	C12	168.8(2)
Ga1	O3	C18	N3	146.8(2)
Ga1	O3	C18	C20	-34.2(4)
Ga1	O4	C28	C20	15.1(4)
Ga1	O4	C28	C29	-164.92(19)
Ga1	O5	C35	N5	-148.5(2)
Ga1	O5	C35	C37	31.1(4)
Ga1	O6	C45	C37	-7.8(4)
Ga1	O6	C45	C46	172.99(18)
O1	C1	C3	C2	-177.9(3)
O1	C1	C3	C11	8.3(5)
O2	C11	C12	C13	84.5(4)
O2	C11	C12	C17	-96.7(4)
O3	C18	C20	C19	-179.4(3)
O3	C18	C20	C28	4.3(5)
O4	C28	C29	C30	62.2(4)
O4	C28	C29	C34	-115.6(3)
O5	C35	C37	C36	-179.6(3)
O5	C35	C37	C45	-2.8(5)
O6	C45	C46	C47	-47.5(4)
O6	C45	C46	C51	128.4(3)
N1	N2	C2	C3	0.3(4)
N1	N2	C2	C10	-178.5(3)
N1	C1	C3	C2	2.2(3)
N1	C1	C3	C11	-171.6(3)
N1	C4	C5	C6	-178.3(3)
N1	C4	C9	C8	-179.9(3)
N2	N1	C1	O1	178.0(3)
N2	N1	C1	C3	-2.1(3)
N2	N1	C4	C5	153.3(3)
N2	N1	C4	C9	-25.3(4)
N2	C2	C3	C1	-1.6(4)
N2	C2	C3	C11	171.5(3)
N3	N4	C19	C20	0.8(3)
N3	N4	C19	C27	178.5(3)
N3	C18	C20	C19	-0.3(3)
N3	C18	C20	C28	-176.5(3)
N3	C21A	C22A	C23A	-178.1(9)
N3	C21A	C26A	C25A	-179.1(8)
N3	C21B	C26B	C25B	-177.8(10)
N3	C21B	C22B	C23B	177.9(9)
N4	N3	C18	O3	180.0(3)
N4	N3	C18	C20	0.8(3)
N4	N3	C21A	C22A	14.6(11)
N4	N3	C21A	C26A	-167.2(9)
N4	N3	C21B	C26B	174.3(6)
N4	N3	C21B	C22B	-3.6(8)
N4	C19	C20	C18	-0.4(3)
N4	C19	C20	C28	175.3(3)
N5	N6	C36	C37	-1.4(4)
N5	N6	C36	C44	-177.9(3)
N5	C35	C37	C36	0.0(3)
N5	C35	C37	C45	176.7(3)

Atom	Atom	Atom	Atom	Angle/˚
N5	C38	C39	C40	-178.6(4)
N5	C38	C43	C42	178.5(3)
N6	N5	C35	O5	178.7(3)
N6	N5	C35	C37	-0.9(3)
N6	N5	C38	C39	151.0(3)
N6	N5	C38	C43	-30.3(5)
N6	C36	C37	C35	0.9(4)
N6	C36	C37	C45	-175.2(3)
C1	N1	N2	C2	1.2(4)
C1	N1	C4	C5	-26.9(5)
C1	N1	C4	C9	154.5(3)
C1	C3	C11	O2	-1.9(5)
C1	C3	C11	C12	175.7(3)
C2	C3	C11	O2	-173.8(3)
C2	C3	C11	C12	3.8(5)
C3	C11	C12	C13	-93.2(4)
C3	C11	C12	C17	85.6(4)
C4	N1	N2	C2	-178.9(3)
C4	N1	C1	O1	-1.9(5)
C4	N1	C1	C3	178.0(3)
C4	C5	C6	C7	-1.0(5)
C5	C4	C9	C8	1.5(5)
C5	C6	C7	C8	0.0(6)
C6	C7	C8	C9	1.7(6)
C7	C8	C9	C4	-2.5(6)
C9	C4	C5	C6	0.2(5)
C10	C2	C3	C1	177.1(4)
C10	C2	C3	C11	-9.8(6)
C11	C12	C13	C14	-179.1(3)
C11	C12	C17	C16	178.9(3)
C12	C13	C14	C15	0.6(6)
C13	C12	C17	C16	-2.3(6)
C13	C14	C15	C16	-3.1(6)
C14	C15	C16	C17	2.9(7)
C15	C16	C17	C12	-0.2(6)
C17	C12	C13	C14	2.1(5)
C18	N3	N4	C19	-1.0(3)
C18	N3	C21A	C22A	-159.5(7)
C18	N3	C21A	C26A	18.8(14)
C18	N3	C21B	C26B	1.7(11)
C18	N3	C21B	C22B	-176.2(5)
C18	C20	C28	O4	7.1(4)
C18	C20	C28	C29	-172.9(3)
C19	C20	C28	O4	-168.0(3)
C19	C20	C28	C29	12.0(5)
C20	C28	C29	C30	-117.8(3)
C20	C28	C29	C34	64.4(4)
C21A	N3	N4	C19	-176.2(6)
C21A	N3	C18	O3	-5.8(8)
C21A	N3	C18	C20	175.0(7)
C21A	C22A	C23A	C24A	-1.7(14)
C21B	N3	N4	C19	-174.7(6)
C21B	N3	C18	O3	-6.9(7)
C21B	N3	C18	C20	173.9(6)
C21B	C26B	C25B	C24B	0.0
C26B	C21B	C22B	C23B	0.0
C26B	C25B	C24B	C23B	0.0
C25B	C24B	C23B	C22B	0.0
C24B	C23B	C22B	C21B	0.0

Atom	Atom	Atom	Atom	Angle/°
C22B	C21B	C26B	C25B	0.0
C22A	C21A	C26A	C25A	-0.9(14)
C22A	C23A	C24A	C25A	-2.8(15)
C23A	C24A	C25A	C26A	5.5(15)
C24A	C25A	C26A	C21A	-3.6(15)
C26A	C21A	C22A	C23A	3.6(13)
C27	C19	C20	C18	-177.7(3)
C27	C19	C20	C28	-2.1(6)
C28	C29	C30	C31	-178.3(3)
C28	C29	C34	C33	175.2(3)
C29	C30	C31	C32	2.3(6)
C30	C29	C34	C33	-2.6(5)
C30	C31	C32	C33	-1.2(6)
C31	C32	C33	C34	-1.8(6)
C32	C33	C34	C29	3.7(5)
C34	C29	C30	C31	-0.4(5)
C35	N5	N6	C36	1.5(4)
C35	N5	C38	C39	-34.0(5)
C35	N5	C38	C43	144.7(3)
C35	C37	C45	O6	-10.8(4)
C35	C37	C45	C46	168.4(3)
C36	C37	C45	O6	164.8(3)
C36	C37	C45	C46	-16.0(5)
C37	C45	C46	C47	133.3(3)
C37	C45	C46	C51	-50.8(4)
C38	N5	N6	C36	177.3(3)
C38	N5	C35	O5	3.4(5)
C38	N5	C35	C37	-176.2(3)
C38	C39	C40	C41	-0.8(8)
C39	C38	C43	C42	-2.9(6)
C39	C40	C41	C42	-1.0(8)
C40	C41	C42	C43	0.8(7)
C41	C42	C43	C38	1.1(6)
C43	C38	C39	C40	2.7(7)
C44	C36	C37	C35	176.9(4)
C44	C36	C37	C45	0.7(6)
C45	C46	C47	C48	179.3(3)
C45	C46	C51	C50	-176.9(3)
C46	C47	C48	C49	-2.4(5)
C47	C46	C51	C50	-1.1(5)
C47	C48	C49	C50	-1.0(5)
C48	C49	C50	C51	3.4(5)
C49	C50	C51	C46	-2.4(5)
C51	C46	C47	C48	3.4(4)

Table S13. Hydrogen Fractional Atomic Coordinates (×10[4]) and Equivalent Isotropic Displacement Parameters (Å[2]×10[3]) for complex **1.** U_{eq} is defined as 1/3 of the trace of the orthogonalised U_{ij}.

Atom	x	y	z	U_{eq}
H5	3096.72	1499.29	8454.39	47
H6	3684.89	651.74	9066.52	57
H7	4780.21	626.97	10435.37	64
H8	5288.49	1439.8	11180.16	68
H9	4649.58	2292.16	10626.58	54
H10A	3120.05	3850.22	10441.21	70
H10B	2209.13	3995.7	9682.68	70
H10C	3299.42	4171.86	9503.7	70
H13	557.24	3844.7	7954.3	54
H14	-23.94	4746.39	8104.52	63
H15	994.61	5493.87	7866.25	67
H16	2614.13	5347.92	7620.34	69
H17	3205.63	4446.82	7476.77	58
H26B	1193.6	1529.2	7318.37	48
H25B	986.95	902.65	8518.89	59
H24B	232.34	49.18	8166.73	55
H23B	-315.64	-177.74	6614.04	55
H22B	-108.99	448.8	5413.5	48
H22A	316.52	369.28	5284.33	44
H23A	-70.59	-264.11	6396.72	54
H24A	-90.49	-15.11	7914.41	61
H25A	423.6	876.85	8420.19	60
H26A	695.59	1551.03	7288.57	53
H27A	99.34	1844.49	2734.05	56
H27B	354.43	1201.17	2844.19	56
H27C	1212.08	1639.12	2743.9	56
H30	3094.2	2703.85	3279.27	53
H31	3010.23	3042.28	1753.51	67
H32	1511.21	3358.41	1005.55	66
H33	86.22	3310.61	1765.74	56
H34	153.34	2900.03	3234.95	47
H39	3297.1	953.85	5553.37	66
H40	2505.59	114.84	5170.74	84
H41	2199.77	-157.82	3628.22	70
H42	2646.63	410.41	2451.28	65
H43	3430.73	1253.36	2819.85	56
H44A	5439.72	3064.38	3668.96	74
H44B	5675.71	2528.34	3083.11	74
H44C	6314.46	2658.86	4059.94	74
H47	4364.33	4038	5565.86	40
H48	5606.24	4711.93	5553.16	50
H49	7243.09	4446.38	5660.46	52
H50	7683.52	3522.62	5827.83	49
H51	6464.28	2844.4	5735.64	42
H52	1903.27	3785.39	4190.99	66

Table S14. Atomic Occupancies for all atoms that are not fully occupied in complex **1**.

Atom	Occupancy
C21A	0.53(2)
C21B	0.47(2)
C26B	0.47(2)
H26B	0.47(2)
C25B	0.47(2)
H25B	0.47(2)
C24B	0.47(2)
H24B	0.47(2)
C23B	0.47(2)
H23B	0.47(2)
C22B	0.47(2)
H22B	0.47(2)
C22A	0.53(2)
H22A	0.53(2)
C23A	0.53(2)
H23A	0.53(2)
C24A	0.53(2)
H24A	0.53(2)
C25A	0.53(2)
H25A	0.53(2)
C26A	0.53(2)
H26A	0.53(2)

Table S15. Solvent masking (PLATON/SQUEEZE) information for complex

No	x	y	z	V	e	Content
1	0.000	0.500	0.000	71.2	2.7	?
2	0.500	1.000	0.500	71.2	2.7	?

Table S16. Crystal data for complex **2**

**Compound**	**Complex 2**
Formula	$C_{45}H_{33}GaN_6O_9$
$D_{calc.}$/ g cm^{-3}	1.507
μ/mm^{-1}	1.565
Formula Weight	871.49
Colour	clear intense orange
Shape	prism-shaped
Size/mm^3	0.13×0.10×0.05
T/K	140.00(10)
Crystal System	triclinic
Space Group	$P\bar{1}$
a/Å	10.69481(16)
b/Å	12.9985(2)
c/Å	15.3252(2)
α/°	73.5674(13)
β/°	84.7723(13)
γ/°	70.0432(14)
V/Å^3	1920.70(5)
Z	2
Z'	1
Wavelength/Å	1.54184
Radiation type	Cu$K\alpha$
Θ_{min}/°	3.006
Θ_{max}/°	75.757
Measured Refl's.	35042
Indep't Refl's	7779
Refl's I≥2σ(I)	7473
R_{int}	0.0133
Parameters	584
Restraints	181
Largest Peak/e Å^{-3}	1.003
Deepest Hole/e Å^{-3}	-0.875
GooF	1.050
wR_2 (all data)	0.1185
wR_2	0.1177
R_1 (all data)	0.0494
R_1	0.0480
CCDC number	2190070

Experimental. Single clear intense orange prism-shaped crystals of **complex 2** were used as supplied. A suitable crystal with dimensions 0.13 × 0.10 × 0.05 mm^3 was selected and mounted on an XtaLAB Synergy R, DW system, HyPix-Arc 150 diffractometer. The crystal was kept at a steady T = 140.00(10) K during data collection. The structure was solved with the **ShelXT** 2018/2[1] solution program using dual methods and by using **Olex2** 1.5[2] as the graphical interface. The model was refined with **ShelXL** 2018/3[3] using full-matrix least-squares minimisation on $\boldsymbol{F}^2$.

Crystal Data. $C_{45}H_{33}GaN_6O_9$, M_r = 871.49, triclinic, $P\bar{1}$ (No. 2), a = 10.69481(16) Å, b =

12.9985(2) Å, c = 15.3252(2) Å, α = 73.5674(13)°, β = 84.7723(13)°, γ = 70.0432(14)°, V = 1920.70(5) Å^3, T = 140.00(10) K, Z = 2, Z' = 1, μ (Cu K$_\alpha$) = 1.565, 35042 reflections measured, 7779 unique (R_{int} = 0.0133) which were used in all calculations. The final wR_2 was 0.1185 (all data) and R_1 was 0.0480 (I≥2σ(I)).

Table S17. Structure Quality Indicators for complex **2**

**Reflections:**	d min (Cu\a) 2Θ=151.5° 0.80	I/σ(I) CIF 81.3	Rint CIF 1.33%	Full 135.4° 97% to 151.5° 99.3
**Refinement:**	Shift CIF 0.001	Max Peak CIF 1.0	Min Peak CIF -0.9	GooF CIF 1.050

A clear intense orange prism-shaped crystal with dimensions 0.13 × 0.10 × 0.05 mm^3 was mounted. Data were collected using an XtaLAB Synergy R, DW system, HyPix-Arc 150 diffractometer operating at T = 140.00(10) K.

Data were measured using ω scans with Cu K$_\alpha$ radiation. The diffraction pattern was indexed and the total number of runs and images was based on the strategy calculation from the program CrysAlisPro 1.171.41.110a.[4] The maximum resolution achieved was Θ = 75.757° (0.80 Å).

The unit cell was refined using CrysAlisPro 1.171.41.110a[4] on 23016 reflections, 66% of the observed reflections.

Data reduction, scaling and absorption corrections were performed using CrysAlisPro 1.171.41.110a.[4] The final completeness is 99.30 % out to 75.757° in Θ. A Gaussian absorption correction was performed using CrysAlisPro 1.171.41.110a[4] Numerical absorption correction based on Gaussian integration over a multifaceted crystal model. Empirical absorption correction using spherical harmonics as implemented in SCALE3 ABSPACK scaling algorithm. The absorption coefficient μ of this material is 1.565 mm^{-1} at this wavelength (λ = 1.54184Å) and the minimum and maximum transmissions are 0.824 and 1.000.

The structure was solved in the space group $P\bar{1}$ (# 2) by the ShelXT 2018/2[1] structure solution program using dual methods and refined by full-matrix least-squares minimisation on $\boldsymbol{F^2}$ using version 2018/3 of **ShelXL** 2018/3.[3] All non-hydrogen atoms

were refined anisotropically. Hydrogen atom positions were calculated geometrically and refined using the riding model.

There is a single molecule in the asymmetric unit, which is represented by the reported sum formula. In other words: Z is 2 and Z' is 1.

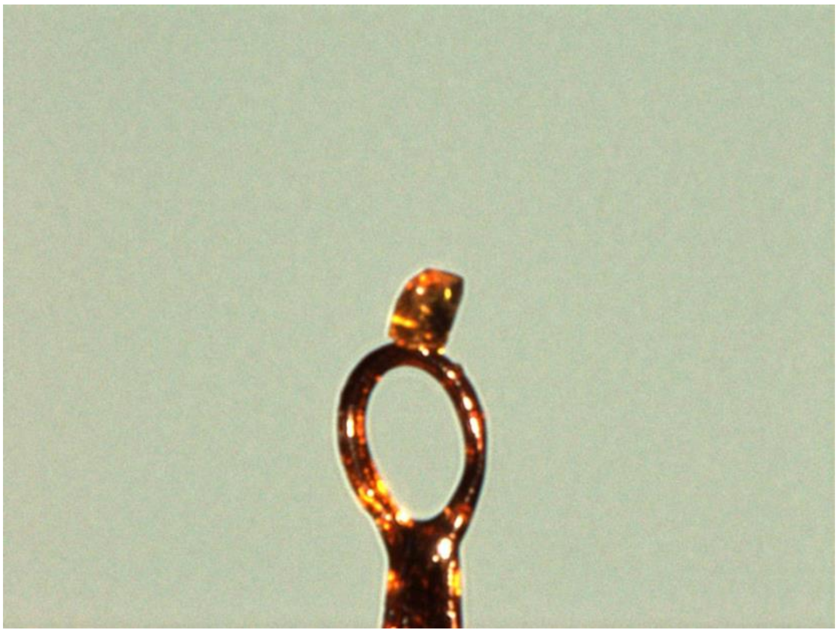

Figure S39. Image of the crystal of complex **2** on the diffractometer.

Table S18. Data plots of diffraction data for complex **2**

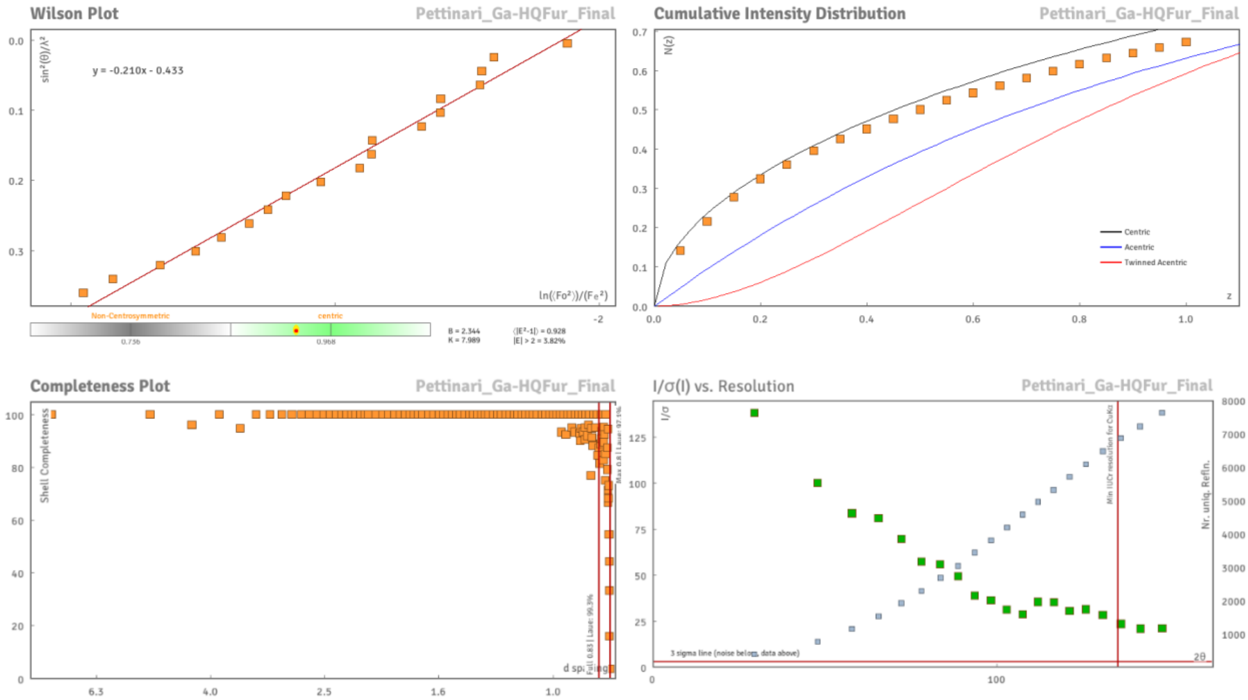

Table S19. Refinement and Data plots for complex **2**

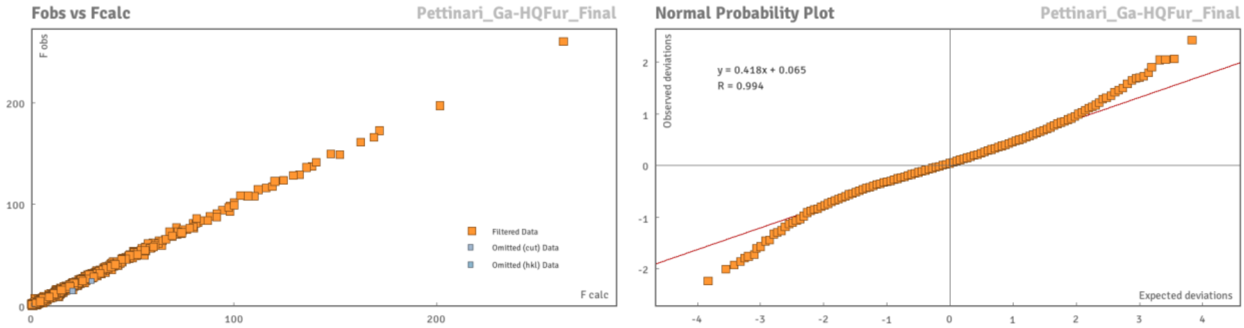

Table S20. Reflection statistics for complex **2**

Total reflections (after filtering)	35078	Unique reflections	7779
Completeness	0.971	Mean I/σ	50.54
hkl_{max} collected	(11, 16, 18)	hkl_{min} collected	(-13, -16, -19)
hkl_{max} used	(13, 16, 19)	hkl_{min} used	(-13, -15, 0)
Lim d_{max} collected	100.0	Lim d_{min} collected	0.77
d_{max} used	14.7	d_{min} used	0.8
Friedel pairs	3430	Friedel pairs merged	1
Inconsistent equivalents	3	R_{int}	0.0133
R_{sigma}	0.0123	Intensity transformed	0
Omitted reflections	0	Omitted by user (OMIT hkl)	36
Multiplicity	(3493, 3501, 1754, 627, 409, 285, 213, 169, 145, 104, 83, 66, 54, 62, 37, 34, 28, 23, 27, 27, 24, 21, 15, 8, 2)	Maximum multiplicity	26
Removed systematic absences	0	Filtered off (Shel/OMIT)	0

Table S21. Fractional Atomic Coordinates (×10⁴) and Equivalent Isotropic Displacement Parameters (Å^2×10^3) for **complex 2**. U_{eq} is defined as 1/3 of the trace of the orthogonalised U_{ij}.

Atom	x	y	z	U_{eq}
Ga1	3976.9(3)	6933.9(2)	7066.7(2)	27.72(10)
O1	5852.4(17)	6283.9(14)	6888.4(12)	29.6(4)
O2	3605.5(17)	5965.7(14)	6422.3(13)	33.4(4)
O3	2069.0(17)	7565.8(14)	7251.4(13)	33.3(4)
O4	3842.1(18)	8122.3(15)	5887.0(12)	34.4(4)
O5	4262.2(17)	7924.1(14)	7716.6(11)	28.9(4)
O6	4029.4(17)	5764.1(14)	8214.8(12)	31.7(4)
O7	4320.0(18)	3551.3(15)	5585.7(12)	34.4(4)
O8	4299(2)	9591.5(18)	4358.1(14)	47.2(5)
O9	3112(2)	4863.7(18)	10507.4(15)	51.0(5)
N1	7649(2)	4688.4(17)	6859.5(15)	30.4(4)
N2	7946(2)	3623.7(18)	6701.3(16)	34.8(5)
N3	481(2)	9270.2(18)	7362.1(16)	35.4(5)
N4	85(2)	10416.5(19)	6877.2(17)	40.0(5)
N5	3812(2)	8612.5(18)	8974.2(14)	32.6(5)
N6	3490(2)	8300.7(19)	9892.2(15)	35.1(5)

Atom	x	y	z	U_{eq}
C1	6345(2)	5282(2)	6753.1(16)	27.5(5)
C2	6821(3)	3575(2)	6468.1(18)	33.8(5)
C3	5728(3)	4611(2)	6476.4(17)	29.7(5)
C4	8693(2)	4965(2)	7147.3(17)	30.5(5)
C5	8694(3)	6084(2)	6909.6(18)	33.5(5)
C6	9736(3)	6306(2)	7192.5(19)	36.9(6)
C7	10766(3)	5450(2)	7710(2)	38.9(6)
C8	10749(3)	4344(2)	7943(2)	39.0(6)
C9	9721(3)	4098(2)	7657.7(19)	36.0(6)
C10	6892(3)	2495(2)	6264(2)	47.0(7)
C11	4366(3)	5029(2)	6273.3(17)	29.2(5)
C12	3645(3)	4494(2)	5875.0(17)	30.2(5)
C13	3392(3)	3252(2)	5258.0(18)	36.7(6)
C14	2159(3)	3976(2)	5326.9(19)	38.9(6)
C15	2323(3)	4790(2)	5725.7(19)	36.9(6)
C16	1502(2)	8653(2)	6940.7(18)	31.9(5)
C17	817(3)	10509(2)	6146(2)	37.5(6)
C18	1773(3)	9397(2)	6135.3(18)	34.2(5)
C19A	-58(7)	8958(8)	8242(3)	39.3(12)
C24A	-949(9)	9822(6)	8573(4)	45.2(13)
C23A	-1468(10)	9571(6)	9442(5)	48.9(14)
C22A	-1095(8)	8455(7)	9981(4)	48.4(12)
C21A	-204(8)	7590(6)	9650(5)	43.5(12)
C20A	314(9)	7842(7)	8780(5)	38.5(12)
C19B	9(8)	8968(8)	8268(3)	39.4(12)
C20B	-575(9)	9798(6)	8725(5)	47.9(14)
C21B	-980(11)	9493(6)	9621(5)	52.5(15)
C22B	-801(9)	8359(7)	10060(4)	50.2(13)
C23B	-217(9)	7529(6)	9603(6)	45.1(13)
C24B	188(10)	7834(7)	8707(6)	37.2(12)
C25	601(3)	11654(3)	5525(2)	52.0(8)
C26	2896(3)	9053(2)	5599.3(18)	31.7(5)
C27	3029(3)	9737(2)	4683.7(19)	36.7(6)
C28	4153(4)	10248(3)	3475(2)	60.0(9)
C29	2867(4)	10751(3)	3234(2)	58.3(9)
C30	2129(3)	10422(3)	4020(2)	45.5(7)
C31	3937(2)	7801(2)	8560.0(17)	28.5(5)
C32	3387(3)	7287(2)	10048.2(17)	31.4(5)
C33	3663(2)	6891(2)	9236.3(17)	28.7(5)
C34	3848(3)	9735(2)	8573.1(17)	32.7(5)
C35	4801(3)	10074(3)	8846(2)	44.0(7)
C36	4801(4)	11184(3)	8464(2)	54.1(8)
C37	3868(4)	11925(2)	7821(2)	49.0(8)
C38	2906(4)	11579(3)	7553(2)	48.5(7)
C39	2891(3)	10477(2)	7937.7(19)	39.6(6)
C40	3035(3)	6777(2)	11002.3(19)	40.4(6)
C41	3720(2)	5893(2)	9014.5(18)	29.8(5)
C42	3434(3)	4911(2)	9621.2(18)	32.9(5)
C43	2910(4)	3850(3)	10879(3)	57.3(9)
C44	3084(3)	3269(2)	10250(2)	44.9(7)
C45	3440(3)	3947(2)	9438(2)	35.9(6)

Table S22. Anisotropic Displacement Parameters (×10[4]) for **complex 2**. The anisotropic displacement factor exponent takes the form: $-2\pi^2[h^2a^{*2} \times U_{11} + \ldots +2hka^* \times b^* \times U_{12}]$

Atom	U_{11}	U_{22}	U_{33}	U_{23}	U_{13}	U_{12}
Ga1	26.01(16)	23.14(15)	32.77(17)	-12.25(12)	0.35(12)	-2.77(11)
O1	28.7(9)	25.3(8)	35.4(9)	-13.3(7)	2.9(7)	-6.1(7)
O2	31.8(9)	27.3(9)	42.4(10)	-18.5(8)	-2.3(7)	-3.3(7)
O3	29.5(9)	24.4(8)	45.7(10)	-13.9(7)	4.1(8)	-5.8(7)
O4	36.6(10)	28.0(9)	34.7(9)	-9.7(7)	-0.4(7)	-4.9(7)
O5	30.8(9)	27.9(8)	30.1(9)	-11.4(7)	4.9(7)	-10.5(7)
O6	33.8(9)	25.9(8)	35.4(9)	-12.5(7)	6.0(7)	-8.1(7)
O7	35.6(10)	32.5(9)	38.4(10)	-16.8(8)	0.1(8)	-9.6(8)
O8	45.4(12)	46.8(12)	43.6(11)	-8.6(9)	7.0(9)	-12.6(9)
O9	69.3(15)	39.4(11)	50.2(12)	-15.4(9)	24.0(11)	-28.3(11)
N1	28.6(10)	26.2(10)	38.7(11)	-14.4(9)	2.2(9)	-7.9(8)
N2	33.1(11)	27.6(11)	46.8(13)	-17.9(9)	3.0(9)	-7.9(9)
N3	32.5(11)	27.4(11)	42.6(12)	-10.8(9)	4.3(9)	-5.4(9)
N4	38.3(12)	26.3(11)	47.9(14)	-6.9(10)	0.3(10)	-3.8(9)
N5	42.3(12)	28.7(11)	29.6(10)	-10.7(9)	6.1(9)	-14.3(9)
N6	43.8(13)	32.8(11)	30.6(11)	-10.8(9)	6.7(9)	-15.0(10)
C1	28.6(12)	27.2(11)	26.3(11)	-9.2(9)	3.8(9)	-7.9(9)
C2	31.9(13)	26.9(12)	40.5(14)	-14.1(10)	3.7(11)	-3.9(10)
C3	32.2(13)	26.8(12)	31.7(12)	-12.0(10)	2.6(10)	-8.9(10)
C4	26.7(12)	35.8(13)	33.6(13)	-14.8(10)	5.9(10)	-13.1(10)
C5	31.9(13)	31.1(13)	36.1(13)	-11.5(10)	1.3(10)	-7.1(10)
C6	38.4(14)	34.4(13)	43.6(15)	-15.2(11)	5.5(11)	-16.6(11)
C7	33.4(14)	40.2(15)	48.3(16)	-17.3(12)	2.5(12)	-14.8(12)
C8	31.6(14)	36.4(14)	48.1(16)	-11.2(12)	-1.1(11)	-9.9(11)
C9	35.0(14)	31.1(13)	42.2(15)	-10.4(11)	1.7(11)	-11.5(11)
C10	35.3(15)	34.0(14)	76(2)	-27.9(15)	-4.3(14)	-4.7(12)
C11	33.5(13)	25.4(11)	30.2(12)	-11.4(9)	1.4(10)	-8.7(10)
C12	37.4(13)	28.3(12)	28.4(12)	-11.3(10)	1.7(10)	-12.5(10)
C13	45.8(16)	34.4(13)	36.8(14)	-15.9(11)	-0.8(11)	-16.4(12)
C14	40.5(15)	44.1(15)	38.9(14)	-16.0(12)	-1.4(11)	-18.1(12)
C15	37.5(14)	35.4(14)	39.4(14)	-17.4(11)	1.4(11)	-8.4(11)
C16	24.7(12)	34.0(13)	37.6(13)	-11.4(11)	0.8(10)	-9.0(10)
C17	27.8(13)	33.0(13)	47.4(16)	-13.6(12)	-1.8(11)	-1.9(10)
C18	32.6(13)	35.1(13)	38.5(14)	-15.0(11)	1.4(11)	-11.9(11)
C19A	30.8(19)	43.4(19)	46.8(19)	-18.2(18)	8.1(17)	-13.1(17)
C24A	39(2)	47.3(19)	50(2)	-19.4(18)	9(2)	-11(2)
C23A	41(3)	51(2)	51(2)	-19.3(19)	12(2)	-9(2)
C22A	42(2)	51(2)	51(2)	-17.7(18)	12.7(19)	-14(2)
C21A	39.0(19)	45(2)	49(2)	-14.3(18)	6.2(18)	-16.4(18)
C20A	31(2)	43.5(19)	45(2)	-22.6(17)	4.4(18)	-10.2(17)
C19B	30.5(19)	44.2(19)	47(2)	-18.1(18)	7.2(18)	-13.3(18)
C20B	40(3)	49(2)	50(2)	-16.3(19)	9(2)	-9(2)
C21B	42(3)	54(2)	51(2)	-17(2)	12(2)	-3(2)
C22B	42(2)	52(2)	51(2)	-13.9(19)	12.9(19)	-12(2)
C23B	40(2)	46(2)	49(2)	-15.3(19)	5.9(19)	-13.8(19)
C24B	28.5(19)	42.8(19)	44(2)	-18.8(18)	4.5(18)	-11.7(17)
C25	51.3(18)	32.7(15)	61(2)	-8.6(14)	6.8(15)	-5.0(13)
C26	32.2(13)	28.3(12)	35.2(13)	-10.3(10)	-1.2(10)	-8.8(10)
C27	41.2(15)	34.9(14)	34.8(13)	-8.7(11)	2.3(11)	-14.7(11)
C28	75(2)	53(2)	50.9(19)	-14.2(16)	29.6(18)	-25.5(18)
C29	89(3)	39.9(17)	33.7(15)	-4.0(13)	-0.5(16)	-9.9(17)
C30	48.5(17)	45.0(16)	38.8(15)	-8.9(13)	-4.4(13)	-11.0(13)
C31	24.8(11)	27.4(12)	34.3(12)	-12.6(10)	2.1(9)	-7.0(9)
C32	32.3(13)	29.5(12)	32.8(13)	-10.4(10)	4.4(10)	-10.2(10)
C33	26.2(12)	28.8(12)	31.5(12)	-9.8(10)	3.3(9)	-9.1(9)
C34	42.6(14)	28.2(12)	30.7(12)	-11.7(10)	8.3(10)	-14.9(11)
C35	51.4(17)	38.2(15)	46.2(16)	-12.4(13)	-4.9(13)	-17.3(13)

Atom	U_{11}	U_{22}	U_{33}	U_{23}	U_{13}	U_{12}
C36	70(2)	44.3(17)	62(2)	-20.6(16)	2.2(17)	-32.6(17)
C37	75(2)	30.7(14)	44.3(16)	-12.9(12)	16.0(15)	-22.8(15)
C38	66(2)	33.6(15)	38.3(15)	-7.4(12)	3.5(14)	-9.5(14)
C39	47.4(16)	33.1(14)	39.4(14)	-11.5(11)	0.5(12)	-13.3(12)
C40	51.4(17)	39.9(15)	32.9(14)	-11.6(11)	9.6(12)	-19.5(13)
C41	22.9(11)	27.8(12)	37.1(13)	-10.0(10)	2.5(10)	-6.0(9)
C42	28.8(12)	30.7(13)	39.4(14)	-9.2(11)	2.5(10)	-10.7(10)
C43	70(2)	42.6(17)	61(2)	-9.3(15)	24.1(17)	-30.9(16)
C44	38.5(15)	31.7(14)	65(2)	-6.7(13)	-0.6(14)	-17.7(12)
C45	32.8(13)	29.7(13)	46.2(15)	-9.7(11)	-5.0(11)	-10.7(11)

Table S23. Bond lengths in Å for complex **2**

Atom	Atom	Length/Å
Ga1	O1	1.9198(17)
Ga1	O2	1.9579(17)
Ga1	O3	1.9494(17)
Ga1	O4	1.9978(18)
Ga1	O5	1.9468(17)
Ga1	O6	1.9639(18)
O1	C1	1.297(3)
O2	C11	1.282(3)
O3	C16	1.296(3)
O4	C26	1.275(3)
O5	C31	1.287(3)
O6	C41	1.282(3)
O7	C12	1.366(3)
O7	C13	1.361(3)
O8	C27	1.372(3)
O8	C28	1.371(4)
O9	C42	1.360(3)
O9	C43	1.361(4)
N1	N2	1.397(3)
N1	C1	1.342(3)
N1	C4	1.423(3)
N2	C2	1.312(4)
N3	N4	1.397(3)
N3	C16	1.349(3)
N3	C19A	1.423(5)
N3	C19B	1.427(5)
N4	C17	1.308(4)
N5	N6	1.395(3)
N5	C31	1.342(3)
N5	C34	1.427(3)
N6	C32	1.311(3)
C1	C3	1.424(3)
C2	C3	1.454(3)
C2	C10	1.499(4)
C3	C11	1.400(4)
C4	C5	1.396(4)
C4	C9	1.379(4)
C5	C6	1.375(4)
C6	C7	1.380(4)
C7	C8	1.385(4)
C8	C9	1.381(4)
C11	C12	1.463(3)
C12	C15	1.356(4)
C13	C14	1.349(4)
C14	C15	1.428(4)
C16	C18	1.412(4)
C17	C18	1.461(4)
C17	C25	1.475(4)
C18	C26	1.407(4)
C19A	C24A	1.3900
C19A	C20A	1.3900
C24A	C23A	1.3900
C23A	C22A	1.3900
C22A	C21A	1.3900
C21A	C20A	1.3900
C19B	C20B	1.3900
C19B	C24B	1.3900
C20B	C21B	1.3900
C21B	C22B	1.3900
C22B	C23B	1.3900
C23B	C24B	1.3900
C26	C27	1.458(4)
C27	C30	1.355(4)
C28	C29	1.341(6)
C29	C30	1.422(5)
C31	C33	1.431(3)
C32	C33	1.446(3)
C32	C40	1.497(4)
C33	C41	1.413(3)
C34	C35	1.376(4)
C34	C39	1.379(4)
C35	C36	1.395(4)
C36	C37	1.371(5)
C37	C38	1.387(5)
C38	C39	1.391(4)
C41	C42	1.459(3)
C42	C45	1.357(4)
C43	C44	1.348(5)
C44	C45	1.411(4)

Table S24. Bond Angles in ° for complex **2**

Atom	Atom	Atom	Angle/°
O1	Ga1	O2	90.73(7)
O1	Ga1	O3	178.92(7)
O1	Ga1	O4	90.23(7)
O1	Ga1	O5	91.80(7)
O1	Ga1	O6	91.88(7)
O2	Ga1	O4	89.39(8)
O2	Ga1	O6	89.03(8)
O3	Ga1	O2	88.42(7)
O3	Ga1	O4	90.42(8)
O3	Ga1	O6	87.45(8)
O5	Ga1	O2	177.47(7)
O5	Ga1	O3	89.05(7)
O5	Ga1	O4	90.67(7)
O5	Ga1	O6	90.83(7)
O6	Ga1	O4	177.38(8)
C1	O1	Ga1	120.66(16)
C11	O2	Ga1	129.81(17)
C16	O3	Ga1	117.98(16)
C26	O4	Ga1	128.33(17)
C31	O5	Ga1	119.56(15)
C41	O6	Ga1	128.52(16)
C13	O7	C12	106.7(2)
C28	O8	C27	105.4(2)
C42	O9	C43	106.5(2)
N2	N1	C4	118.5(2)
C1	N1	N2	111.6(2)
C1	N1	C4	129.8(2)
C2	N2	N1	106.3(2)
N4	N3	C19A	118.8(4)
N4	N3	C19B	118.6(4)
C16	N3	N4	110.5(2)
C16	N3	C19A	129.9(4)
C16	N3	C19B	129.2(4)
C17	N4	N3	107.7(2)
N6	N5	C34	119.2(2)
C31	N5	N6	112.5(2)
C31	N5	C34	128.0(2)
C32	N6	N5	106.0(2)
O1	C1	N1	121.6(2)
O1	C1	C3	131.0(2)
N1	C1	C3	107.4(2)
N2	C2	C3	111.2(2)
N2	C2	C10	116.1(2)
C3	C2	C10	132.8(2)
C1	C3	C2	103.5(2)
C11	C3	C1	120.7(2)
C11	C3	C2	135.8(2)
C5	C4	N1	121.1(2)
C9	C4	N1	118.5(2)
C9	C4	C5	120.4(2)
C6	C5	C4	118.9(2)
C5	C6	C7	121.4(3)
C6	C7	C8	119.1(3)
C9	C8	C7	120.7(3)
C4	C9	C8	119.6(2)
O2	C11	C3	121.5(2)
O2	C11	C12	112.3(2)
C3	C11	C12	126.2(2)
O7	C12	C11	120.3(2)
C15	C12	O7	109.9(2)
C15	C12	C11	129.8(2)
C14	C13	O7	110.8(2)
C13	C14	C15	106.0(2)
C12	C15	C14	106.6(2)
O3	C16	N3	123.2(2)
O3	C16	C18	129.0(2)
N3	C16	C18	107.8(2)
N4	C17	C18	109.9(2)
N4	C17	C25	117.9(2)
C18	C17	C25	132.1(3)
C16	C18	C17	104.1(2)
C26	C18	C16	122.1(2)
C26	C18	C17	132.9(3)
C24A	C19A	N3	117.5(5)
C24A	C19A	C20A	120.0
C20A	C19A	N3	122.4(5)
C23A	C24A	C19A	120.0
C22A	C23A	C24A	120.0
C23A	C22A	C21A	120.0
C20A	C21A	C22A	120.0
C21A	C20A	C19A	120.0
C20B	C19B	N3	120.2(6)
C20B	C19B	C24B	120.0
C24B	C19B	N3	119.7(6)
C19B	C20B	C21B	120.0
C22B	C21B	C20B	120.0
C23B	C22B	C21B	120.0
C22B	C23B	C24B	120.0
C23B	C24B	C19B	120.0
O4	C26	C18	121.7(2)
O4	C26	C27	116.7(2)
C18	C26	C27	121.6(2)
O8	C27	C26	116.7(2)
C30	C27	O8	110.4(3)
C30	C27	C26	132.5(3)
C29	C28	O8	111.5(3)
C28	C29	C30	106.1(3)
C27	C30	C29	106.6(3)
O5	C31	N5	121.5(2)
O5	C31	C33	132.4(2)
N5	C31	C33	106.2(2)
N6	C32	C33	111.1(2)
N6	C32	C40	115.6(2)
C33	C32	C40	133.3(2)
C31	C33	C32	104.2(2)
C41	C33	C31	120.3(2)
C41	C33	C32	135.5(2)
C35	C34	N5	119.9(3)
C35	C34	C39	121.1(3)
C39	C34	N5	119.0(2)
C34	C35	C36	119.1(3)
C37	C36	C35	120.4(3)
C36	C37	C38	120.1(3)
C37	C38	C39	119.8(3)

Atom	Atom	Atom	Angle/°
C34	C39	C38	119.4(3)
O6	C41	C33	121.7(2)
O6	C41	C42	111.8(2)
C33	C41	C42	126.5(2)
O9	C42	C41	121.3(2)
C45	C42	O9	109.8(2)
C45	C42	C41	128.9(3)
C44	C43	O9	110.6(3)
C43	C44	C45	106.3(3)
C42	C45	C44	106.8(3)

Table S25. Torsion Angles in ° for complex **2**

Atom	Atom	Atom	Atom	Angle/°
Ga1	O1	C1	N1	-162.53(18)
Ga1	O1	C1	C3	18.3(4)
Ga1	O2	C11	C3	-8.5(4)
Ga1	O2	C11	C12	171.93(16)
Ga1	O3	C16	N3	-145.2(2)
Ga1	O3	C16	C18	36.3(3)
Ga1	O4	C26	C18	2.7(4)
Ga1	O4	C26	C27	-176.77(17)
Ga1	O5	C31	N5	-163.78(18)
Ga1	O5	C31	C33	17.1(4)
Ga1	O6	C41	C33	-19.6(3)
Ga1	O6	C41	C42	159.63(17)
O1	C1	C3	C2	-179.1(3)
O1	C1	C3	C11	2.0(4)
O2	C11	C12	O7	173.3(2)
O2	C11	C12	C15	-6.4(4)
O3	C16	C18	C17	179.6(3)
O3	C16	C18	C26	-10.0(4)
O4	C26	C27	O8	-25.0(4)
O4	C26	C27	C30	146.0(3)
O5	C31	C33	C32	178.9(3)
O5	C31	C33	C41	-0.5(4)
O6	C41	C42	O9	178.6(2)
O6	C41	C42	C45	-1.1(4)
O7	C12	C15	C14	0.6(3)
O7	C13	C14	C15	0.1(3)
O8	C27	C30	C29	-1.4(4)
O8	C28	C29	C30	1.5(4)
O9	C42	C45	C44	0.8(3)
O9	C43	C44	C45	1.1(4)
N1	N2	C2	C3	-0.8(3)
N1	N2	C2	C10	-179.9(2)
N1	C1	C3	C2	1.6(3)
N1	C1	C3	C11	-177.3(2)
N1	C4	C5	C6	-179.0(2)
N1	C4	C9	C8	179.8(2)
N2	N1	C1	O1	178.4(2)
N2	N1	C1	C3	-2.2(3)
N2	N1	C4	C5	148.7(2)
N2	N1	C4	C9	-30.3(3)
N2	C2	C3	C1	-0.5(3)
N2	C2	C3	C11	178.1(3)
N3	N4	C17	C18	-1.5(3)
N3	N4	C17	C25	-178.9(3)
N3	C16	C18	C17	0.9(3)
N3	C16	C18	C26	171.3(2)

Atom	Atom	Atom	Atom	Angle/°
N3	C19A	C24A	C23A	-177.6(6)
N3	C19A	C20A	C21A	177.4(7)
N3	C19B	C20B	C21B	-177.1(6)
N3	C19B	C24B	C23B	177.1(6)
N4	N3	C16	O3	179.3(2)
N4	N3	C16	C18	-1.9(3)
N4	N3	C19A	C24A	-0.5(7)
N4	N3	C19A	C20A	-178.0(4)
N4	N3	C19B	C20B	-14.2(7)
N4	N3	C19B	C24B	168.6(4)
N4	C17	C18	C16	0.4(3)
N4	C17	C18	C26	-168.5(3)
N5	N6	C32	C33	1.0(3)
N5	N6	C32	C40	-179.6(2)
N5	C31	C33	C32	-0.3(3)
N5	C31	C33	C41	-179.7(2)
N5	C34	C35	C36	-178.4(3)
N5	C34	C39	C38	179.0(3)
N6	N5	C31	O5	-178.4(2)
N6	N5	C31	C33	0.9(3)
N6	N5	C34	C35	66.6(3)
N6	N5	C34	C39	-111.1(3)
N6	C32	C33	C31	-0.4(3)
N6	C32	C33	C41	178.8(3)
C1	N1	N2	C2	1.9(3)
C1	N1	C4	C5	-35.3(4)
C1	N1	C4	C9	145.6(3)
C1	C3	C11	O2	-7.7(4)
C1	C3	C11	C12	171.8(2)
C2	C3	C11	O2	173.9(3)
C2	C3	C11	C12	-6.7(5)
C3	C11	C12	O7	-6.2(4)
C3	C11	C12	C15	174.1(3)
C4	N1	N2	C2	178.5(2)
C4	N1	C1	O1	2.3(4)
C4	N1	C1	C3	-178.4(2)
C4	C5	C6	C7	-0.6(4)
C5	C4	C9	C8	0.7(4)
C5	C6	C7	C8	0.4(4)
C6	C7	C8	C9	0.4(4)
C7	C8	C9	C4	-0.9(4)
C9	C4	C5	C6	0.0(4)
C10	C2	C3	C1	178.4(3)
C10	C2	C3	C11	-3.0(5)
C11	C12	C15	C14	-179.6(3)
C12	O7	C13	C14	0.3(3)
C13	O7	C12	C11	179.7(2)
C13	O7	C12	C15	-0.5(3)
C13	C14	C15	C12	-0.4(3)
C16	N3	N4	C17	2.2(3)
C16	N3	C19A	C24A	168.1(5)
C16	N3	C19A	C20A	-9.4(7)
C16	N3	C19B	C20B	149.4(5)
C16	N3	C19B	C24B	-27.8(7)
C16	C18	C26	O4	-12.2(4)
C16	C18	C26	C27	167.2(2)
C17	C18	C26	O4	155.0(3)
C17	C18	C26	C27	-25.5(4)
C18	C26	C27	O8	155.5(2)

Atom	Atom	Atom	Atom	Angle/˚
C18	C26	C27	C30	-33.4(5)
C19A	N3	N4	C17	172.8(4)
C19A	N3	C16	O3	10.0(6)
C19A	N3	C16	C18	-171.2(5)
C19A	C24A	C23A	C22A	0.0
C24A	C19A	C20A	C21A	0.0
C24A	C23A	C22A	C21A	0.0
C23A	C22A	C21A	C20A	0.0
C22A	C21A	C20A	C19A	0.0
C20A	C19A	C24A	C23A	0.0
C19B	N3	N4	C17	168.6(5)
C19B	N3	C16	O3	14.7(7)
C19B	N3	C16	C18	-166.5(6)
C19B	C20B	C21B	C22B	0.0
C20B	C19B	C24B	C23B	0.0
C20B	C21B	C22B	C23B	0.0
C21B	C22B	C23B	C24B	0.0
C22B	C23B	C24B	C19B	0.0
C24B	C19B	C20B	C21B	0.0
C25	C17	C18	C16	177.3(3)
C25	C17	C18	C26	8.4(5)
C26	C27	C30	C29	-172.9(3)
C27	O8	C28	C29	-2.3(4)
C28	O8	C27	C26	175.2(3)
C28	O8	C27	C30	2.2(3)
C28	C29	C30	C27	-0.1(4)
C31	N5	N6	C32	-1.2(3)
C31	N5	C34	C35	-120.0(3)
C31	N5	C34	C39	62.3(4)
C31	C33	C41	O6	0.8(4)
C31	C33	C41	C42	-178.4(2)
C32	C33	C41	O6	-178.3(3)
C32	C33	C41	C42	2.5(5)
C33	C41	C42	O9	-2.2(4)
C33	C41	C42	C45	178.1(3)
C34	N5	N6	C32	173.2(2)
C34	N5	C31	O5	7.8(4)
C34	N5	C31	C33	-172.9(2)
C34	C35	C36	C37	-0.3(5)
C35	C34	C39	C38	1.3(4)
C35	C36	C37	C38	0.7(5)
C36	C37	C38	C39	-0.1(5)
C37	C38	C39	C34	-0.9(4)
C39	C34	C35	C36	-0.7(5)
C40	C32	C33	C31	-179.7(3)
C40	C32	C33	C41	-0.5(5)
C41	C42	C45	C44	-179.4(3)
C42	O9	C43	C44	-0.6(4)
C43	O9	C42	C41	-180.0(3)
C43	O9	C42	C45	-0.2(3)
C43	C44	C45	C42	-1.2(3)

Table S26. Hydrogen Fractional Atomic Coordinates (×10[4]) and Equivalent Isotropic Displacement Parameters (Å^2×10^3) for complex **2.** U_{eq} is defined as 1/3 of the trace of the orthogonalised U_{ij}.

Atom	x	y	z	U_{eq}
H5	7986.61	6681.81	6558.2	40
H6	9746.51	7064.76	7028.44	44
H7	11477.04	5616.04	7903.65	47
H8	11451.03	3749.79	8301.68	47
H9	9722.69	3336.65	7812.34	43
H10A	6199.51	2213.14	6604.13	71
H10B	7767.47	1924.12	6443.07	71
H10C	6753.97	2640.61	5610.44	71
H13	3587	2619.54	5013.79	44
H14	1346.1	3948.65	5146.77	47
H15	1638.89	5417.41	5860.27	44
H24A	-1203.56	10585.14	8205.02	54
H23A	-2076.71	10162.05	9668.47	59
H22A	-1449.86	8283.26	10574.73	58
H21A	50.15	6827.55	10017.55	52
H20A	923.32	7250.61	8554.1	46
H20B	-697.73	10572.86	8425.29	57
H21B	-1379.51	10059.88	9934.15	63
H22B	-1078.2	8150.64	10672.9	60
H23B	-95.11	6754.36	9902.8	54
H24B	586.68	7267.31	8393.93	45
H25A	82.37	11747.98	4995.7	78
H25B	1462.07	11745.87	5323.44	78
H25C	115.07	12227.31	5844.03	78
H28	4872.26	10335.25	3082.66	72
H29	2518.86	11230.94	2655.29	70
H30	1187.74	10639.88	4069.31	55
H35	5449.48	9558.41	9289.33	53
H36	5451.49	11427.17	8649.44	65
H37	3880.36	12676.73	7557.94	59
H38	2259.18	12093.89	7108.52	58
H39	2227.8	10238.13	7764.26	48
H40A	3746.86	6065.91	11264.14	61
H40B	2201.24	6620.68	10998.48	61
H40C	2924.71	7309.17	11368.56	61
H43	2679.03	3588.92	11495.09	69
H44	2987.07	2547.7	10335.57	54
H45	3643.62	3764.46	8872.51	43

Table S27. Atomic Occupancies for all atoms that are not fully occupied in complex **2**

Atom	Occupancy
C19A	0.515(17)
C24A	0.515(17)
H24A	0.515(17)
C23A	0.515(17)

Atom	Occupancy
H23A	0.515(17)
C22A	0.515(17)
H22A	0.515(17)
C21A	0.515(17)
H21A	0.515(17)
C20A	0.515(17)
H20A	0.515(17)
C19B	0.485(17)
C20B	0.485(17)
H20B	0.485(17)

Atom	Occupancy
C21B	0.485(17)
H21B	0.485(17)
C22B	0.485(17)
H22B	0.485(17)
C23B	0.485(17)
H23B	0.485(17)
C24B	0.485(17)
H24B	0.485(17)

Molecular docking.

The complexes of **1-5** with the biological macromolecules considered were modelled using Autodock Vina.[5] 3D model of 3'-CCACCCACTACCCTGGTTGGATGCTAATGT-5' dsDNA (prepared and energy minimized using Avogadro[6], the crystal structures of human serum albumin (PDB ID: 1AO6[7]), HMGR (PDB ID: 1HW8[8]), and proteasome catalytic subunits (PDB ID: 6RGQ[9]) were used as receptor the docking calculations. Grid boxes of 90 × 100 × 90 points (for DNA) and 130 × 130 × 130 points (for the proteins) with a spacing of 0.375 Å between the grid points were was centered on the macromolecules. Parameters optimized by other research group for analogous interactions[10] were used throughout.

Binding to BSA

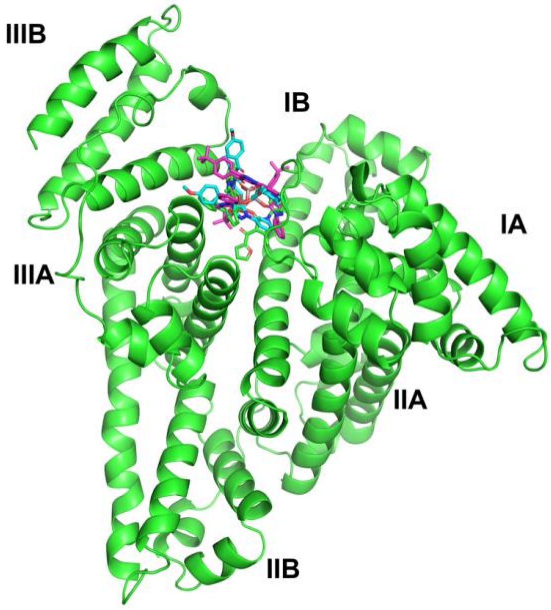

Figure S40. Superimposition of best scoring molecular docking models for complexes **1-5** binding to HSA (PDB ID: 1AO6).

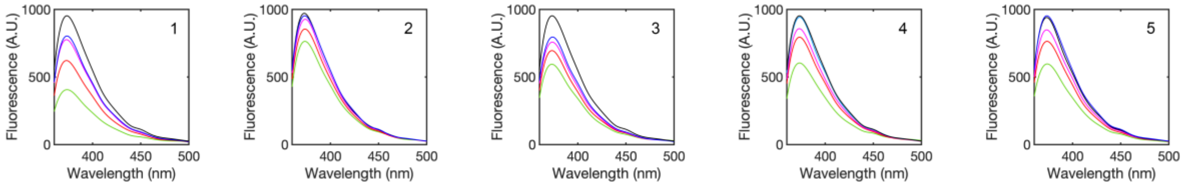

Figure S41. Changes in fluorescence emission spectra of BSA (black curves) upon titration with 1-5 in the range 0–10 μM (control, black line; 1 μM, blue line; 2 μM, purple line; 5 μM, red line; 10 μM, green line).

Table S28. Representative comparison of equilibrium and kinetic parameters for the interaction between complex **4** and BSA.

	k_{ass} ($M^{-1}s^{-1}$)	k_{diss} (s^{-1})	K_D (μM)
pH 6.8	3210 ± 667	0.036 ± 0.005	11.2 ± 1.6
pH 7.4	2350 ± 540	0.008 ± 0.003	3.4 ± 1.3

Binding to DNA

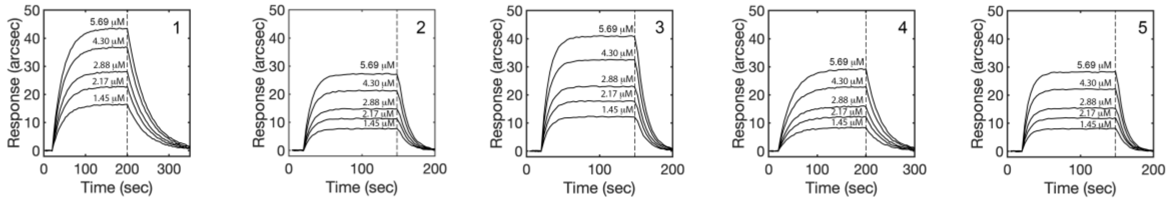

Figure S42. Comparison of mono-exponential binding kinetics for compounds **1-5** to surface-blocked DNA 30-mer.

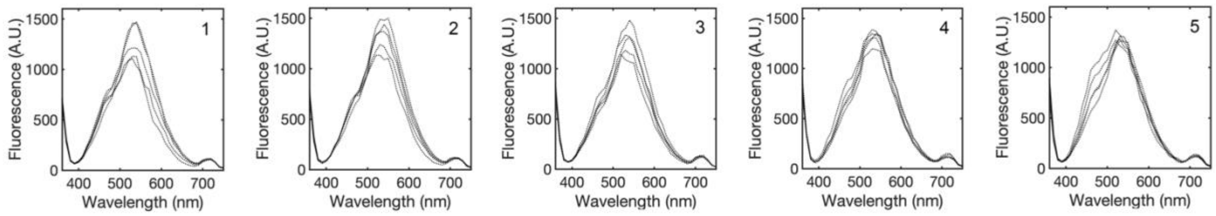

Figure S43. Changes in fluorescence emission spectra of DAPI-DNA complex upon titration with complexes **1-5** in the range 1-200 μM.

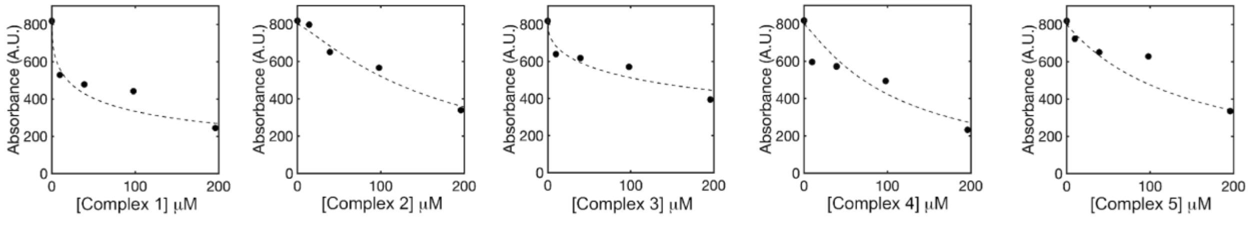

Figure S44. Changes in absorbance at 630 nm of Methyl green-DNA complex upon titration with complexes **1-5** in the range 1-200 μM.

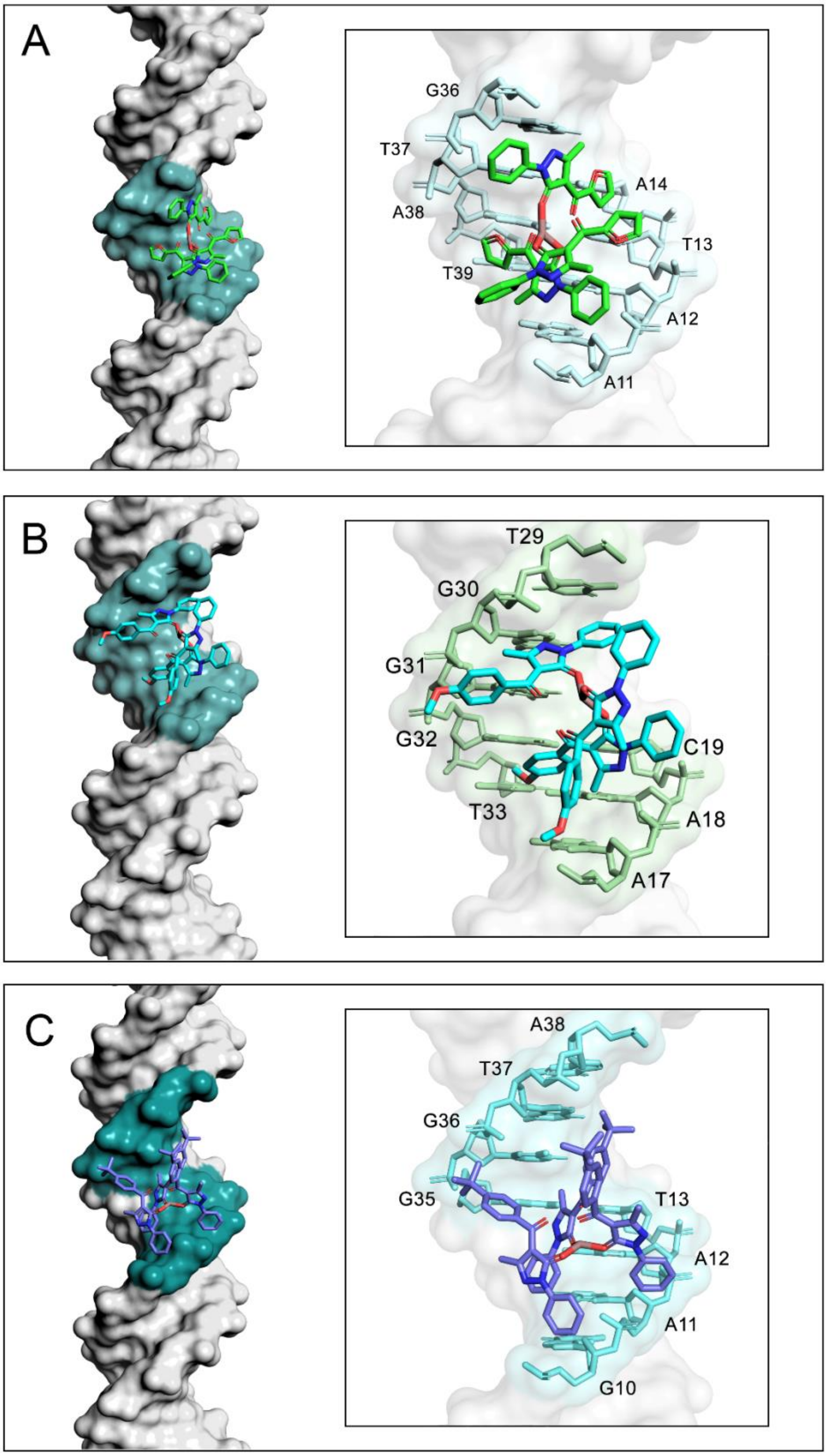

Figure S45. Comparison of the best scoring complexes formed upon docking **2** (Panel A), **4** (Panel B) and **5** (Panel C) on dsDNA (prepared and energy minimized using Avogadro). Major groove is emphasized as solid cyan surface (left panels).

Cell membrane permeability

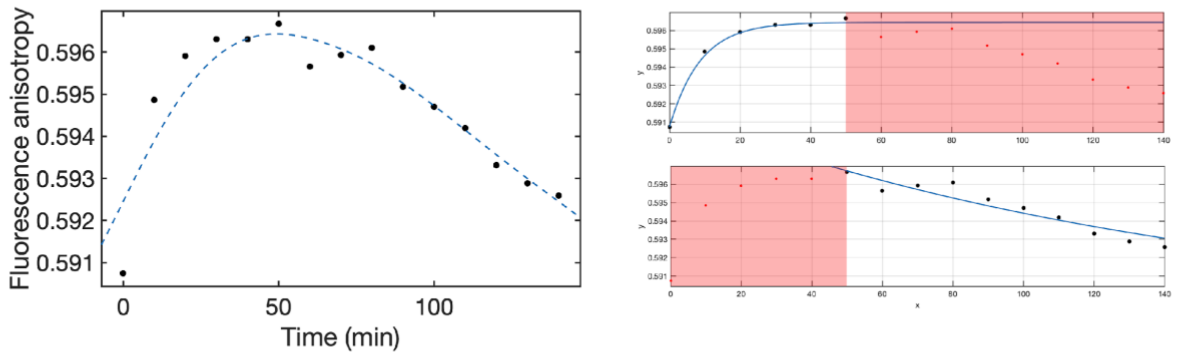

Figure S46. Visualization of changes in emission anisotropy with time observed upon Caco-2 cell membrane passage of **1** (left panel). Kinetic analysis of entry and release stages (right panel).

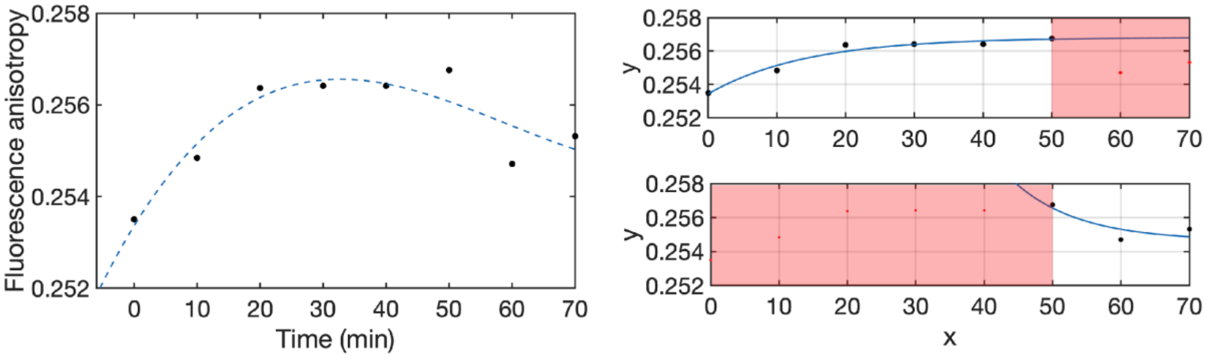

Figure S47. Visualization of changes in emission anisotropy with time observed upon Caco-2 cell membrane passage of **2** (left panel). Kinetic analysis of entry and release stages (right panel).

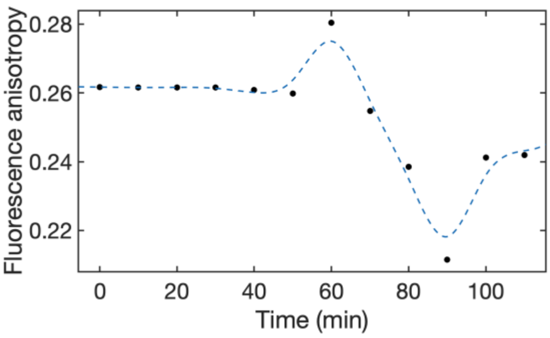

Figure S48. Visualization of changes in emission anisotropy with time observed upon Caco-2 cell membrane passage of **3**.

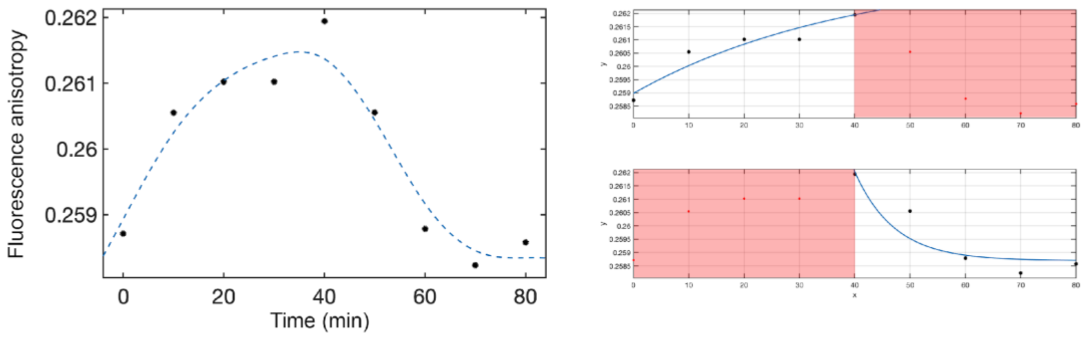

Figure S49. Visualization of changes in emission anisotropy with time observed upon Caco-2 cell membrane passage of **4** (left panel). Kinetic analysis of entry and release stages (right panel).

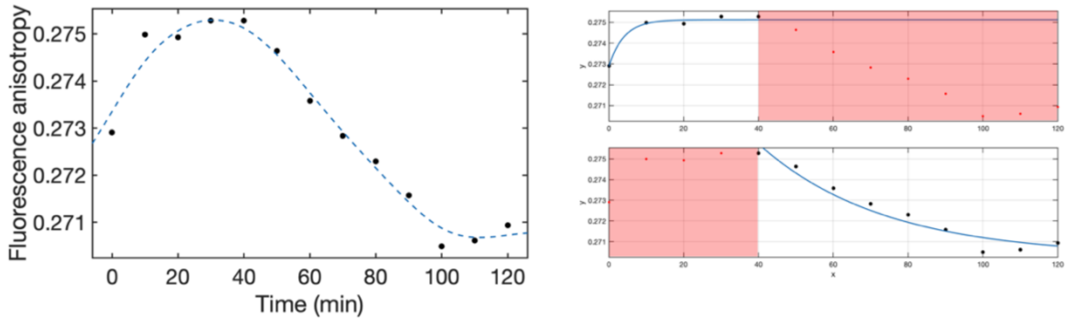

Figure S50. Visualization of changes in emission anisotropy with time observed upon Caco-2 cell membrane passage of **5** (left panel). Kinetic analysis of entry and release stages (right panel).

Table S29. Rate constants for membrane entry (k_{in}) and release from membrane (k_{out}) of complex **1**, **2**, **4** and **5**.

Complex	k_{in} ($M^{-1}s^{-1}$)	k_{out} ($M^{-1}s^{-1}$)
**1**	0.1192 ± 0.0102	0.0061 ± 0.0032
**2**	0.0701 ± 0.0082	0.1046 ± 0.0099
**4**	0.0257 ± 0.0076	0.1394 ± 0.0131
**5**	0.1791 ± 0.0085	0.0400 ± 0.0033

Table S30. Calculated lipophilicity of complexes **1-5** expressed as logarithm of octanol/water partition coefficient (Molinspiration Cheminformatics web services, https://www.molinspiration.com, Slovensky Grob, Slovakia).

Complex	logP
1	6.35
2	4.12
3	6.04
4	5.12
5	9.05

Binding to HMGR

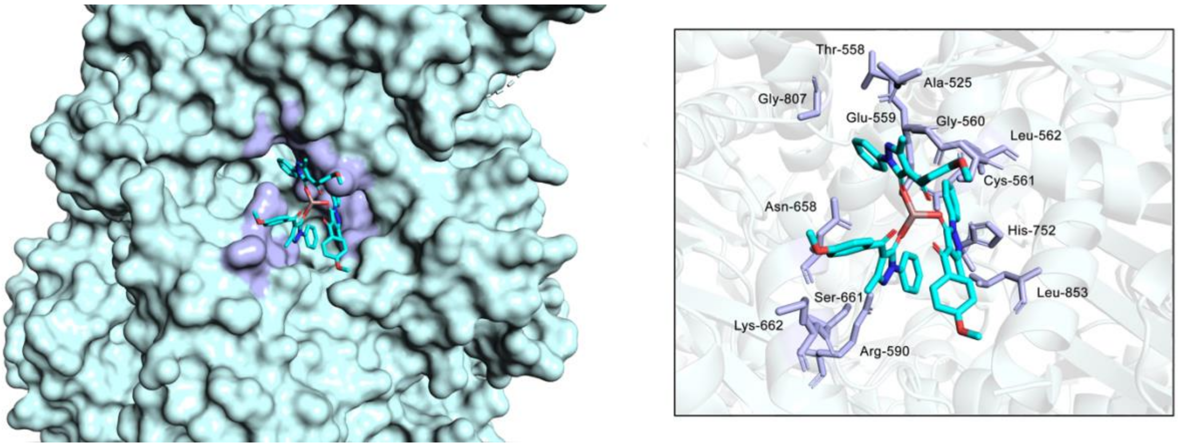

Figure S51. Molecular docking of the crystallographic structure of **4** within the catalytic region of HMG-CoA reductase (PDB ID: 1HW8). Surface, and cartoon and stick representations are reported in left and right panels, respectively. Residues involved in the formation of the complex are rendered as light violet.

SPR binding study.

HMGR was covalently immobilized onto a carboxylate surface as described elsewhere.[11] Briefly, the sensing surface was set at 37 °C, and rinsed with PBS (10 mM Na_2HPO_4, 2.7 mM KCl, 138 mM NaCl, pH 7.4), prior to the activation of carboxylic groups with an equimolar EDC/NHS solution.[12] HMGR was dissolved 10 mM CH_3COONa, pH 5.5 to a final concentration of 0.2 mg mL^{-1} and incubated over the surface for 20 min, Unreacted carboxylic groups of the biosensor surface were deactivated with 1 M ethanolamine. Next, 1 µM of complex 4 was added to HMGR in the presence and in the absence of saturating HMG-CoA and NADPH. Baseline recovery was always assessed before any new binding.

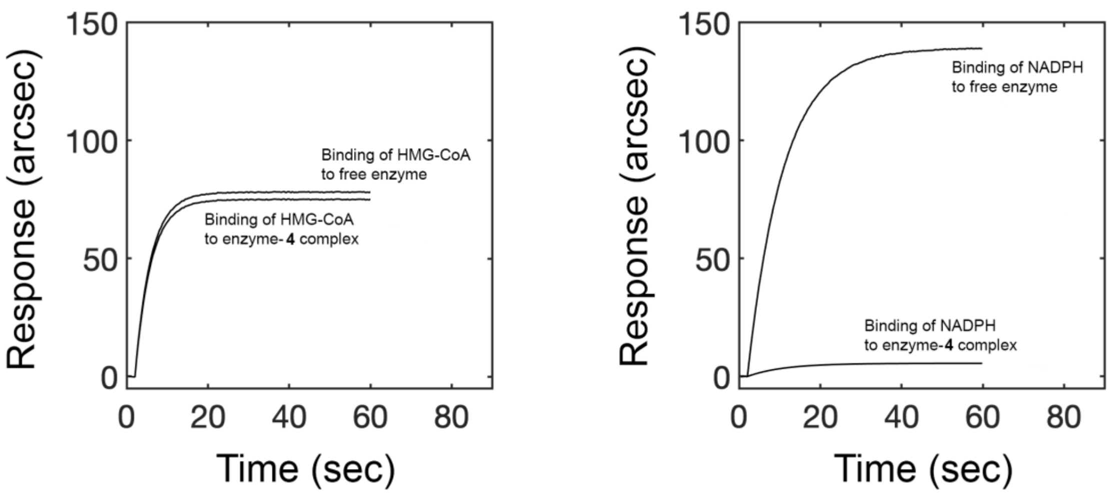

Figure S52. Competitive binding assay of complex **4** to HMGR. Comparative binding of **4** to surface-blocked HMGR in the presence and in the absence of HMG-CoA (left panel) and NADPH (right panel).

Effect on cell cycle

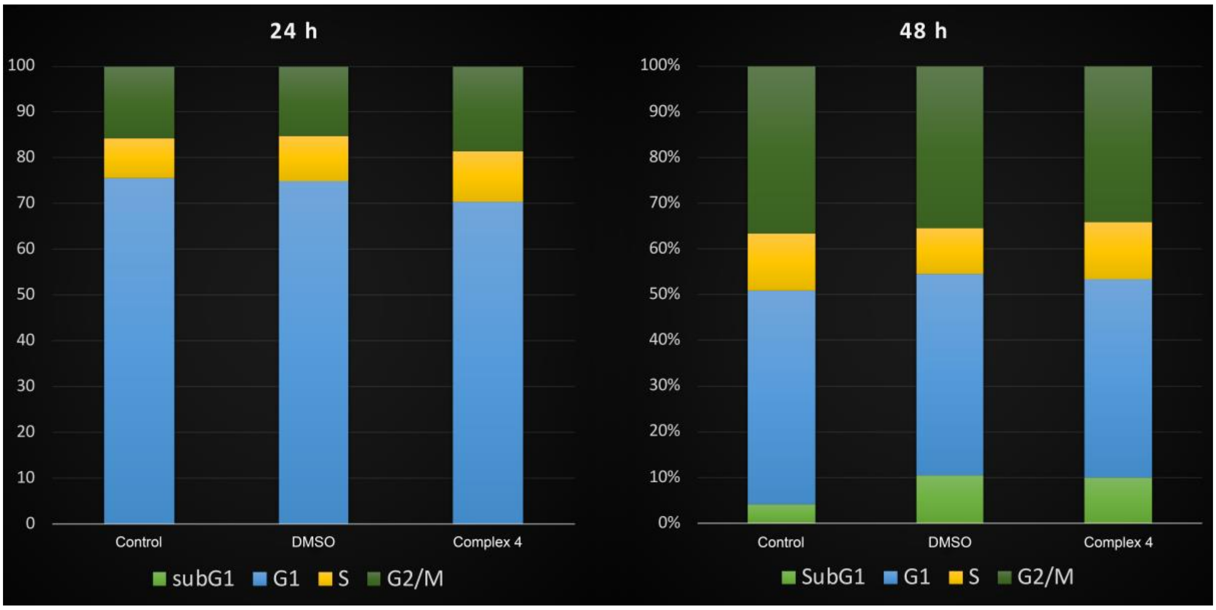

Figure S53. Effect of complex **4** on cell cycle distribution in Caco-2 cell

Effect on cytochrome p450 oxidase

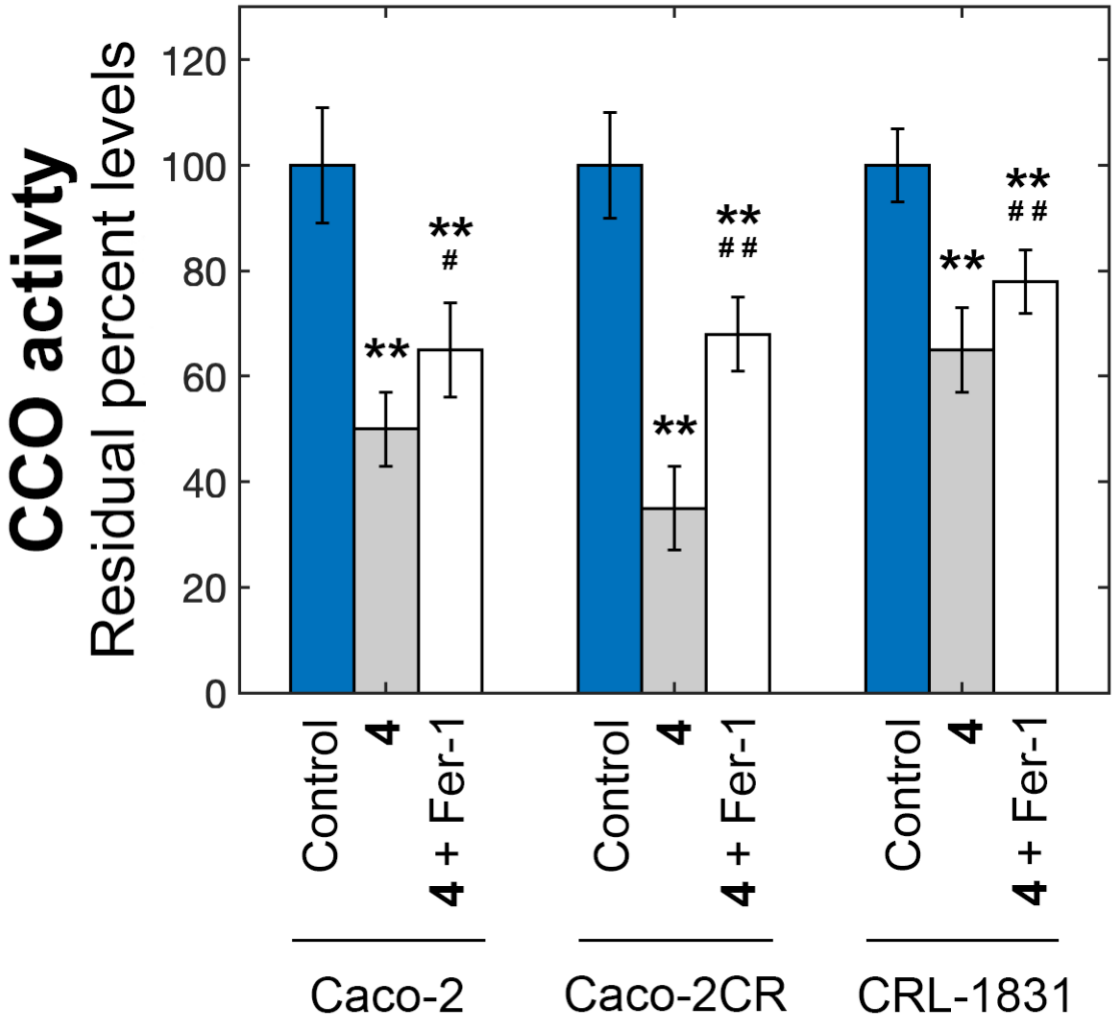

Figure S54. Increase in cytochrome p450 oxidase activity after 48 h treatment of Caco-2, Caco-2CR and CRL-1831 cells with complex **4** in the presence and in the absence of ferrostatin-1, calculated as percentage decrease in the levels of reduced cytochrome c (*p<0.05 and **p<0.01 compared with the control; #p<0.05 and ##p<0.01 compared with complex **4**).

Effect on 20S and 26S cellular proteasomes

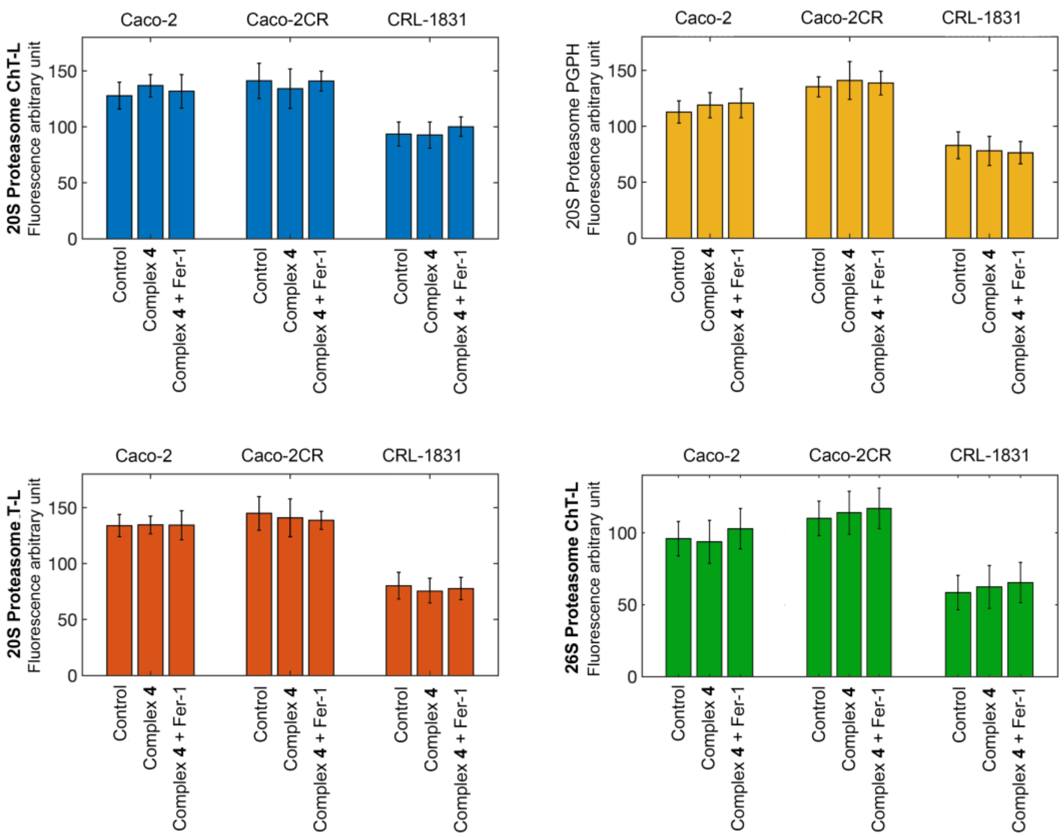

Figure S55. Effect of complex **4** on the main proteolytic activities of 20S proteasome after 48 h treatment of Caco-2, Caco-2CR and CRL-1831 cells in the presence and in the absence of Fer-1.

Effect of ferrostatin-1 on complex 4 cytotoxicity

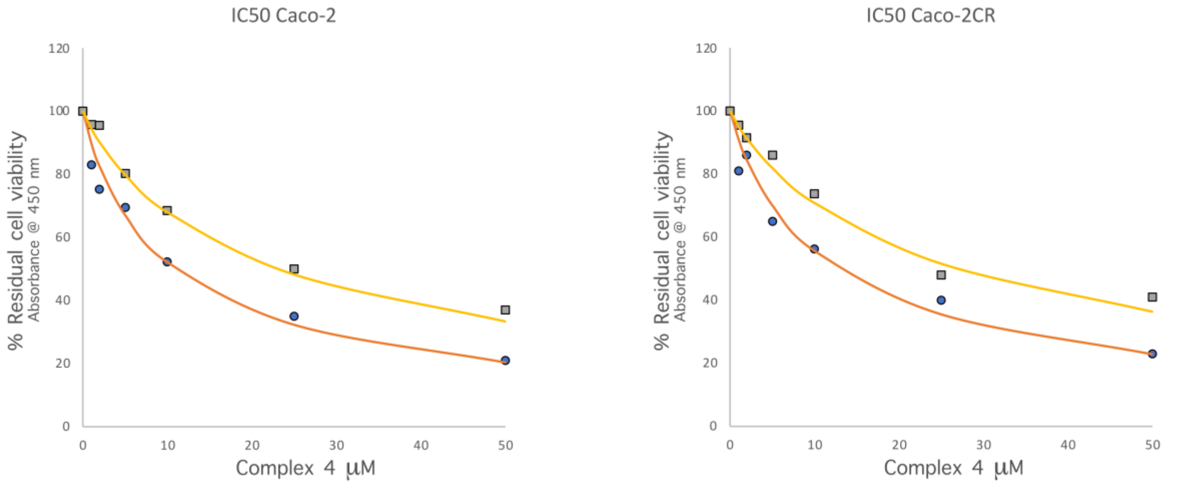

Figure S56. Cytotoxic effect of different concentrations of complex **4** on Caco-2 (Left Panel) and on Caco-2CR (Right Panel) cells viability in the absence (●, orange line) and in the presence (■, yellow line) of 60 nM of ferrostatin.

Effect of ferrostatin-1 on cellular redox homeostasis

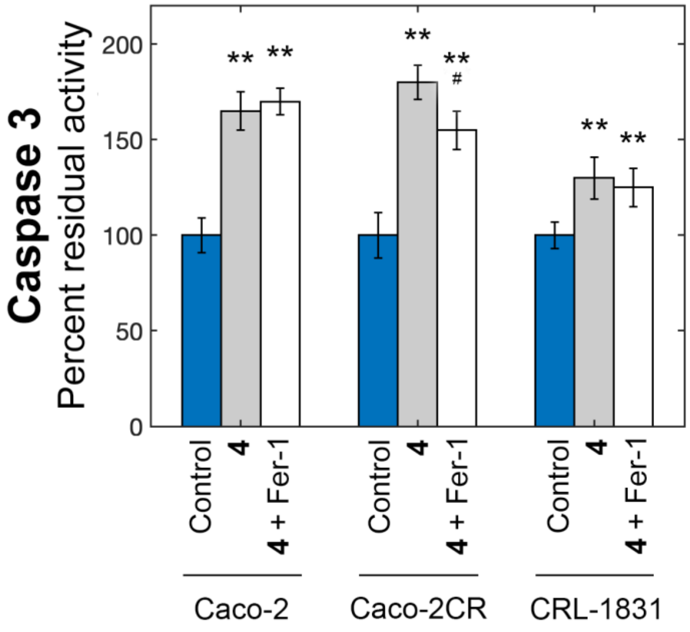

Figure S57. Changes in caspase 3 activity upon treatment of Caco-2, Caco-2CR and CRL-1831 cells with complex **4**, in the presence and in the absence 60 nM of ferrostatin (*p<0.05 and **p<0.01 compared with the control; #p<0.05 and ##p<0.01 compared with complex **4**).

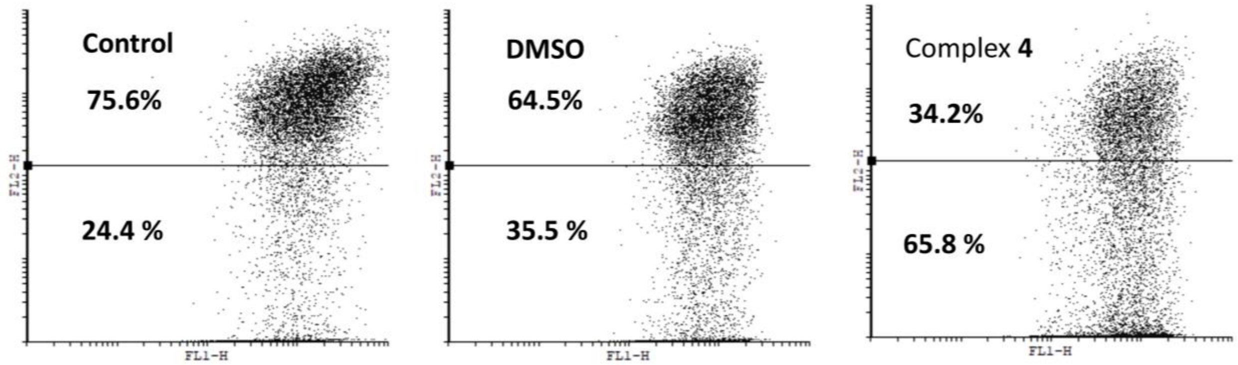

Figure S58. Analysis of ΔΨm changes in Caco-2 cell line treated for 48 h with medium, vehicle, and 10 μM of complex **4** evaluated by JC-1 staining and biparametric FL1(green)/FL2(red) flow cytometric analysis. Numbers in the bottom right square indicate the percentage of cells showing a drop in ΔΨm-related red fluoresce intensity. Data are representative of two separate experiments.

Purity evaluation

RP-HPLC was used for final purity assessment upon synthesis of complexes **1**-**5**. The complexes were dissolved in DMSO and filtered with 20 μm filters. Chromatographic analyses were run on an AKTA HPLC system equipped with a Kinetex® C18 column (2.6 μm, 100 × 4.6 mm), using a linear gradient from 10-90% mobile phase B in 3 min (mobile phase A: 0.1% TFA in H_2O; mobile phase B: 0.1% TFA in CH_3CN). Flow rate: 1.0 mL/min.

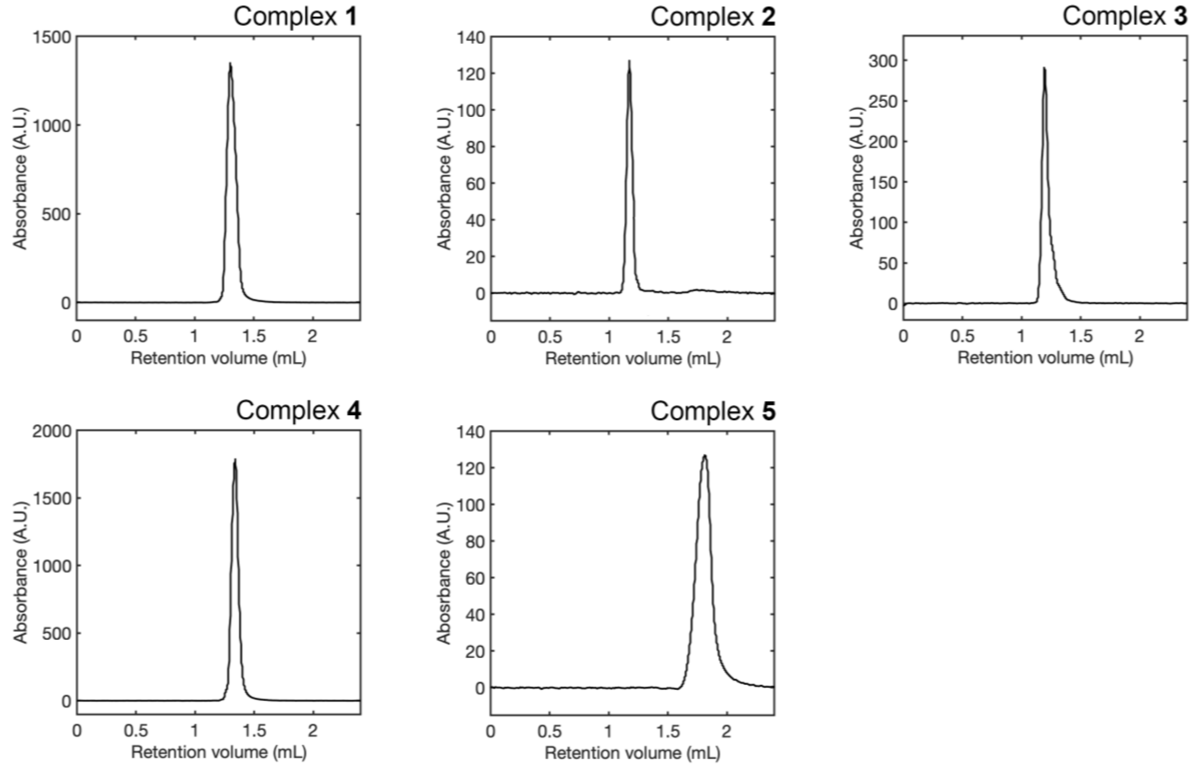

Figure S59. HPLC chromatographic profiles of complexes **1-5**.

Table S31. HPLC analysis of complex **1.**

Retention (ml)	Area (mAU*ml)	Height (mAU)
-0,03	0,101	2,197
0,02	0,0218	0,881
0,07	0,0266	0,906
0,09	0,0178	0,764
0,15	0,0363	0,985

0,2	0,0289	0,876
0,28	0,0224	0,664
0,33	0,013	0,742
0,41	0,0181	0,923
0,53	0,0136	0,631
0,84	0,0163	0,675
1,17	0,1505	3,917
1,3	125,0153	1351,732
1,7	0,0685	1,981
1,75	0,0452	1,281
1,79	0,0322	1,005
1,82	0,0178	0,676
1,85	0,0191	0,992
2,32	0,0146	0,601
2,37	0,0168	0,839

**Total peak area**	125,6958
**Purity (% area)**	99,46

Table S32. HPLC analysis of complex **2.**

Retention (ml)	Area (mAU*ml)	Height (mAU)
-0,04	0,0269	1,592
0,2	0,0179	0,575
0,3	0,0226	0,825
0,42	0,0186	0,693
0,52	0,0194	0,72
0,56	0,0207	0,59

0,74	0,031	1,161
0,91	0,0172	0,659
0,95	0,0189	0,656
1,06	0,023	0,672
1,17	17,5437	127,385
1,39	0,0321	2,773
1,69	0,0315	1,127
1,72	0,0385	1,464
1,74	0,0234	1,723
1,78	0,0293	1,492
1,8	0,0348	1,336
1,84	0,0361	1,152
1,87	0,0234	0,931
2,01	0,0172	0,655

**Total peak area**	9,0262
**Purity (% area)**	97,35

Table S33. HPLC analysis of complex **3**.

Retention (ml)	Area (mAU*ml)	Height (mAU)
0,04	0,1426	2,421
0,1	0,0882	2,509
0,13	0,0441	2,308
0,16	0,0585	2,092
0,18	0,0344	1,823
0,21	0,0552	1,978
0,24	0,091	1,951
0,28	0,0307	1,802

0,3	0,0408	1,939
0,33	0,0755	2,026
0,36	0,0636	1,813
0,42	0,0346	1,314
0,46	0,0955	1,764
0,58	0,0469	1,353
0,64	0,0302	1,329
0,68	0,0325	1,027
0,74	0,035	1,089
0,89	0,0374	0,853
1,19	21,4746	292,097
1,55	0,0435	0,823

**Total peak area**	22,5548
**Purity (% area)**	95,21

Table S34. HPLC analysis of complex **4**.

Retention (ml)	Area (mAU*ml)	Height (mAU)
-0,05	0,0324	0,877
0,04	0,0404	0,871
0,09	0,0165	0,626
0,14	0,0184	0,646
0,25	0,0169	0,82
0,37	0,0171	0,549
0,42	0,0261	0,863
0,51	0,0159	0,9
0,6	0,0267	0,668
0,68	0,0224	0,666

1,17	0,0599	1,449
1,34	131,7192	1792,606
1,67	0,0853	2,233
1,74	0,0553	1,139
1,78	0,0163	1,021
1,81	0,0196	0,88
1,94	0,022	0,611
2,06	0,021	0,703
2,17	0,0157	0,477
2,35	0,0269	0,727

**Total peak area**	132,274
**Purity (% area)**	99,58

Table S35. HPLC analysis of complex **5**.

Retention (ml)	Area (mAU*ml)	Height (mAU)
0,01	0,0347	1,205
0,04	0,0271	1,203
0,07	0,0371	0,942
0,14	0,0442	0,867
0,22	0,0225	0,705
0,32	0,0249	0,885
0,4	0,0215	0,837
0,52	0,0399	0,793
0,63	0,0241	0,705
0,68	0,0314	0,828
1,15	0,0817	3,011
1,17	0,087	3,124

1,26	0,0899	4,576
1,4	0,4073	7,07
1,81	28,5473	127,142
2,07	0,0679	1,157
2,16	0,0647	1,837
2,2	0,0221	1,258
2,22	0,0346	1,349
2,29	0,0246	0,862

**Total peak area**	21,7345
**Purity (% area)**	96,01

REFERENCES

(1) Sheldrick, G. M. SHELXT - Integrated Space-Group and Crystal-Structure Determination. *Acta Crystallogr. Sect. A* **2015**, *71* (1), 3–8.

(2) Dolomanov, O. V; Bourhis, L. J.; Gildea, R. J.; Howard, J. A. K.; Puschmann, H. Olex2: A Complete Structure Solution, Refinement and Analysis Program. *J. Appl. Crystallogr.* **2009**, *42* (2), 339–341.

(3) Sheldrick, G. M. Crystal Structure Refinement with SHELXL. *Acta Crystallogr. Sect. C Struct. Chem.* **2015**, *71* (1), 3–8.

(4) CrysAlisPro Software System. Rigaku Oxford Diffraction 2021.

(5) Eberhardt, J.; Santos-martins, D.; Tillack, A. F.; Forli, S. AutoDock Vina 1.2.0: New Docking Methods, Expanded Force Field, and Python Bindings. *J. Chem. Inf. Model.* **2021**, 1–7.

(6) Hanwell, M. D.; Curtis, D. E.; Lonie, D. C.; Vandermeersch, T.; Zurek, E.; Hutchison, G. R. Avogadro: An Advanced Semantic Chemical Editor, Visualization, and Analysis Platform. *J. Cheminform.* **2012**, *4* (1), 17.

(7) Sugio, S.; Kashima, A.; Mochizuki, S.; Noda, M.; Kobayashi, K. Crystal Structure of Human Serum Albumin at 2.5 A Resolution. *Protein Eng.* **1999**, *12* (6), 439–446.

(8) Istvan, E. S.; Deisenhofer, J. Structural Mechanism for Statin Inhibition of HMG-CoA Reductase. *Science* **2001**, *292* (5519), 1160–1164.

(9) Toste Rêgo, A.; da Fonseca, P. C. A. Characterization of Fully Recombinant Human 20S and 20S-PA200 Proteasome Complexes. *Mol. Cell* **2019**, *76* (1), 138-147.e5.

(10) Beckford, F. A.; Brock, A.; Gonzalez-Sarrías, A.; Seeram, N. P. Cytotoxic Gallium Complexes Containing Thiosemicarbazones Derived from 9-Anthraldehyde: Molecular Docking with Biomolecules. *J. Mol. Struct.* **2016**, *1121*, 156–166.

(11) Cuccioloni, M.; Mozzicafreddo, M.; Spina, M.; Tran, C. N.; Falconi, M.; Eleuteri, A. M.; Angeletti, M. Epigallocatechin-3-Gallate Potently Inhibits the in Vitro Activity of Hydroxy-3-Methyl-Glutaryl-CoA Reductase. *J. Lipid Res.* **2011**, *52* (5), 897–907.

(12) Edwards, P. R.; Lowe, P. A.; Leatherbarrow, R. J. Ligand Loading at the Surface of an Optical Biosensor and Its Effect upon the Kinetics of Protein-Protein Interactions. *J. Mol. Recognit.* **1997**, *10* (3), 128–134.
